# A tiled amplicon protocol for culture-free whole-genome sequencing of *M. tuberculosis* from clinical specimens

**DOI:** 10.1128/jcm.01823-25

**Published:** 2026-02-09

**Authors:** Chaney C. Kalinich, Freddy L. Gonzalez, Alice Osmaston, Mallery I. Breban, Isabel Distefano, Candy Leon, Jorge Coronel, Grace Tan, Valeriu Crudu, Nelly Ciobanu, Alexandru Codreanu, Walter Solano, Jimena Ráez, Patricia Sheen, Mirko Zimic, Orchid M. Allicock, Chrispin Chaguza, Anne L. Wyllie, Matthew Brandt, Daniel M. Weinberger, Benjamin Sobkowiak, Ted Cohen, Louis Grandjean, Nathan D. Grubaugh, Seth N. Redmond

**Affiliations:** 1 Department of Epidemiology of Microbial Diseases, Yale School of Public Health 50296 , New Haven, Connecticut, USA; 2 Department of Ecology and Evolutionary Biology, Yale University 5755, New Haven, Connecticut, USA; 3 Department of Infection, Immunity, and Inflammation, Institute of Child Health, University College London, London, England; 4 Universidad Peruana Cayetano Heredia 33216, Lima, Peru; 5 Institute of Phthisiopneumology, Chisinau, Moldova; 6 Yale Institute for Global Health, Yale University, New Haven, Connecticut, USA; 7 Public Health Modeling Unit, Yale School of Public Health 50296 , New Haven, Connecticut, USA; University of Manitoba, Winnipeg, Manitoba, Canada

**Keywords:** genomic epidemiology, amplicon sequencing, pathogen genomics, *Mycobacterium tuberculosis*

## Abstract

Whole-genome sequencing of *Mycobacterium tuberculosis* can be a valuable tool for TB surveillance and treatment, providing insights into transmission patterns and comprehensive drug susceptibility testing. However, the slow growth of *M. tuberculosis* means traditional culture-based sequencing methods can take weeks to return results, which has limited the widespread adoption of these techniques and limited their use in clinical decision-making. Tiled amplicon sequencing is a fast, reliable, and cost-effective method of whole-genome sequencing that can be done directly on clinical specimens and has been implemented at scale in academic and public health laboratories across the world; it was the cornerstone of SARS-CoV-2 sequencing and has been adapted for a wide range of viral pathogens. However, similar methods are not yet available for far larger bacterial genomes. Extending this approach to *M. tuberculosis* would significantly reduce the cost, labor, and turnaround time for whole-genome sequencing. We designed a tiled amplicon panel consisting of 5,128 primers that covers the entire *M. tuberculosis* genome, the largest tiled amplicon sequencing panel we are aware of to date. Applying our amplicon panels to clinical samples of sputum, we show the ability to recover whole-genome bacterial sequences without the need for culture. The resulting sequence data can be used to determine *M. tuberculosis* lineage and reliably identify markers of drug resistance. Using this approach in clinical settings could reduce the time needed for comprehensive drug susceptibility testing from weeks to days and enable genomic epidemiology to be performed at scale, even in resource-limited settings.

**IMPORTANCE:**

We have developed and tested an amplicon panel, TB-seq, for the priority pathogen *Mycobacterium tuberculosis*, demonstrating recovery of near-full genomes directly from patient sputum, including mixed and low-concentration samples. This approach significantly reduces the turnaround time for this slow-growing bacterium while maintaining high accuracy in detecting clinically relevant mutations, including those associated with drug resistance. Given the global burden of tuberculosis and the critical need for faster diagnostic solutions, we believe our method has the potential to improve clinical decision-making and public health strategies.

## INTRODUCTION

Tuberculosis (TB) is the world’s leading cause of death from a single infectious agent, resulting in more than a million deaths per year (1). COVID-19-related disruptions in disease detection and treatment were responsible for approximately 700,000 excess TB deaths from 2020 to 2023 (1), and worldwide cases have continued to increase over the past 2 years (2).

However, the pandemic also saw impressive advances in the use of viral whole-genome sequencing (WGS) to identify viral lineages (3) and track disease spread on both global (4) and local scales (5), enabling real improvements in disease control (6).

Applying similar approaches to TB could make up for recent lost ground. Indeed, the application of WGS to inform TB control is far from a new idea, having been shown more than a decade ago to be able to reconstruct fine-scale transmission networks (7), and shows particular value in detecting superspreading (8), distinguishing recrudescence from reinfection (9), and has been used to characterize transmission dynamics at a national scale (10, 11). Perhaps more importantly, given the complexity of TB treatment regimes and the high global prevalence of drug resistance, WGS of *Mycobacterium tuberculosis* offers unique advantages for detecting antimicrobial resistance (12, 13), enabling tailored treatment regimens (14). Genotypic predictions of TB drug resistance have been shown to be highly accurate (15, 16).

While routine WGS of *M. tuberculosis* has been adopted for national TB surveillance in the UK, Italy, and the USA, it has not been implemented at scale outside of high-income countries (17, 18). This is, in large part, due to the challenges of reliably sequencing patient specimens: direct sequencing from patient sputum does not consistently produce sufficient data for resistance prediction or epidemiologic investigation (19, 20). Enrichment approaches such as hybrid capture have been developed to enable direct sequencing of *M. tuberculosis* from sputum (21), though high library preparation costs and a laborious protocol have prevented them from being widely adopted.

Reliable WGS can be achieved with the addition of a prior culturing step, but imposes delays of up to 6 weeks (22) and significantly increases the risk of airborne contagion: while sputum samples, having high viscosity and low bacterial load, can be handled in many local health centers, bacterial culture is typically handled in regional reference laboratories (23). The culturing process itself may also introduce discrepancies, with loss of diversity following culturing having been reported in some (24, 25), but not all, cases (26), a finding that may impair detection of heteroresistance.

Rapid drug susceptibility can be achieved by targeted assays such as nucleic acid amplification tests (NAATs) or line-probe assays (LPAs) within 1–2 days, but these are limited in the number of loci they can cover. NAATs work well for common rifampicin resistance but show poor sensitivity for second-line drugs (27), while LPAs offer high sensitivity to both first- and second-line drugs (28, 29) but perform poorly where secondary resistance mechanisms are at high prevalence (30). The World Health Organization (WHO) continues to recommend additional phenotyping via liquid culture assay for suspected extremely drug-resistant (XDR) samples (31). None of these approaches to drug susceptibility testing enable the bacterial lineage to be identified or transmission networks to be inferred.

While genotyping approaches such as genotyping restriction fragment length polymorphisms and variable number tandem repeats can identify TB lineages, both are hampered by the low genomic diversity of *M. tuberculosis*, meaning they are insufficient to infer transmission in many circumstances (22). In recent years, the WHO has also recommended assays that use a targeted amplicon approach to sequence resistance-associated loci for first- and second-line drugs (32). Encouragingly, these have been successfully implemented in resource-limited settings (33); however, the targeted approach is not suitable for assaying resistance markers outside of the targeted regions or for the discovery of novel resistance loci. In comparison, WGS is able to simultaneously assay all 450 SNPs that have shown association or preliminary association with drug resistance (16), making it particularly well suited for tailoring treatment regimens for highly drug-resistant TB (34).

The dramatic scale-up in WGS of viral pathogens was achieved using tiled amplicon sequencing. Initially developed for genomic surveillance during the 2016 Zika epidemic (35), where low viremia had precluded direct sequencing of clinical samples even where the infection had been confirmed, tiled amplicon sequencing uses multiplex PCR of tiled overlapping regions of a target genome to recover whole genomes from samples of low concentration or complex microbial communities. This has proven particularly useful for sequencing remnant samples from diagnostic tests. Its use in the “ARTIC” protocol for sequencing SARS-CoV-2 (36) has led to its deployment in thousands of public health laboratories around the world, facilitating true global surveillance of viral dynamics (6). The ease, reliability, and low cost of amplicon sequencing have seen its adaptation to a broad range of viral pathogens both in respiratory disease (37, 38) and beyond (39, 40). Adapting these techniques to bacterial pathogens could allow rapid culture-free sequencing from low input volumes, at large scale, and with minimal infrastructure requirements.

We present here TB-seq, a tiled amplicon panel for *M. tuberculosis* and the first use of amplicon-based WGS for a bacterial pathogen. This panel is able to generate complete genome coverage from samples with minimal input concentrations without the need for bacterial culturing. We show that TB-seq can be used to identify clinically relevant phenotypes, such as antimicrobial susceptibility, within days of sample collection and can detect resistance loci that were not found by rapid diagnostics. We anticipate that this work will not only generate opportunities for genomic epidemiology of *M. tuberculosis* but will also provide a roadmap for the development of amplicon sequencing for other clinically important bacterial pathogens.

## RESULTS

### Amplicon sequencing enables recovery of *M. tuberculosis* whole genomes from clinical specimens without prior culturing

The TB-seq panel consists of 2,564 primer pairs generating two pools of 2 kb amplicons that are combined to achieve whole or near-whole coverage of the 4.4 Mb *M*. *tuberculosis* genome. Sequencing libraries were prepared using a standard amplicon sequencing workflow, with and without adding amplicon panel primers to allow comparison of amplified and unamplified DNA libraries. This approach was applied to cultured isolates, sourced from Moldova, and sputum samples from patients in Peru (Table S1).

**Fig 1:**
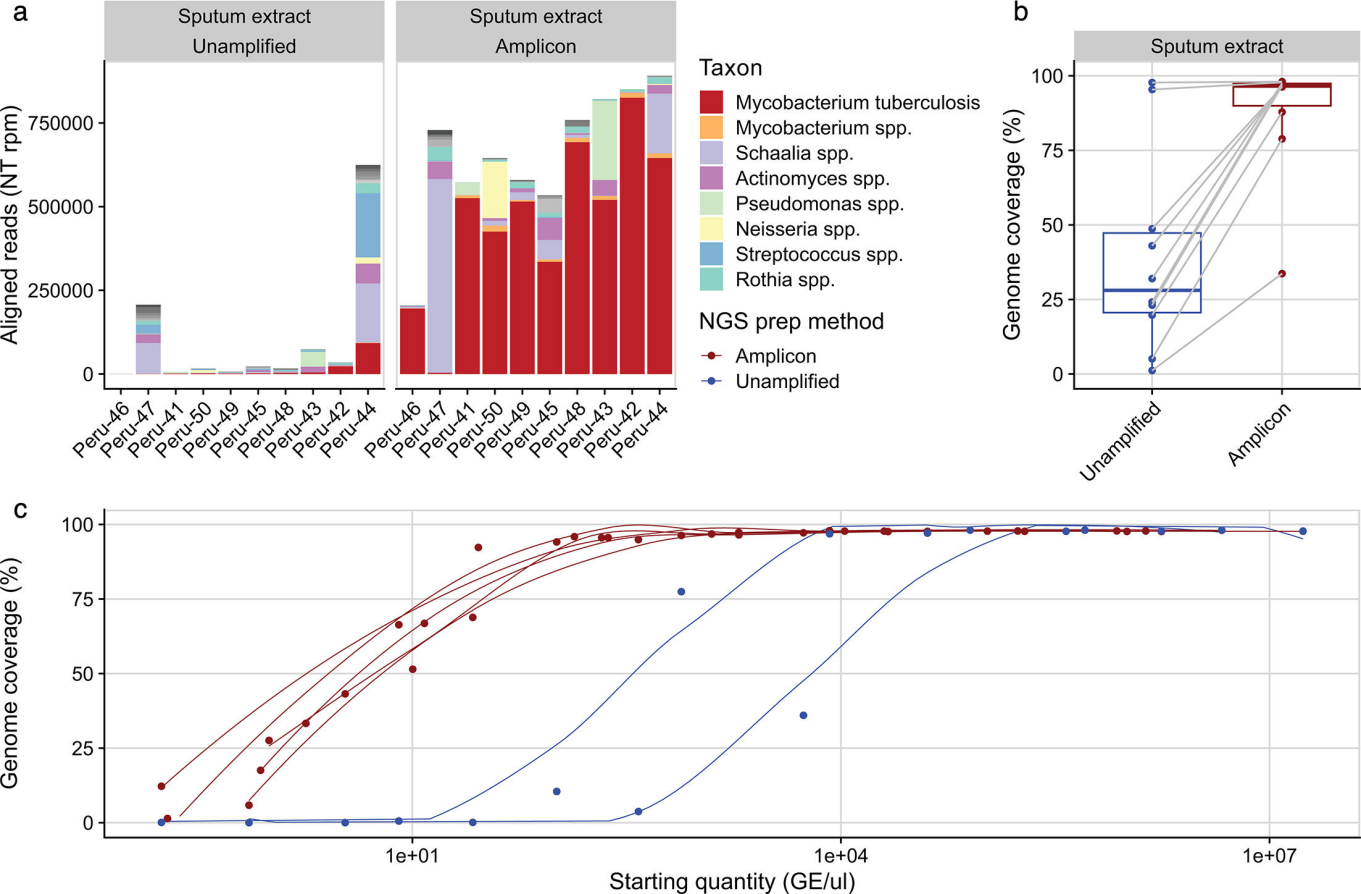
Tiled amplicon sequencing enables culture-free recovery of whole-genome sequences from *Mycobacterium tuberculosis* sputum specimens. We compared sequencing results for unamplified and amplified *M. tuberculosis* clinical specimens with regard to (**a**) microbial content via the CZID metagenomics pipeline and (**b**) average genome coverage. *M. tuberculosis*-positive sputum samples sequenced directly showed dramatic increases in genome coverage, with 8/10 samples generating more than 80% coverage after amplification with our protocol, and a ninth sample generating 78% coverage despite a significant infection with *Schaalia odontolytica*. Serial dilutions of DNA from cultured isolates (**c**) demonstrated that the amplicon scheme enables recovery of genomes with >95% coverage from all samples with ≥100 GE/μL, compared to ≥10,000 GE/μL without amplification.

We assessed the limits of detection for each amplicon panel by sequencing serial dilutions of six cultured isolates using both amplified and unamplified sequencing approaches. For *M. tuberculosis*, high genome coverage (>95%) was achieved in all amplified samples above 100 genome equivalents per microliter (GE/µL), compared to 10,000 GE/µL for unamplified samples (Fig. 1; Table S2).

Conversion of GE/µL in extracted DNA to sputum concentration is imprecise, with the concentration dependent on sample quality and extraction efficiency. For a specimen with 10^4^ AFB/mL (at which concentration 60% of samples are smear-positive [41]), 1 mL of sputum eluted into 50 µL (as used in our extraction protocols) would result in a DNA concentration of 10^4^ ÷ 50 = 2e^3^ GE/µL at 100% efficiency of extraction; we have used a 1e^3^ GE/µL (i.e., 50% efficiency) as our lower limit of extracted DNA concentration from smear-positive sputum. At this concentration, all of our dilution series achieved over 75% genome coverage using TB-seq (Fig. 1).

DNA was extracted from 60 sputum specimens with a range of acid-fast bacilli semi-quantitative measurements (e.g., 1+ to 3+). Several different DNA extraction methods were tested during optimization (methods A–E: detailed in Table S1, Figure S1b and c and Appendix S1). For the 10 sputum samples extracted with our optimized protocol (E), *M. tuberculosis*-positive sputum samples demonstrated dramatic increases in coverage for amplified samples when compared to their unamplified replicates (Fig. 1a through c). While only 2/10 of unamplified samples achieved more than 75% genome coverage, 9/10 of the amplified samples sequenced above this threshold, with 7 of those generating more than 95% coverage. The remaining sample achieved 33% coverage amplified and negligible coverage unamplified. Metagenomic sequencing indicated successful amplification from samples containing both commensal and pathogenic bacteria including *Streptococcus, Pseudomonas, Actinomycetes,* and *Schaalia* spp.

The protocol is extremely cost-effective: while library preparation costs vary between sites, the per-sample cost for amplicon generation is just $19.07 per sample (Table S3).

### Direct sputum sequencing enables phylogenetic classification and detects markers of antimicrobial resistance

*M. tuberculosis* lineages were called with the Mykrobe package (v.0.13.0) (42), which assigned all samples to lineage 2 (sublineage 2.2.1) or 4. Mykrobe performed equally well when calling lineages for all high-coverage (>75%) samples, regardless of whether they were derived from cell culture or sputum. We constructed maximum-likelihood (ML) phylogenies using IQ-TREE (43) including all sequenced specimens and the broad reference set of *M. tuberculosis* sequences used for primer design (Fig. S2 and Appendix S2). In all cases, the primary lineage predicted by Mykrobe aligned with lineages from an ML tree, though in some cases, secondary lineages were predicted based on minor variants that did not concord with the ML tree.

**Fig 2:**
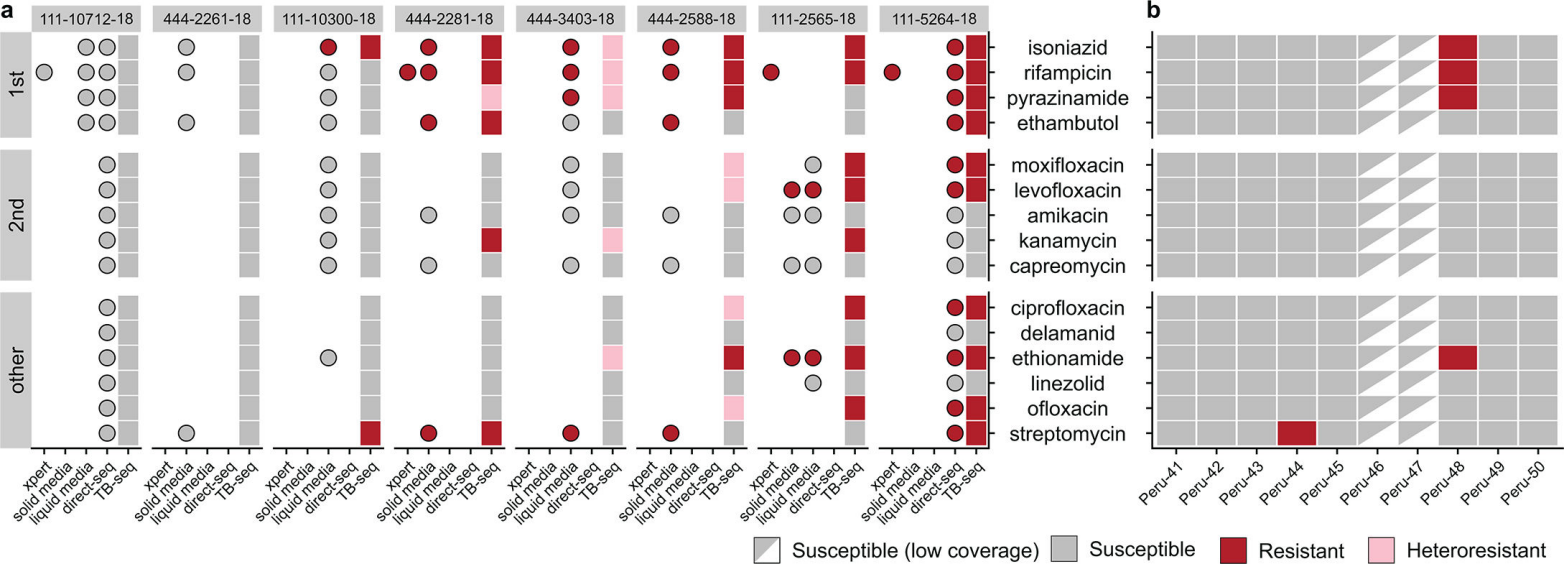
Amplicon sequencing predicts TB antimicrobial resistance *in silico*. Drug susceptibility to 15 anti-TB drugs was predicted by Mykrobe on amplicon-sequenced data for 8 colony samples (at 0.01 template dilution) and 10 sputum samples using our optimized extraction protocol. Colony samples (**a**) showed strong concordance with prior phenotyping on both liquid and solid media, enabling detection of both MDR and XDR samples. Sputum-derived samples (**b**) were able to generate susceptibility predictions for 8/10 samples, including at least one predicted MDR isolate (Peru-48).

Sequencing data were of sufficiently high quality to produce a drug susceptibility prediction for all template dilutions from cultured isolates with at least 10 GE/µL starting quantity (Fig. 2a; Table S4). Seven samples had been previously phenotyped at diagnostic centers in Moldova, via growth on liquid media (BD BACTEC mycobacterial growth indicator tubes [MGIT]) or solid media (Löwenstein–Jensen) using approved protocols (44). A further sample was assayed for rifampicin resistance using GeneXpert rapid testing (Xpert MTB/Rif, Cepheid, Sunnyvale, CA, USA), and two of our samples were also sequenced without amplification, and drug susceptibility predicted. In the overwhelming majority of cases (42/47) where a resistance phenotype was available, our susceptibility predictions were consistent with phenotyping.

Four discordant results were found: we failed to predict resistance for ethambutol and streptomycin in sample 444-2588-18 and failed to predict resistance to streptomycin in sample 444-3403-18, despite growth on medium containing these drugs. Resistance to moxifloxacin was predicted for sample 111-2565-18 despite phenotypic susceptibility. We also identified heteroresistance for three first-line drugs in one sample (444-3403-18) was found to be phenotypically fully resistant; the effects of growth in drug-free medium during maintenance cannot be determined.

When comparing unamplified sequencing and TB-seq on these samples, all resistance predictions were identical. While coverage in resistance-associated genes was typically extremely high (Table S4), lower coverage was seen in all samples in the rpsL gene associated with streptomycin resistance, suggesting discordant predictions may derive from poor sequencing coverage; further modifications to the amplicon panel may be necessary to reliably predict resistance for streptomycin.

Predictions were internally consistent for all template dilutions above 100 GE/µL (with some variability between samples called as heteroresistant or with fixed resistance alleles), with the exception of streptomycin, suggesting the potential for robust susceptibility calling using TB-seq at lower bacterial loads. While a comprehensive assessment of the sensitivity of resistance prediction is beyond the scope of this study, our results do suggest that TB-seq may be useful for rapid susceptibility prediction in public health settings.

Of the 60 extracted sputum specimens with all protocols, sequencing data were robust enough to produce a drug susceptibility prediction for 53/60 sputum specimens (Fig. S1a). Of the seven specimens which failed, four (Yale-TB121, Yale-TB123, Yale-TB149, Yale-TB150) had starting quantities following extraction below 10 GE/µL. None of the other three (Yale-TB126, Yale-TB139, Yale-TB148) had GeneXpert results available for comparison. Eight out of 10 specimens that were extracted with the best-performing protocol (Protocol E; see Appendix S1), which included a NALC-NaOH treatment to deplete non-mycobacterial DNA, had adequate data to predict resistance (Fig. 2b). At least one sample was predicted to be resistant to isoniazid, rifampicin, and pyrazinamide and would be classed as MDR-TB. We do not have access to phenotypic susceptibility results for those sputum samples; however, genotypic-based predictions of drug resistance in *M. tuberculosis* are usually highly accurate for most drug classes (15).

### *In silico* predictions indicate applicability of amplicon schemes to large bacterial genomes

To explore the limits of amplicon sequencing and assess performance on open and closed bacterial genomes, we assessed genome coverage *in silico* against a set of bacterial assemblies representative of global diversity.

**Fig 3:**
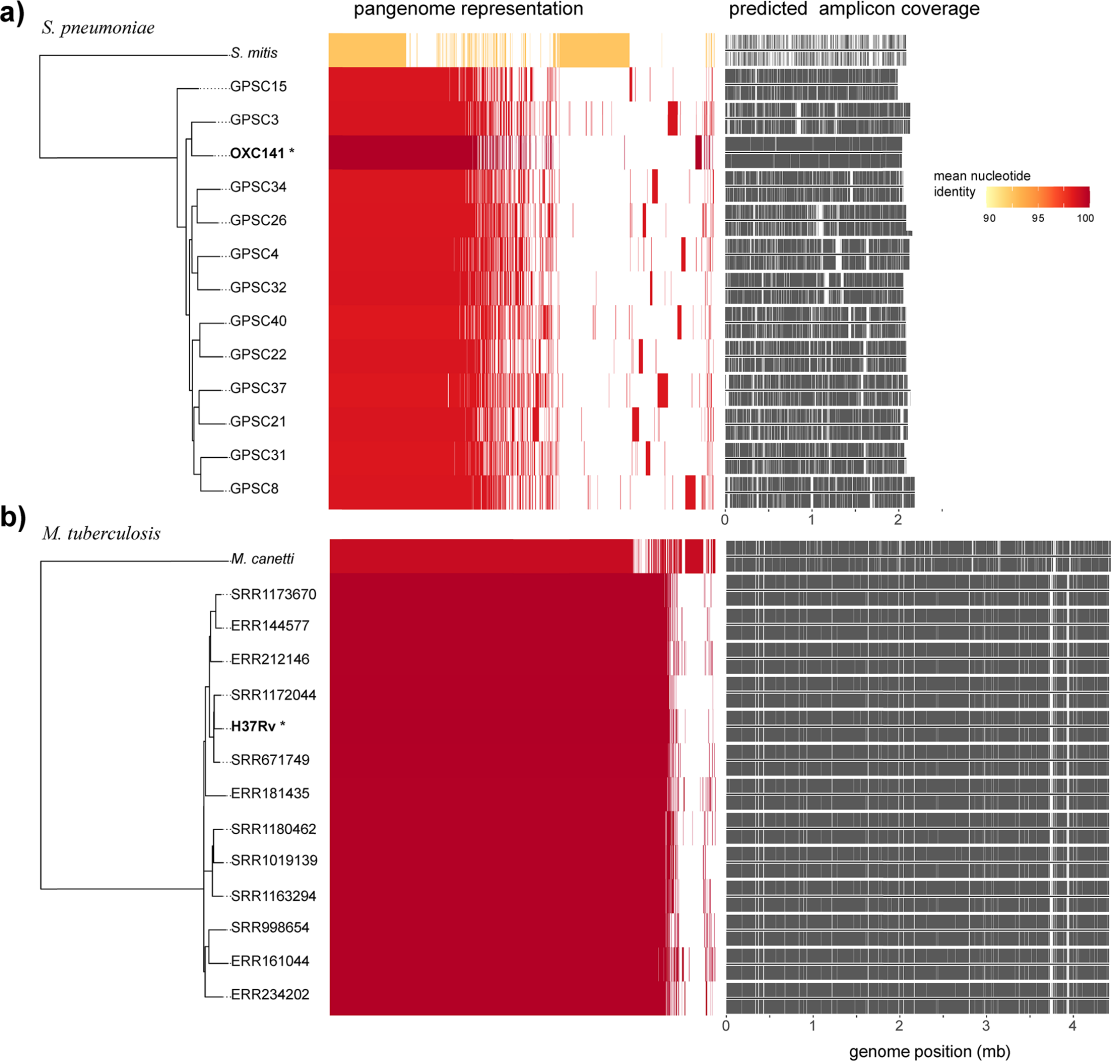
*In silico* modeling indicates broad applicability across diverse *Streptococcus pneumoniae* serotypes and *Mycobacterium tuberculosis* lineages. Pangenome representation of (**a**) *S. pneumoniae* whole-genome sequences (*n* = 13) and *Streptococcus mitis* outgroup (accession: AP023349) and (**b**) *M. tuberculosis* whole-genome sequences (*n* = 13) and *Mycobacterium canettii* outgroup (accession: NC_019950). Starred phylogenetic tree tips mark the reference sequences used for primer design. Shaded bar graphs (middle) denote genes shared among clades; the yellow-red color scale denotes average nucleotide identity. Predicted amplicon coverage (right) is shown in gray, with forward and reverse amplicon pairs displayed above and below the line. *M. tuberculosis* has a higher proportion of genes shared between clades and higher ANI than the open pangenome of *S. pneumoniae*, driving the higher predicted amplicon coverage. A list of the sequences used in this analysis can be found in Table S2.

We designed a comparable amplicon schema for *Streptococcus pneumoniae*, a prolifically recombinogenic pathogen with an open genome, and compared performance for both panels *in silico* across a representative set of closely related genomes (Fig. 3; Fig. S3b). *S. pneumoniae* has an average core genome size of 2,160,000 bp (45), requiring 2,292 primers for full coverage, while *M. tuberculosis*, with an average genome size of 4,411,000 bp (46), required 5,128 primers to amplify the entire genome.

For each bacterial species, we selected a small number of genetically diverse sequences from the larger set of publicly available sequences to predict coverage in related strains (Fig. 3; Table S5a). As expected, predicted amplicon coverage was highest against the strains used as a reference for panel design (*Sp*:OXC141: 98.93%; *Mt*/H37Rv: 94.31%). *M. tuberculosis* coverage is reduced due to the omission of PE/PPE regions from the design, which accounts for up to 8%–10% of its genome (12). However, predicted coverage in *M. tuberculosis* remained high across all members of the *M. tuberculosis* complex, including all seven major lineages, *Mycobacterium bovis* (≥94.23%), and *Mycobacterium canettii* (89.44%). In contrast, coverage of *S. pneumoniae* fell sharply between Global Pneumococcal Sequence Clusters (GPSCs) (<82.00%–>88.00%) and in the *Streptococcus mitis* outgroup (32.18%). The range of average nucleotide identity was narrower across *M. tuberculosis* (99.98%–99.27%) compared to the broader variation observed among *S. pneumoniae* (98.76%–92.51%), reflecting the diversity in the GPSC lineages included. Pangenome size and similarity were also markedly different between the two species, with *M. tuberculosis* having a smaller relative pangenome (4,335 total genes and a mean genome size of 4,067 genes, a ratio of 1.07, based on the 13 genomes included) than *S. pneumoniae* (3,942 total genes and a mean genome size of 2,071 genes*,* a ratio of 1.90, based on the 13 genomes included). As a result, far more sharing of genes with the reference strain was observed for *M. tuberculosis* (4,002–4,050 shared genes) than *S. pneumoniae* (1,705–1,793 shared genes). Our findings suggest that amplicon panels are tolerant to increasing genetic distance but negatively affected by genome rearrangements.

Amplicon predictions for expected coinfections and commensals indicated limited enrichment of off-target species (Fig. S4a and b; Table S5b).

## DISCUSSION

Tiled amplicon sequencing of pathogens has proven extremely useful for reconstructing disease spread and gaining insight into transmission patterns for a variety of viruses (47). The COVID-19 pandemic stimulated a global effort to adopt these methods and use genomics to track and monitor SARS-CoV-2; however, they have not previously been applied to the significantly larger and often more complex genomes of bacteria. We report the use of a tiled amplicon panel to sequence *M. tuberculosis,* generating whole-genome coverage from specimens with minimal input DNA and demonstrating the ability to identify lineages and markers of drug resistance. This is the first application of this technique to sequence a pathogenic bacterium, and the use of TB-seq in public health settings could have a major impact on TB control.

As of 2023, with *M. tuberculosis* reclaiming the title of most deadly single pathogen (1), there is a renewed focus on improving TB treatment and control. In particular, there are six vaccine candidates currently in phase III clinical trials (48) and continuing efforts to develop shorter and more effective treatment regimens (49). This may be an ideal time to apply tools used to control and monitor interventions for COVID-19 to TB (1).

Antimicrobial resistance is a critical issue in treating and controlling TB, due to the prevalence of resistance to first-line drugs and the length, cost, and complexity of treatment regimens (31). Despite the introduction of shorter regimens, the time taken to find an effective treatment can be long, and incomplete treatment remains a problem (50). For this reason, the WHO now recommends the use of targeted sequence-based diagnostics for rapid drug susceptibility testing for patients who are at high risk of, or have already experienced, treatment failure (32). However, designing such an assay is not simple; more than 40 separate loci, each containing numerous individual mutations, have been implicated in drug resistance (51), and uncertainty can be higher for new or second-line drugs (52). WGS works around these limitations of targeted amplicon sequencing. Unlike targeted sequencing approaches, data are being generated across the entire genome; therefore, as new genetic markers of resistance are discovered, these can be added bioinformatically without adding new primers. As per-sample primer costs are low (Table S3), the required time, infrastructure, and costs for tiled amplicon sequencing are almost identical to targeted amplicons; the additional data generated through WGS can be used along with phenotypic drug susceptibility to expand our understanding of the genetic markers of drug resistance, especially for third-line or novel drugs, increasing the accuracy of predictions over time (12).

WGS obviates the need to design a targeted assay and can also return resistance predictions within days of a positive culture. However, the requirement of most existing WGS approaches to first culture a sample means that the overall sample-to-sequence turnaround time for *M. tuberculosis* is measured in weeks or months (53), and significant biases can be introduced during the culturing process itself (12). WGS without culture has not consistently produced data of high enough quality for resistance prediction or thorough epidemiologic investigation (19, 20), or has been limited to specimens with a high bacterial load (20, 54); subsequent efforts to implement WGS have relied on expensive techniques such as hybrid capture (21, 55). For this reason, routine WGS still relies on prior culturing in MGIT to ensure reliable whole-genome recovery, both in public health settings (56–59) and ongoing trials for clinical application (60).

Our technique recovers whole or near-whole genomes directly from sputum, enabling lineage classification and providing genome coverage of many first- and second-line resistance loci, something that was not possible with prior WGS approaches (19–21, 53, 61). For a notoriously slow-growing organism such as *M. tuberculosis*, eliminating the culturing step reduces the time from sample collection to genome from weeks to days, meaning genomic epidemiology could be used in real-time to inform outbreak investigations (10, 62) and public health measures to reduce spread (11, 63).

Consistent with the *in silico* modeling prediction, TB-seq did not show higher rates of amplicon dropout when faced with targets that had drifted from the H37Rv reference strain. However, comparison with similar results for *S. pneumoniae* is instructive, with significant coverage gaps emerging even in clades closely related to the OXC141 reference strain. This suggests a weaker applicability of the tiling amplicon approach in species that undergo significant levels of recombination and horizontal gene transfer, and the ratio of genome to pangenome size is likely to be a key metric for our ability to design an amplicon panel. This ratio is highly sensitive to the diversity of habitats in which the pathogen is found: free-living or commensal species, such as *S. pneumoniae*, gain particularly large pangenomes to enable adaptation to diverse environments, while intracellular pathogens, such as *M. tuberculosis*, show strong purifying selection, low effective population sizes, and low genome-to-pangenome ratios (64). Other intracellular pathogens such as *Yersinia pestis*, *Listeria monocytogenes*, *Legionella pneumophila*, *Chlamydia trachomatis, Treponema pallidum, and Neisseria gonorrhoeae* may be suitable targets.

For bacteria with large pangenomes, designing tiling amplicon schemes will rely upon databases of previously sequenced genomes to determine circulating genetic diversity and to map horizontal exchange and genome rearrangements. The improving availability of vast databases of assembled genomes (65), as well as methods for bacterial genome graph construction (66) and recombination-aware graph aligners (67), suggests this could be a viable approach for high-priority pathogens.

Within our TB-seq panel, gaps remain in our coverage of the highly repetitive PE/PPE regions. While these are frequently omitted from *M. tuberculosis* analyses, increasing evidence of functions in host-cell invasion (68) and importance for vaccine design (69) suggests inclusion of these regions in future iterations of this amplicon panel would be an important improvement.

As all clinical specimens used were smear-positive, the usability of this technique with smear-negative (or paucibacillary) specimens was not determined. Approximately 60% of specimens with a bacterial load of 10^4^ AFB/mL are smear-positive (41); while the precise limit of detection of smear microscopy is dependent on sample quality, quantity, and user experience, our estimated DNA yield for such samples (1e3 GE/µL) is comfortably above the limit of applicability determined by serial dilution, suggesting that amplicon sequencing is likely to be successful with at least a subset of smear-negative specimens. We successfully recovered genomes classified as “low” by GeneXpert Ultra that are likely to consist of “scanty” smear-positive samples (70). Yet, with a limit of detection as low as 15 AFB/µL (71), GeneXpert Ultra will uncover smear-negative samples that cannot be sequenced with TB-seq, and direct sequencing of sputum is unlikely to be suitable as a diagnostic in the near future.

In the near term, our expected use case for this technology is to sequence confirmed, smear-positive cases for the purposes of genomic epidemiology. However, due to the importance of drug resistance to effective TB treatment, we wished to assess whether tiled amplicon sequencing could plausibly determine drug susceptibility. For the majority of our specimens (53/60), tiled amplicon sequencing from uncultured sputum was able to make accurate drug susceptibility predictions for all first- and second-line drugs (Fig. S1). Barriers to clinical application are necessarily higher (72): if diagnostics and resistance prediction are to be used to tailor treatment regimens, a comprehensive performance assessment will be necessary, including spike-ins with calibrated numbers of organisms and large numbers of drug-resistant samples. It will also be important to assess performance in a range of other likely scenarios: paucibacillary infections; mixed *M. tuberculosis* strains; mixed *M. tuberculosis* and nontuberculous mycobacteria; mixed resistant and susceptible strains. This would be a major undertaking: previous sensitivity assessments for sequence-based diagnostics have required many thousands of samples (73), and the use of sputum or live cultures would incur additional logistical challenges and significant biosafety risks.

Yet despite this complex landscape, prior experience suggests this technique could have a significant impact. The widespread use of tiled amplicon sequencing for pathogen genomics during the COVID-19 pandemic has ensured that this method is trusted, understood, and easily implemented in academic and public health laboratories worldwide. As the focus now turns to adapting this capacity to other public health threats (6), it is important to prioritize the development of tools for global priority pathogens that can be implemented in the regions suffering the greatest burden. The method presented here uses the same workflow as used by thousands of public health labs to sequence SARS-CoV-2, utilizing an off-the-shelf commercial sequencing library preparation kit with the principal change of swapping out primer pools used to generate amplicons. While the total cost of the primer pool is almost $10,000 at current prices, only a small amount is used per reaction, and the per-sample cost for library preparation is just $19.07 (Table S3). Genomic surveillance of *M. tuberculosis* has demonstrated capacity to guide TB interventions in high-income countries (8, 9); the reductions in cost and turnaround time afforded by tiled amplicon sequencing could enable this to be implemented in LMICs with high TB burden. Just four countries (India, Bangladesh, Indonesia, Democratic Republic of the Congo) account for over half of all TB deaths; all four have prior in-country experience with amplicon sequencing of SARS-CoV-2 (74–77), suggesting a ready capacity for tiled amplicon sequencing of *M. tuberculosis*. Extensive use of alternative sequencing methods such as Oxford Nanopore in these regions (74, 76, 78) suggests adaptation to cheaper and more portable sequencing platforms may further increase surveillance capacity.

If successfully implemented in a clinical environment, the capacity for culture-free *M. tuberculosis* sequencing to provide rapid lineage determination and resistance profiling could be transformative—not only by increasing rates of successful TB treatment at the patient level (79), but by preventing the further transmission of MDR-TB at the population level (80, 81). With several new TB vaccines in phase III trials, WGS can also provide lineage-specific estimates of vaccine efficacy and early signals of vaccine escape. Optimizing the use of available antimicrobials and vaccines, both old and new, is vital to improve TB control, and culture-free WGS can be used to ensure the right interventions are reaching the right populations in a timely manner.

## MATERIALS AND METHODS

### TB-seq primer design

Raw reads for *M. tuberculosis* sequences were downloaded from a previously described globally representative data set (13) (*n* = 489) (Appendix S2) from the European Nucleotide Archive at the European Molecular Biology Laboratory-European Bioinformatics Institute. The reference genome (H37Rv; accession NC_000962.3) was obtained from the National Center for Biotechnology Information (NCBI) GenBank.

Variants were called against the reference using Snippy, and a time-resolved ML tree was built using our variant call file, along with sample data generated from Augur (v.22.4.0) (82), IQ-TREE (v.2.23) (83), and TreeTime (v.0.10.1) (84). Representative sequences (*n* = 6) were selected from across this tree using Parnas (v.0.1.4) (85), to cover >50% of the expected overall diversity. We used these representatives to create an *M. tuberculosis* core genome assembly using Snippy (v.4.6.0) (https://github.com/tseemann/snippy).

Tiled primer schemes (target amplicon size 2 kb) were designed for the full H37Rv reference genome (NCBI accession NC_000962.3) using PrimalScheme (v.1.4.1) (35). PE/PPE regions (conserved proline-glutamine/proline-proline-glutamine domains and hypervariable C-termini (86) and sites with known resistance-related polymorphisms were hard-masked to prevent primers from being designed at these loci. Primer sequences are included in Appendix S3.

### SP-seq primer design

For comparison to TB-seq, an amplicon schema was designed for *S. pneumoniae*, which is highly recombinogenic and frequently reshuffles its genome via horizontal gene transfer. Because of the open nature of its pangenome, we targeted primers only in the core genomic regions shared across several strains (Appendix S2).

We downloaded all available *S. pneumoniae* serotype 3 contigs (*n* = 490) (Appendix S2) from the Global Pneumococcal Sequencing (GPS) database (87) as of 2 February 2023. These sequences were assembled into a reference-guided “metaconsensus sequence” based on the alignment of genomes from multiple strains mapped to the serotype 3 reference. PrimalScheme (35) was run on this metaconsensus sequence as described for TB-seq.

### Primer pooling

Both primer sets were synthesized by Integrated DNA Technologies (Coralville, IA, USA), desalted, and in IDTE pH 8.0, at concentrations of 200 µM and 100 µM for *M. tuberculosis* and *S. pneumoniae*, respectively. Primer pairs in each set were divided into two pools, to separate overlapping tiled primers, and combined in equal proportions to create working primer solutions. These solutions were not diluted any further prior to use in the PCR reaction.

### Clinical specimens

*M. tuberculosis* clinical samples consisted of DNA extracted from positive solid or liquid cultures from sputum and DNA extracted directly from sputum specimens. Extracts from culture consisted of remnant specimens from a study site in Moldova, where sputum specimens were tested at a number of diagnostic centers in Moldova by microscopy, GeneXpert MTB/Rif, and culture; positive cultures were sent to the National TB Reference Laboratory in Chisinau for extraction by the cetyltrimethylammonium bromide method, as described previously (11). Extracts from sputum consisted of specimens collected in Peru after routine diagnostics confirmed the presence of *M. tuberculosis*. In order to test the efficiency of different methods for extracting DNA from sputum, each specimen was split into two and processed with two different protocols. A total of 30 unique sputum specimens were processed with two protocols each, and a total of six different protocols were tested. A full list of all *M. tuberculosis* samples and the extraction methods used can be found in Table S1, and a detailed description of extraction methods can be found in Appendix S1.

### *M. tuberculosis* quantification

Following extraction, DNA was quantified using a *M. tuberculosis*-complex-specific RT-qPCR probe-based assay using a previously designed primer and probe set (88) and a custom-designed gBlock gene fragment for use as a positive control, all synthesized by Integrated DNA Technologies (Newark, NJ, USA). Lyophilized primers and probes were resuspended in nuclease-free water to achieve a working stock concentration of 10 µM. The lyophilized gBlock was resuspended in nuclease-free water following manufacturer’s instructions (89), and serially diluted to generate a standard curve that was assayed alongside the clinical samples. All oligonucleotide sequences can be found in Table S6.

RT-qPCR was performed using the Luna Universal Probe One-Step RT-qPCR Kit (New England Biolabs, Ipswich, MA, USA), following the manufacturer’s recommendations for reaction composition (NEB): 10 µL Luna Universal Probe qPCR Master Mix, 0.8 µL forward primer (10 µM), 0.8 µL reverse primer (10 µM), and 0.4 µL probe; with 3 µL nuclease-free water and 5 µL template DNA, for a total reaction volume of 20 µL. Each RT-qPCR plate included a negative template control and eight gBlock 10-fold serial dilutions with known copy numbers of the target DNA region from 1 × 10^7^ to 1 × 10^0^. All assays were run on a CFX 96 thermal cycler (Bio-Rad Laboratories, Hercules, CA, USA) and interpreted using the CFX Maestro Software for CFX Instruments (Bio-Rad). Genome copy number was calculated by the CFX Maestro Software using the Cq values for the 10-fold serial dilution standards to calculate reaction efficiency and generate a standard curve of Cq vs log-concentration. The standard curve was used to calculate genome copy numbers for experimental samples.

### Amplicon sequencing

DNA libraries were prepared using the Illumina COVIDSeq DNA Prep Kit (Illumina, San Diego, CA, USA) with the included SARS-CoV-2 primers replaced with primer pools for *M. tuberculosis*, as previously described (90). Template DNA was amplified in two separate multiplex PCR reactions, with each primer pool containing non-overlapping sections of the tiled primer scheme. After amplification, PCR products were combined in equal parts and subjected to a 3 minute tagmentation. Tagmented amplicons were purified with a bead cleanup, followed by library amplification with IDT for Illumina Unique Dual Indexes (Illumina, San Diego, CA, USA). Individual indexed libraries were pooled together in equal proportions and underwent a double-ended bead cleanup, selecting for DNA fragments between 300 and 600 bp. The purified library pool was quantified using the 1× dsDNA High-Sensitivity Assay Kit on the Qubit 4 Fluorometer (Thermo Fisher, Waltham, MA, USA), and fragment distribution was verified using the dsDNA High-Sensitivity kit on the 2100 Agilent Bioanalyzer Instrument (Agilent, Santa Clara, CA, USA). Pooled libraries were sequenced in 150 bp paired-end reads at the Yale Center for Genome Analysis on an Illumina NovaSeq (Illumina, San Diego, CA, USA), with an average of 10 million reads per library.

Unamplified libraries were prepared as described above, with the primer pools in the first PCR reaction omitted and replaced with nuclease-free water.

### Alignments and calling

Reads were aligned to the *M. tuberculosis* H37Rv reference using BWA-MEM (v.2.2.1) (91) and SAMtools (v.1.15.1) (92). Amplicon sequencing data were filtered (using defaults; Q > 20 over a sliding window of 4, minimum read length of 50% of the average length). *M. tuberculosis* primer sequences were trimmed using iVar (v.1.4.2) (93). Metagenomic sequences were trimmed and filtered for quality and length (<100 bp) using Trim Galore (v.0.6.10) (94). Variants were called and filtered (Phred score Q > 10 and read depth >10) using BCFtools (v.1.21) (95). Read subsampling, depth, and coverage were calculated using SAMtools (92). Raw reads were directly submitted to the CZID mNGS Illumina pipeline (96) for microbial composition characterization within samples. Further data analyses and visualizations were carried out in RStudio (v.2024.04.2+764) (97) using the tidyverse suite (v.2.0.0) (98).

### Off-target amplification prediction

For both the *M. tuberculosis* and *S. pneumoniae* primer schemes, off-target amplification was assessed *in silico* against a set of related genomes. We first downloaded *S. pneumoniae* complete genome assemblies (*n* = 35) (87) representing 62% of the GPS database lineages. Lineages containing serotype 3 (i.e., GPSC 12) were excluded to demonstrate off-target amplification, though the reference genome for serotype 3 (i.e., OXC 141) was retained (Fig. S3b). We similarly selected a subset of *M. tuberculosis* sequences (*n* = 76) from our initial set used to develop the *M. tuberculosis* primers (Fig. S3a). For each species, we further compiled a genome cluster consisting of the reference genome used for primer design, 12 diverse strains representing various lineages, and an outgroup (Table S5a) using Parnas (v.0.1.4). The pangenome for each cluster was calculated using Roary (v.3.13.0) (99) and an ML phylogeny was constructed using FastTree (v.2.1.11) (100). Average nucleotide identity was calculated between our references and all other genomes in the cluster using FastANI (v.1.34) (101). Off-target amplification was inferred by primer alignment using Bowtie (v.1.3.1) (102); amplicons were predicted for any properly oriented amplicon pairs within 2,200 bp.

Following sequencing of clinical samples, off-target amplicons from our TB panel only were predicted against a range of co-infections and commensal bacteria found within our patient samples; a list of reference genomes used in this analysis is given in Table S5b.

### Serotyping, lineage assignment, and resistance prediction

Mykrobe (42) was used to both assign lineages and predict resistance using the built-in panel 202309 for *M. tuberculosis* (103). Mykrobe generates calls for both fixed resistant and heteroresistant samples (those that are heterozygous at the resistance-associated locus; with an expected sensitivity of >90% at an allele frequency of 8% or above [104]). A time-resolved ML tree was built using our variant call file, along with sample data generated from Augur (v.22.4.0), IQ-TREE (v.2.23), and TreeTime (v.0.10.1) (Fig. S2). Tree visualizations were done using Auspice (v.2.57.0).

## Data Availability

Pipelines for data processing and drug susceptibility prediction, and code for analysis and publication figures, are available on Github (https://github.com/cck42/YSPH_TBseq/releases/tag/v2.0.2). All raw sequences used in this study are available on SRA under BioProject PRJNA1280504 (https://www.ncbi.nlm.nih.gov/bioproject/1280504). While stocks are available, aliquots of the TB-seq primer pool can be sent to academic or public health partners for testing on request.

## SUPPLEMENTAL MATERIAL

Supplemental material jcm.01823-25-s0001.docx Figures S1 to S4, Tables S1 to S6, and Appendices S1 and S3.

Appendix S2 jcm.01823-25-s0002.xlsx TB-seq and *S. pneumoniae* pangenome assembly and primer design reference sequences.

ASM does not own the copyrights to Supplemental Material that may be linked to, or accessed through, an article. The authors have granted ASM a non-exclusive, world-wide license to publish the Supplemental Material files. Please contact the corresponding author directly for reuse.
