# Supplementary material for: A tiled amplicon protocol for culture-free whole-genome sequencing of M. tuberculosis from clinical specimens
Source: J Clin Microbiol. 2026 Feb 9;64(3):e01823-25. doi: 10.1128/jcm.01823-25 (PMC12977623; doi:10.1128/jcm.01823-25)
Supplement: Supplemental material — Figures S1 to S4, Tables S1 to S6, and Appendices S1 and S3. [file jcm.01823-25-s0001.docx]

## TB-seq: A Tiled Amplicon Panel for Culture-free Whole-Genome Sequencing of *M. tuberculosis* from Clinical Specimens+

Chaney C Kalinich^1 *^, Freddy L Gonzalez^2 *^, Alice Osmaston^3,4^, Mallery I Breban^1^, Isabel Distefano^1^, Candy Leon^4^, Patricia Sheen^4^, Grace Tan^3^, Valeriu Crudu^5^, Nelly Ciobanu^5^, Alexandru Codreanu^5^, Walter Solano^4^, Jimena Ráez^4^, Mirko Zimic^4^, Jorge Coronel^4^, Orchid M Allicock^1,6^, Chrispin Chaguza^1^, Anne L Wyllie^1^, Matthew Brandt^1^, Daniel M Weinberger^1,6,7^, Benjamin Sobkowiak^3,7^,Ted Cohen^1,7^, Louis Grandjean^3,4^, Nathan D Grubaugh^1,2,6,7^, Seth N Redmond^1,6^

1 Department of Epidemiology of Microbial Diseases, Yale School of Public Health, New Haven, Connecticut, USA

2 Department of Ecology and Evolutionary Biology, Yale University, New Haven, Connecticut, USA

3 Department of Infection, Immunity, and Inflammation, Institute of Child Health, University College Longon, London, England

4 Universidad Peruana Cayetano Heredia, Lima, Peru

5 Institute of Phthisiopneumology, Chisinau, Moldova

6 Yale Institute for Global Health, Yale University, New Haven, Connecticut, USA

7 Public Health Modeling Unit, Yale School of Public Health, New Haven, Connecticut, USA

* these authors contributed equally to this work

Contents

[Figure S1. Efficiency of extraction protocol affects antimicrobial resistance predictions. 2](#_1zko7qhpihen)

[Figure S2. Amplicon sequencing can be used in phylogenetic investigation. 3](#_ulqte4fzftxb)

[Figure S3A. Mycobacterium tuberculosis pangenome. 6](#_nvjuf1opgg1p)

[Figure S3B. Streptococcus pneumoniae pangenome. 6](#_o81ljhpjxfjh)

[Fig S4a: In silico off-target amplification prediction 6](#_um49de23n0s)

[Fig S4b: Peru 47 sample alignment to S. Odontolytica 7](#_f76umtp4sexk)

[Table S1. M. tuberculosis clinical specimens. 8](#_pnrhbtih5c1f)

[Table S2 Dilution curve sample coverage; unamplified vs TB-seq samples 11](#_t4hy7zrglow)

[Table S3: TB-seq library preparation costs 12](#_nbuctlq1cpur)

[Table S4a Resistance predictions and phenotypes 13](#_s0p4c8x10na3)

[Table S4b Mean sequencing depths in resistance-associated genes; colony samples 15](#_v0pjwfn35eyd)

[Table S4c Mean sequencing depths in resistance-associated genes; sputum samples 16](#_abdnkq7k94w)

[Table S5a. Samples used to predict clade amplification. 17](#_ig39md728169)

[Table S5b. References used for commensal / coinfection comparisons 18](#_9mulxfzf6jx7)

[Table S6 M. tuberculosis RT-qPCR Oligonucleotide Sequences 18](#_m3rrwj61bzoi)

[Appendix S1. M. tuberculosis extraction methods from sputum 19](#_wjb01ohhoseo)

[Appendix S3. TB-seq primer sequences and genomic positions 21](#_khsuzlflnnht)

#


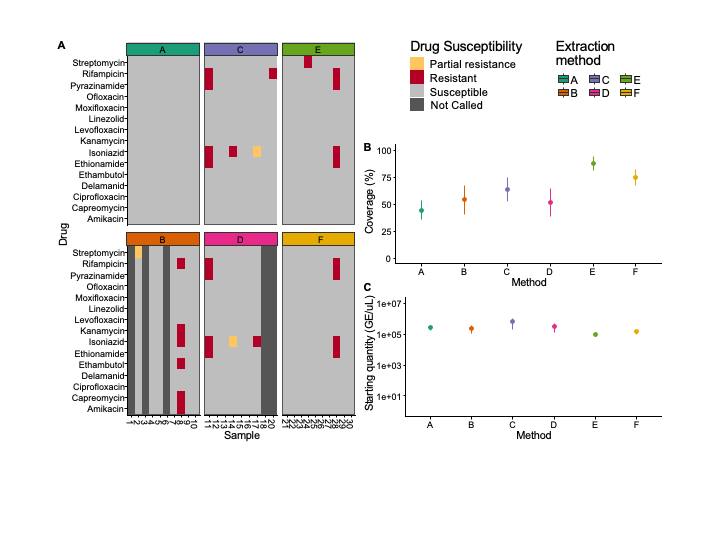


# Figure S1. Efficiency of extraction protocol affects antimicrobial resistance predictions.

Six different extraction protocols were assessed; 30 total unique specimens were extracted using 2 different protocols each. Mapping between patient and sample IDs is available in Table S1B. (A) Susceptibility to 14 anti-TB drugs by amplicon sequencing was predicted for all DNA extracted directly from sputum. (B) Comparison of mean coverage for each extraction protocol (dot) and standard error (line). (C) Comparison of mean starting quantity for each extraction protocol (dot) and standard error (line). Method E produced the highest average genome coverage and starting quantity with the least variation and will be used going forward.

#
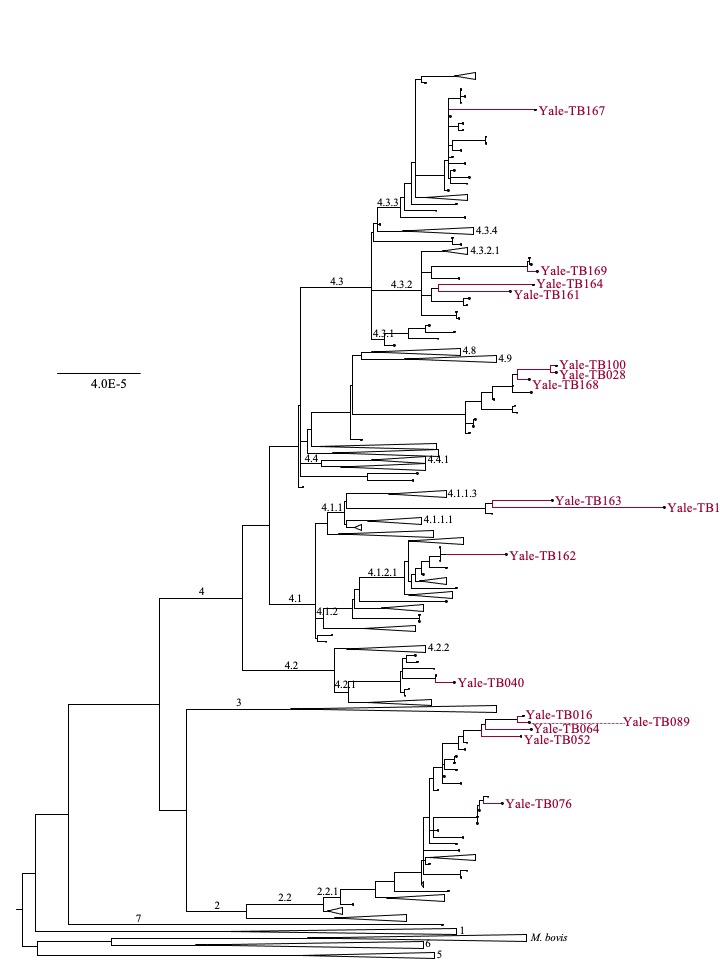


# Figure S2. Amplicon sequencing can be used in phylogenetic investigation.

Maximum likelihood tree including all specimens with >80% coverage across the whole genome (highlighted in red), with sequences used for primer design (File S1A) as context. Branches are labeled according to lineage/sublineage according to a previously-described SNP barcoding system [(Coll et al. 2014)](https://paperpile.com/c/e1N22e/4VxF). For all samples, the primary lineage and sublineage called by Mykrobe is in accordance with the sublineage in the tree. A complete and interactive tree can be found at: [https://nextstrain.org/community/cck42/tbtrees@master/20241022](https://nextstrain.org/community/cck42/tbtrees@master/20241022?c=mykrobe_lineage)


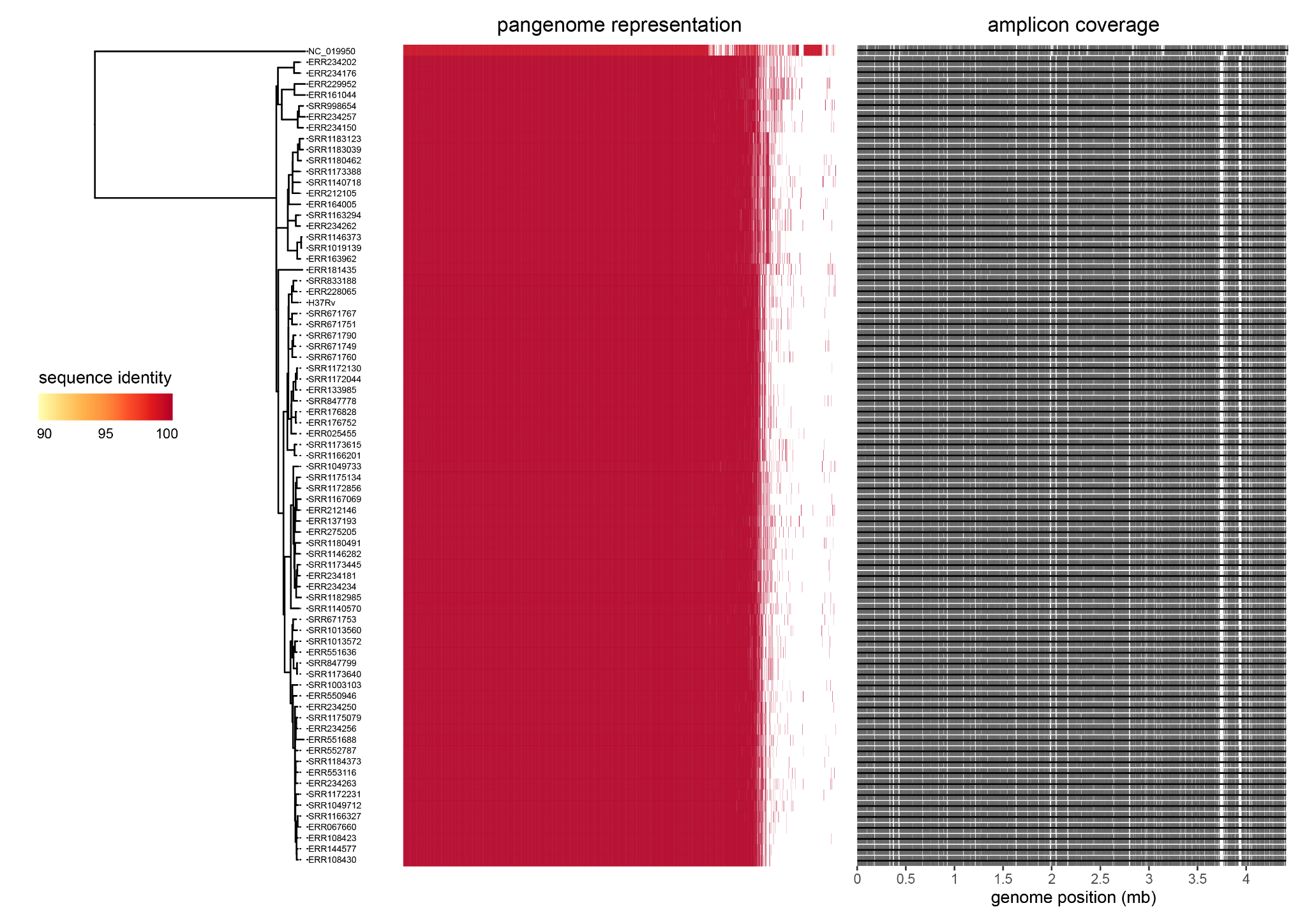


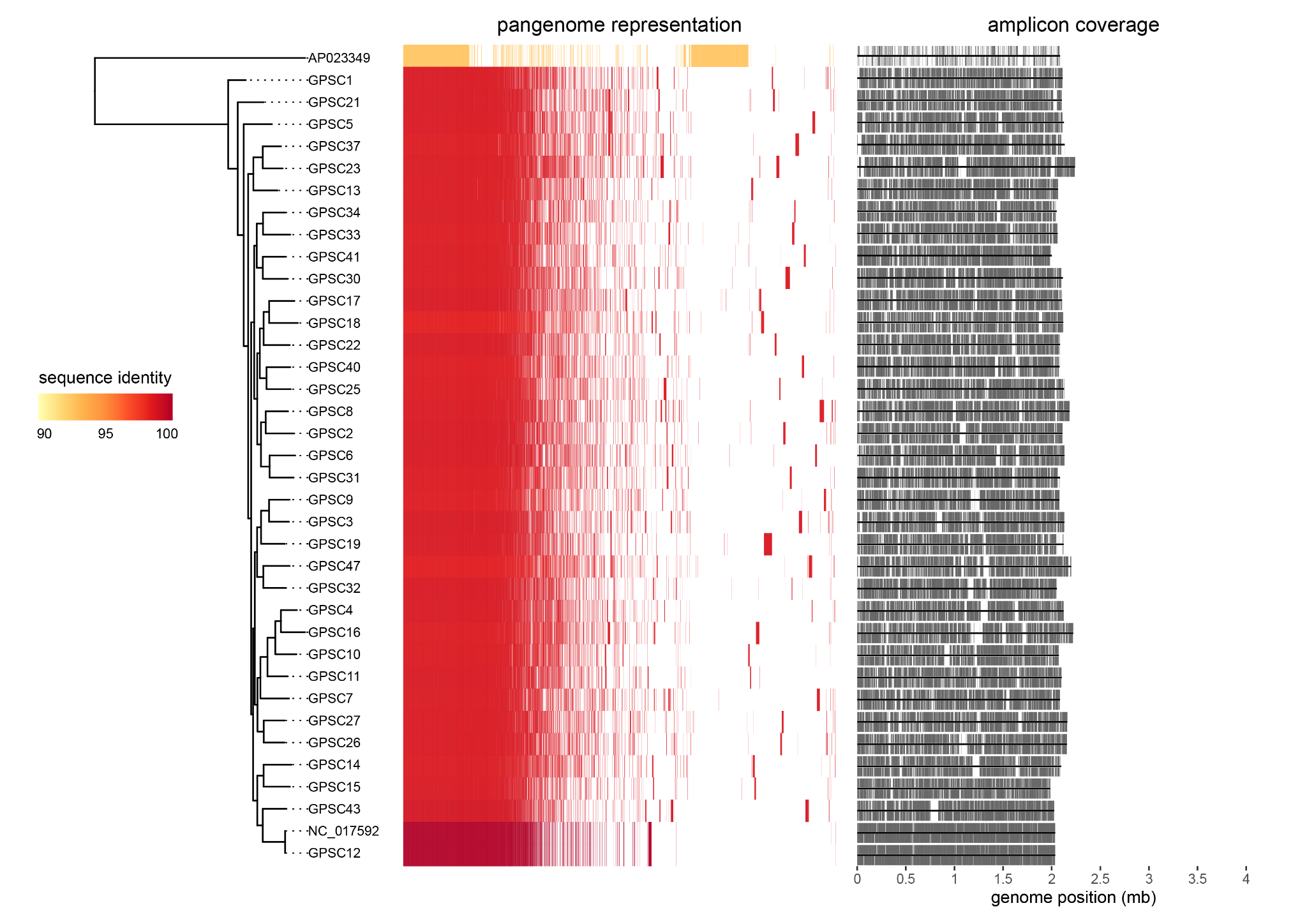


# Figure S3A. *Mycobacterium tuberculosis* pangenome.

Pangenome representation of *M. tuberculosis* whole genome sequences (n=76), *Mycobacterium canettii* outgroup (Accession: NC_019950), and reference *M. tuberculosis* sequence used to build primers (Accession: H37Rv). Shaded bar graphs (middle) denote genes shared amongst clades, color denotes average nucleotide identity. Predicted amplicon coverage (right) is shown in grey with forward and reverse amplicon pairs displayed above and below the line.

#

# Figure S3B. *Streptococcus pneumoniae* pangenome.

Pangenome representation of *S. pneumoniae* whole genome Global Pneumococcal Sequence Clusters (GPSC) sequences (n=35), *Streptococcus mitis* outgroup (Accession: AP023349), and reference *S. pneumoniae* used to build primers (Accession: NC_017592). Shaded bar graphs (middle) denote genes shared amongst clades, color denotes average nucleotide identity. Predicted amplicon coverage (right) is shown in grey with forward and reverse amplicon pairs displayed above and below the line.

# Fig S4a: In silico off-target amplification prediction

**
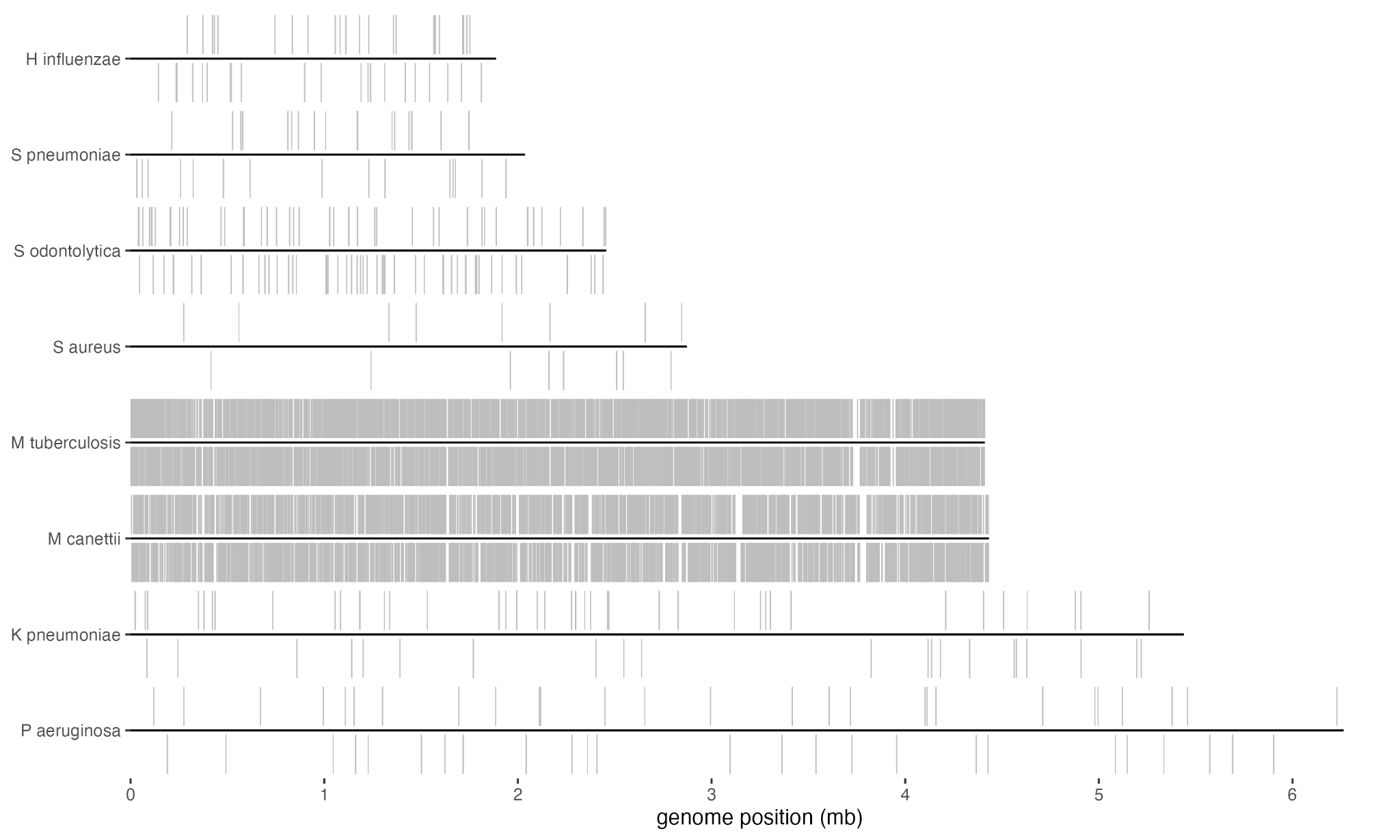
**

Off-target amplicons were predicted to a range of bacterial references representing coinfections and commensal bacteria encountered in our sputum samples; while high coverage was predicted for all species within the Mycobacterium clade, only *S. odontolytica* showed significant predicted amplification, with 10% of the genome covered by potential amplicons.

# Fig S4b: Peru 47 sample alignment to *S. Odontolytica*

**
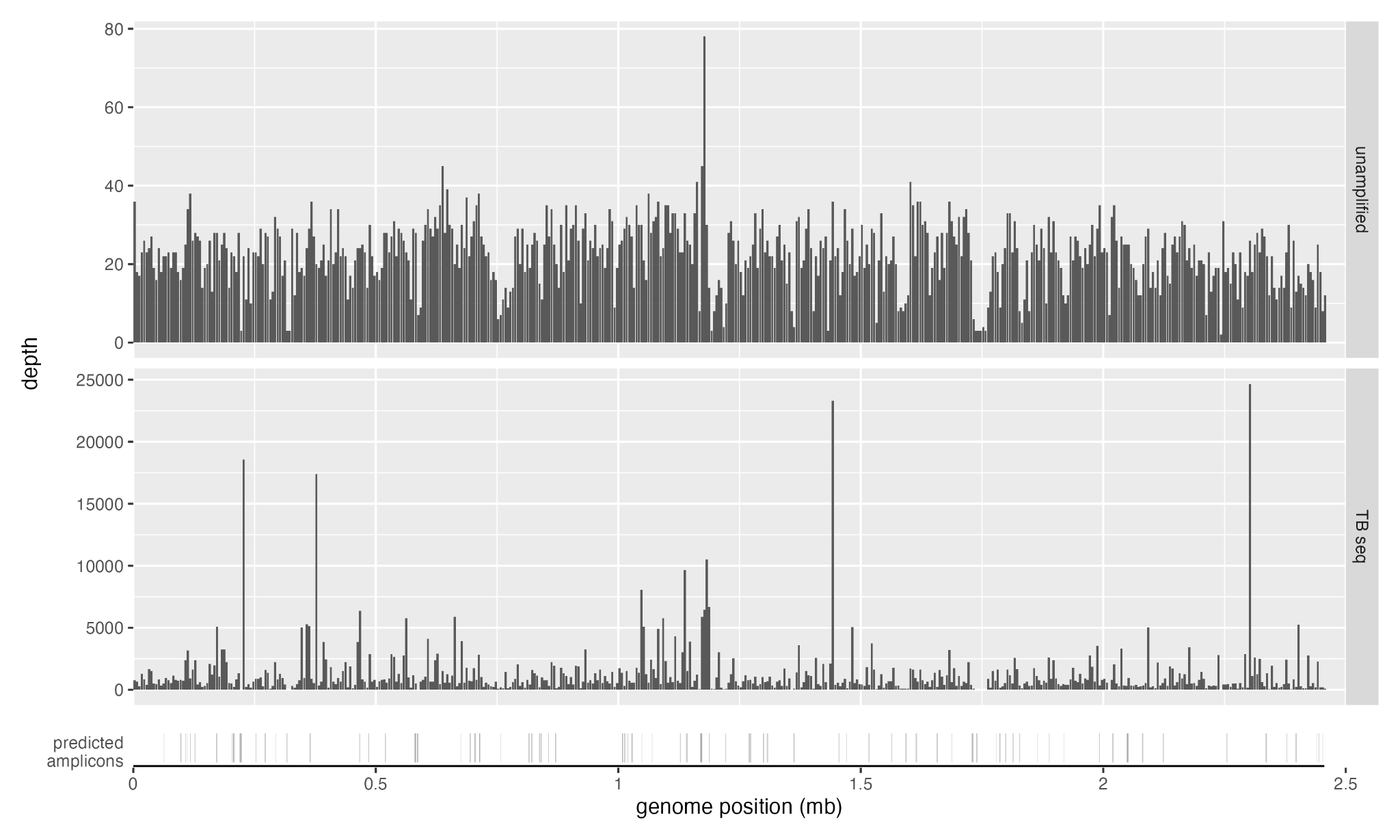
**

Sequences generated from the Peru 47 sample were aligned, unamplified and after TB-seq enrichment, to the *S. odontolytica* genome NZ_CP133472.1. Consistent with metagenomics results, while unamplified samples showed low but consistent coverage across the *S. odontolytica* genome, TB-seq enrichment led to extreme amplification in some areas, leading to high levels of read recovery, but without generating consistent high depth across the genome.

# Table S1. *M. tuberculosis* clinical specimens.

| **Sample** | **Patient No.** | **Type** | **Origin*** | **Smear grade** | **Protocol** | **Heat** | **Liquefaction** | **Decontamination** | **Homogenization** |
| --- | --- | --- | --- | --- | --- | --- | --- | --- | --- |
| 111-10300-18 | - | Cultured sputum | Moldova | - | - | - | NALC 0.5% | NaOH | - |
| 444-3403-18 | - | Cultured sputum | Moldova | - | - | - | NALC 0.5% | NaOH | - |
| 111-5264-18 | - | Cultured sputum | Moldova | - | - | - | NALC 0.5% | NaOH | - |
| 111-10712-18 | - | Cultured sputum | Moldova | - | - | - | NALC 0.5% | NaOH | - |
| 111-2565-18 | - | Cultured sputum | Moldova | - | - | - | NALC 0.5% | NaOH | - |
| 444-2261-18 | - | Cultured sputum | Moldova | - | - | - | NALC 0.5% | NaOH | - |
| 444-2281-18 | - | Cultured sputum | Moldova | - | - | - | NALC 0.5% | NaOH | - |
| 444-2588-18 | - | Cultured sputum | Moldova | - | - | - | NALC 0.5% | NaOH | - |
| Peru-1 | 1 | Sputum | Peru | 3 BAAR | A | 95℃ x 20 min | Saponin | None | 5.5m/s x 40s x 2 cycles |
| Peru-2 | 2 | Sputum | Peru | 1+ | A | 95℃ x 20 min | Saponin | None | 5.5m/s x 40s x 2 cycles |
| Peru-3 | 3 | Sputum | Peru | 1+ | A | 95℃ x 20 min | Saponin | None | 5.5m/s x 40s x 2 cycles |
| Peru-4 | 4 | Sputum | Peru | 2+ | A | 95℃ x 20 min | Saponin | None | 5.5m/s x 40s x 2 cycles |
| Peru-5 | 5 | Sputum | Peru | 2+ | A | 95℃ x 20 min | Saponin | None | 5.5m/s x 40s x 2 cycles |
| Peru-6 | 6 | Sputum | Peru | 2+ | A | 95℃ x 20 min | Saponin | None | 5.5m/s x 40s x 2 cycles |
| Peru-7 | 7 | Sputum | Peru | 3+ | A | 95℃ x 20 min | Saponin | None | 5.5m/s x 40s x 2 cycles |
| Peru-8 | 8 | Sputum | Peru | 1+ | A | 95℃ x 20 min | Saponin | None | 5.5m/s x 40s x 2 cycles |
| Peru-9 | 9 | Sputum | Peru | 1+ | A | 95℃ x 20 min | Saponin | None | 5.5m/s x 40s x 2 cycles |
| Peru-10 | 10 | Sputum | Peru | 3+ | A | 95℃ x 20 min | Saponin | None | 5.5m/s x 40s x 2 cycles |
| Peru-11 | 1 | Sputum | Peru | 3 BAAR | B | 95℃ x 20 min | NALC 0.5% | None | 5.5m/s x 40s x 2 cycles |
| Peru-12 | 2 | Sputum | Peru | 1+ | B | 95℃ x 20 min | NALC 0.5% | None | 5.5m/s x 40s x 2 cycles |
| Peru-13 | 3 | Sputum | Peru | 1+ | B | 95℃ x 20 min | NALC 0.5% | None | 5.5m/s x 40s x 2 cycles |
| Peru-14 | 4 | Sputum | Peru | 2+ | B | 95℃ x 20 min | NALC 0.5% | None | 5.5m/s x 40s x 2 cycles |
| Peru-15 | 5 | Sputum | Peru | 2+ | B | 95℃ x 20 min | NALC 0.5% | None | 5.5m/s x 40s x 2 cycles |
| Peru-16 | 6 | Sputum | Peru | 2+ | B | 95℃ x 20 min | NALC 0.5% | None | 5.5m/s x 40s x 2 cycles |
| Peru-17 | 7 | Sputum | Peru | 3+ | B | 95℃ x 20 min | NALC 0.5% | None | 5.5m/s x 40s x 2 cycles |
| Peru-18 | 8 | Sputum | Peru | 1+ | B | 95℃ x 20 min | NALC 0.5% | None | 5.5m/s x 40s x 2 cycles |
| Peru-19 | 9 | Sputum | Peru | 1+ | B | 95℃ x 20 min | NALC 0.5% | None | 5.5m/s x 40s x 2 cycles |
| Peru-20 | 10 | Sputum | Peru | 3+ | B | 95℃ x 20 min | NALC 0.5% | None | 5.5m/s x 40s x 2 cycles |
| Peru-21 | 11 | Sputum | Peru | 2+ | C | None | NALC 1% | None | 5.5m/s x 40s x 2 cycles |
| Peru-22 | 12 | Sputum | Peru | 2+ | C | None | NALC 1% | None | 5.5m/s x 40s x 2 cycles |
| Peru-23 | 13 | Sputum | Peru | 3+ | C | None | NALC 1% | None | 5.5m/s x 40s x 2 cycles |
| Peru-24 | 14 | Sputum | Peru | 1+ | C | None | NALC 1% | None | 5.5m/s x 40s x 2 cycles |
| Peru-25 | 15 | Sputum | Peru | 3+ | C | None | NALC 1% | None | 5.5m/s x 40s x 2 cycles |
| Peru-26 | 16 | Sputum | Peru | 3+ | C | None | NALC 1% | None | 5.5m/s x 40s x 2 cycles |
| Peru-27 | 17 | Sputum | Peru | 2+ | C | None | NALC 1% | None | 5.5m/s x 40s x 2 cycles |
| Peru-28 | 18 | Sputum | Peru | 2+ | C | None | NALC 1% | None | 5.5m/s x 40s x 2 cycles |
| Peru-29 | 19 | Sputum | Peru | 1+ | C | None | NALC 1% | None | 5.5m/s x 40s x 2 cycles |
| Peru-30 | 20 | Sputum | Peru | 3 baar | C | None | NALC 1% | None | 5.5m/s x 40s x 2 cycles |
| Peru-31 | 11 | Sputum | Peru | 2+ | D | None | NALC 2% | None | 5.5m/s x 40s x 2 cycles |
| Peru-32 | 12 | Sputum | Peru | 2+ | D | None | NALC 2% | None | 5.5m/s x 40s x 2 cycles |
| Peru-33 | 13 | Sputum | Peru | 3+ | D | None | NALC 2% | None | 5.5m/s x 40s x 2 cycles |
| Peru-34 | 14 | Sputum | Peru | 1+ | D | None | NALC 2% | None | 5.5m/s x 40s x 2 cycles |
| Peru-35 | 15 | Sputum | Peru | 3+ | D | None | NALC 2% | None | 5.5m/s x 40s x 2 cycles |
| Peru-36 | 16 | Sputum | Peru | 3+ | D | None | NALC 2% | None | 5.5m/s x 40s x 2 cycles |
| Peru-37 | 17 | Sputum | Peru | 2+ | D | None | NALC 2% | None | 5.5m/s x 40s x 2 cycles |
| Peru-38 | 18 | Sputum | Peru | 2+ | D | None | NALC 2% | None | 5.5m/s x 40s x 2 cycles |
| Peru-39 | 19 | Sputum | Peru | 1+ | D | None | NALC 2% | None | 5.5m/s x 40s x 2 cycles |
| Peru-40 | 20 | Sputum | Peru | 3 baar | D | None | NALC 2% | None | 5.5m/s x 40s x 2 cycles |
| Peru-41 | 21 | Sputum | Peru | 1+ | E | None | NALC 0.5% | NaOH | 5.5m/s x 40s x 2 cycles |
| Peru-42 | 22 | Sputum | Peru | 2+ | E | None | NALC 0.5% | NaOH | 5.5m/s x 40s x 2 cycles |
| Peru-43 | 23 | Sputum | Peru | 1+ | E | None | NALC 0.5% | NaOH | 5.5m/s x 40s x 2 cycles |
| Peru-44 | 24 | Sputum | Peru | 1+ | E | None | NALC 0.5% | NaOH | 5.5m/s x 40s x 2 cycles |
| Peru-45 | 25 | Sputum | Peru | 1+ | E | None | NALC 0.5% | NaOH | 5.5m/s x 40s x 2 cycles |
| Peru-46 | 26 | Sputum | Peru | 1+ | E | None | NALC 0.5% | NaOH | 5.5m/s x 40s x 2 cycles |
| Peru-47 | 27 | Sputum | Peru | 8 baar | E | None | NALC 0.5% | NaOH | 5.5m/s x 40s x 2 cycles |
| Peru-48 | 28 | Sputum | Peru | 3+ | E | None | NALC 0.5% | NaOH | 5.5m/s x 40s x 2 cycles |
| Peru-49 | 29 | Sputum | Peru | 1+ | E | None | NALC 0.5% | NaOH | 5.5m/s x 40s x 2 cycles |
| Peru-50 | 30 | Sputum | Peru | 2+ | E | None | NALC 0.5% | NaOH | 5.5m/s x 40s x 2 cycles |
| Peru-51 | 21 | Sputum | Peru | 1+ | F | None | NALC 0.5% | NaOH | 5.5m/s x 20s x 2 cycles |
| Peru-52 | 22 | Sputum | Peru | 2+ | F | None | NALC 0.5% | NaOH | 5.5m/s x 20s x 2 cycles |
| Peru-53 | 23 | Sputum | Peru | 1+ | F | None | NALC 0.5% | NaOH | 5.5m/s x 20s x 2 cycles |
| Peru-54 | 24 | Sputum | Peru | 1+ | F | None | NALC 0.5% | NaOH | 5.5m/s x 20s x 2 cycles |
| Peru-55 | 25 | Sputum | Peru | 1+ | F | None | NALC 0.5% | NaOH | 5.5m/s x 20s x 2 cycles |
| Peru-56 | 26 | Sputum | Peru | 1+ | F | None | NALC 0.5% | NaOH | 5.5m/s x 20s x 2 cycles |
| Peru-57 | 27 | Sputum | Peru | 8 baar | F | None | NALC 0.5% | NaOH | 5.5m/s x 20s x 2 cycles |
| Peru-58 | 28 | Sputum | Peru | 3+ | F | None | NALC 0.5% | NaOH | 5.5m/s x 20s x 2 cycles |
| Peru-59 | 29 | Sputum | Peru | 1+ | F | None | NALC 0.5% | NaOH | 5.5m/s x 20s x 2 cycles |
| Peru-60 | 30 | Sputum | Peru | 2+ | F | None | NALC 0.5% | NaOH | 5.5m/s x 20s x 2 cycles |

*All samples from Moldova were remnant samples of DNA extracted from culture for a previous study [(Yang et al. 2022)](https://paperpile.com/c/D8faXY/VNPR). Peru specimens were extracted at Cayetano University, Peru.

**Cetyltrimethylammonium bromide (CTAB) method was used as described previously [(Schiebelhut et al. 2017)](https://paperpile.com/c/D8faXY/8E19).

# Table S2 Dilution curve sample coverage; unamplified vs TB-seq samples

#

|  | **Specimen** | **Genome coverage (%)** | | | | | | | |
| --- | --- | --- | --- | --- | --- | --- | --- | --- | --- |
|  |  | **1E-08** | **1E-07** | **1E-06** | **1E-05** | **1E-04** | **1E-03** | **1E-02** | **1E-01** |
| **TB-seq** | **111-5264-18** | 43.19 | 68.84 | 94.91 | 97.21 | 97.69 | 97.70 | 97.65 | 97.73 |
|  | **111-10712-18** | 12.21 | 66.38 | 94.15 | 96.32 | 97.91 | 98.09 | 98.10 | 98.06 |
| **Unamplified** | **111-5264-18** | 0.01 | 0.10 | 3.73 | 36.00 | 97.11 | 97.71 | 97.77 | 97.78 |
|  | **111-10712-18** | 0.09 | 0.56 | 10.47 | 77.46 | 96.88 | 98.10 | 98.11 | 98.11 |

*M. tuberculosis* genome coverage for serial dilutions of DNA from concentrated (cultured) isolates. Samples were prepared for sequencing with and without TB-seq primers added in order to determine whether tiled amplification improved genome recovery. Results indicate that tiled amplicon sequencing enables high (>90%) genome recovery from 100-fold lower starting concentrations than preparation without amplification.

# Table S3: TB-seq library preparation costs

| **Component** | **Vendor** | **Catalogue Number** | **Price** | **units/ea** | **units/rxn** | **Price/rxn** |
| --- | --- | --- | --- | --- | --- | --- |
| **Reagents** |  |  |  |  |  |  |
| COVIDSeq™ Test 3072 Samples | Illumina | 20043675 | $40,006 | 3072 | 1 | $ 13.02 |
| IDT® for Illumina PCR Unique Dual Indexes Set 1-4 (384 Indexes) | Illumina | 20043137 | $1,544 | 384 | 1 | $ 4.02 |
| **TB-seq Primers** |  |  |  |  |  |  |
| TB-seq primers (total pool) | IDT | n/a | $9,994 | 256,400 | 7.2 | $ 0.28 |
| **Major Consumables - Manual Pipetting** |  |  |  |  |  |  |
| P10 XL Filtered Pipette Tips | USA Scientific | 1120-3710 | $68 | 960 | 10 | $ 0.70 |
| P300 Filtered Pipette Tips (Refill) | USA Scientific | 1180-9710 | $79 | 960 | 6 | $ 0.49 |
| 96-well twin/tec PCR Plate, 250 uL PCR clean, colorless | Eppendorf | 30129504 | $147 | 25 | 4 | $ 0.24 |
| Eppendorf Heat Sealing Foil, PCR clean, 100 pcs. | Eppendorf | 30127854 | $155 | 100 | 7 | $ 0.11 |
| Eppendorf PCR Foil, self-adhesive, PCR clean, 100 pcs. | Eppendorf | 30127790 | $208 | 100 | 7 | $ 0.15 |
| Qubit Flex Assay Tube Strips | Thermo Fisher | Q33252 | $528 | 125 | 1 | $ 0.04 |
|  |  |  |  |  |  |  |
| **Total for Manual Protocol** |  |  |  |  |  | **$ 19.07** |
|  |  |  |  |  |  |  |
| **Sequencing** (10m 2x150bp reads) | Yale Center for Genome Analysis |  |  |  |  | **$ 10.00** |

IDT: Integrated DNA Technologies; P10/P300: 10µL/300µL pipette; µL: Microlitre; ea: each; rxn: reaction; pcs: pieces; bp: base pairs

#

# Table S4a Resistance predictions and phenotypes

#

|  | 111-10300-18 | | | | | 111-10712-18 | | | | | 111-2565-18 | | | | | 111-5264-18 | | | | |
| --- | --- | --- | --- | --- | --- | --- | --- | --- | --- | --- | --- | --- | --- | --- | --- | --- | --- | --- | --- | --- |
|  | phenotyping | | | prediction | | phenotyping | | | prediction | | phenotyping | | | prediction | | phenotyping | | | prediction | |
| drug | liquid | solid | xpert | seq | TB-seq | liquid | solid | xpert | seq | TB-seq | liquid | solid | xpert | seq | TB-seq | liquid | solid | xpert | seq | TB-seq |
| ethambutol | S | - | - | - | S | S | - | - | S | S | - | - | - | - | S | - | - | - | R | R |
| pyrazinamide | S | - | - | - | S | S | - | - | S | S | - | - | - | - | S | - | - | - | R | R |
| rifampicin | S | - | - | - | S | S | - | S | S | S | - | - | R | - | R | - | - | R | R | R |
| isoniazid | R | - | - | - | R | S | - | - | S | S | - | - | - | - | R | - | - | - | R | R |
| capreomycin | S | - | - | - | S | - | - | - | S | S | S | S | - | - | S | - | - | - | S | S |
| kanamycin | S | - | - | - | S | - | - | - | S | S | - | - | - | - | R | - | - | - | S | S |
| amikacin | S | - | - | - | S | - | - | - | S | S | S | S | - | - | S | - | - | - | S | S |
| levofloxacin | S | - | - | - | S | - | - | - | S | S | R | R | - | - | R | - | - | - | R | R |
| moxifloxacin | S | - | - | - | S | - | - | - | S | S | S | - | - | - | R | - | - | - | R | R |
| streptomycin | - | - | - | - | R | - | - | - | S | S | - | - | - | - | S | - | - | - | R | R |
| linezolid | - | - | - | - | S | - | - | - | S | S | S | - | - | - | S | - | - | - | S | S |
| ethionamide | S | - | - | - | S | - | - | - | S | S | R | R | - | - | R | - | - | - | R | R |
| delamanid | - | - | - | - | S | - | - | - | S | S | - | - | - | - | S | - | - | - | S | S |

**S4a Resistance predictions and phenotypes (cntd)**

|  | 444-2261-18 | | | | | 444-2281-18 | | | | | 444-2588-18 | | | | | 444-3403-18 | | | | |
| --- | --- | --- | --- | --- | --- | --- | --- | --- | --- | --- | --- | --- | --- | --- | --- | --- | --- | --- | --- | --- |
|  | phenotyping | | | prediction | | phenotyping | | | prediction | | phenotyping | | | prediction | | phenotyping | | | prediction | |
|  | liquid | solid | xpert* | seq | TB-seq | liquid | solid | xpert* | seq | TB-seq | liquid | solid | xpert* | seq | TB-seq | liquid | solid | xpert* | seq | TB-seq |
| ethambutol | - | S | - | - | S | - | R | - | - | R | - | R | - | - | S | S | - | - | - | S |
| pyrazinamide | - | - | - | - | S | - | - | - | - | r | - | - | - | - | R | R | - | - | - | r |
| rifampicin | - | S | - | - | S | - | R | R | - | R | - | R | - | - | R | R | - | - | - | r |
| isoniazid | - | S | - | - | S | - | R | - | - | R | - | R | - | - | R | R | - | - | - | r |
| capreomycin | - | - | - | - | S | - | S | - | - | S | - | S | - | - | S | S | - | - | - | S |
| kanamycin | - | - | - | - | S | - | - | - | - | R | - | - | - | - | S | - | - | - | - | r |
| amikacin | - | - | - | - | S | - | S | - | - | S | - | S | - | - | S | S | - | - | - | S |
| levofloxacin | - | - | - | - | S | - | - | - | - | S | - | - | - | - | r | S | - | - | - | S |
| moxifloxacin | - | - | - | - | S | - | - | - | - | S | - | - | - | - | r | S | - | - | - | S |
| streptomycin | - | S | - | - | S | - | R | - | - | R | - | R | - | - | S | R | - | - | - | S |
| linezolid | - | - | - | - | S | - | - | - | - | S | - | - | - | - | S | - | - | - | - | S |
| ethionamide | - | - | - | - | S | - | - | - | - | S | - | - | - | - | R | - | - | - | - | r |
| delamanid | - | - | - | - | S | - | - | - | - | S | - | - | - | - | S | - | - | - | - | S |

S = susceptible; R = resistant; r = heteroresistant

* Cepheid Xpert MTB/RIF

# Table S4b Mean sequencing depths in resistance-associated genes; colony samples

| **Resistance** | **Gene** | **111-10300-18** | **444-3403-18** | **111-5264-18** | **111-10712-18** | **111-2565-18** | **444-2261-18** | **444-2281-18** | **444-2588-18** |
| --- | --- | --- | --- | --- | --- | --- | --- | --- | --- |
| Isoniazid | ahpC | 153 | 114 | 49 | 249 | 81 | 213 | 316 | 163 |
| Isoniazid | fabG1 | 893 | 929 | 170 | 971 | 368 | 967 | 1350 | 1099 |
| Isoniazid | inhA | 183 | 134 | 59 | 291 | 92 | 244 | 357 | 194 |
| Isoniazid | katG | 219 | 204 | 70 | 276 | 120 | 281 | 400 | 222 |
| Isoniazid | ndh | 548 | 471 | 137 | 643 | 244 | 815 | 979 | 477 |
| Rifampicin | rpoB | 482 | 627 | 197 | 790 | 394 | 1241 | 1405 | 817 |
| Pyrazinamide | pncA | 2632 | 2205 | 593 | 2170 | 972 | 2570 | 2706 | 1338 |
| Pyrazinamide | rpsA | 201 | 126 | 74 | 249 | 110 | 367 | 329 | 177 |
| Ethambutol | embA | 255 | 267 | 113 | 412 | 175 | 480 | 561 | 282 |
| Ethambutol | embB | 562 | 424 | 185 | 680 | 203 | 446 | 597 | 316 |
| Quinolones | gyrA | 6701 | 4010 | 1065 | 3383 | 931 | 2744 | 3695 | 2053 |
| Levofloxacin,Moxifloxacin | gyrB | 2308 | 1411 | 239 | 876 | 237 | 861 | 1196 | 592 |
| Kanamycin | eis | 543 | 606 | 105 | 372 | 202 | 271 | 295 | 167 |
| Capreomycin | gid | 143 | 100 | 51 | 166 | 69 | 168 | 191 | 110 |
| Capreomycin | tlyA | 682 | 621 | 247 | 883 | 405 | 1035 | 1121 | 600 |
| Delamanid | ddn | 720 | 667 | 247 | 602 | 295 | 647 | 675 | 346 |
| Ethionamide | ethA | 1306 | 1507 | 176 | 717 | 217 | 708 | 1272 | 636 |
| Linezolid | rplC | 236 | 250 | 83 | 287 | 165 | 402 | 382 | 205 |
| Streptomycin | rpsL | 39 | 18 | 27 | 83 | 32 | 68 | 113 | 51 |

#

# Table S4c Mean sequencing depths in resistance-associated genes; sputum samples

| **Resistance** | **Gene** | **Peru-41** | **Peru-42** | **Peru-43** | **Peru-44** | **Peru-45** | **Peru-46** | **Peru-47** | **Peru-48** | **Peru-49** | **Peru-50** |
| --- | --- | --- | --- | --- | --- | --- | --- | --- | --- | --- | --- |
| Isoniazid | ahpC | 13 | 242 | 97 | 196 | 148 | 0 | 4 | 151 | 136 | 90 |
| Isoniazid | fabG1 | 23 | 445 | 217 | 459 | 325 | 2 | 8 | 311 | 300 | 213 |
| Isoniazid | inhA | 12 | 312 | 158 | 329 | 219 | 1 | 3 | 238 | 208 | 140 |
| Isoniazid | katG | 9 | 280 | 110 | 258 | 206 | 0 | 4 | 186 | 162 | 124 |
| Isoniazid | ndh | 65 | 645 | 256 | 498 | 352 | 0 | 5 | 495 | 352 | 235 |
| Rifampicin | rpoB | 27 | 465 | 204 | 480 | 384 | 2 | 175 | 470 | 348 | 201 |
| Pyrazinamide | pncA | 171 | 1609 | 718 | 1175 | 664 | 15 | 12 | 1526 | 952 | 594 |
| Pyrazinamide | rpsA | 8 | 454 | 235 | 12360 | 4823 | 17 | 23122 | 1414 | 1933 | 1153 |
| Ethambutol | embA | 9 | 335 | 117 | 315 | 233 | 1 | 6 | 232 | 231 | 122 |
| Ethambutol | embB | 50 | 500 | 170 | 471 | 310 | 7 | 5 | 352 | 410 | 177 |
| Quinolones | gyrA | 3650 | 4399 | 2665 | 4741 | 1959 | 1091 | 69 | 5471 | 4312 | 1730 |
| Levofloxacin,Moxifloxacin | gyrB | 1724 | 2237 | 835 | 1974 | 847 | 34 | 6 | 2329 | 1922 | 573 |
| Kanamycin | eis | 38 | 508 | 260 | 457 | 280 | 2 | 5 | 406 | 319 | 198 |
| Capreomycin | gid | 21 | 268 | 113 | 196 | 144 | 0 | 4 | 179 | 213 | 116 |
| Capreomycin | tlyA | 32 | 378 | 147 | 367 | 234 | 8 | 4 | 335 | 244 | 152 |
| Delamanid | ddn | 17 | 689 | 199 | 556 | 366 | 1 | 6 | 532 | 482 | 207 |
| Ethionamide | ethA | 179 | 848 | 314 | 645 | 370 | 9 | 6 | 644 | 568 | 221 |
| Linezolid | rplC | 4 | 416 | 166 | 348 | 215 | 0 | 4 | 353 | 255 | 156 |
| Streptomycin | rpsL | 13 | 32 | 15 | 44 | 37 | 0 | 53 | 11 | 31 | 18 |

#

# Table S5a. Samples used to predict clade amplification.

| **Pathogen** | **Sample ID** | **Serotype List** | **Predicted Coverage** |
| --- | --- | --- | --- |
| *S. mitis* | AP023349 |  | 32.18% |
| *S. pneumoniae* | NC_017592 |  | 98.93% |
| *S. pneumoniae* | GPSC3 | 8,33F,11A,22F,18C,3,15A,33A,23F,31 | 81.44% |
| *S. pneumoniae* | GPSC4 | 19A,15BC,14,19B,19F | 81.60% |
| *S. pneumoniae* | GPSC8 | 5 | 82.74% |
| *S. pneumoniae* | GPSC15 | 7F,19F | 88.50% |
| *S. pneumoniae* | GPSC21 | 19F,19A,14 | 82.07% |
| *S. pneumoniae* | GPSC22 | 11A,15A,20A,9V,35A,19A,19F,6A | 84.29% |
| *S. pneumoniae* | GPSC26 | 12F,46,12A,40,9V | 81.38% |
| *S. pneumoniae* | GPSC31 | 1 | 82.32% |
| *S. pneumoniae* | GPSC32 | 12F,7F,8,9N | 85.34% |
| *S. pneumoniae* | GPSC34 | 34,22F,23F,15A | 85.13% |
| *S. pneumoniae* | GPSC37 | 6B,23F | 82.08% |
| *S. pneumoniae* | GPSC40 | 15BC,22A,18C,10A,17F,15A,23F,11A,6B | 82.00% |
| *M. canettii* | NC_019950 |  | 89.44% |
| *M. tuberculosis* | H37Rv |  | 94.30% |
| *M. tuberculosis* | SRR1173640 |  | 94.26% |
| *M. tuberculosis* | ERR144577 |  | 94.27% |
| *M. tuberculosis* | ERR212146 |  | 94.26% |
| *M. tuberculosis* | SRR1172044 |  | 94.30% |
| *M. tuberculosis* | SRR671749 |  | 94.30% |
| *M. tuberculosis* | ERR181435 |  | 94.30% |
| *M. tuberculosis* | SRR1180462 |  | 94.23% |
| *M. tuberculosis* | SRR1019139 |  | 94.26% |
| *M. tuberculosis* | SRR1163294 |  | 94.24% |
| *M. tuberculosis* | SRR998654 |  | 94.24% |
| *M. tuberculosis* | ERR161044 |  | 94.26% |
| *M. tuberculosis* | ERR234202 |  | 94.25% |

#

# Table S5b. References used for commensal / coinfection comparisons

| **Pathogen** | **Sample ID** |
| --- | --- |
| *H influenzae* | CP000672.1 |
| *K pneumoniae* | FO834906.1 |
| *M canetti* | NC_019950.1 |
| *M tuberculosis* | NC_000962.3 |
| *P aeruginosa* | NC_002516.2 |
| *S aureus* | NZ_CP027476.1 |
| *S odontolytica* | NZ_CP133472.1 |
| *S pneumoniae* | NC_017592.1 |

#

# Table S6 *M. tuberculosis* RT-qPCR Oligonucleotide Sequences

| **Oligo Category** | **5’** | **Sequence** | **3’** |
| --- | --- | --- | --- |
| Forward Primer |  | GCCGCTCATGCTCCTTGGAT |  |
| Reverse Primer |  | AGGTCGGTTCGCTGGTCTTG |  |
| Probe | 56-FAM | TGAGTGCCTGCGGCCGCAGCGC | 3BHQ_1 |
| gBlock Gene Fragment |  | ACTCTAGCGTACTGGTGTGACGGCGCCCAACTAGGGAGATTCCTTACCGATGGGAGCAGGCTGATGGCAGCAGGCACGATGCCAGTAGGTGGTCGGCAGCACGTTTTCGAGAAGCTGGCCAGCATCCTGGGCTTGGTCGCCGCGCCGCTCATGCTCCTTGGATTGAGTGCCTGCGGCCGCAGCGCCGGCAAGACCAGCGAACCGACCTGCCCCACGGAGCCGATCGATGCGGCCGACAGCTCGACAACA |  |

#

#

#

# Appendix S1. *M. tuberculosis* extraction methods from sputum

Method A

In a 15-mL falcon tube, 1 mL of sputum was heated on a thermal block at 95℃ for 30 minutes to inactivate. Liquefaction was performed by adding 1 mL of saponin, followed by vortexing for 20 seconds and inverting the tube 4-5 times, then allowing the sample to stand at room temperature for a minimum of 15 minutes but no longer than 20 minutes. Next, 13 mL phosphate buffer (pH 6.8) was added and the tube vortexed and inverted to neutralize the sample and terminate decontamination/liquefaction. The sample was then centrifuged at 3000 g for 15 minutes and supernatant removed. 300 µL PBS was added to the remaining pellet and vortexed. 300 µL of the resuspended pellet was homogenized in 2 cycles of 5.5 m/s for 40 seconds each, placing the sample on ice for 5 minutes after each cycle. The homogenized sample was then centrifuged at 16,000 g for 10 minutes. The supernatant was then transferred to a new 1.5 mL tube and a 1:1 magnetic bead cleanup performed.

Method B

In a 15-mL falcon tube, 1 mL of sputum was heated on a thermal block at 95℃ for 30 minutes to inactivate. Liquefaction was performed by adding 1 mL of 0.5% NALC, followed by vortexing for 20 seconds and inverting the tube 4-5 times, then allowing the sample to stand at room temperature for a minimum of 15 minutes but no longer than 20 minutes. Next, 13 mL phosphate buffer (pH 6.8) was added and the tube vortexed and inverted to neutralize the sample and terminate decontamination/liquefaction. The sample was then centrifuged at 3000 g for 15 minutes and supernatant removed. 300 µL PBS was added to the remaining pellet and vortexed. 300 µL of the resuspended pellet was homogenized in 2 cycles of 5.5 m/s for 40 seconds each, placing the sample on ice for 5 minutes after each cycle. The homogenized sample was then centrifuged at 16,000 g for 10 minutes. The supernatant was then transferred to a new 1.5 mL tube and a 1:1 magnetic bead cleanup performed.

Method C

1 mL of sputum was added to a 15-mL falcon tube, then combined decontamination/liquefaction was performed by adding 1% NALC, followed by vortexing for 20 seconds and inverting the tube 4-5 times, then allowing the sample to stand at room temperature for a minimum of 15 minutes but no longer than 20 minutes. Next, a 13 mL phosphate buffer (pH 6.8) was added and the tube vortexed and inverted to neutralize the sample and terminate decontamination/liquefaction. The sample was then centrifuged at 3000 g for 15 minutes and supernatant removed. 300 µL PBS was added to the remaining pellet and vortexed. 300 µL of the resuspended pellet was homogenized in 2 cycles of 5.5 m/s for 40 seconds each, placing the sample on ice for 5 minutes after each cycle. The homogenized sample was then centrifuged at 16,000 g for 10 minutes. The supernatant was then transferred to a new 1.5 mL tube and a 1:1 magnetic bead cleanup performed.

Method D

1 mL of sputum was added to a 15-mL falcon tube, then combined decontamination/liquefaction was performed by adding 2% NALC, followed by vortexing for 20 seconds and inverting the tube 4-5 times, then allowing the sample to stand at room temperature for a minimum of 15 minutes but no longer than 20 minutes. Next, a 13 mL phosphate buffer (pH 6.8) was added and the tube vortexed and inverted to neutralize the sample and terminate decontamination/liquefaction. The sample was then centrifuged at 3000 g for 15 minutes and supernatant removed. 300 µL PBS was added to the remaining pellet and vortexed. 300 µL of the resuspended pellet was homogenized in 2 cycles of 5.5 m/s for 40 seconds each, placing the sample on ice for 5 minutes after each cycle. The homogenized sample was then centrifuged at 16,000 g for 10 minutes. The supernatant was then transferred to a new 1.5 mL tube and a 1:1 magnetic bead cleanup performed.

Method E

1 mL of sputum was added to a 15-mL falcon tube, then combined decontamination/liquefaction was performed by adding a solution of 4% NaOH/2.9% Na citrate/0.5% NALC followed by vortexing for 20 seconds and inverting the tube 4-5 times, then allowing the sample to stand at room temperature for a minimum of 15 minutes but no longer than 20 minutes. Next, a 13 mL phosphate buffer (pH 6.8) was added and the tube vortexed and inverted to neutralize the sample and terminate decontamination/liquefaction. The sample was then centrifuged at 3000 g for 15 minutes and supernatant removed. 300 µL PBS was added to the remaining pellet and vortexed. 300 µL of the resuspended pellet was homogenized in 2 cycles of 5.5 m/s for 40 seconds each, placing the sample on ice for 5 minutes after each cycle. The homogenized sample was then centrifuged at 16,000 g for 10 minutes. The supernatant was then transferred to a new 1.5 mL tube and a 1:1 magnetic bead cleanup performed.

Method F

1 mL of sputum was added to a 15-mL falcon tube, then combined decontamination/liquefaction was performed by adding a solution of 4% NaOH/2.9% Na citrate/0.5% NALC. followed by vortexing for 20 seconds and inverting the tube 4-5 times, then allowing the sample to stand at room temperature for a minimum of 15 minutes but no longer than 20 minutes. Next, a 13 mL phosphate buffer (pH 6.8) was added and the tube vortexed and inverted to neutralize the sample and terminate decontamination/liquefaction. The sample was then centrifuged at 3000 g for 15 minutes and supernatant removed. 300 µL PBS was added to the remaining pellet and vortexed. 300 µL of the resuspended pellet was homogenized in 2 cycles of 5.5 m/s for 20 seconds each, placing the sample on ice for 5 minutes after each cycle. The homogenized sample was then centrifuged at 16,000 g for 10 minutes. The supernatant was then transferred to a new 1.5 mL tube and a 1:1 magnetic bead cleanup performed.

# Appendix S3. *TB-seq* primer sequences and genomic positions

**Chromosome Start End Primer_ID Pool Strand Seq**

NC_000962.3 14 34 TBseq_1.0_1_LEFT 1 + CGGTTCAGGCTTCACCACAG

NC_000962.3 2059 2079 TBseq_1.0_1_RIGHT 1 - GGCCAACTCTTGTCGTAGCC

NC_000962.3 1828 1847 TBseq_1.0_2_LEFT 2 + TGGGGATCGTTCGCTGGAT

NC_000962.3 3902 3922 TBseq_1.0_2_RIGHT 2 - CCTTCTTCACTTCCGGTGCC

NC_000962.3 3690 3710 TBseq_1.0_3_LEFT 1 + GATCTGGCGATCGTGCGTAG

NC_000962.3 5663 5683 TBseq_1.0_3_RIGHT 1 - CTGAGACCACTCGTACCCGT

NC_000962.3 5445 5464 TBseq_1.0_4_LEFT 2 + GCTTGAGGATGGCGGTGTC

NC_000962.3 7475 7495 TBseq_1.0_4_RIGHT 2 - CGGAAGCCGGAATCGAACAT

NC_000962.3 7301 7321 TBseq_1.0_5_LEFT 1 + ATGACAGACACGACGTTGCC

NC_000962.3 9262 9281 TBseq_1.0_5_RIGHT 1 - GACCAGCTCGTCGTTGTCG

NC_000962.3 9088 9108 TBseq_1.0_6_LEFT 2 + GGCCAACCTGTTAGCCTTCC

NC_000962.3 11095 11115 TBseq_1.0_6_RIGHT 2 - CCCCTCTTCGTCACCGGTTA

NC_000962.3 10878 10898 TBseq_1.0_7_LEFT 1 + GCGAGTACGGGCCTATAGCT

NC_000962.3 12862 12882 TBseq_1.0_7_RIGHT 1 - GGTTCAGGTGCGGAGTCTTG

NC_000962.3 12696 12716 TBseq_1.0_8_LEFT 2 + GCTTCATGATCCAGGGTGGC

NC_000962.3 14663 14683 TBseq_1.0_8_RIGHT 2 - TCGTGTAGGGCGGGCTATAG

NC_000962.3 14447 14467 TBseq_1.0_9_LEFT 1 + GGTGGTTACCCTGCAAGACG

NC_000962.3 16512 16532 TBseq_1.0_9_RIGHT 1 - ACGCCAGGACTTAGACGACA

NC_000962.3 16287 16307 TBseq_1.0_10_LEFT 2 + GGATTGTCGAGTCCGGCTTC

NC_000962.3 18305 18325 TBseq_1.0_10_RIGHT 2 - CATCTTGATCACCCCCACCG

NC_000962.3 18124 18144 TBseq_1.0_11_LEFT 1 + CCCGAAACCGCTTCATACCC

NC_000962.3 20144 20164 TBseq_1.0_11_RIGHT 1 - CCATGACGCAGGTCTTCACC

NC_000962.3 19979 19999 TBseq_1.0_12_LEFT 2 + CGCGTACACCTCAGGATTGG

NC_000962.3 21955 21974 TBseq_1.0_12_RIGHT 2 - GCACTCTCGATGACGCCAT

NC_000962.3 21760 21780 TBseq_1.0_13_LEFT 1 + GTGGGGATGGCAGGAGTAGT

NC_000962.3 23734 23754 TBseq_1.0_13_RIGHT 1 - GACGGAAAGGACGCCAGATG

NC_000962.3 23520 23540 TBseq_1.0_14_LEFT 2 + CAGTCAACGCACCTTCGGTC

NC_000962.3 25526 25546 TBseq_1.0_14_RIGHT 2 - CGCTCACACTGTCCCGATAC

NC_000962.3 25347 25367 TBseq_1.0_15_LEFT 1 + GGACGATCGAGCCTCCAAAG

NC_000962.3 27318 27336 TBseq_1.0_15_RIGHT 1 - AAGCGCGGATCCAATGCC

NC_000962.3 27140 27160 TBseq_1.0_16_LEFT 2 + CTGGAATTGATCTCGCCGCC

NC_000962.3 29162 29181 TBseq_1.0_16_RIGHT 2 - TCTGCACGTTGTTGCCCAG

NC_000962.3 28950 28970 TBseq_1.0_17_LEFT 1 + GTTCCGGTTTGGTCCAGTGG

NC_000962.3 30994 31014 TBseq_1.0_17_RIGHT 1 - GAGCTTTGCTAGGGCCAAGG

NC_000962.3 30861 30880 TBseq_1.0_18_LEFT 2 + GGCACACCGATCGCAGATG

NC_000962.3 32751 32771 TBseq_1.0_18_RIGHT 2 - TGCAACACGTCGTGATACCG

NC_000962.3 32545 32564 TBseq_1.0_19_LEFT 1 + GATGACGAAGCCCGACTGC

NC_000962.3 34508 34528 TBseq_1.0_19_RIGHT 1 - GCACTCGAGGAAACGGAAGG

NC_000962.3 34333 34353 TBseq_1.0_20_LEFT 2 + GAGGACTCGGGGATCAACGA

NC_000962.3 36353 36374 TBseq_1.0_20_RIGHT 2 - CGTGATACTCGGCCTGATAGC

NC_000962.3 36186 36206 TBseq_1.0_21_LEFT 1 + GGACTACATCCGGCACAACG

NC_000962.3 38190 38209 TBseq_1.0_21_RIGHT 1 - GAAGGTGGCGGCGAATCTC

NC_000962.3 38042 38062 TBseq_1.0_22_LEFT 2 + GGGCTACCCACACGTTCATG

NC_000962.3 39981 40001 TBseq_1.0_22_RIGHT 2 - CAGCCTTGATTCCCGAGCAC

NC_000962.3 39845 39865 TBseq_1.0_23_LEFT 1 + AACAGTTCGTCGGGAAAGGC

NC_000962.3 41800 41820 TBseq_1.0_23_RIGHT 1 - GCCCCACCAAAACGTCAGAT

NC_000962.3 41656 41676 TBseq_1.0_24_LEFT 2 + GATGGTCGATCTGGATGCCG

NC_000962.3 43624 43644 TBseq_1.0_24_RIGHT 2 - GTATAGCGGTACCGTGGCAC

NC_000962.3 43485 43505 TBseq_1.0_25_LEFT 1 + CAACGGAACCGAGACCAACC

NC_000962.3 45403 45423 TBseq_1.0_25_RIGHT 1 - GGATCGGTGTAGCGCAGTTC

NC_000962.3 45220 45240 TBseq_1.0_26_LEFT 2 + CTGCCTGATGTCCCGGACTA

NC_000962.3 47231 47251 TBseq_1.0_26_RIGHT 2 - CCATGGATCCGGCAGAACTG

NC_000962.3 47064 47083 TBseq_1.0_27_LEFT 1 + GTGAATTCGCTGTCCGCCA

NC_000962.3 49021 49041 TBseq_1.0_27_RIGHT 1 - CCTACGGTGGACCCATGACT

NC_000962.3 48873 48893 TBseq_1.0_28_LEFT 2 + GCGGAAAGAAGTGGTCCCAG

NC_000962.3 50774 50794 TBseq_1.0_28_RIGHT 2 - GGCATCGGCAAGTACTACGC

NC_000962.3 50637 50657 TBseq_1.0_29_LEFT 1 + CGATAGCACACTGGGCGTAG

NC_000962.3 52559 52579 TBseq_1.0_29_RIGHT 1 - TCTCGATGGAGGAACACCGG

NC_000962.3 52427 52445 TBseq_1.0_30_LEFT 2 + GCCCAACTCGGTGCGTTT

NC_000962.3 54351 54371 TBseq_1.0_30_RIGHT 2 - CTGTCTCGGGAAACACCTGC

NC_000962.3 54121 54141 TBseq_1.0_31_LEFT 1 + ATCATCTACTTCGGCCGGGG

NC_000962.3 56166 56186 TBseq_1.0_31_RIGHT 1 - TAGATCCCAGTCAGCACCGG

NC_000962.3 56027 56047 TBseq_1.0_32_LEFT 2 + CGATACGGTGCCGCTCTATG

NC_000962.3 57938 57959 TBseq_1.0_32_RIGHT 2 - GATAGAACTCCAACGCGAGCC

NC_000962.3 57800 57820 TBseq_1.0_33_LEFT 1 + GGATCGACATGGGATTGCGG

NC_000962.3 59768 59787 TBseq_1.0_33_RIGHT 1 - CGGCCTTGATGTGCGTCTT

NC_000962.3 59539 59559 TBseq_1.0_34_LEFT 2 + GAAGCAGGCTGACGAGATCC

NC_000962.3 61599 61619 TBseq_1.0_34_RIGHT 2 - CAAGATTCTGGTGGACGCGG

NC_000962.3 61447 61467 TBseq_1.0_35_LEFT 1 + GGTGGAGGTGTCGGAGTTCT

NC_000962.3 63437 63456 TBseq_1.0_35_RIGHT 1 - TGATCGCTTGCCATCGACG

NC_000962.3 63291 63311 TBseq_1.0_36_LEFT 2 + CACCCATGCCGACAATCTCC

NC_000962.3 65228 65248 TBseq_1.0_36_RIGHT 2 - CAGCCGGACATGACGAAAGG

NC_000962.3 65085 65103 TBseq_1.0_37_LEFT 1 + CACCGCCGCTGACACAAT

NC_000962.3 67027 67047 TBseq_1.0_37_RIGHT 1 - GAAAGAGCCTCCCAGCCAGA

NC_000962.3 66814 66834 TBseq_1.0_38_LEFT 2 + GGTTCGGGTGGTCGACAATG

NC_000962.3 68877 68896 TBseq_1.0_38_RIGHT 2 - GTACAAAGAACGGCCGCGA

NC_000962.3 68703 68722 TBseq_1.0_39_LEFT 1 + TTGCTGCTGTTTGGACCCC

NC_000962.3 70636 70656 TBseq_1.0_39_RIGHT 1 - CTTGACGGTTCCGGGAAAGG

NC_000962.3 70431 70451 TBseq_1.0_40_LEFT 2 + GCCTACACCACCTTGGACAC

NC_000962.3 72427 72447 TBseq_1.0_40_RIGHT 2 - TCGCTGGCAGATGTGTTGAC

NC_000962.3 72287 72306 TBseq_1.0_41_LEFT 1 + CCTCCAGCGCTGCATTGAA

NC_000962.3 74207 74227 TBseq_1.0_41_RIGHT 1 - GGTAGCCGCCATCAAGGAAC

NC_000962.3 74042 74062 TBseq_1.0_42_LEFT 2 + CGTACTCCTTCACCGCCTTG

NC_000962.3 75980 75999 TBseq_1.0_42_RIGHT 2 - CATCAGCGGCGCCAGTATG

NC_000962.3 75777 75798 TBseq_1.0_43_LEFT 1 + CATTTCGACGACCTCCAGTGG

NC_000962.3 77745 77766 TBseq_1.0_43_RIGHT 1 - CTTCTCCCACAACGACTTCCG

NC_000962.3 77611 77631 TBseq_1.0_44_LEFT 2 + CTGATGGTCATGTCCGGTGC

NC_000962.3 79573 79593 TBseq_1.0_44_RIGHT 2 - CACAGTGAGCCCGTCTGATC

NC_000962.3 79381 79401 TBseq_1.0_45_LEFT 1 + CAGACAGCGCGGGTCATATC

NC_000962.3 81383 81403 TBseq_1.0_45_RIGHT 1 - GTCACGTAGCCGCTCAAGAG

NC_000962.3 81185 81205 TBseq_1.0_46_LEFT 2 + GGCAGCCGAATATCACGTCC

NC_000962.3 83165 83185 TBseq_1.0_46_RIGHT 2 - CCCAGAAACCACCAGTTCCG

NC_000962.3 82989 83009 TBseq_1.0_47_LEFT 1 + CGACGGTAGGCCAGAGGATT

NC_000962.3 84953 84973 TBseq_1.0_47_RIGHT 1 - ATCCACCCACGACAGGTAGG

NC_000962.3 84819 84839 TBseq_1.0_48_LEFT 2 + GAACATCGCCGCCTACCATC

NC_000962.3 86730 86750 TBseq_1.0_48_RIGHT 2 - CCATCACGTCTTGCTCCACG

NC_000962.3 86534 86554 TBseq_1.0_49_LEFT 1 + TCAAGAGACGCACCCAGGAG

NC_000962.3 88492 88512 TBseq_1.0_49_RIGHT 1 - CAGTCTGATCAGTGCGGGTC

NC_000962.3 88268 88288 TBseq_1.0_50_LEFT 2 + GGATGTCGCCGTATTCTCCG

NC_000962.3 90319 90339 TBseq_1.0_50_RIGHT 2 - ATCTCGACGTCGACGGGTAC

NC_000962.3 90152 90171 TBseq_1.0_51_LEFT 1 + ATGCGTTGTTGGTGACCGG

NC_000962.3 92094 92114 TBseq_1.0_51_RIGHT 1 - GTGACCTCGATGTCGGTGTC

NC_000962.3 91833 91853 TBseq_1.0_52_LEFT 2 + GTCAAGTTCACCGGTCTGGG

NC_000962.3 93866 93886 TBseq_1.0_52_RIGHT 2 - TCAACACGCCGATCACGATG

NC_000962.3 93703 93721 TBseq_1.0_53_LEFT 1 + TGCTGATCGCGCTGTTCG

NC_000962.3 95668 95688 TBseq_1.0_53_RIGHT 1 - CCGTACAGGTCCGCCATTTC

NC_000962.3 95532 95552 TBseq_1.0_54_LEFT 2 + CAGTCTGCGTGTCGTGTACC

NC_000962.3 97514 97534 TBseq_1.0_54_RIGHT 2 - GACTTCGCGAAACCCGTCAT

NC_000962.3 97333 97353 TBseq_1.0_55_LEFT 1 + GTATCGCATCACCAGCAGGG

NC_000962.3 99360 99380 TBseq_1.0_55_RIGHT 1 - ATTGCGCCACCTGATCCAAG

NC_000962.3 99152 99170 TBseq_1.0_56_LEFT 2 + AGCGGTTGTTCGGTGTGC

NC_000962.3 101135 101154 TBseq_1.0_56_RIGHT 2 - TCGATGGGCGAGTGATTGC

NC_000962.3 100926 100946 TBseq_1.0_57_LEFT 1 + GTCGGTGATGTTTGGGGTCG

NC_000962.3 102946 102966 TBseq_1.0_57_RIGHT 1 - CGGTCCTGGCAATCATGGTG

NC_000962.3 101660 101679 TBseq_1.0_58_LEFT 2 + CGGTGTTTGTTCCCGCTGT

NC_000962.3 103643 103662 TBseq_1.0_58_RIGHT 2 - TGCTCGCACAAGCCACAAC

NC_000962.3 103390 103409 TBseq_1.0_59_LEFT 1 + GCTGGCCACCTGGGTAAAC

NC_000962.3 105285 105306 TBseq_1.0_59_RIGHT 1 - GGACTCACCTCCTTGCCTAGG

NC_000962.3 105216 105233 TBseq_1.0_60_LEFT 2 + TTTCCTGGGCGCGATGG

NC_000962.3 107200 107220 TBseq_1.0_60_RIGHT 2 - CGCTGGGTCGGATCTTGATG

NC_000962.3 107001 107020 TBseq_1.0_61_LEFT 1 + CAAAAACCGGCGCGTTCTG

NC_000962.3 109016 109036 TBseq_1.0_61_RIGHT 1 - CCGTATCCAACTAGGCGCAG

NC_000962.3 108827 108847 TBseq_1.0_62_LEFT 2 + CACATCGGTGGACTGTGGTG

NC_000962.3 110864 110883 TBseq_1.0_62_RIGHT 2 - CCGACGCAAATGGGGGAAA

NC_000962.3 110701 110721 TBseq_1.0_63_LEFT 1 + GGTCCCGCTTAATGTGCTGG

NC_000962.3 112654 112674 TBseq_1.0_63_RIGHT 1 - ACACTCCCAGACTCACGAGG

NC_000962.3 112492 112512 TBseq_1.0_64_LEFT 2 + GCGTGTCGATATCGCTGAGG

NC_000962.3 114475 114495 TBseq_1.0_64_RIGHT 2 - CCAACATCTGCGGGATCGAC

NC_000962.3 114304 114323 TBseq_1.0_65_LEFT 1 + AGCCCAAGCAATCGACGTG

NC_000962.3 116232 116250 TBseq_1.0_65_RIGHT 1 - TGAGGTCGCTGGCATGCA

NC_000962.3 116089 116109 TBseq_1.0_66_LEFT 2 + CACCACCCTAAACGCCGATC

NC_000962.3 118021 118041 TBseq_1.0_66_RIGHT 2 - CCAGCAGGTGAATCCGGAAC

NC_000962.3 117796 117815 TBseq_1.0_67_LEFT 1 + AATTGGCCTGCTGGGTGTG

NC_000962.3 119832 119852 TBseq_1.0_67_RIGHT 1 - CGGGGTCAGATCCAGGACTT

NC_000962.3 119653 119673 TBseq_1.0_68_LEFT 2 + AGCCTACAACGCGATGCTTC

NC_000962.3 121653 121673 TBseq_1.0_68_RIGHT 2 - GGAAACGCTGATCTCGGTCG

NC_000962.3 121474 121494 TBseq_1.0_69_LEFT 1 + CTTGGACTTGGCTCGTGCTT

NC_000962.3 123412 123431 TBseq_1.0_69_RIGHT 1 - AACGATGCCACGATGTCGC

NC_000962.3 123229 123249 TBseq_1.0_70_LEFT 2 + GCAGTGTCGACGCTACCTTG

NC_000962.3 125197 125217 TBseq_1.0_70_RIGHT 2 - CGAGCCACATGACCTGATCC

NC_000962.3 124996 125016 TBseq_1.0_71_LEFT 1 + CAACTCACGGAGGGGTCGTA

NC_000962.3 127034 127053 TBseq_1.0_71_RIGHT 1 - GTTGATGCTCAAGGGCGCT

NC_000962.3 126823 126843 TBseq_1.0_72_LEFT 2 + CGACATAGCCGATCAGCTCG

NC_000962.3 128807 128828 TBseq_1.0_72_RIGHT 2 - GGACCGGTATTGCTGATCACC

NC_000962.3 128612 128632 TBseq_1.0_73_LEFT 1 + GTGTACCGCGGATTCACTGG

NC_000962.3 130560 130580 TBseq_1.0_73_RIGHT 1 - CAGGATTAGGCGCGGTATCG

NC_000962.3 129457 129474 TBseq_1.0_74_LEFT 2 + CCGCGGCGATCAACGAA

NC_000962.3 131361 131381 TBseq_1.0_74_RIGHT 2 - CAGGGACCTCTGCCGTTTTC

NC_000962.3 131153 131173 TBseq_1.0_75_LEFT 1 + CGACTAGGGAACACTCTGCG

NC_000962.3 133188 133207 TBseq_1.0_75_RIGHT 1 - TGCCGTTCCAGACCCATCA

NC_000962.3 133012 133031 TBseq_1.0_76_LEFT 2 + CGAATGTATGCGCGTGGGT

NC_000962.3 134974 134994 TBseq_1.0_76_RIGHT 2 - CGTACGATATGGTGCCCAGC

NC_000962.3 134780 134798 TBseq_1.0_77_LEFT 1 + ATTGCCCGTCTGTTGCCG

NC_000962.3 136778 136798 TBseq_1.0_77_RIGHT 1 - GAAGATACGAGCGACGACGG

NC_000962.3 136584 136603 TBseq_1.0_78_LEFT 2 + CGAAGCACTACGTCGCGTG

NC_000962.3 138563 138583 TBseq_1.0_78_RIGHT 2 - TACGGTTCCACGTCTGTCCC

NC_000962.3 138401 138421 TBseq_1.0_79_LEFT 1 + TGCCAGTGTCCAGATAGGGG

NC_000962.3 140412 140432 TBseq_1.0_79_RIGHT 1 - GGTTCGTGTTACCTGGAGCG

NC_000962.3 140265 140285 TBseq_1.0_80_LEFT 2 + GCTAGCCGACCACCTCAATG

NC_000962.3 142220 142240 TBseq_1.0_80_RIGHT 2 - GACCGTCACTCATCGACTGC

NC_000962.3 142055 142074 TBseq_1.0_81_LEFT 1 + TTGCCCGAGCGCTCATAAC

NC_000962.3 144016 144035 TBseq_1.0_81_RIGHT 1 - TCCATGTGTTCGGTGTGCG

NC_000962.3 143860 143880 TBseq_1.0_82_LEFT 2 + CCGATCGTGTGGTCATGGTG

NC_000962.3 145860 145881 TBseq_1.0_82_RIGHT 2 - GTGCTGGTACCCGACGATTTC

NC_000962.3 145687 145707 TBseq_1.0_83_LEFT 1 + AACGAACGGGTGAAGGATGC

NC_000962.3 147648 147668 TBseq_1.0_83_RIGHT 1 - GCAAGACCACCCTCATCGAG

NC_000962.3 147503 147523 TBseq_1.0_84_LEFT 2 + CCTTGATGCCGTCGTAAGCC

NC_000962.3 149442 149462 TBseq_1.0_84_RIGHT 2 - AATCTGAACGTAGCGCGACC

NC_000962.3 149250 149270 TBseq_1.0_85_LEFT 1 + GCGAAGTCTGGACAGTCAGC

NC_000962.3 151254 151273 TBseq_1.0_85_RIGHT 1 - GAACCGGTCCTGCGACAAG

NC_000962.3 151050 151070 TBseq_1.0_86_LEFT 2 + GGCGATCTGGACTACGTTGG

NC_000962.3 153014 153033 TBseq_1.0_86_RIGHT 2 - AACCGAAACCCGTCGATGC

NC_000962.3 152850 152871 TBseq_1.0_87_LEFT 1 + CCGGATCATCTTCGTCGACAC

NC_000962.3 154863 154883 TBseq_1.0_87_RIGHT 1 - CGTTCGCCTCATACTCGGTC

NC_000962.3 154640 154660 TBseq_1.0_88_LEFT 2 + GGGTATGTGACGCCGAACAG

NC_000962.3 156655 156675 TBseq_1.0_88_RIGHT 2 - CCTACTGGAACGAGCAGCTG

NC_000962.3 156518 156537 TBseq_1.0_89_LEFT 1 + GTTCCACACATCGGCAGCA

NC_000962.3 158450 158470 TBseq_1.0_89_RIGHT 1 - GCCACTGAGCTGCACAAGAT

NC_000962.3 158311 158331 TBseq_1.0_90_LEFT 2 + CGGTCAGAATTCGGCGATCG

NC_000962.3 160250 160270 TBseq_1.0_90_RIGHT 2 - CATCGTACTGATCCGCCAGC

NC_000962.3 160092 160111 TBseq_1.0_91_LEFT 1 + GGCGATCCAACCATCACCG

NC_000962.3 162016 162036 TBseq_1.0_91_RIGHT 1 - TCGTCAGCCATCTCGGTCTT

NC_000962.3 161832 161851 TBseq_1.0_92_LEFT 2 + CAGGATCCACGTTGCGGAC

NC_000962.3 163862 163882 TBseq_1.0_92_RIGHT 2 - GCTCGTCTAGTTCACTGCCG

NC_000962.3 163704 163724 TBseq_1.0_93_LEFT 1 + CCTTTCCACGGCAAGAGCAT

NC_000962.3 165640 165659 TBseq_1.0_93_RIGHT 1 - TGTTCGTCGCGCTGATCAC

NC_000962.3 165436 165456 TBseq_1.0_94_LEFT 2 + ACCCCCGACGTCGAATACAT

NC_000962.3 167486 167506 TBseq_1.0_94_RIGHT 2 - GCAAGCAGGTCTTCGAGGTC

NC_000962.3 167340 167360 TBseq_1.0_95_LEFT 1 + CGCAGCGAGATGATCATCCC

NC_000962.3 169334 169354 TBseq_1.0_95_RIGHT 1 - CTGTGGTTCGTGGTGATCGG

NC_000962.3 169107 169127 TBseq_1.0_96_LEFT 2 + CGATGACGATCCACAACGGG

NC_000962.3 171120 171140 TBseq_1.0_96_RIGHT 2 - CGGTTAGGTCTCGGTCAGGT

NC_000962.3 170902 170922 TBseq_1.0_97_LEFT 1 + TGCGAGCAACCCGAGAATTC

NC_000962.3 172953 172973 TBseq_1.0_97_RIGHT 1 - GGTAGACCAGTTCCTGCACG

NC_000962.3 172775 172795 TBseq_1.0_98_LEFT 2 + TGATGTACCTACCGGCCGAG

NC_000962.3 174802 174822 TBseq_1.0_98_RIGHT 2 - TCCGCAATCACAATTCGGGG

NC_000962.3 174634 174654 TBseq_1.0_99_LEFT 1 + GGGTTTCGAGGAGTTCAGCC

NC_000962.3 176637 176656 TBseq_1.0_99_RIGHT 1 - CACAACCTTGCCGAGCACA

NC_000962.3 175615 175635 TBseq_1.0_100_LEFT 2 + TCGGTACAAGATGTTGCGGC

NC_000962.3 177517 177537 TBseq_1.0_100_RIGHT 2 - CGATGCTCGGGTCATTCAGC

NC_000962.3 180959 180978 TBseq_1.0_101_LEFT 1 + CCGAACCGATTCCTGCCAC

NC_000962.3 182888 182908 TBseq_1.0_101_RIGHT 1 - CACCGGCAACATGGAGATCC

NC_000962.3 182699 182719 TBseq_1.0_102_LEFT 2 + CTCCCGACGTCCACCACTTA

NC_000962.3 184640 184660 TBseq_1.0_102_RIGHT 2 - CCAGCCTGCCGTCTTTGATC

NC_000962.3 184443 184463 TBseq_1.0_103_LEFT 1 + GTGGAAGCGATGAAGCCTGG

NC_000962.3 186456 186476 TBseq_1.0_103_RIGHT 1 - CAACGCCTTGAGTTCCTCGG

NC_000962.3 185465 185485 TBseq_1.0_104_LEFT 2 + TCGATCTCGTTCTGGGGGTC

NC_000962.3 187396 187416 TBseq_1.0_104_RIGHT 2 - CGAGTTCCAAGCGGAGACTG

NC_000962.3 186962 186982 TBseq_1.0_105_LEFT 1 + TCGACATTTACCGTCAGGCG

NC_000962.3 188906 188925 TBseq_1.0_105_RIGHT 1 - CCGCGATAACGATTGGCCG

NC_000962.3 188839 188858 TBseq_1.0_106_LEFT 2 + CTGGTCTCCTGTTCGACGC

NC_000962.3 190739 190759 TBseq_1.0_106_RIGHT 2 - CACTTCGGCGACCTCTTCAG

NC_000962.3 190596 190615 TBseq_1.0_107_LEFT 1 + AACCGCGAGCATGCTAACC

NC_000962.3 192533 192552 TBseq_1.0_107_RIGHT 1 - GTGCGGTACTCAACGTCGG

NC_000962.3 192358 192379 TBseq_1.0_108_LEFT 2 + CTGCTGCGGTGTGTAGATCTC

NC_000962.3 194373 194393 TBseq_1.0_108_RIGHT 2 - GATCGAGTTCGCGTTCCACC

NC_000962.3 194168 194187 TBseq_1.0_109_LEFT 1 + GTGCCCCATCGGTGAACTG

NC_000962.3 196131 196151 TBseq_1.0_109_RIGHT 1 - CCCGGTAGACAATTTCGCCC

NC_000962.3 195996 196016 TBseq_1.0_110_LEFT 2 + CGAGATGTCACCGGTCACCT

NC_000962.3 197931 197950 TBseq_1.0_110_RIGHT 2 - GAAGCCCTGGATTGCGACC

NC_000962.3 197713 197733 TBseq_1.0_111_LEFT 1 + CGTCAATATGGAGGTGCGGC

NC_000962.3 199691 199709 TBseq_1.0_111_RIGHT 1 - GTTCACCCGCGGCAGATT

NC_000962.3 199485 199505 TBseq_1.0_112_LEFT 2 + GCTACTCGCTGAGGACGAAC

NC_000962.3 201513 201533 TBseq_1.0_112_RIGHT 2 - TTGGCCTGGGCTAGTAGGTG

NC_000962.3 201340 201359 TBseq_1.0_113_LEFT 1 + ACGCGTTCTTCGACGTCAC

NC_000962.3 203353 203373 TBseq_1.0_113_RIGHT 1 - CCAACCCATCCAACGGATCG

NC_000962.3 203198 203218 TBseq_1.0_114_LEFT 2 + GACAGGTTGACCCACTCCGA

NC_000962.3 205169 205189 TBseq_1.0_114_RIGHT 2 - GTTGGGGTTCGGTGTGTACC

NC_000962.3 204949 204968 TBseq_1.0_115_LEFT 1 + TATCTGGTGGGTGCGCTCA

NC_000962.3 206977 206995 TBseq_1.0_115_RIGHT 1 - GCCGCAATGCCAACAAGC

NC_000962.3 206776 206795 TBseq_1.0_116_LEFT 2 + GAACCCTGCCGATGCGAAT

NC_000962.3 208750 208769 TBseq_1.0_116_RIGHT 2 - ACCCCGGAGATGCGAATCA

NC_000962.3 208562 208582 TBseq_1.0_117_LEFT 1 + GCGGATCTCCCATGAATCGC

NC_000962.3 210584 210604 TBseq_1.0_117_RIGHT 1 - GTCATCCTATGCCGCTGCTC

NC_000962.3 210401 210421 TBseq_1.0_118_LEFT 2 + CGGGTAGATCTCGGAGGCTT

NC_000962.3 212405 212424 TBseq_1.0_118_RIGHT 2 - CCGCCTCACTCTGGAAGGT

NC_000962.3 212181 212201 TBseq_1.0_119_LEFT 1 + CACCGTCCGAATCGCTTTGA

NC_000962.3 214175 214192 TBseq_1.0_119_RIGHT 1 - CAGCACGACCACCGCTT

NC_000962.3 214035 214055 TBseq_1.0_120_LEFT 2 + CCTACGCAGAGTCTCCGACA

NC_000962.3 215998 216016 TBseq_1.0_120_RIGHT 2 - CCTGTGCCGCCATGGTTT

NC_000962.3 215856 215876 TBseq_1.0_121_LEFT 1 + CAGAAGGTCGGTTCGGTTCG

NC_000962.3 217768 217788 TBseq_1.0_121_RIGHT 1 - CCGGTCACAATCTCCGCAAC

NC_000962.3 217541 217561 TBseq_1.0_122_LEFT 2 + GAGCAGACATCGCGATCGTG

NC_000962.3 219560 219579 TBseq_1.0_122_RIGHT 2 - AATGCGCGATCGTCGACTC

NC_000962.3 219410 219430 TBseq_1.0_123_LEFT 1 + CGGGCGTAATCTTGGAGCAG

NC_000962.3 221386 221406 TBseq_1.0_123_RIGHT 1 - CTTCTCGCTGGTTTCCCGTG

NC_000962.3 221187 221207 TBseq_1.0_124_LEFT 2 + GTGACGTCTCGTTCGCTACC

NC_000962.3 223202 223221 TBseq_1.0_124_RIGHT 2 - GGTGTGGCGTTCACCGAAT

NC_000962.3 223039 223059 TBseq_1.0_125_LEFT 1 + GTGTACGTGGGCCGAATCTG

NC_000962.3 225022 225042 TBseq_1.0_125_RIGHT 1 - GATCCACGCGCTAATCTCCG

NC_000962.3 224859 224878 TBseq_1.0_126_LEFT 2 + AGGTAAGCGGCCATGTCCT

NC_000962.3 226829 226849 TBseq_1.0_126_RIGHT 2 - CGATCACCCGGTGGTTGAAC

NC_000962.3 226624 226644 TBseq_1.0_127_LEFT 1 + CTGCAGAACGTACCTCCGGA

NC_000962.3 228589 228608 TBseq_1.0_127_RIGHT 1 - AACCAGGTCATCGGCGAGA

NC_000962.3 228399 228419 TBseq_1.0_128_LEFT 2 + GAATTCAGGAGGTGCTGCGG

NC_000962.3 230422 230442 TBseq_1.0_128_RIGHT 2 - GCTGAGTGATTTCCGGCGAA

NC_000962.3 230288 230307 TBseq_1.0_129_LEFT 1 + CTAGCCATCGCCGAACACG

NC_000962.3 232213 232233 TBseq_1.0_129_RIGHT 1 - CATCATCGGCTCCTTTCGGG

NC_000962.3 232028 232047 TBseq_1.0_130_LEFT 2 + GCTGGTCGGACTTGACTGC

NC_000962.3 234015 234034 TBseq_1.0_130_RIGHT 2 - ACCGGATCTCTGCCAGCAT

NC_000962.3 233827 233846 TBseq_1.0_131_LEFT 1 + GACTTGCCCAGAAGACGGC

NC_000962.3 235769 235789 TBseq_1.0_131_RIGHT 1 - GTTTCGACTGGGTCAGCTGG

NC_000962.3 235566 235586 TBseq_1.0_132_LEFT 2 + GTGCGGCCGTAGAATTCGAA

NC_000962.3 237525 237545 TBseq_1.0_132_RIGHT 2 - CAAGATGCACCACTTCCCGG

NC_000962.3 237310 237329 TBseq_1.0_133_LEFT 1 + GTTTCGACGTGCTCGGTGT

NC_000962.3 239341 239359 TBseq_1.0_133_RIGHT 1 - GCGCCGCGTTGATCATGA

NC_000962.3 239114 239134 TBseq_1.0_134_LEFT 2 + GTCACCGAGGTCGACTTTCG

NC_000962.3 241152 241171 TBseq_1.0_134_RIGHT 2 - GTTTCGAAGTCGCCGGGTC

NC_000962.3 240926 240945 TBseq_1.0_135_LEFT 1 + CCGCAGCGACACCACATAC

NC_000962.3 243001 243021 TBseq_1.0_135_RIGHT 1 - CCTGGGTGAGCTTGTATCGC

NC_000962.3 242783 242802 TBseq_1.0_136_LEFT 2 + CCCCAGATGCGTTGCTGAC

NC_000962.3 244821 244841 TBseq_1.0_136_RIGHT 2 - CACCACACGGATGCAGATCG

NC_000962.3 244615 244635 TBseq_1.0_137_LEFT 1 + CGACCGTATGATTGGCAGGG

NC_000962.3 246624 246644 TBseq_1.0_137_RIGHT 1 - GTTCCTCGCGATCTTCGGTC

NC_000962.3 246480 246498 TBseq_1.0_138_LEFT 2 + TGATCACCGTGCGCCGTA

NC_000962.3 248423 248443 TBseq_1.0_138_RIGHT 2 - TCTTCTTCCCCGATCCGTGG

NC_000962.3 248231 248250 TBseq_1.0_139_LEFT 1 + AATCGGCAGCAGTTCGGTG

NC_000962.3 250186 250203 TBseq_1.0_139_RIGHT 1 - CAAACCCAGCCCGGCAA

NC_000962.3 250047 250067 TBseq_1.0_140_LEFT 2 + TATCGCTCGGGGATCCCTTC

NC_000962.3 251951 251971 TBseq_1.0_140_RIGHT 2 - CGGGGTTGAGCCTGATGAAC

NC_000962.3 251750 251770 TBseq_1.0_141_LEFT 1 + CGAGCACCCGATAACTACGC

NC_000962.3 253714 253735 TBseq_1.0_141_RIGHT 1 - CGGTTGGGAAGAGAGGCTTTC

NC_000962.3 253565 253585 TBseq_1.0_142_LEFT 2 + AGATGAGTTCGACGCCCTGA

NC_000962.3 255502 255522 TBseq_1.0_142_RIGHT 2 - CGACCTTGACGAGTTGCCTC

NC_000962.3 255362 255382 TBseq_1.0_143_LEFT 1 + CCGGTAGCTGTCCTTCCCTA

NC_000962.3 257331 257351 TBseq_1.0_143_RIGHT 1 - CGGTCAGGAACAAGTAGCCG

NC_000962.3 257134 257152 TBseq_1.0_144_LEFT 2 + TCGGTCGGCAAGCCCATA

NC_000962.3 259163 259182 TBseq_1.0_144_RIGHT 2 - GGCTTTGACCCGCTGAGTC

NC_000962.3 258971 258991 TBseq_1.0_145_LEFT 1 + AGGTCAGGTGTTCGACTGGG

NC_000962.3 260917 260937 TBseq_1.0_145_RIGHT 1 - GCTGGATCACTCATCAGGCC

NC_000962.3 260700 260720 TBseq_1.0_146_LEFT 2 + GTACGGGACATTCGAAGCCC

NC_000962.3 262672 262691 TBseq_1.0_146_RIGHT 2 - GGTGAGCAGCAACCACCTG

NC_000962.3 262445 262465 TBseq_1.0_147_LEFT 1 + CTGTTGTACCCGCTCTACGC

NC_000962.3 264483 264501 TBseq_1.0_147_RIGHT 1 - AACACCGTCAGCCAACGC

NC_000962.3 264306 264326 TBseq_1.0_148_LEFT 2 + CTCAACTACCACATCCGGCC

NC_000962.3 266325 266346 TBseq_1.0_148_RIGHT 2 - CCAGCAAAAGAGTGTCCTGCT

NC_000962.3 266175 266196 TBseq_1.0_149_LEFT 1 + CCCGACACTATGTTCGCTGAG

NC_000962.3 268143 268163 TBseq_1.0_149_RIGHT 1 - GCTGTCCTACACGGTTTGGC

NC_000962.3 267916 267935 TBseq_1.0_150_LEFT 2 + GACGGATGTCAGCCACCAC

NC_000962.3 269975 269995 TBseq_1.0_150_RIGHT 2 - CAAACCTCCGGTGCATCGTC

NC_000962.3 269828 269848 TBseq_1.0_151_LEFT 1 + CAGACTTAATCCTGGGCGCG

NC_000962.3 271804 271824 TBseq_1.0_151_RIGHT 1 - CGTCGTTTAGCCTTCGGACC

NC_000962.3 271549 271566 TBseq_1.0_152_LEFT 2 + TCGGAACCAGCGCACGT

NC_000962.3 273534 273554 TBseq_1.0_152_RIGHT 2 - AGGCTCCACATCTGGGTCAG

NC_000962.3 273398 273418 TBseq_1.0_153_LEFT 1 + CATCATGCCGGCCTATGTGG

NC_000962.3 275338 275358 TBseq_1.0_153_RIGHT 1 - GGTGGTTATCGGACACTGCG

NC_000962.3 275184 275203 TBseq_1.0_154_LEFT 2 + ACCATCTTGTCGGCATGGC

NC_000962.3 277103 277123 TBseq_1.0_154_RIGHT 2 - CCTTTTCCACCCCTTTGGCC

NC_000962.3 276895 276914 TBseq_1.0_155_LEFT 1 + TGGTGACCGACTTCAGCCA

NC_000962.3 278883 278903 TBseq_1.0_155_RIGHT 1 - CCTGGGTGTGTTTGGCTTCC

NC_000962.3 278713 278733 TBseq_1.0_156_LEFT 2 + GCGGACTGGGAGAAGTTGTC

NC_000962.3 280692 280712 TBseq_1.0_156_RIGHT 2 - GGGTTTTCGCTACTACGCCG

NC_000962.3 280494 280512 TBseq_1.0_157_LEFT 1 + CGTTCGACGCGTGCTTGA

NC_000962.3 282484 282502 TBseq_1.0_157_RIGHT 1 - TCATCGGAGAGCACGGCA

NC_000962.3 282265 282285 TBseq_1.0_158_LEFT 2 + CGACGTTGACGATGGACAGG

NC_000962.3 284325 284345 TBseq_1.0_158_RIGHT 2 - GCATCACCGATCTGGCCATC

NC_000962.3 284122 284142 TBseq_1.0_159_LEFT 1 + CTGAGATTGGCCGGTTCCTC

NC_000962.3 286093 286113 TBseq_1.0_159_RIGHT 1 - GAATCTTCCGGCGTGACCAC

NC_000962.3 285885 285904 TBseq_1.0_160_LEFT 2 + CACCCCGACCAACAGCATC

NC_000962.3 287878 287897 TBseq_1.0_160_RIGHT 2 - CGTTGTGCGAATCACCCGA

NC_000962.3 287674 287694 TBseq_1.0_161_LEFT 1 + CGGCAGATGAGAAAGCTGGG

NC_000962.3 289678 289697 TBseq_1.0_161_RIGHT 1 - ACACCATGATGGCGCAAGG

NC_000962.3 289453 289473 TBseq_1.0_162_LEFT 2 + CAGTCTGCGAACTCGTGCTT

NC_000962.3 291440 291460 TBseq_1.0_162_RIGHT 2 - CGGCCAAGTCTTCTCTGTCG

NC_000962.3 291149 291168 TBseq_1.0_163_LEFT 1 + TGACCAGGATGTCGGCCTT

NC_000962.3 293149 293169 TBseq_1.0_163_RIGHT 1 - CTGCAGGCTCAACCCGTTAC

NC_000962.3 292963 292983 TBseq_1.0_164_LEFT 2 + GCAATTCGACTCCGCTGACC

NC_000962.3 294990 295010 TBseq_1.0_164_RIGHT 2 - CATCTTCCACCTGGTGCTGG

NC_000962.3 294770 294789 TBseq_1.0_165_LEFT 1 + TTGTGTACCTCGCCGACCA

NC_000962.3 296771 296791 TBseq_1.0_165_RIGHT 1 - GAGACCTCAAGTGGGCATGC

NC_000962.3 296602 296622 TBseq_1.0_166_LEFT 2 + GCCCATGTTTGAGAGGCACA

NC_000962.3 298610 298630 TBseq_1.0_166_RIGHT 2 - CGACATTCGACGAGGACGAG

NC_000962.3 298473 298492 TBseq_1.0_167_LEFT 1 + ATTCGGTACTCGCTGGGCT

NC_000962.3 300451 300471 TBseq_1.0_167_RIGHT 1 - CGACGATGGCCGAATCAGTC

NC_000962.3 300256 300275 TBseq_1.0_168_LEFT 2 + CGCCTTGGTCCTTGAGCAG

NC_000962.3 302244 302263 TBseq_1.0_168_RIGHT 2 - CTATGACGCCGGTGTGCTG

NC_000962.3 302041 302061 TBseq_1.0_169_LEFT 1 + GGCTGGCGGTGTTACAGATC

NC_000962.3 303991 304011 TBseq_1.0_169_RIGHT 1 - GTCGGAGAGCACCAACTTGG

NC_000962.3 303773 303793 TBseq_1.0_170_LEFT 2 + GATAGACGGGCGGTGTTACG

NC_000962.3 305824 305844 TBseq_1.0_170_RIGHT 2 - GACGTGTGCTCAAGCTGTGA

NC_000962.3 305671 305691 TBseq_1.0_171_LEFT 1 + GTGCAATCGCCGATCCTGAA

NC_000962.3 307612 307632 TBseq_1.0_171_RIGHT 1 - CGATGAACCCGGTCTTGCTC

NC_000962.3 305925 305945 TBseq_1.0_172_LEFT 2 + CCGAAGAATGGGATGGCACC

NC_000962.3 307844 307862 TBseq_1.0_172_RIGHT 2 - ACGGATGTCGGGTCTGCT

NC_000962.3 307712 307732 TBseq_1.0_173_LEFT 1 + CGGCAGACCATCGAGTTGTT

NC_000962.3 309642 309662 TBseq_1.0_173_RIGHT 1 - GCTAACCGGTCATGACGCTC

NC_000962.3 309590 309609 TBseq_1.0_174_LEFT 2 + CCACGTTAAGGCCCAGCAG

NC_000962.3 311495 311515 TBseq_1.0_174_RIGHT 2 - GAACCTGATCTTGACGGCCC

NC_000962.3 311309 311330 TBseq_1.0_175_LEFT 1 + GATATCCGCGGGACAAGAACG

NC_000962.3 313298 313318 TBseq_1.0_175_RIGHT 1 - CGCTATTGGGATCGCTGTCC

NC_000962.3 313128 313147 TBseq_1.0_176_LEFT 2 + AAGCCGATGACGTAGCCGA

NC_000962.3 315126 315146 TBseq_1.0_176_RIGHT 2 - CGTAGGAATGCGGCTACAGG

NC_000962.3 314920 314940 TBseq_1.0_177_LEFT 1 + CAGTCGCACATATTGGCCGG

NC_000962.3 316936 316955 TBseq_1.0_177_RIGHT 1 - CGTCGACCAGAAGTTCGCC

NC_000962.3 316799 316819 TBseq_1.0_178_LEFT 2 + GCGATCACCAGACCCATGTC

NC_000962.3 318757 318777 TBseq_1.0_178_RIGHT 2 - CAGTGATCACCCCGGTGTTC

NC_000962.3 318621 318641 TBseq_1.0_179_LEFT 1 + GCCAGTTGTCGAACAGCACG

NC_000962.3 320553 320573 TBseq_1.0_179_RIGHT 1 - CCCGCTGATGAAACTGGTCC

NC_000962.3 320384 320404 TBseq_1.0_180_LEFT 2 + GGACCAGACAGAATCGCGTC

NC_000962.3 322308 322327 TBseq_1.0_180_RIGHT 2 - CCGCCAGCATGACGAAACT

NC_000962.3 322095 322115 TBseq_1.0_181_LEFT 1 + GCCGACACCTGGATCATGTC

NC_000962.3 324092 324111 TBseq_1.0_181_RIGHT 1 - GTGGACTTGGACCCGATGC

NC_000962.3 323873 323890 TBseq_1.0_182_LEFT 2 + AACGACTGGTTGCCGCG

NC_000962.3 325922 325941 TBseq_1.0_182_RIGHT 2 - CGCTCACATACAGCAGGCC

NC_000962.3 325745 325766 TBseq_1.0_183_LEFT 1 + GTCAAGGGGGTGACGGTTAAG

NC_000962.3 327700 327720 TBseq_1.0_183_RIGHT 1 - GGGCAATTCCAGGCGATCAA

NC_000962.3 327483 327503 TBseq_1.0_184_LEFT 2 + GTTCGTGTCATGCTCCCAGG

NC_000962.3 329456 329476 TBseq_1.0_184_RIGHT 2 - CCCGATTCCGTGGTTTCGAA

NC_000962.3 329292 329312 TBseq_1.0_185_LEFT 1 + AGGGGAGTCACGTTCGGATC

NC_000962.3 331250 331268 TBseq_1.0_185_RIGHT 1 - AAGCGTGCGGAATCCAGC

NC_000962.3 331035 331055 TBseq_1.0_186_LEFT 2 + GGACGGGACAAGTTTGGGTC

NC_000962.3 333069 333089 TBseq_1.0_186_RIGHT 2 - CCATCCGAATCACCGAACCG

NC_000962.3 331479 331499 TBseq_1.0_187_LEFT 1 + TCGTAGAAGTAGCGCACCGA

NC_000962.3 333403 333421 TBseq_1.0_187_RIGHT 1 - CATCATGCGCACCACCGT

NC_000962.3 339074 339095 TBseq_1.0_188_LEFT 2 + TGACACCTCCCAATACGCATG

NC_000962.3 340985 341004 TBseq_1.0_188_RIGHT 2 - TACGCACCCAATCGACCCT

NC_000962.3 339329 339352 TBseq_1.0_189_LEFT 1 + TCATGTTCATCAGTCCTCTGTGG

NC_000962.3 341361 341380 TBseq_1.0_189_RIGHT 1 - GGGCGGTCCAGCTCAAAAA

NC_000962.3 341169 341188 TBseq_1.0_190_LEFT 2 + GCAAGCTTCCCGACCACAA

NC_000962.3 343207 343227 TBseq_1.0_190_RIGHT 2 - GACCAGTTCCATGGCCACTG

NC_000962.3 342992 343012 TBseq_1.0_191_LEFT 1 + GTCGGATACCAGCTTCGGGA

NC_000962.3 344960 344980 TBseq_1.0_191_RIGHT 1 - GCGTAGTACTGCACGGTGTC

NC_000962.3 344819 344839 TBseq_1.0_192_LEFT 2 + TTCAACGCGATACCCGAAGC

NC_000962.3 346784 346804 TBseq_1.0_192_RIGHT 2 - GGCCTCATCAGCGCTGAATT

NC_000962.3 346547 346565 TBseq_1.0_193_LEFT 1 + GGCAGCCAACAGATGCGT

NC_000962.3 348537 348556 TBseq_1.0_193_RIGHT 1 - CCAGCTCGGCGTGGTAATG

NC_000962.3 347633 347653 TBseq_1.0_194_LEFT 2 + TGGAGGACGCCTACCACATC

NC_000962.3 349601 349622 TBseq_1.0_194_RIGHT 2 - ACTGACTCCCCTTCTGTTGGG

NC_000962.3 349444 349463 TBseq_1.0_195_LEFT 1 + ATGTTGGCAGGCAATCCGG

NC_000962.3 351476 351497 TBseq_1.0_195_RIGHT 1 - TCCACGTGATTCGGAATTCGG

NC_000962.3 351484 351504 TBseq_1.0_196_LEFT 2 + CGAATCACGTGGACCCGTAC

NC_000962.3 353380 353400 TBseq_1.0_196_RIGHT 2 - GATGTCTTCGACGATGCCCG

NC_000962.3 353193 353213 TBseq_1.0_197_LEFT 1 + CAACGCTTGGTGGTTCCCTC

NC_000962.3 355230 355249 TBseq_1.0_197_RIGHT 1 - GAATCACACGACGCGCTGA

NC_000962.3 355094 355114 TBseq_1.0_198_LEFT 2 + GTCTCCACGATCACCTGCCT

NC_000962.3 357027 357047 TBseq_1.0_198_RIGHT 2 - GACCGGCGGATGATCATCAC

NC_000962.3 356807 356827 TBseq_1.0_199_LEFT 1 + TGCACTGGCCTACTCCTACC

NC_000962.3 358842 358861 TBseq_1.0_199_RIGHT 1 - CGTCGTTCAGCAGCGGAAT

NC_000962.3 358630 358649 TBseq_1.0_200_LEFT 2 + TACCTTTTCGTGTGGGCGC

NC_000962.3 360597 360617 TBseq_1.0_200_RIGHT 2 - TCGAGCTGCCCGACTATCTG

NC_000962.3 359349 359368 TBseq_1.0_201_LEFT 1 + GCCAATGACGTCGCGGATT

NC_000962.3 361312 361331 TBseq_1.0_201_RIGHT 1 - GAACCTCCCGTCGACTCGT

NC_000962.3 361102 361122 TBseq_1.0_202_LEFT 2 + CAGTGCACGATCAGCAGGTT

NC_000962.3 363157 363175 TBseq_1.0_202_RIGHT 2 - GGCTGTAGTTGGCGCACA

NC_000962.3 363120 363142 TBseq_1.0_203_LEFT 1 + CCGAGTCTGTAGATACCGTGGT

NC_000962.3 365042 365061 TBseq_1.0_203_RIGHT 1 - GGATGTCCGATGGCAGCAG

NC_000962.3 364187 364206 TBseq_1.0_204_LEFT 2 + GAATGCGGGGAGATAGCGC

NC_000962.3 366124 366144 TBseq_1.0_204_RIGHT 2 - AGCTATGTCACCTGGGCCAG

NC_000962.3 375712 375732 TBseq_1.0_205_LEFT 1 + CGTCATACCCGTTCGTCAGC

NC_000962.3 377647 377667 TBseq_1.0_205_RIGHT 1 - GGTCGGATGATGCACGTTCA

NC_000962.3 377464 377483 TBseq_1.0_206_LEFT 2 + CGACTGCGCTGGTATCTGC

NC_000962.3 379435 379454 TBseq_1.0_206_RIGHT 2 - AGTCCATGAATTGCGGCGG

NC_000962.3 379297 379317 TBseq_1.0_207_LEFT 1 + CAGGAGATGCTGCAGATCGC

NC_000962.3 381196 381216 TBseq_1.0_207_RIGHT 1 - CATCGCCGTACCCGAATTGG

NC_000962.3 381060 381080 TBseq_1.0_208_LEFT 2 + CCGCGACTTACGTCACCTTG

NC_000962.3 383001 383020 TBseq_1.0_208_RIGHT 2 - CGTGTCCGGCATGAGGAAC

NC_000962.3 382784 382803 TBseq_1.0_209_LEFT 1 + AGGACAACACCGACCCGAA

NC_000962.3 384841 384861 TBseq_1.0_209_RIGHT 1 - AAACTCCGGACCCTTACCCG

NC_000962.3 384646 384666 TBseq_1.0_210_LEFT 2 + GAAAGCTACTCCGCCTGTGG

NC_000962.3 386681 386701 TBseq_1.0_210_RIGHT 2 - CCTCTTCATCGACGGGTTGC

NC_000962.3 386520 386538 TBseq_1.0_211_LEFT 1 + TAGGCTCAGGGCGCTTGT

NC_000962.3 388476 388496 TBseq_1.0_211_RIGHT 1 - GTGGATGTGGTCGTAGTGGC

NC_000962.3 388253 388273 TBseq_1.0_212_LEFT 2 + GAAATTCGCGAAATCGGCGG

NC_000962.3 390237 390257 TBseq_1.0_212_RIGHT 2 - TCGGATTCGGGTTTGAACGC

NC_000962.3 390053 390073 TBseq_1.0_213_LEFT 1 + CTGCTTGCCCAAGGACATCC

NC_000962.3 392014 392034 TBseq_1.0_213_RIGHT 1 - GATCAATCCCCATGCCCGAC

NC_000962.3 391843 391862 TBseq_1.0_214_LEFT 2 + GCCGAACTAACTGGCGACC

NC_000962.3 393796 393816 TBseq_1.0_214_RIGHT 2 - GCGAAGGCAACCACATCCTT

NC_000962.3 393583 393603 TBseq_1.0_215_LEFT 1 + GATGATGTCGAGGGTGAGCG

NC_000962.3 395610 395629 TBseq_1.0_215_RIGHT 1 - CCTCGCCGAGGTTTGGTTC

NC_000962.3 395412 395431 TBseq_1.0_216_LEFT 2 + ATCGATCACGCCAGGTTGC

NC_000962.3 397408 397428 TBseq_1.0_216_RIGHT 2 - CAGTCTGGTGTTTCAGGGGC

NC_000962.3 397227 397247 TBseq_1.0_217_LEFT 1 + GTGATCACCAGGCAGCCAAG

NC_000962.3 399160 399179 TBseq_1.0_217_RIGHT 1 - GAAATACAGGCCCGGCACC

NC_000962.3 398985 399005 TBseq_1.0_218_LEFT 2 + TCTTCTACGGCCCAGGTCTG

NC_000962.3 400997 401017 TBseq_1.0_218_RIGHT 2 - CCTCGGCGATCAGGTGAATC

NC_000962.3 400762 400782 TBseq_1.0_219_LEFT 1 + AAAGCCAAGACGGTGTGTGC

NC_000962.3 402759 402779 TBseq_1.0_219_RIGHT 1 - GGTCTCCGAGCTGATCACGA

NC_000962.3 402597 402616 TBseq_1.0_220_LEFT 2 + TGGATTCCAGGTCGGCGAT

NC_000962.3 404586 404605 TBseq_1.0_220_RIGHT 2 - GTCGATATGCGCCGCTACC

NC_000962.3 404391 404411 TBseq_1.0_221_LEFT 1 + CACAACCCACCCAGAACAGG

NC_000962.3 406408 406427 TBseq_1.0_221_RIGHT 1 - GGTCCTGGCCGAAAACGTC

NC_000962.3 406257 406277 TBseq_1.0_222_LEFT 2 + CCAGCTTAAGATCCCGTGCC

NC_000962.3 408165 408183 TBseq_1.0_222_RIGHT 2 - TCATCGACGACGCCCAGT

NC_000962.3 407978 407997 TBseq_1.0_223_LEFT 1 + GTGCAATCTCGCAGGTGCT

NC_000962.3 409973 409993 TBseq_1.0_223_RIGHT 1 - GCAACTCCGATCTGGCCATT

NC_000962.3 409831 409851 TBseq_1.0_224_LEFT 2 + TATTGGGACGGGTCTGGGTG

NC_000962.3 411786 411806 TBseq_1.0_224_RIGHT 2 - AGACACCGCCAATTGCTCTG

NC_000962.3 411558 411577 TBseq_1.0_225_LEFT 1 + GGCGGGAGATCGTCAATGC

NC_000962.3 413587 413607 TBseq_1.0_225_RIGHT 1 - AGCAGGGCCCTATTGAGCTC

NC_000962.3 413454 413473 TBseq_1.0_226_LEFT 2 + CACCCGCGAACTGAACCAG

NC_000962.3 415348 415368 TBseq_1.0_226_RIGHT 2 - CCACCACCTTGGCATTGACA

NC_000962.3 415186 415205 TBseq_1.0_227_LEFT 1 + TTTGGTGCTGGGTGCTGTC

NC_000962.3 417134 417156 TBseq_1.0_227_RIGHT 1 - ACCGCAATATCGACTGTCAACG

NC_000962.3 416977 416997 TBseq_1.0_228_LEFT 2 + GATGGTGGCTTCTTGGGACG

NC_000962.3 418980 419000 TBseq_1.0_228_RIGHT 2 - GGCGGGCAAGGTATATCGAC

NC_000962.3 418790 418810 TBseq_1.0_229_LEFT 1 + CTCGTATTGGCGCTGGCTAG

NC_000962.3 420759 420777 TBseq_1.0_229_RIGHT 1 - CACCCACGAGCACAACGT

NC_000962.3 420594 420614 TBseq_1.0_230_LEFT 2 + ACCTGCCCTACATCACCGTC

NC_000962.3 422603 422623 TBseq_1.0_230_RIGHT 2 - CCTCCGAAACCGCCTTGAAC

NC_000962.3 422326 422346 TBseq_1.0_231_LEFT 1 + GCAGATACCGCCGAAAACGA

NC_000962.3 424239 424261 TBseq_1.0_231_RIGHT 1 - CGTCTCCGGTTTCTACAACACG

NC_000962.3 422833 422852 TBseq_1.0_232_LEFT 2 + TGTTCGGTGGCTTGTTCGG

NC_000962.3 424750 424768 TBseq_1.0_232_RIGHT 2 - TCGAGGGCGACATCAACG

NC_000962.3 434686 434705 TBseq_1.0_233_LEFT 1 + CCCTAACCGGAACCGCTGA

NC_000962.3 436598 436618 TBseq_1.0_233_RIGHT 1 - TCGCATTGCATCTCATCCCG

NC_000962.3 436402 436421 TBseq_1.0_234_LEFT 2 + TGCCCATATAGCGCTCGGT

NC_000962.3 438433 438453 TBseq_1.0_234_RIGHT 2 - CGTATTCCCACCAGCCCAAC

NC_000962.3 438213 438233 TBseq_1.0_235_LEFT 1 + TGGCTCTTCGACCTGTCTGG

NC_000962.3 440235 440253 TBseq_1.0_235_RIGHT 1 - AAGCCACGCAGCACCATC

NC_000962.3 440048 440068 TBseq_1.0_236_LEFT 2 + CCAACGCCGTCGAGCTATTC

NC_000962.3 441992 442012 TBseq_1.0_236_RIGHT 2 - CTGCCCCAAGGACAAGTTGG

NC_000962.3 441805 441824 TBseq_1.0_237_LEFT 1 + ACGACGCCGATCTCGATCT

NC_000962.3 443745 443765 TBseq_1.0_237_RIGHT 1 - GACCAGTTCTTCAACGCCCC

NC_000962.3 443557 443576 TBseq_1.0_238_LEFT 2 + CCACCCCTTGGCAGTAGGT

NC_000962.3 445492 445511 TBseq_1.0_238_RIGHT 2 - GTGTTGGTGCACGCACTGA

NC_000962.3 445250 445276 TBseq_1.0_239_LEFT 1 + GTAGCGATCTGTTACAGCTTATAGCA

NC_000962.3 447323 447343 TBseq_1.0_239_RIGHT 1 - TCGATCTGGACAAGCCACCC

NC_000962.3 447187 447207 TBseq_1.0_240_LEFT 2 + GAATCTGCGTACGGTCGTCG

NC_000962.3 449126 449145 TBseq_1.0_240_RIGHT 2 - GACGTGCACTTCCCGGAAG

NC_000962.3 448993 449012 TBseq_1.0_241_LEFT 1 + AGTTCGATCAACGCGTCGG

NC_000962.3 450896 450915 TBseq_1.0_241_RIGHT 1 - AACCTGACGTCCACCGGAT

NC_000962.3 450680 450700 TBseq_1.0_242_LEFT 2 + CGGGCGCCTTGTTGGTATAC

NC_000962.3 452734 452754 TBseq_1.0_242_RIGHT 2 - CGGGTGAACGTGAGATAGCG

NC_000962.3 452521 452540 TBseq_1.0_243_LEFT 1 + ATTCACCGCGGTCAAACCC

NC_000962.3 454560 454579 TBseq_1.0_243_RIGHT 1 - GGCACCGGTCCTGGTAAAC

NC_000962.3 454390 454410 TBseq_1.0_244_LEFT 2 + ACGCACGCTTCCCAAAGTAG

NC_000962.3 456410 456428 TBseq_1.0_244_RIGHT 2 - AGGAAGGGCCCGGCATTA

NC_000962.3 456219 456239 TBseq_1.0_245_LEFT 1 + GCACGGTTTGCGGTTGATTC

NC_000962.3 458177 458197 TBseq_1.0_245_RIGHT 1 - GACTATTCGGTTGTCGGCGG

NC_000962.3 458004 458024 TBseq_1.0_246_LEFT 2 + CGGTGGTACTGGTGTCCTCT

NC_000962.3 459945 459964 TBseq_1.0_246_RIGHT 2 - AGATCGAGAAGGCGCACCC

NC_000962.3 459718 459738 TBseq_1.0_247_LEFT 1 + CCAGCTCTTCGGGGTTGAGA

NC_000962.3 461723 461742 TBseq_1.0_247_RIGHT 1 - GTTAGCGGCGATCACCACC

NC_000962.3 461508 461528 TBseq_1.0_248_LEFT 2 + GAGTACTTCTGCAGCGCCTG

NC_000962.3 463481 463501 TBseq_1.0_248_RIGHT 2 - TGTCGTCTGGATGGGTCTCC

NC_000962.3 463335 463355 TBseq_1.0_249_LEFT 1 + TCGCTTCGACATGGCATACG

NC_000962.3 465280 465299 TBseq_1.0_249_RIGHT 1 - AAGATGTCGAGCAGGGCCT

NC_000962.3 465078 465097 TBseq_1.0_250_LEFT 2 + AACCTACGTGCCGCCTTTG

NC_000962.3 467074 467094 TBseq_1.0_250_RIGHT 2 - GCTAACATCGGCGGACTCTC

NC_000962.3 466708 466725 TBseq_1.0_251_LEFT 1 + ATGGTCGCTCCGGGGAT

NC_000962.3 468765 468784 TBseq_1.0_251_RIGHT 1 - GAACCAGAACGGGGCTGTG

NC_000962.3 468630 468651 TBseq_1.0_252_LEFT 2 + GTCTGTGGATGCTCTCGATGC

NC_000962.3 470524 470544 TBseq_1.0_252_RIGHT 2 - CAGCGATATCCACCAGCGAC

NC_000962.3 470387 470406 TBseq_1.0_253_LEFT 1 + GTGGTGTGCAGCGAGATCC

NC_000962.3 472357 472376 TBseq_1.0_253_RIGHT 1 - GGTCAACGCGATCGACCTG

NC_000962.3 472216 472236 TBseq_1.0_254_LEFT 2 + GATGGTCTTCATTCCGGGCG

NC_000962.3 474175 474195 TBseq_1.0_254_RIGHT 2 - TCCTCATCCTCCCCTCTGGA

NC_000962.3 473795 473814 TBseq_1.0_255_LEFT 1 + GCACGGTGATTTGGACGCT

NC_000962.3 475802 475822 TBseq_1.0_255_RIGHT 1 - CCAGCAAATGATGGCACCGA

NC_000962.3 475616 475636 TBseq_1.0_256_LEFT 2 + GAACCGATCGACACGACCTG

NC_000962.3 477561 477582 TBseq_1.0_256_RIGHT 2 - GAACGACGGCTACAGCTATGG

NC_000962.3 477406 477424 TBseq_1.0_257_LEFT 1 + TGCGGTAGTTGCCCTGGT

NC_000962.3 479334 479352 TBseq_1.0_257_RIGHT 1 - TGACCTGCTCGGCTGCTT

NC_000962.3 479108 479128 TBseq_1.0_258_LEFT 2 + CGACAGCTTGTGACCGATGG

NC_000962.3 481127 481147 TBseq_1.0_258_RIGHT 2 - CTTCCTATCCCCCGATGGCA

NC_000962.3 480936 480957 TBseq_1.0_259_LEFT 1 + CATGACGTCATACAGGGTGCC

NC_000962.3 482974 482993 TBseq_1.0_259_RIGHT 1 - CAATGGTGCTGCTGGAGGG

NC_000962.3 482800 482820 TBseq_1.0_260_LEFT 2 + CCAGTGAGGTTCAGCTGGAC

NC_000962.3 484814 484834 TBseq_1.0_260_RIGHT 2 - CGCCATGTCCTCTTCGGAAG

NC_000962.3 484618 484637 TBseq_1.0_261_LEFT 1 + TCGTGGTTGCCGCTTTACC

NC_000962.3 486646 486666 TBseq_1.0_261_RIGHT 1 - GATGCCCGCTTCCCTAAACC

NC_000962.3 486500 486520 TBseq_1.0_262_LEFT 2 + TCAGGGTCCGTCCATTGTCA

NC_000962.3 488403 488423 TBseq_1.0_262_RIGHT 2 - GGCCCGACTTCGATGAAACG

NC_000962.3 488242 488262 TBseq_1.0_263_LEFT 1 + CCGAACTGTCTGTGGGCTTG

NC_000962.3 490176 490196 TBseq_1.0_263_RIGHT 1 - GGGTCAACCGATGGCGATTT

NC_000962.3 490014 490034 TBseq_1.0_264_LEFT 2 + CCAGGCTGCGGATACTGTTC

NC_000962.3 491990 492010 TBseq_1.0_264_RIGHT 2 - CTGGTAGGTCACGCCAACAC

NC_000962.3 491817 491836 TBseq_1.0_265_LEFT 1 + ACCAGAATCGCAGACGGGT

NC_000962.3 493774 493794 TBseq_1.0_265_RIGHT 1 - AGTGCACCCCGAGATAGGTC

NC_000962.3 493568 493587 TBseq_1.0_266_LEFT 2 + CGGTCGAGGGTCCCATTCA

NC_000962.3 495619 495639 TBseq_1.0_266_RIGHT 2 - TCACCGAGGTGCTGGATACC

NC_000962.3 495399 495419 TBseq_1.0_267_LEFT 1 + CCGTGGTGAAATGCCGAGAA

NC_000962.3 497423 497443 TBseq_1.0_267_RIGHT 1 - ATTGGTCCGGTTCGTCAACG

NC_000962.3 497287 497306 TBseq_1.0_268_LEFT 2 + ACGTTCGGTCTCTGACGCT

NC_000962.3 499270 499290 TBseq_1.0_268_RIGHT 2 - CGAGCAGGCCATTACCGATG

NC_000962.3 499113 499133 TBseq_1.0_269_LEFT 1 + AGATATGACGACCCGACCGG

NC_000962.3 501037 501057 TBseq_1.0_269_RIGHT 1 - TGTCTACAGGGTTCCACGCT

NC_000962.3 500885 500904 TBseq_1.0_270_LEFT 2 + CCTTGTCGCGCAACTGGAT

NC_000962.3 502852 502871 TBseq_1.0_270_RIGHT 2 - TTGTGCGGGTTGGCGATAC

NC_000962.3 502703 502723 TBseq_1.0_271_LEFT 1 + CGAATTAGTCCGGGCTGCAG

NC_000962.3 504647 504668 TBseq_1.0_271_RIGHT 1 - GGCCATCTTCAGATAGGCGAC

NC_000962.3 504497 504517 TBseq_1.0_272_LEFT 2 + CTCAAAGGCATCGCGCTGTA

NC_000962.3 506403 506423 TBseq_1.0_272_RIGHT 2 - CGCGGTAAGTACGAGTTGCT

NC_000962.3 506254 506275 TBseq_1.0_273_LEFT 1 + GTTGTCGATTGTGATGTCGCG

NC_000962.3 508230 508249 TBseq_1.0_273_RIGHT 1 - CTCGTCGTCGACCCGGTAT

NC_000962.3 507968 507988 TBseq_1.0_274_LEFT 2 + CAGCTGACGCCGTCATAGAG

NC_000962.3 509992 510011 TBseq_1.0_274_RIGHT 2 - ATAGGCCCGGAGTGGTTCG

NC_000962.3 509772 509790 TBseq_1.0_275_LEFT 1 + CGCCGAGTTGCCGATGTT

NC_000962.3 511801 511820 TBseq_1.0_275_RIGHT 1 - GAGGAGCTCGGCATGCAAG

NC_000962.3 511518 511535 TBseq_1.0_276_LEFT 2 + ATCCGGCCGTCGACCAA

NC_000962.3 513568 513588 TBseq_1.0_276_RIGHT 2 - TTTGGGAGCCCTGTTGATGC

NC_000962.3 513407 513426 TBseq_1.0_277_LEFT 1 + TTTGCGGACTTGCTCGACC

NC_000962.3 515367 515387 TBseq_1.0_277_RIGHT 1 - CGCAGCACCAACTAGTCTGG

NC_000962.3 515193 515213 TBseq_1.0_278_LEFT 2 + GCGACCTTCATACCGGTTCC

NC_000962.3 517104 517123 TBseq_1.0_278_RIGHT 2 - GGTGGGTCTGTCGGCAATG

NC_000962.3 516896 516916 TBseq_1.0_279_LEFT 1 + AGAAGGTATCCCAGCCGACC

NC_000962.3 518911 518930 TBseq_1.0_279_RIGHT 1 - ATCCACCAGCGCATTGAGC

NC_000962.3 518716 518736 TBseq_1.0_280_LEFT 2 + CCCAGGGGAAAGCGATATGG

NC_000962.3 520724 520744 TBseq_1.0_280_RIGHT 2 - GCACGTGTACACCCCAGATC

NC_000962.3 520540 520560 TBseq_1.0_281_LEFT 1 + CCGAGGCAATGCAGGATCTG

NC_000962.3 522527 522548 TBseq_1.0_281_RIGHT 1 - CGGACTCGACGGTTATAGTGC

NC_000962.3 522362 522382 TBseq_1.0_282_LEFT 2 + CCCTTGGTGCCGAACTTACG

NC_000962.3 524291 524311 TBseq_1.0_282_RIGHT 2 - GTCACACTGTCCAATGCGGG

NC_000962.3 524147 524166 TBseq_1.0_283_LEFT 1 + ACGGTCATCACCTTGCCGA

NC_000962.3 526138 526159 TBseq_1.0_283_RIGHT 1 - TCTGGGACTTGACCTAACCGG

NC_000962.3 525776 525796 TBseq_1.0_284_LEFT 2 + CGTGTCACCCATGCTGAGTT

NC_000962.3 527794 527814 TBseq_1.0_284_RIGHT 2 - CTTCGACGACTTGCAGTGGG

NC_000962.3 527644 527664 TBseq_1.0_285_LEFT 1 + GAGTCAGCTCGGTGTTGGAG

NC_000962.3 529571 529591 TBseq_1.0_285_RIGHT 1 - GGTCTCGTCCTTGGTGACCA

NC_000962.3 528761 528781 TBseq_1.0_286_LEFT 2 + GTGTGTCCATCGCCAAGGAG

NC_000962.3 530730 530749 TBseq_1.0_286_RIGHT 2 - ACATCGGGCGCCTATCCTC

NC_000962.3 530520 530539 TBseq_1.0_287_LEFT 1 + CAGGTTTTGCCGCGTACGA

NC_000962.3 532515 532533 TBseq_1.0_287_RIGHT 1 - AATGCTGTTGGCGCTGGG

NC_000962.3 532368 532386 TBseq_1.0_288_LEFT 2 + TGCCACCTGCCCCGTAAT

NC_000962.3 534269 534290 TBseq_1.0_288_RIGHT 2 - GGGTGTATGGACTGGTGATGC

NC_000962.3 534077 534096 TBseq_1.0_289_LEFT 1 + CACCATAGCGCACTTCCCG

NC_000962.3 536089 536109 TBseq_1.0_289_RIGHT 1 - TCGACCTAGTTGGCCAGACC

NC_000962.3 535856 535875 TBseq_1.0_290_LEFT 2 + GGTGCCGATCTCGAGAACG

NC_000962.3 537861 537881 TBseq_1.0_290_RIGHT 2 - GAGGTTCCGCCAACTACGTG

NC_000962.3 537636 537656 TBseq_1.0_291_LEFT 1 + GGCGCTGTTGGTGGAGTATG

NC_000962.3 539675 539695 TBseq_1.0_291_RIGHT 1 - CGATGCACTGGACGGAATCG

NC_000962.3 539525 539544 TBseq_1.0_292_LEFT 2 + CGGTCATGGTGCTGTGCAT

NC_000962.3 541448 541468 TBseq_1.0_292_RIGHT 2 - CTCCAATACCAACGCTCGCC

NC_000962.3 541155 541175 TBseq_1.0_293_LEFT 1 + CAATTGAGCAACCAGGCCGT

NC_000962.3 543136 543154 TBseq_1.0_293_RIGHT 1 - AAGTCGCCCCTGACGCAT

NC_000962.3 542908 542929 TBseq_1.0_294_LEFT 2 + ACCAAACTCACGCTTCTTGGG

NC_000962.3 544911 544931 TBseq_1.0_294_RIGHT 2 - CGAATGTAACGACCCCGACC

NC_000962.3 544756 544776 TBseq_1.0_295_LEFT 1 + CATGATGTCGGTGGGTTCGC

NC_000962.3 546674 546694 TBseq_1.0_295_RIGHT 1 - ACGAGATCGAGGCCGCTATC

NC_000962.3 546526 546545 TBseq_1.0_296_LEFT 2 + AATCCTTGCCCGGATCCCA

NC_000962.3 548443 548463 TBseq_1.0_296_RIGHT 2 - CCAGTGGATTCGATACGCCG

NC_000962.3 548244 548264 TBseq_1.0_297_LEFT 1 + CGAATCCACCGTAGCCGTTC

NC_000962.3 550265 550286 TBseq_1.0_297_RIGHT 1 - CAGCGACATCAGGTAGAGCAC

NC_000962.3 550032 550052 TBseq_1.0_298_LEFT 2 + CGTTGGCGGTCGATCACTTC

NC_000962.3 551981 552001 TBseq_1.0_298_RIGHT 2 - CCGGTAATCGTTGTCAGCCA

NC_000962.3 551820 551840 TBseq_1.0_299_LEFT 1 + CCGGACATCGTCATCGCAAT

NC_000962.3 553733 553752 TBseq_1.0_299_RIGHT 1 - GTGAACGGGAACTTGGCCA

NC_000962.3 553508 553528 TBseq_1.0_300_LEFT 2 + CCAACGTGGGCCACATCTAC

NC_000962.3 555563 555583 TBseq_1.0_300_RIGHT 2 - CTCATCGACGCCATGGTCAC

NC_000962.3 555366 555386 TBseq_1.0_301_LEFT 1 + GTTGTAGTGTCGACAGCCGG

NC_000962.3 557395 557413 TBseq_1.0_301_RIGHT 1 - TCAACGCTGGTCCACCCA

NC_000962.3 557242 557262 TBseq_1.0_302_LEFT 2 + CAATCTGTGACCGGATCCGC

NC_000962.3 559155 559174 TBseq_1.0_302_RIGHT 2 - CGACAACGGCCTCGATCAC

NC_000962.3 558892 558912 TBseq_1.0_303_LEFT 1 + CAGTGAGCGATGCGATCCAG

NC_000962.3 560878 560898 TBseq_1.0_303_RIGHT 1 - CTGTTCCGCCAGGGTTACAC

NC_000962.3 560706 560725 TBseq_1.0_304_LEFT 2 + AAGGGCATCAGCAACGTGG

NC_000962.3 562651 562669 TBseq_1.0_304_RIGHT 2 - CGCACTTCGCACCACCAA

NC_000962.3 562490 562510 TBseq_1.0_305_LEFT 1 + GTGATTCCCACCACAGGACG

NC_000962.3 564425 564444 TBseq_1.0_305_RIGHT 1 - AACTCGTGATCGGGGTTGC

NC_000962.3 564275 564293 TBseq_1.0_306_LEFT 2 + ATCGGGCGTTCCAGTGGT

NC_000962.3 566187 566206 TBseq_1.0_306_RIGHT 2 - AACGCCTCCTGGGTCAACT

NC_000962.3 565966 565988 TBseq_1.0_307_LEFT 1 + GACCAAGCTGCAGGAAGATCTG

NC_000962.3 568002 568021 TBseq_1.0_307_RIGHT 1 - ACAGCGTGCAAGTCACCTC

NC_000962.3 567827 567844 TBseq_1.0_308_LEFT 2 + ATGCTCAACGCCGGTGC

NC_000962.3 569853 569870 TBseq_1.0_308_RIGHT 2 - TGGATTGCCCGGCTCCT

NC_000962.3 569682 569702 TBseq_1.0_309_LEFT 1 + TCGCCTCAGGAAACACCACC

NC_000962.3 571624 571643 TBseq_1.0_309_RIGHT 1 - ACATGCAGCCGATCAGCAC

NC_000962.3 571457 571476 TBseq_1.0_310_LEFT 2 + GCAAGGGCTATCCGGATGC

NC_000962.3 573424 573444 TBseq_1.0_310_RIGHT 2 - CTGCCCAAGCTGATCGACTC

NC_000962.3 573182 573202 TBseq_1.0_311_LEFT 1 + GTGTCATGCCGGCATAAACC

NC_000962.3 575212 575233 TBseq_1.0_311_RIGHT 1 - CCCAATGGATCGGCATAGAGC

NC_000962.3 575077 575097 TBseq_1.0_312_LEFT 2 + CGAATATCCCGAGGCGATGG

NC_000962.3 576975 576994 TBseq_1.0_312_RIGHT 2 - CAGGCTTGCGACACACGAA

NC_000962.3 576818 576838 TBseq_1.0_313_LEFT 1 + GATCCAGAATGCGCTCGAGG

NC_000962.3 578807 578825 TBseq_1.0_313_RIGHT 1 - TGAACTGACTGCCCCGCT

NC_000962.3 578590 578610 TBseq_1.0_314_LEFT 2 + CTCTACACCTCGTTGCTGCG

NC_000962.3 580559 580578 TBseq_1.0_314_RIGHT 2 - TCGGCTCAGCTCTTCCTCT

NC_000962.3 580406 580425 TBseq_1.0_315_LEFT 1 + GGCTAATCACGACGGCACC

NC_000962.3 582410 582428 TBseq_1.0_315_RIGHT 1 - CAGCAACGCCAAGTTCGG

NC_000962.3 582213 582232 TBseq_1.0_316_LEFT 2 + GCAACAGTCCTGGCGTCTC

NC_000962.3 584120 584139 TBseq_1.0_316_RIGHT 2 - CAAGCGGTGGGGATGGATC

NC_000962.3 583980 584000 TBseq_1.0_317_LEFT 1 + GACGGTAAAGGACTTGCGGG

NC_000962.3 585877 585896 TBseq_1.0_317_RIGHT 1 - ACCTGGAACTCGACGAGCA

NC_000962.3 585648 585668 TBseq_1.0_318_LEFT 2 + GGTGAGGGTGGTCTTGAGGA

NC_000962.3 587671 587691 TBseq_1.0_318_RIGHT 2 - GGTTCTCGTTGGCTCTTCGG

NC_000962.3 587536 587557 TBseq_1.0_319_LEFT 1 + TTCCGATCATTCGTGACGACC

NC_000962.3 589467 589486 TBseq_1.0_319_RIGHT 1 - CATCGGCCACGCTAATCCG

NC_000962.3 589269 589290 TBseq_1.0_320_LEFT 2 + CTGAACGAGCACTGGACCATC

NC_000962.3 591300 591318 TBseq_1.0_320_RIGHT 2 - ACATGTCGTGGACGGCCT

NC_000962.3 591091 591111 TBseq_1.0_321_LEFT 1 + CTGACACGATTGGGTTGCGA

NC_000962.3 593058 593078 TBseq_1.0_321_RIGHT 1 - CAGGCGCTGTCGGAGAAATC

NC_000962.3 592837 592857 TBseq_1.0_322_LEFT 2 + CACAAATCGGAGCCGGGTAG

NC_000962.3 594871 594891 TBseq_1.0_322_RIGHT 2 - GTGGTCACCAGTGTCGTCAC

NC_000962.3 594660 594681 TBseq_1.0_323_LEFT 1 + GTCATCGATGGGTCAAGCCAG

NC_000962.3 596701 596721 TBseq_1.0_323_RIGHT 1 - TAGGCCTCCCGATACCTCCT

NC_000962.3 596559 596579 TBseq_1.0_324_LEFT 2 + GTTCGGGGCCAACATGTCAG

NC_000962.3 598496 598516 TBseq_1.0_324_RIGHT 2 - CGATCAACAGCACCTCAGGG

NC_000962.3 598274 598293 TBseq_1.0_325_LEFT 1 + CAAGCGGCGGATGAACACT

NC_000962.3 600302 600322 TBseq_1.0_325_RIGHT 1 - GTCGACCTCCCAGTAGCTGT

NC_000962.3 600106 600126 TBseq_1.0_326_LEFT 2 + CCCCAGGTGGAGCTTTTGAC

NC_000962.3 602101 602120 TBseq_1.0_326_RIGHT 2 - CGCAACCGTGAACCTTGGA

NC_000962.3 601943 601963 TBseq_1.0_327_LEFT 1 + GCGGAGTTGGTGACCATCAG

NC_000962.3 603918 603937 TBseq_1.0_327_RIGHT 1 - CAGACCGAACTCGCCGAAC

NC_000962.3 603665 603687 TBseq_1.0_328_LEFT 2 + CAAAGCTGAACTGGTGGGAGAG

NC_000962.3 605729 605749 TBseq_1.0_328_RIGHT 2 - GGCGTTATCAACCCAACCCC

NC_000962.3 605595 605615 TBseq_1.0_329_LEFT 1 + GGCCTGACATGACACCAACC

NC_000962.3 607522 607540 TBseq_1.0_329_RIGHT 1 - CGCTTTCGACGGCGCATA

NC_000962.3 607304 607321 TBseq_1.0_330_LEFT 2 + GTGGGCGCGCTGATTGT

NC_000962.3 609316 609336 TBseq_1.0_330_RIGHT 2 - GAGCAGACCGAACAGCAAGT

NC_000962.3 609139 609160 TBseq_1.0_331_LEFT 1 + CCGTGTGGGTCATCTTTACGC

NC_000962.3 611120 611140 TBseq_1.0_331_RIGHT 1 - ACAGCTGAATTCGGACGGAC

NC_000962.3 610924 610944 TBseq_1.0_332_LEFT 2 + GGTCGACGTGCTAGTCCTCT

NC_000962.3 612893 612913 TBseq_1.0_332_RIGHT 2 - GTCCGTGGGGTCAACGATAC

NC_000962.3 612710 612730 TBseq_1.0_333_LEFT 1 + CGAACTCGAGGGGAAGTCCT

NC_000962.3 614687 614707 TBseq_1.0_333_RIGHT 1 - GTGGCTGGCTCGGTTCAATC

NC_000962.3 614487 614507 TBseq_1.0_334_LEFT 2 + CGCGGTGATGTTCTTCAGCC

NC_000962.3 616451 616471 TBseq_1.0_334_RIGHT 2 - CAGATCCGGGTCTGTCTCCA

NC_000962.3 616221 616241 TBseq_1.0_335_LEFT 1 + GATGCCCGAGGAAACCCAAG

NC_000962.3 618263 618282 TBseq_1.0_335_RIGHT 1 - CCCTGCCCACTCAAATCGG

NC_000962.3 618067 618087 TBseq_1.0_336_LEFT 2 + GGGGATTCCGTTCGTGCTTT

NC_000962.3 619993 620010 TBseq_1.0_336_RIGHT 2 - GCGAACCTGGGCGAACT

NC_000962.3 619830 619847 TBseq_1.0_337_LEFT 1 + TTTTGAGGCGCGGTCCC

NC_000962.3 621761 621781 TBseq_1.0_337_RIGHT 1 - ACCAGTACGTCGGACCTAGG

NC_000962.3 620785 620805 TBseq_1.0_338_LEFT 2 + GCTTCGTGGCCATGGTCTTC

NC_000962.3 622755 622772 TBseq_1.0_338_RIGHT 2 - ACACCCGGTGCAGCCAA

NC_000962.3 622514 622534 TBseq_1.0_339_LEFT 1 + GGCATTTGGGTGTTCAGCCC

NC_000962.3 624583 624602 TBseq_1.0_339_RIGHT 1 - CTCCATTCCGCTCGCGATG

NC_000962.3 624609 624628 TBseq_1.0_340_LEFT 2 + AGGTGTTTCCGGTGTGCTC

NC_000962.3 626527 626547 TBseq_1.0_340_RIGHT 2 - TGTCCGAATTGACTGTGCGG

NC_000962.3 626299 626318 TBseq_1.0_341_LEFT 1 + AACAGCCAGTGCCAACAGC

NC_000962.3 628321 628341 TBseq_1.0_341_RIGHT 1 - CAGTGCGGCCGAAGATCTAC

NC_000962.3 628181 628200 TBseq_1.0_342_LEFT 2 + GCCACATTGTCGCCGATCC

NC_000962.3 630164 630184 TBseq_1.0_342_RIGHT 2 - CCACCACGGTCTCGGTTAAC

NC_000962.3 629980 630000 TBseq_1.0_343_LEFT 1 + CAGTGTAACGATGCCGGGAC

NC_000962.3 631975 631994 TBseq_1.0_343_RIGHT 1 - CAAGTCGCCGGCATCCATC

NC_000962.3 631748 631768 TBseq_1.0_344_LEFT 2 + CCCTGTCTCAACGAGGAGGA

NC_000962.3 633781 633798 TBseq_1.0_344_RIGHT 2 - GTGCTGGCCAGTGAGCA

NC_000962.3 633582 633603 TBseq_1.0_345_LEFT 1 + GTTGAGAAAGACACCCCAGCC

NC_000962.3 635567 635587 TBseq_1.0_345_RIGHT 1 - GAGGAGCACGCATAGCCATC

NC_000962.3 635376 635396 TBseq_1.0_346_LEFT 2 + GTGGTTCCTGACGTCGTCAC

NC_000962.3 637394 637414 TBseq_1.0_346_RIGHT 2 - CGTTCGACTTCACCAACGGG

NC_000962.3 637165 637185 TBseq_1.0_347_LEFT 1 + CCAGCAACCAGGTCAACAGG

NC_000962.3 639164 639183 TBseq_1.0_347_RIGHT 1 - ACGGATGCTGAAGTTCGCG

NC_000962.3 638934 638955 TBseq_1.0_348_LEFT 2 + TGCCGAGCCTGTAGTTACTGT

NC_000962.3 640968 640988 TBseq_1.0_348_RIGHT 2 - CAGATATGCCGGCGATGCTC

NC_000962.3 640711 640731 TBseq_1.0_349_LEFT 1 + GTTCCAACCCCTCAAGCCAC

NC_000962.3 642689 642709 TBseq_1.0_349_RIGHT 1 - CGTGACCAGGATTGTTCGGG

NC_000962.3 642480 642500 TBseq_1.0_350_LEFT 2 + CTTCAGGACGGTTGAGCGAC

NC_000962.3 644523 644542 TBseq_1.0_350_RIGHT 2 - ACCACATACAGGCGGTCCA

NC_000962.3 644330 644350 TBseq_1.0_351_LEFT 1 + CTGGCAACTGTTCGCTGAGG

NC_000962.3 646289 646310 TBseq_1.0_351_RIGHT 1 - TCGAGGGGTTCACCAGTACAG

NC_000962.3 646069 646089 TBseq_1.0_352_LEFT 2 + CCGGTGCTGGTGATTGGTTT

NC_000962.3 648046 648066 TBseq_1.0_352_RIGHT 2 - CAGGACGGCGATTCGTAACC

NC_000962.3 647790 647808 TBseq_1.0_353_LEFT 1 + CGTCGATGTGGGCGCATT

NC_000962.3 649768 649789 TBseq_1.0_353_RIGHT 1 - CCCAGGGACAACACGGTATTG

NC_000962.3 649535 649552 TBseq_1.0_354_LEFT 2 + ACCTGGTGCACGAACGC

NC_000962.3 651563 651581 TBseq_1.0_354_RIGHT 2 - GCGGTGGCGGTCAATTGT

NC_000962.3 651415 651435 TBseq_1.0_355_LEFT 1 + CGGTAGGCGGATTCGAACTC

NC_000962.3 653354 653373 TBseq_1.0_355_RIGHT 1 - GGTGGCACCGGAGAACATC

NC_000962.3 653148 653168 TBseq_1.0_356_LEFT 2 + TACCTACTGGCCACCGCATC

NC_000962.3 655188 655208 TBseq_1.0_356_RIGHT 2 - GACCTGATCGTCACCTGCAC

NC_000962.3 655006 655024 TBseq_1.0_357_LEFT 1 + TGATGACGGCGTCGACCT

NC_000962.3 656943 656963 TBseq_1.0_357_RIGHT 1 - CAGCACTGGCCAGAGGATCT

NC_000962.3 656759 656779 TBseq_1.0_358_LEFT 2 + AGTTTTGGGAGGCAACGCTT

NC_000962.3 658760 658780 TBseq_1.0_358_RIGHT 2 - CGTCCATAGCCGCCATGAAC

NC_000962.3 658565 658585 TBseq_1.0_359_LEFT 1 + GGAGACAGCGCACTTTCTGG

NC_000962.3 660528 660548 TBseq_1.0_359_RIGHT 1 - GATCAGGGTCGAACCGTTCC

NC_000962.3 660255 660274 TBseq_1.0_360_LEFT 2 + ACTGTCGTGGGCACTGGAG

NC_000962.3 662311 662328 TBseq_1.0_360_RIGHT 2 - GCGGCTTTGCAGATGGC

NC_000962.3 662121 662141 TBseq_1.0_361_LEFT 1 + GTCGCACCCGGATATCTGTG

NC_000962.3 664114 664133 TBseq_1.0_361_RIGHT 1 - GCGATGAGGAGGTCCAGGT

NC_000962.3 663893 663914 TBseq_1.0_362_LEFT 2 + CGAGCAGCTCAATGTCGAAGA

NC_000962.3 665963 665983 TBseq_1.0_362_RIGHT 2 - GGACACCGGCTACTCGTATC

NC_000962.3 665788 665808 TBseq_1.0_363_LEFT 1 + GGGTAACGGCAAGCTTTCGA

NC_000962.3 667740 667757 TBseq_1.0_363_RIGHT 1 - ATGTGGCGGCACAGGTG

NC_000962.3 667588 667608 TBseq_1.0_364_LEFT 2 + GGATGGTGCGAGGAATGTCC

NC_000962.3 669534 669555 TBseq_1.0_364_RIGHT 2 - CGGGTTACCACATGGAACACG

NC_000962.3 669323 669343 TBseq_1.0_365_LEFT 1 + CTTTCGAAGGTGAGCCGGAC

NC_000962.3 671311 671330 TBseq_1.0_365_RIGHT 1 - GTTCAGCGTGGCCATGGAA

NC_000962.3 669983 670003 TBseq_1.0_366_LEFT 2 + CCGAAGCGGGATTGGTTACC

NC_000962.3 671940 671960 TBseq_1.0_366_RIGHT 2 - GGATGCTCCCTATTGCTGCG

NC_000962.3 675951 675971 TBseq_1.0_367_LEFT 1 + GGCGTGGCAAGGAATTGTGT

NC_000962.3 677916 677936 TBseq_1.0_367_RIGHT 1 - CGTGTCGATGATCACCGCTC

NC_000962.3 677722 677741 TBseq_1.0_368_LEFT 2 + CGGTCTACCTGCCGGATGA

NC_000962.3 679763 679781 TBseq_1.0_368_RIGHT 2 - CGGAGCGCACGTGAAACA

NC_000962.3 679566 679585 TBseq_1.0_369_LEFT 1 + GATGTTGCCCACGACCACC

NC_000962.3 681556 681577 TBseq_1.0_369_RIGHT 1 - GATGGGCCGGGCGATATTTAC

NC_000962.3 681379 681399 TBseq_1.0_370_LEFT 2 + TACATCGACGGCCTGACCAT

NC_000962.3 683318 683338 TBseq_1.0_370_RIGHT 2 - CCTTCGGCGATCTAGGCATG

NC_000962.3 683134 683154 TBseq_1.0_371_LEFT 1 + TCTCGGCACTACACAGGTCC

NC_000962.3 685135 685154 TBseq_1.0_371_RIGHT 1 - GTGATGATCACGGCGTGGG

NC_000962.3 684949 684968 TBseq_1.0_372_LEFT 2 + ACGCTAGCTGCTCTGACCA

NC_000962.3 686979 686999 TBseq_1.0_372_RIGHT 2 - CCAGCTTCCATAACCAGCCC

NC_000962.3 686824 686844 TBseq_1.0_373_LEFT 1 + CAACGCTGGTGACGAGGAAG

NC_000962.3 688816 688833 TBseq_1.0_373_RIGHT 1 - AGGTGCCCGAAGCTGCT

NC_000962.3 688598 688618 TBseq_1.0_374_LEFT 2 + CGGGACCATGCGATAGGTGA

NC_000962.3 690570 690589 TBseq_1.0_374_RIGHT 2 - GCCAAGACCACCACCAACC

NC_000962.3 690362 690381 TBseq_1.0_375_LEFT 1 + CATCCTAAGTGCTGGGCGG

NC_000962.3 692326 692345 TBseq_1.0_375_RIGHT 1 - TCAGGGTCGCGATCCAGTT

NC_000962.3 692138 692158 TBseq_1.0_376_LEFT 2 + CCCCGGTGGTTTTGACTTCG

NC_000962.3 694095 694113 TBseq_1.0_376_RIGHT 2 - CGGACTGCGGCAAGAACA

NC_000962.3 693896 693914 TBseq_1.0_377_LEFT 1 + AACCTCAACACGCTGGCC

NC_000962.3 695862 695883 TBseq_1.0_377_RIGHT 1 - GATCTGGTGCTCGAACATCCC

NC_000962.3 695657 695677 TBseq_1.0_378_LEFT 2 + GTTCGAAGAGTTAGCCGGCC

NC_000962.3 697663 697683 TBseq_1.0_378_RIGHT 2 - CTGATTCCCACCGGACGAAC

NC_000962.3 697517 697535 TBseq_1.0_379_LEFT 1 + ACCAGCAACGGAATCGCG

NC_000962.3 699420 699441 TBseq_1.0_379_RIGHT 1 - ACCTCACCAAACCGTTCAACC

NC_000962.3 699129 699149 TBseq_1.0_380_LEFT 2 + CGATCTTGTCGCGCAGGTAG

NC_000962.3 701108 701127 TBseq_1.0_380_RIGHT 2 - TCTCGGCCCAGAAGTGCAT

NC_000962.3 700945 700964 TBseq_1.0_381_LEFT 1 + CCTCAACTTCCAGCCCACC

NC_000962.3 702882 702902 TBseq_1.0_381_RIGHT 1 - GTCTTCCCAGCATTCGGTCC

NC_000962.3 702651 702670 TBseq_1.0_382_LEFT 2 + GTGGAACTGCCTGAGTCGC

NC_000962.3 704645 704665 TBseq_1.0_382_RIGHT 2 - ACGAGAAGTGCGTGAGATGC

NC_000962.3 704491 704511 TBseq_1.0_383_LEFT 1 + GGGCACGGCTTTCGAGATAG

NC_000962.3 706469 706489 TBseq_1.0_383_RIGHT 1 - GCCAGTAGAGGTTCCACAGC

NC_000962.3 706205 706225 TBseq_1.0_384_LEFT 2 + GCGGTAACCAATCGAGGCAC

NC_000962.3 708121 708142 TBseq_1.0_384_RIGHT 2 - CCGCAAGTACAAGAAATGCCA

NC_000962.3 707923 707941 TBseq_1.0_385_LEFT 1 + AACAACACCGCGTCCAGC

NC_000962.3 709904 709924 TBseq_1.0_385_RIGHT 1 - GGACTAGTGCCGCCATCTTG

NC_000962.3 709245 709262 TBseq_1.0_386_LEFT 2 + GGTCACGCCGAGCATGT

NC_000962.3 711237 711257 TBseq_1.0_386_RIGHT 2 - GACACCGGCGAAAGAGAGTG

NC_000962.3 711047 711065 TBseq_1.0_387_LEFT 1 + GTCGCCGAGCATGTGCAT

NC_000962.3 713016 713034 TBseq_1.0_387_RIGHT 1 - AAAGCCCCGACCCGATCT

NC_000962.3 712817 712836 TBseq_1.0_388_LEFT 2 + GTTGTCACGTTCACCCCCG

NC_000962.3 714796 714816 TBseq_1.0_388_RIGHT 2 - CGGAAAGACCAGGGTTCGAC

NC_000962.3 714610 714630 TBseq_1.0_389_LEFT 1 + AGTCGATCGACCTGACGTCC

NC_000962.3 716635 716655 TBseq_1.0_389_RIGHT 1 - CAATCCGCGCTCGTCATAGC

NC_000962.3 716419 716439 TBseq_1.0_390_LEFT 2 + GTATCAAGCACCCGGAAGCC

NC_000962.3 718463 718482 TBseq_1.0_390_RIGHT 2 - GGCAACGGATTCCAGGGTC

NC_000962.3 718162 718182 TBseq_1.0_391_LEFT 1 + CGTTGGCTGAGCAAAACGGA

NC_000962.3 720203 720222 TBseq_1.0_391_RIGHT 1 - ACAAGAGTCAGGGCAGCCA

NC_000962.3 720009 720029 TBseq_1.0_392_LEFT 2 + CACACCCCGTCGACTGTAAC

NC_000962.3 721961 721981 TBseq_1.0_392_RIGHT 2 - GCTGCATTCGGACTACCCAC

NC_000962.3 721750 721769 TBseq_1.0_393_LEFT 1 + TCGAGCAGATCCGAGAGCG

NC_000962.3 723705 723723 TBseq_1.0_393_RIGHT 1 - CATCACAAAGACGCGCGG

NC_000962.3 723411 723429 TBseq_1.0_394_LEFT 2 + CAGCTGAGCGGCCTGAAA

NC_000962.3 725312 725332 TBseq_1.0_394_RIGHT 2 - GGGAGCTGGTGTTGCTGTAC

NC_000962.3 725117 725136 TBseq_1.0_395_LEFT 1 + GAGCAACACCTCGAAGGGT

NC_000962.3 727144 727164 TBseq_1.0_395_RIGHT 1 - CACCTACGCACCGTTGATCG

NC_000962.3 727006 727025 TBseq_1.0_396_LEFT 2 + TTTCGGCGATGGTGAGCAG

NC_000962.3 728901 728920 TBseq_1.0_396_RIGHT 2 - CGGCCCACGCATACAACAT

NC_000962.3 728646 728663 TBseq_1.0_397_LEFT 1 + CGGGAGCGCAACTTGGT

NC_000962.3 730654 730674 TBseq_1.0_397_RIGHT 1 - CGACCTGATCACCCATGAGC

NC_000962.3 730432 730452 TBseq_1.0_398_LEFT 2 + GAAAGACGGTTGGGTCACCC

NC_000962.3 732502 732522 TBseq_1.0_398_RIGHT 2 - CGTCCCAGTGAATCGGGTTC

NC_000962.3 732310 732330 TBseq_1.0_399_LEFT 1 + ACCAACGAGGAAGGTGACCT

NC_000962.3 734283 734303 TBseq_1.0_399_RIGHT 1 - GTTAGATCGACCGCCTCACC

NC_000962.3 734081 734101 TBseq_1.0_400_LEFT 2 + ATGCGGAAGGTAATCTGGCC

NC_000962.3 736035 736055 TBseq_1.0_400_RIGHT 2 - AACGACGCTTTCCCGATGAC

NC_000962.3 735816 735836 TBseq_1.0_401_LEFT 1 + ATCGAGAGGATTCAGGGCGG

NC_000962.3 737841 737860 TBseq_1.0_401_RIGHT 1 - TCGGCTTGACCCTGTCGAA

NC_000962.3 737689 737708 TBseq_1.0_402_LEFT 2 + CGTAGCGCTCGAAACCGAA

NC_000962.3 739671 739692 TBseq_1.0_402_RIGHT 2 - CACAGCCTGTTCACCTACGAC

NC_000962.3 739473 739493 TBseq_1.0_403_LEFT 1 + CCATGCATCGAGAGTCCGTG

NC_000962.3 741493 741513 TBseq_1.0_403_RIGHT 1 - GTGAAGAAGGACCACGCAGC

NC_000962.3 741265 741285 TBseq_1.0_404_LEFT 2 + TAGCGCTCCACGTACAGGAA

NC_000962.3 743341 743361 TBseq_1.0_404_RIGHT 2 - CGTCCTTGCCGGTGTAGATC

NC_000962.3 743183 743202 TBseq_1.0_405_LEFT 1 + CCGAACAAGTGGGTCACCG

NC_000962.3 745104 745124 TBseq_1.0_405_RIGHT 1 - GAAATTCGAGCCATCCGGGG

NC_000962.3 744680 744698 TBseq_1.0_406_LEFT 2 + TTCACCAAGGCCGTGCTG

NC_000962.3 746676 746693 TBseq_1.0_406_RIGHT 2 - TGCCGATCTCCAGCCGA

NC_000962.3 746348 746368 TBseq_1.0_407_LEFT 1 + AAGGTGAGCGTGACATGAGC

NC_000962.3 748337 748356 TBseq_1.0_407_RIGHT 1 - TTCGGTGATCAACGTCGCG

NC_000962.3 748127 748144 TBseq_1.0_408_LEFT 2 + AGGAGGACGAGGCAGCA

NC_000962.3 750170 750190 TBseq_1.0_408_RIGHT 2 - GTGAACCAGTGGTAGGTGGC

NC_000962.3 750031 750052 TBseq_1.0_409_LEFT 1 + CCCATATCTCGAGGGCTTCCT

NC_000962.3 751973 751993 TBseq_1.0_409_RIGHT 1 - CTTCTTCTCGTCCCCACCCA

NC_000962.3 751799 751819 TBseq_1.0_410_LEFT 2 + CGGCCAAGGAGCTTTACGAG

NC_000962.3 753733 753753 TBseq_1.0_410_RIGHT 2 - CAACCTATTGCCCGGGATCG

NC_000962.3 753523 753543 TBseq_1.0_411_LEFT 1 + CGAGGTTCACGGCTTCCTTC

NC_000962.3 755526 755546 TBseq_1.0_411_RIGHT 1 - CACCATCAATCGAGGCCACC

NC_000962.3 755304 755323 TBseq_1.0_412_LEFT 2 + GGGGCTTATGCGACTGCTC

NC_000962.3 757367 757387 TBseq_1.0_412_RIGHT 2 - CGAGGCCACTTGTTTGGTCC

NC_000962.3 757073 757092 TBseq_1.0_413_LEFT 1 + AGGTCTACGCGGGCTTTCT

NC_000962.3 759094 759114 TBseq_1.0_413_RIGHT 1 - GCAATGAGAGCGTCGGTCAG

NC_000962.3 758918 758938 TBseq_1.0_414_LEFT 2 + GTGAGCTGCAACTCGGTCTG

NC_000962.3 760931 760951 TBseq_1.0_414_RIGHT 2 - TGGTTTTGGATCAGCTCGCC

NC_000962.3 760795 760813 TBseq_1.0_415_LEFT 1 + AGACGTCGTGGCCACCAT

NC_000962.3 762687 762706 TBseq_1.0_415_RIGHT 1 - GACACAATGGCGTTCGGCT

NC_000962.3 762487 762507 TBseq_1.0_416_LEFT 2 + GGTTGAGGACATGCCGTTCC

NC_000962.3 764448 764466 TBseq_1.0_416_RIGHT 2 - ATGATTTCCGGCGCACCC

NC_000962.3 764219 764239 TBseq_1.0_417_LEFT 1 + GCAAGGGGCAGAAGAAGCTT

NC_000962.3 766203 766223 TBseq_1.0_417_RIGHT 1 - GAGCGTCAATCTCCGGATCG

NC_000962.3 765988 766005 TBseq_1.0_418_LEFT 2 + ACCCGACGTCTGGTGGA

NC_000962.3 768019 768038 TBseq_1.0_418_RIGHT 2 - GTCGTTCGGAGCGGTCATC

NC_000962.3 767783 767803 TBseq_1.0_419_LEFT 1 + GTGGTATCGCCGGGAACATC

NC_000962.3 769812 769829 TBseq_1.0_419_RIGHT 1 - AGCGGATCGGTTGGGCT

NC_000962.3 769677 769696 TBseq_1.0_420_LEFT 2 + AACACTTTATGCGCCGGGG

NC_000962.3 771566 771585 TBseq_1.0_420_RIGHT 2 - GCCACCCTCCTGGATCAGA

NC_000962.3 771382 771401 TBseq_1.0_421_LEFT 1 + ACCCAGACACTGTGGCGAT

NC_000962.3 773327 773345 TBseq_1.0_421_RIGHT 1 - AGCCGGCACAGAATCCCT

NC_000962.3 773172 773192 TBseq_1.0_422_LEFT 2 + CTATGAGGTCACCGGTCGGA

NC_000962.3 775161 775180 TBseq_1.0_422_RIGHT 2 - CAGCGACGGCAAAACACAC

NC_000962.3 774998 775017 TBseq_1.0_423_LEFT 1 + GAGGCCAACTCTGTGCACC

NC_000962.3 776997 777016 TBseq_1.0_423_RIGHT 1 - GGCAAACCGATCGAGCACA

NC_000962.3 776848 776867 TBseq_1.0_424_LEFT 2 + CTGGGTGAGGCTGTGCATC

NC_000962.3 778822 778840 TBseq_1.0_424_RIGHT 2 - CTTCACGGTGCAGCGGAT

NC_000962.3 778619 778639 TBseq_1.0_425_LEFT 1 + CCGTGGTCTTCAAGGTGAGC

NC_000962.3 780613 780632 TBseq_1.0_425_RIGHT 1 - GTGATCGGCTTTGGGTCCG

NC_000962.3 780432 780450 TBseq_1.0_426_LEFT 2 + ACTGGGCGGGTGATGCTT

NC_000962.3 782428 782450 TBseq_1.0_426_RIGHT 2 - TGTCTATCACTGTCGGTTTGCG

NC_000962.3 782261 782281 TBseq_1.0_427_LEFT 1 + AACGCCGTGAGAAGACGATG

NC_000962.3 784226 784247 TBseq_1.0_427_RIGHT 1 - GCCATTTCCGAGGAGTCAACC

NC_000962.3 784025 784046 TBseq_1.0_428_LEFT 2 + GGTCATCATCAACCTCGAGCC

NC_000962.3 786076 786095 TBseq_1.0_428_RIGHT 2 - GGGCATTTTCGCGAGCAGA

NC_000962.3 785925 785944 TBseq_1.0_429_LEFT 1 + TCGCCATGGACGAAGGTCT

NC_000962.3 787923 787942 TBseq_1.0_429_RIGHT 1 - CATACACGATCGCGCGTCA

NC_000962.3 787703 787723 TBseq_1.0_430_LEFT 2 + TCCTTACTCGGTCGACACCC

NC_000962.3 789774 789791 TBseq_1.0_430_RIGHT 2 - GTGGCGCGACACAAGCT

NC_000962.3 789551 789571 TBseq_1.0_431_LEFT 1 + GTCGGTAGCATCGAGGATGG

NC_000962.3 791559 791579 TBseq_1.0_431_RIGHT 1 - GGTTTCACCGATGTCAGCGT

NC_000962.3 791364 791385 TBseq_1.0_432_LEFT 2 + TCGTCAAAGGTGTTGAAGGCC

NC_000962.3 793359 793377 TBseq_1.0_432_RIGHT 2 - GTTGCTGCGCGATGGCTA

NC_000962.3 793178 793197 TBseq_1.0_433_LEFT 1 + CGTGCAAGGCCATAGCGAG

NC_000962.3 795204 795223 TBseq_1.0_433_RIGHT 1 - ACGGATGTTTCGGTGTGGC

NC_000962.3 795031 795052 TBseq_1.0_434_LEFT 2 + GTTGCTCGTGGAGTATGGCAG

NC_000962.3 797021 797040 TBseq_1.0_434_RIGHT 2 - CACGGTCACCACACAGCTC

NC_000962.3 796850 796868 TBseq_1.0_435_LEFT 1 + TTGGCTTATGGCGCTGGC

NC_000962.3 798830 798850 TBseq_1.0_435_RIGHT 1 - GTTACCCCTGCGACCCAATG

NC_000962.3 798607 798628 TBseq_1.0_436_LEFT 2 + TTGTCGCTTAGGGTTTCTGGC

NC_000962.3 800651 800673 TBseq_1.0_436_RIGHT 2 - CCCGCGAGTCCTTGTACTTATG

NC_000962.3 800365 800385 TBseq_1.0_437_LEFT 1 + GGTAAACAGCGGCGCAGTAT

NC_000962.3 802287 802307 TBseq_1.0_437_RIGHT 1 - TGTTCACCGATGCGACCTTG

NC_000962.3 802115 802135 TBseq_1.0_438_LEFT 2 + GAGGAGGTTTCGGCCTGATG

NC_000962.3 804022 804041 TBseq_1.0_438_RIGHT 2 - TCCACCACCACCGTGATGT

NC_000962.3 803884 803903 TBseq_1.0_439_LEFT 1 + CCAACGCGCAAAACAACGG

NC_000962.3 805872 805891 TBseq_1.0_439_RIGHT 1 - TGGGCCCCTTAACGTTTGC

NC_000962.3 805715 805735 TBseq_1.0_440_LEFT 2 + GAGAACTGGGTCTGGCGACT

NC_000962.3 807697 807716 TBseq_1.0_440_RIGHT 2 - TAGACGGTGGGCGTGATGT

NC_000962.3 807497 807517 TBseq_1.0_441_LEFT 1 + GAAGCTCTTCGACCACCTCG

NC_000962.3 809465 809484 TBseq_1.0_441_RIGHT 1 - CTGTAGCGAGCTTGACCGG

NC_000962.3 809316 809335 TBseq_1.0_442_LEFT 2 + CATTGGGCTGGGTGGGAAC

NC_000962.3 811297 811315 TBseq_1.0_442_RIGHT 2 - CTGACGTGCGCGGTCTTT

NC_000962.3 811144 811164 TBseq_1.0_443_LEFT 1 + CGACATGGGTTGATCTGCCC

NC_000962.3 813135 813155 TBseq_1.0_443_RIGHT 1 - CAGCGATTTACCGACCCGAG

NC_000962.3 812986 813007 TBseq_1.0_444_LEFT 2 + GGACCCGATCGCAGACTTTTT

NC_000962.3 814927 814946 TBseq_1.0_444_RIGHT 2 - TTTTCCGACGCGCCTTCAG

NC_000962.3 814777 814796 TBseq_1.0_445_LEFT 1 + GTGCACGACATCTTGGCCA

NC_000962.3 816673 816692 TBseq_1.0_445_RIGHT 1 - CGTTGACGATCGGGCCTTC

NC_000962.3 816514 816534 TBseq_1.0_446_LEFT 2 + GGTTCACCGGAATCCAGTGG

NC_000962.3 818440 818459 TBseq_1.0_446_RIGHT 2 - GGACATAGGGCGCAGATGC

NC_000962.3 818280 818301 TBseq_1.0_447_LEFT 1 + GTGAAGAAATCCAGCCCGACC

NC_000962.3 820213 820232 TBseq_1.0_447_RIGHT 1 - GACGACATTGGCAGCGTGA

NC_000962.3 819933 819953 TBseq_1.0_448_LEFT 2 + GCACCCCAAACGATTTGTGC

NC_000962.3 821968 821988 TBseq_1.0_448_RIGHT 2 - GCCGTGGCATAGTCGATGAC

NC_000962.3 821779 821796 TBseq_1.0_449_LEFT 1 + ATCACACCCGGGCTGCT

NC_000962.3 823687 823710 TBseq_1.0_449_RIGHT 1 - GAAGCAGAAACACCAAGACACAC

NC_000962.3 823494 823513 TBseq_1.0_450_LEFT 2 + CAAGTCGCGCTCGGAGATG

NC_000962.3 825486 825505 TBseq_1.0_450_RIGHT 2 - CGACGAACACCACACCGAC

NC_000962.3 825323 825344 TBseq_1.0_451_LEFT 1 + GTGGATGGGCGAGTTGATCAC

NC_000962.3 827328 827348 TBseq_1.0_451_RIGHT 1 - TGGTACCGAGGGTCAGCATC

NC_000962.3 827183 827203 TBseq_1.0_452_LEFT 2 + GTTATGGACGCTCGTTCGGG

NC_000962.3 829156 829177 TBseq_1.0_452_RIGHT 2 - GGGCCAACCTTACGGAATTCA

NC_000962.3 828970 828989 TBseq_1.0_453_LEFT 1 + CGGAGTGGGCTGCATGATG

NC_000962.3 830994 831014 TBseq_1.0_453_RIGHT 1 - CGTTCTCGATCTCGGCTCCA

NC_000962.3 830845 830864 TBseq_1.0_454_LEFT 2 + AGACAGGCCTTGGTGCTGA

NC_000962.3 832826 832846 TBseq_1.0_454_RIGHT 2 - ACTGACTGATCCGTGGGTGT

NC_000962.3 832679 832703 TBseq_1.0_455_LEFT 1 + CCTATGGCAAGGACTGATCGAATC

NC_000962.3 834631 834650 TBseq_1.0_455_RIGHT 1 - GGCGGGGTGGTATTTCACG

NC_000962.3 833663 833683 TBseq_1.0_456_LEFT 2 + AGACCAACGCTCCCTACGAG

NC_000962.3 835643 835663 TBseq_1.0_456_RIGHT 2 - CGACACCCCCGATGACCATA

NC_000962.3 840901 840920 TBseq_1.0_457_LEFT 1 + CCGCTCGACCGCAGATGAT

NC_000962.3 842828 842847 TBseq_1.0_457_RIGHT 1 - ACGCTGGCATTCATGGTCG

NC_000962.3 842631 842652 TBseq_1.0_458_LEFT 2 + GAACAACGATTGTGCGGACAG

NC_000962.3 844625 844642 TBseq_1.0_458_RIGHT 2 - TTCGTCTCCCGGGTGCA

NC_000962.3 844155 844173 TBseq_1.0_459_LEFT 1 + ACAAAAACGCGGCGGTCA

NC_000962.3 846127 846147 TBseq_1.0_459_RIGHT 1 - GGGTGTTGTCGCGTACTAGC

NC_000962.3 845951 845971 TBseq_1.0_460_LEFT 2 + ATGGTTGTCCTGTGCAAGCC

NC_000962.3 847984 848003 TBseq_1.0_460_RIGHT 2 - GCACGATCAGGGAAGCCTG

NC_000962.3 847992 848011 TBseq_1.0_461_LEFT 1 + CCTGATCGTGCGGAACCTG

NC_000962.3 850043 850062 TBseq_1.0_461_RIGHT 1 - TCAATGGTGTGAGGGGGCC

NC_000962.3 848079 848099 TBseq_1.0_462_LEFT 2 + GGACGATACCGGGCTAGTGT

NC_000962.3 850079 850101 TBseq_1.0_462_RIGHT 2 - CTTCGCGTTGTGTAGTTAGGCT

NC_000962.3 850095 850118 TBseq_1.0_463_LEFT 1 + GCGAAGGGATTGGGACTTCTATC

NC_000962.3 852093 852112 TBseq_1.0_463_RIGHT 1 - CCGCCTTCCACACTTCGTG

NC_000962.3 851905 851924 TBseq_1.0_464_LEFT 2 + CCCGTGACTCGCTACAGGA

NC_000962.3 853849 853869 TBseq_1.0_464_RIGHT 2 - GAAGCGCAGGCCAAGATCAG

NC_000962.3 853608 853628 TBseq_1.0_465_LEFT 1 + GCATGAGTCAGGAGGATGCG

NC_000962.3 855558 855579 TBseq_1.0_465_RIGHT 1 - ACGTGGTGTTGGCATTCATCC

NC_000962.3 855333 855351 TBseq_1.0_466_LEFT 2 + AGGCGACTTCGAAGGGCA

NC_000962.3 857348 857368 TBseq_1.0_466_RIGHT 2 - GACATGCTCGACGTGCTCAT

NC_000962.3 857135 857154 TBseq_1.0_467_LEFT 1 + AGCGCATGGAAACTCACCG

NC_000962.3 859176 859194 TBseq_1.0_467_RIGHT 1 - CGCGCACGATCTCACGTT

NC_000962.3 858995 859012 TBseq_1.0_468_LEFT 2 + TTCCATCCGGGCCAGGT

NC_000962.3 860928 860948 TBseq_1.0_468_RIGHT 2 - TGAGCAGCGCACTAATTCCG

NC_000962.3 860764 860785 TBseq_1.0_469_LEFT 1 + CAGCTAACATGGGACTGTCCG

NC_000962.3 862718 862741 TBseq_1.0_469_RIGHT 1 - TTTCTTGTAGTAGTCCAACGGCA

NC_000962.3 862546 862565 TBseq_1.0_470_LEFT 2 + AAGCAGATTGTCGCCGACG

NC_000962.3 864520 864538 TBseq_1.0_470_RIGHT 2 - GGCGACGTTGGCTTTCGT

NC_000962.3 864339 864359 TBseq_1.0_471_LEFT 1 + GCTCGAGGTCCAAGTCAACG

NC_000962.3 866275 866295 TBseq_1.0_471_RIGHT 1 - CGGCATTTCGCTGCACAATC

NC_000962.3 866030 866048 TBseq_1.0_472_LEFT 2 + CCGCATACCGTTCACCCA

NC_000962.3 868025 868043 TBseq_1.0_472_RIGHT 2 - GATCGCACTGCACGGCTT

NC_000962.3 867830 867849 TBseq_1.0_473_LEFT 1 + AGCCGCTGGGTGTCCAATA

NC_000962.3 869815 869835 TBseq_1.0_473_RIGHT 1 - CTCTGACAGAGGCGCAAGTG

NC_000962.3 869524 869546 TBseq_1.0_474_LEFT 2 + TGTCGGAAGTCTCGATTCAGTG

NC_000962.3 871512 871531 TBseq_1.0_474_RIGHT 2 - GCTTCACGACGGTGGATGG

NC_000962.3 871342 871363 TBseq_1.0_475_LEFT 1 + GTCGATGATGTGGTCGCGATG

NC_000962.3 873349 873369 TBseq_1.0_475_RIGHT 1 - CTGGTAGTCGGACAATGCGG

NC_000962.3 873113 873130 TBseq_1.0_476_LEFT 2 + ATCCCGTGCGAGCCGAT

NC_000962.3 875166 875186 TBseq_1.0_476_RIGHT 2 - GGGGCCTCTACCAGTGTGAA

NC_000962.3 875001 875021 TBseq_1.0_477_LEFT 1 + GGGTCGTCCATCACTTCGGA

NC_000962.3 876928 876948 TBseq_1.0_477_RIGHT 1 - ATGACCTTTCGCACGCCTAC

NC_000962.3 876764 876782 TBseq_1.0_478_LEFT 2 + GCTTTTCGACGCTGTGGC

NC_000962.3 878716 878735 TBseq_1.0_478_RIGHT 2 - GCCACCAGCAACGATACCG

NC_000962.3 878580 878600 TBseq_1.0_479_LEFT 1 + CTTCACCGTCCGGAAACCTG

NC_000962.3 880528 880547 TBseq_1.0_479_RIGHT 1 - TCGATGAATGCCTGCACCG

NC_000962.3 880320 880340 TBseq_1.0_480_LEFT 2 + TTGCCGGTACCGTTGTTTCC

NC_000962.3 882282 882302 TBseq_1.0_480_RIGHT 2 - GTTCGCTGATCGTGCTGCTT

NC_000962.3 882074 882092 TBseq_1.0_481_LEFT 1 + AGGCTCAGGTGCTGCACT

NC_000962.3 884055 884075 TBseq_1.0_481_RIGHT 1 - GTGAATCTCACGACGCCTCC

NC_000962.3 883903 883925 TBseq_1.0_482_LEFT 2 + CTCGCCTATCGACTCAGTCATG

NC_000962.3 885820 885840 TBseq_1.0_482_RIGHT 2 - ATGAACGCCAAAGACGACCC

NC_000962.3 885676 885695 TBseq_1.0_483_LEFT 1 + CCGGGGCTTATCGTCAGGA

NC_000962.3 887662 887681 TBseq_1.0_483_RIGHT 1 - TTGACGACGGCTGGCTCTA

NC_000962.3 887480 887500 TBseq_1.0_484_LEFT 2 + CGGGGTCGGTAAAGAATGCC

NC_000962.3 889505 889525 TBseq_1.0_484_RIGHT 2 - TCGTAGTAGGTCGATGGGGC

NC_000962.3 889309 889327 TBseq_1.0_485_LEFT 1 + GGGACAACGCCGAATTGC

NC_000962.3 891291 891311 TBseq_1.0_485_RIGHT 1 - CCTTTGGTAGTAGGCCTGGC

NC_000962.3 891056 891075 TBseq_1.0_486_LEFT 2 + CTGGAAATCCCTGGCTGCG

NC_000962.3 893088 893108 TBseq_1.0_486_RIGHT 2 - CACTTGTCCGTGGTCGTCTC

NC_000962.3 892927 892947 TBseq_1.0_487_LEFT 1 + CCAACTCGAAGCACACGTCC

NC_000962.3 894901 894920 TBseq_1.0_487_RIGHT 1 - AAGCTCTCGCCGACCTGAT

NC_000962.3 894714 894732 TBseq_1.0_488_LEFT 2 + ACGACGCGTGAACTGCTG

NC_000962.3 896714 896734 TBseq_1.0_488_RIGHT 2 - CAGCTGGATCGTCATTCCGC

NC_000962.3 896580 896600 TBseq_1.0_489_LEFT 1 + CGACCCGTTCATGGAGAAGG

NC_000962.3 898466 898485 TBseq_1.0_489_RIGHT 1 - CTGCAGTATCCGTCCACCC

NC_000962.3 898330 898350 TBseq_1.0_490_LEFT 2 + CGATGTCGGCTCGTGAGTTG

NC_000962.3 900279 900300 TBseq_1.0_490_RIGHT 2 - GGTTCATCGAAGCCAAGACCC

NC_000962.3 900136 900156 TBseq_1.0_491_LEFT 1 + CACACTCTTGCGCAACGGTA

NC_000962.3 902045 902065 TBseq_1.0_491_RIGHT 1 - GCCACCCTCACACACCATAG

NC_000962.3 901827 901847 TBseq_1.0_492_LEFT 2 + CGGTGCAGTGCATTTCTGGG

NC_000962.3 903816 903836 TBseq_1.0_492_RIGHT 2 - GCTTGAACAGGTCGATGGCG

NC_000962.3 903682 903701 TBseq_1.0_493_LEFT 1 + GTTGGGCGCTGACAAAACG

NC_000962.3 905634 905654 TBseq_1.0_493_RIGHT 1 - CGATGAACGCACCATCCCTC

NC_000962.3 905496 905517 TBseq_1.0_494_LEFT 2 + CGTCCAGATGCAACAACACCA

NC_000962.3 907466 907485 TBseq_1.0_494_RIGHT 2 - CAGTCGGTCTGGCGTTCTG

NC_000962.3 907323 907343 TBseq_1.0_495_LEFT 1 + CATACCGCTCGCCGTTATCC

NC_000962.3 909221 909241 TBseq_1.0_495_RIGHT 1 - AGTGGACGAGGACACCAGTG

NC_000962.3 909075 909095 TBseq_1.0_496_LEFT 2 + GATCACCGTGTCCTCGTTGG

NC_000962.3 910967 910990 TBseq_1.0_496_RIGHT 2 - GCAGTAATAACTCCAACAAGCCC

NC_000962.3 910605 910628 TBseq_1.0_497_LEFT 1 + AAATGCCTTGATTTCTAGCTCCG

NC_000962.3 912679 912699 TBseq_1.0_497_RIGHT 1 - GGGGGAACATCTTCGGTCAG

NC_000962.3 912404 912424 TBseq_1.0_498_LEFT 2 + AAAGTGCATCCCGATCACCC

NC_000962.3 914438 914458 TBseq_1.0_498_RIGHT 2 - CGGCCAGTCAAAGATCGAGC

NC_000962.3 914208 914228 TBseq_1.0_499_LEFT 1 + CCTACGTTCCCGACCTTGGA

NC_000962.3 916250 916270 TBseq_1.0_499_RIGHT 1 - CGAGTCAGCATTCCCCGATG

NC_000962.3 916099 916119 TBseq_1.0_500_LEFT 2 + GTGGGGAGGTCAGGAAAAGC

NC_000962.3 918099 918118 TBseq_1.0_500_RIGHT 2 - GTTCGACCTCGTGCCCAAC

NC_000962.3 917951 917971 TBseq_1.0_501_LEFT 1 + GTACCGGCATGACGACTTCG

NC_000962.3 919935 919953 TBseq_1.0_501_RIGHT 1 - TACAACGACCGCAGCAGC

NC_000962.3 919707 919727 TBseq_1.0_502_LEFT 2 + TGGCAGGCCTATAGGGTTCC

NC_000962.3 921690 921710 TBseq_1.0_502_RIGHT 2 - CGTAGCGTGCGAGGTTGATC

NC_000962.3 921477 921497 TBseq_1.0_503_LEFT 1 + GCACGTTGAGAGTGGTACCG

NC_000962.3 923443 923461 TBseq_1.0_503_RIGHT 1 - CACCGCCGCTTCACTGAA

NC_000962.3 922981 923002 TBseq_1.0_504_LEFT 2 + GACACTAAGGCGTCCCTGTTG

NC_000962.3 924926 924946 TBseq_1.0_504_RIGHT 2 - CATCTCCTCCCACAGCGACA

NC_000962.3 930512 930531 TBseq_1.0_505_LEFT 1 + TCTGACTGTCGCCCATCGT

NC_000962.3 932416 932436 TBseq_1.0_505_RIGHT 1 - GTCACGACTTCGACCTACGC

NC_000962.3 932222 932242 TBseq_1.0_506_LEFT 2 + GTGAGCTGGTCGAGTAACGC

NC_000962.3 934218 934238 TBseq_1.0_506_RIGHT 2 - TCAGACAGGCAACGAGGAGG

NC_000962.3 934008 934028 TBseq_1.0_507_LEFT 1 + AGGTCAGCCCCGATCTGATC

NC_000962.3 936052 936071 TBseq_1.0_507_RIGHT 1 - AAGGTCCAACCACCGGTCC

NC_000962.3 935896 935915 TBseq_1.0_508_LEFT 2 + GTTCGATGTCGTCCACGCA

NC_000962.3 937892 937911 TBseq_1.0_508_RIGHT 2 - CTGGGCTCCGATTGTGCTG

NC_000962.3 937708 937728 TBseq_1.0_509_LEFT 1 + CTACGGCCCGATTCATGGTG

NC_000962.3 939697 939717 TBseq_1.0_509_RIGHT 1 - AATGTGCGCATCCTCGTCAG

NC_000962.3 939536 939556 TBseq_1.0_510_LEFT 2 + CGGCCAGGAAGCAGTAAGTG

NC_000962.3 941526 941546 TBseq_1.0_510_RIGHT 2 - TATCAAAACGTCGACGGCGG

NC_000962.3 941319 941339 TBseq_1.0_511_LEFT 1 + CGGTATTGCGGATCGGTGTC

NC_000962.3 943369 943388 TBseq_1.0_511_RIGHT 1 - GCGAATCCGGATCCGCATC

NC_000962.3 943189 943209 TBseq_1.0_512_LEFT 2 + GAGTGCGACCAGGGGAAAGA

NC_000962.3 945203 945223 TBseq_1.0_512_RIGHT 2 - GAGTGCCACCGGTTGATTCG

NC_000962.3 945022 945042 TBseq_1.0_513_LEFT 1 + CCATGGTTGGCAATACCCCG

NC_000962.3 946978 946997 TBseq_1.0_513_RIGHT 1 - CAATGCCATGACCGCAACG

NC_000962.3 946770 946789 TBseq_1.0_514_LEFT 2 + CGATCCTCATGCCACGCAA

NC_000962.3 948811 948830 TBseq_1.0_514_RIGHT 2 - GGCCACACTCTACCGGGAT

NC_000962.3 948643 948662 TBseq_1.0_515_LEFT 1 + ACGATCCAGGTGCCACTGT

NC_000962.3 950645 950665 TBseq_1.0_515_RIGHT 1 - GCAACGGCCGGTAGAGAAAT

NC_000962.3 950456 950476 TBseq_1.0_516_LEFT 2 + GTCAACTGGTGGTCGGCAAT

NC_000962.3 952414 952433 TBseq_1.0_516_RIGHT 2 - CCACATCGAGCTGAGTCGG

NC_000962.3 952235 952255 TBseq_1.0_517_LEFT 1 + GGACCATGAAGGGGATTGGC

NC_000962.3 954183 954202 TBseq_1.0_517_RIGHT 1 - AAATCGGATGGGCTTGCGG

NC_000962.3 953968 953988 TBseq_1.0_518_LEFT 2 + GGAAGTACGTGCCGTAGCTG

NC_000962.3 955958 955978 TBseq_1.0_518_RIGHT 2 - GTGAGCATGATCACCGGGTC

NC_000962.3 955786 955805 TBseq_1.0_519_LEFT 1 + GGCGCTGCAGAAGTACCAC

NC_000962.3 957730 957750 TBseq_1.0_519_RIGHT 1 - CAGGGCCTCGTCAGAAGTCT

NC_000962.3 957570 957591 TBseq_1.0_520_LEFT 2 + GAAGACATCGTCGAGCCCAAC

NC_000962.3 959490 959509 TBseq_1.0_520_RIGHT 2 - GCGACGACGTTGATCCTGG

NC_000962.3 959340 959359 TBseq_1.0_521_LEFT 1 + TAGTGCGGCGGGTGATCAT

NC_000962.3 961246 961267 TBseq_1.0_521_RIGHT 1 - CACTGTTGGACGAGGCCTTAG

NC_000962.3 960982 961005 TBseq_1.0_522_LEFT 2 + AACGATTTGCGAAGAATTCCTGC

NC_000962.3 962977 962997 TBseq_1.0_522_RIGHT 2 - CTTCGTGGTTGCCTTCTCGG

NC_000962.3 962836 962856 TBseq_1.0_523_LEFT 1 + ACGACCTGCCCTACATCCAC

NC_000962.3 964747 964768 TBseq_1.0_523_RIGHT 1 - GGAACTTGCCGTAAACGACCT

NC_000962.3 964533 964550 TBseq_1.0_524_LEFT 2 + AGCTGTGGCGGGCTGAT

NC_000962.3 966547 966566 TBseq_1.0_524_RIGHT 2 - AACTCTGGCTGGTCGACGC

NC_000962.3 966330 966348 TBseq_1.0_525_LEFT 1 + GGCGGGTTTGCTCCACAT

NC_000962.3 968375 968394 TBseq_1.0_525_RIGHT 1 - GAGTGCGGCGGAATCGATC

NC_000962.3 968287 968307 TBseq_1.0_526_LEFT 2 + CGGGAGTTCGAGTCCTAACG

NC_000962.3 970276 970298 TBseq_1.0_526_RIGHT 2 - CGCTAACGTTAGTAACAGCCGA

NC_000962.3 968395 968415 TBseq_1.0_527_LEFT 1 + GTTTGACGAGACGACGACCG

NC_000962.3 970400 970420 TBseq_1.0_527_RIGHT 1 - CCGCTACCAAAGTCACGGTC

NC_000962.3 970311 970336 TBseq_1.0_528_LEFT 2 + GCGTCTCCAGTTTCTATTTCTTGAC

NC_000962.3 972312 972332 TBseq_1.0_528_RIGHT 2 - CTTGCTCCAGCCAGGTGTAG

NC_000962.3 972177 972197 TBseq_1.0_529_LEFT 1 + GGGTGAGCAGGCATACGAAC

NC_000962.3 974102 974122 TBseq_1.0_529_RIGHT 1 - TCGACCTCGACGACTGTCAG

NC_000962.3 973876 973895 TBseq_1.0_530_LEFT 2 + ACGTCAGCAACTGCACGAC

NC_000962.3 975863 975882 TBseq_1.0_530_RIGHT 2 - GCATGGCCAACTACCCTCC

NC_000962.3 974919 974939 TBseq_1.0_531_LEFT 1 + CCAACGGTTGTCGGAGTGTT

NC_000962.3 976848 976872 TBseq_1.0_531_RIGHT 1 - AGAGAAAACCTATGATTCAGCCGG

NC_000962.3 976650 976670 TBseq_1.0_532_LEFT 2 + GACTGCGGTTTCTTCCTGCC

NC_000962.3 978548 978568 TBseq_1.0_532_RIGHT 2 - GACCATCTTCGTCTGGCAGC

NC_000962.3 978343 978363 TBseq_1.0_533_LEFT 1 + GTATGTCCAATCGCGGCACA

NC_000962.3 980304 980321 TBseq_1.0_533_RIGHT 1 - TCAGCACCACGACCGCA

NC_000962.3 980095 980115 TBseq_1.0_534_LEFT 2 + ACTGCGGATTAGCGATGTGC

NC_000962.3 982099 982116 TBseq_1.0_534_RIGHT 2 - ATCGCCTGGGCCCACAA

NC_000962.3 981963 981983 TBseq_1.0_535_LEFT 1 + TGCGAAGTAGTAGGCGTCGG

NC_000962.3 983943 983963 TBseq_1.0_535_RIGHT 1 - GTTACGCAGGCACCACAGTC

NC_000962.3 983786 983805 TBseq_1.0_536_LEFT 2 + TGCTACGAGTGCACGCATG

NC_000962.3 985721 985741 TBseq_1.0_536_RIGHT 2 - CGGAACACCGGTCACCATTC

NC_000962.3 985509 985529 TBseq_1.0_537_LEFT 1 + GTGCTAACCGTCCCCTTGTG

NC_000962.3 987533 987553 TBseq_1.0_537_RIGHT 1 - CGGAGTGCGGTTTTCGATGA

NC_000962.3 987399 987418 TBseq_1.0_538_LEFT 2 + TTCGTTGGCTGCGATACCG

NC_000962.3 989373 989393 TBseq_1.0_538_RIGHT 2 - GAGCCAGACCCCAGACACAT

NC_000962.3 989116 989137 TBseq_1.0_539_LEFT 1 + CGAACCCCATCAGTTTCTCGC

NC_000962.3 991137 991155 TBseq_1.0_539_RIGHT 1 - TGGCACGCGCTATTGACG

NC_000962.3 990945 990965 TBseq_1.0_540_LEFT 2 + CTTCGGTCAGATTGCCAGCC

NC_000962.3 992900 992919 TBseq_1.0_540_RIGHT 2 - GGTACAGTTGCGCGACGAA

NC_000962.3 992713 992734 TBseq_1.0_541_LEFT 1 + ACAACTGCATGACCCATTCGG

NC_000962.3 994689 994709 TBseq_1.0_541_RIGHT 1 - CGTTTGCACATCGGCTCGTA

NC_000962.3 994468 994485 TBseq_1.0_542_LEFT 2 + TGCCGTGGCCTAACCCT

NC_000962.3 996494 996514 TBseq_1.0_542_RIGHT 2 - GCATAAGCGAGCCCTCTCAC

NC_000962.3 996285 996305 TBseq_1.0_543_LEFT 1 + CGGTCCGCATTCATCGAACT

NC_000962.3 998296 998314 TBseq_1.0_543_RIGHT 1 - AGCCGCATCACCGAGACT

NC_000962.3 998118 998136 TBseq_1.0_544_LEFT 2 + TGCGGCTTAGCGCATTGC

NC_000962.3 1000134 1000158 TBseq_1.0_544_RIGHT 2 - GTGCAAGATGAACAACATGTCCAG

NC_000962.3 999918 999938 TBseq_1.0_545_LEFT 1 + GATGCTCTGGACCCCATGGA

NC_000962.3 1001915 1001932 TBseq_1.0_545_RIGHT 1 - GAATACGGCGGCCACGA

NC_000962.3 1001765 1001785 TBseq_1.0_546_LEFT 2 + TTCTGCATCAGCGACGGTTC

NC_000962.3 1003757 1003777 TBseq_1.0_546_RIGHT 2 - CTCGACGCGACGATTCTTGG

NC_000962.3 1003536 1003556 TBseq_1.0_547_LEFT 1 + GGTAGCCGACAAGCTCAAGG

NC_000962.3 1005530 1005550 TBseq_1.0_547_RIGHT 1 - CTGCCCAAACTGCAAGACGA

NC_000962.3 1005153 1005170 TBseq_1.0_548_LEFT 2 + ACGCAAGCGCCTCCTTG

NC_000962.3 1007167 1007186 TBseq_1.0_548_RIGHT 2 - TGGTGCTGGTCATTGACGC

NC_000962.3 1006943 1006960 TBseq_1.0_549_LEFT 1 + ACACGATCGCGCTGGCT

NC_000962.3 1008922 1008943 TBseq_1.0_549_RIGHT 1 - GACGGTTAAGCCCCTTGGAAC

NC_000962.3 1008681 1008700 TBseq_1.0_550_LEFT 2 + CCGAGATCGCACTGCACAC

NC_000962.3 1010681 1010704 TBseq_1.0_550_RIGHT 2 - CGGTCATAACCCAAATCCTCCAA

NC_000962.3 1010490 1010510 TBseq_1.0_551_LEFT 1 + CGTCGGTGTCCAAATCGGTC

NC_000962.3 1012518 1012538 TBseq_1.0_551_RIGHT 1 - CGAAGGCGATCGAGGTCATC

NC_000962.3 1012318 1012337 TBseq_1.0_552_LEFT 2 + GAGGCCAGCAAGTTCACCC

NC_000962.3 1014334 1014356 TBseq_1.0_552_RIGHT 2 - GGAGATCCGCTCTACCGAGTAC

NC_000962.3 1014103 1014125 TBseq_1.0_553_LEFT 1 + AAACCGAGAGTGTGGCGATAAC

NC_000962.3 1016093 1016112 TBseq_1.0_553_RIGHT 1 - ATCGGCCATACGGGGTGTT

NC_000962.3 1015902 1015923 TBseq_1.0_554_LEFT 2 + CCGACACTGACGAATTCTGCT

NC_000962.3 1017890 1017909 TBseq_1.0_554_RIGHT 2 - CGGTTTGCGGTTGTTGCTC

NC_000962.3 1017750 1017770 TBseq_1.0_555_LEFT 1 + CGTTCCTCCGGTGTCAAGTG

NC_000962.3 1019717 1019737 TBseq_1.0_555_RIGHT 1 - GTCTGCGACGATCTCTGGGA

NC_000962.3 1018075 1018095 TBseq_1.0_556_LEFT 2 + TTGTTCTGGTCGACAACGCC

NC_000962.3 1020017 1020034 TBseq_1.0_556_RIGHT 2 - GCGCGGAAAATGCCTGG

NC_000962.3 1019901 1019921 TBseq_1.0_557_LEFT 1 + CTTTGCTGAGGTTGCGAGCA

NC_000962.3 1021828 1021848 TBseq_1.0_557_RIGHT 1 - CGTTCGGGAATGGCGAATCA

NC_000962.3 1021661 1021683 TBseq_1.0_558_LEFT 2 + GATCGCTTCATCTGATCGGTCG

NC_000962.3 1023631 1023650 TBseq_1.0_558_RIGHT 2 - TGACGTGCAGCAGCCTAGG

NC_000962.3 1023497 1023516 TBseq_1.0_559_LEFT 1 + TCCTGATCGGAGGTGCTGG

NC_000962.3 1025392 1025412 TBseq_1.0_559_RIGHT 1 - GGTATTCAAAGGCGTCCCCG

NC_000962.3 1025205 1025225 TBseq_1.0_560_LEFT 2 + GAGCCAGGCTACGTCATCTG

NC_000962.3 1027199 1027218 TBseq_1.0_560_RIGHT 2 - CCAGGATCAACCGGCCAAC

NC_000962.3 1027029 1027048 TBseq_1.0_561_LEFT 1 + CCAACGCTCTGGGGTTCAG

NC_000962.3 1028999 1029018 TBseq_1.0_561_RIGHT 1 - CAGCCGTGGTGGATTTGGC

NC_000962.3 1028848 1028866 TBseq_1.0_562_LEFT 2 + GACGTGGCCGCGATGAAA

NC_000962.3 1030752 1030773 TBseq_1.0_562_RIGHT 2 - CACATTGGTGCTCTCACAGGT

NC_000962.3 1030600 1030621 TBseq_1.0_563_LEFT 1 + TCAGCATCACGTTGAGCAGAC

NC_000962.3 1032528 1032546 TBseq_1.0_563_RIGHT 1 - TCGCCAGAGCTCAGCCAT

NC_000962.3 1032374 1032393 TBseq_1.0_564_LEFT 2 + GATACAGGGCGGGCCATTC

NC_000962.3 1034304 1034325 TBseq_1.0_564_RIGHT 2 - CTGCTAAGCACCTCGACCAAG

NC_000962.3 1034048 1034067 TBseq_1.0_565_LEFT 1 + AGGTCAGGATGGAACCCGG

NC_000962.3 1036009 1036030 TBseq_1.0_565_RIGHT 1 - CACGTCAACCTCAGATCAGGC

NC_000962.3 1035791 1035810 TBseq_1.0_566_LEFT 2 + ACTCGTTCTACCGGCCGAA

NC_000962.3 1037841 1037861 TBseq_1.0_566_RIGHT 2 - GTGGCGACCACGATGATCAG

NC_000962.3 1037651 1037672 TBseq_1.0_567_LEFT 1 + AGGCATCTTGTTGTCCATCGC

NC_000962.3 1039655 1039675 TBseq_1.0_567_RIGHT 1 - CTACGGCGAGATCAACGGAC

NC_000962.3 1039442 1039464 TBseq_1.0_568_LEFT 2 + CGAAATCGACCAGATAGGCGAA

NC_000962.3 1041426 1041446 TBseq_1.0_568_RIGHT 2 - CGTCTTACCTGAACCGGTCG

NC_000962.3 1041229 1041251 TBseq_1.0_569_LEFT 1 + GAGCATCCCAAGGAGCATGTAG

NC_000962.3 1043192 1043213 TBseq_1.0_569_RIGHT 1 - CGTCAGACAACTTCACCACCG

NC_000962.3 1043038 1043062 TBseq_1.0_570_LEFT 2 + TACCCGATCATCAACTACGAGTAC

NC_000962.3 1045017 1045034 TBseq_1.0_570_RIGHT 2 - TCGGCGGCGAATTCGAC

NC_000962.3 1044801 1044823 TBseq_1.0_571_LEFT 1 + GATGAGCATTCCCTACATCGCC

NC_000962.3 1046803 1046823 TBseq_1.0_571_RIGHT 1 - GGCTCCAGTCCACAAACACC

NC_000962.3 1046595 1046614 TBseq_1.0_572_LEFT 2 + GCGCGGTTCGTGATCTTCT

NC_000962.3 1048645 1048665 TBseq_1.0_572_RIGHT 2 - CGCATGTGGTCCAGAAAGCA

NC_000962.3 1048418 1048439 TBseq_1.0_573_LEFT 1 + GGGTGACGTATCGAAGTGACC

NC_000962.3 1050399 1050419 TBseq_1.0_573_RIGHT 1 - CTTTGTCGCTCGTGGACAGG

NC_000962.3 1050217 1050237 TBseq_1.0_574_LEFT 2 + CTGGACTCCGAACGGGTAGT

NC_000962.3 1052211 1052229 TBseq_1.0_574_RIGHT 2 - ACGGTGCGCTTGACTCCA

NC_000962.3 1051973 1051992 TBseq_1.0_575_LEFT 1 + GGGTACACCGGCATGAAGC

NC_000962.3 1053965 1053984 TBseq_1.0_575_RIGHT 1 - GCTGTGCGCCGGATAACTT

NC_000962.3 1053479 1053499 TBseq_1.0_576_LEFT 2 + GTCCACAGTGGTACTCCGGT

NC_000962.3 1055467 1055487 TBseq_1.0_576_RIGHT 2 - TGGTGCCAGCCGATTTCATC

NC_000962.3 1055265 1055285 TBseq_1.0_577_LEFT 1 + CCAGAATTGAGGTGGACGGC

NC_000962.3 1057316 1057336 TBseq_1.0_577_RIGHT 1 - CAACGGCCGGGATATACACC

NC_000962.3 1057142 1057161 TBseq_1.0_578_LEFT 2 + ACCACGCCATTTCCACTCG

NC_000962.3 1059131 1059151 TBseq_1.0_578_RIGHT 2 - CGGGGTAGTCACGTTCGAAG

NC_000962.3 1058984 1059003 TBseq_1.0_579_LEFT 1 + CACCAACCACGCCCAGTAC

NC_000962.3 1060965 1060985 TBseq_1.0_579_RIGHT 1 - GGTATCGACCTGGCTAACGC

NC_000962.3 1060814 1060833 TBseq_1.0_580_LEFT 2 + CTCACCGACGCTGACCAAC

NC_000962.3 1062769 1062789 TBseq_1.0_580_RIGHT 2 - CGTGCTTCTCACCGGCATAC

NC_000962.3 1062559 1062579 TBseq_1.0_581_LEFT 1 + CAAGATCCTCGCGCTGGATG

NC_000962.3 1064491 1064511 TBseq_1.0_581_RIGHT 1 - CCCAAGAGGAGGCCTGCTAT

NC_000962.3 1064342 1064361 TBseq_1.0_582_LEFT 2 + TCGCGTGGAGTGGTGATCC

NC_000962.3 1066319 1066337 TBseq_1.0_582_RIGHT 2 - AACAGGACCGGCAACAGC

NC_000962.3 1066135 1066153 TBseq_1.0_583_LEFT 1 + ACCTCGTCAGGGTTGCGT

NC_000962.3 1068161 1068179 TBseq_1.0_583_RIGHT 1 - TCGCTGTTCGTCCGACCA

NC_000962.3 1067991 1068011 TBseq_1.0_584_LEFT 2 + TACGGTGCACCTGGTAGACG

NC_000962.3 1069948 1069969 TBseq_1.0_584_RIGHT 2 - TTCCCGGATTTCCTGCTTGAC

NC_000962.3 1069793 1069814 TBseq_1.0_585_LEFT 1 + GTGAAATCCACGACGTGACAC

NC_000962.3 1071731 1071755 TBseq_1.0_585_RIGHT 1 - CTTGATCTGCTCATACTTTTCGCG

NC_000962.3 1071558 1071579 TBseq_1.0_586_LEFT 2 + TGCAGGAGATCAAGAAGCTGC

NC_000962.3 1073578 1073597 TBseq_1.0_586_RIGHT 2 - TGTCCATCGTTGAGCAGCG

NC_000962.3 1073389 1073408 TBseq_1.0_587_LEFT 1 + GAACTCGCCAAGACGTCGG

NC_000962.3 1075341 1075360 TBseq_1.0_587_RIGHT 1 - GGTCAACACCGGTTTGGCG

NC_000962.3 1075187 1075207 TBseq_1.0_588_LEFT 2 + CAGGGGGTTTGATGAGGTCG

NC_000962.3 1077124 1077144 TBseq_1.0_588_RIGHT 2 - GGTTCGTGTGGAAGCGAGTT

NC_000962.3 1076768 1076790 TBseq_1.0_589_LEFT 1 + CGCTCAATGTCAGCTAACGTCA

NC_000962.3 1078790 1078810 TBseq_1.0_589_RIGHT 1 - GAGACCGTCTCCTCAATCGC

NC_000962.3 1078577 1078598 TBseq_1.0_590_LEFT 2 + AGCAAGCTCGACTGATGTTCG

NC_000962.3 1080613 1080632 TBseq_1.0_590_RIGHT 2 - CTCGATGCCGACCTGCTTG

NC_000962.3 1080391 1080411 TBseq_1.0_591_LEFT 1 + ACATTTGGTTCTGCCCGACC

NC_000962.3 1082458 1082480 TBseq_1.0_591_RIGHT 1 - GGTCAGCCAACTACATCAAGGG

NC_000962.3 1082300 1082319 TBseq_1.0_592_LEFT 2 + AACAGCGCCGTCATTTCCC

NC_000962.3 1084192 1084214 TBseq_1.0_592_RIGHT 2 - GTCGAATACCGGTTCACCAGAA

NC_000962.3 1084014 1084032 TBseq_1.0_593_LEFT 1 + TCGGGGAAGCGTGACAGT

NC_000962.3 1085906 1085925 TBseq_1.0_593_RIGHT 1 - TCGAGGGCCAGATCGAAGC

NC_000962.3 1085736 1085758 TBseq_1.0_594_LEFT 2 + CGAGTGATTCCCATCAGCATCA

NC_000962.3 1087654 1087674 TBseq_1.0_594_RIGHT 2 - CTGATTTCACGCCAGTCGGT

NC_000962.3 1087428 1087447 TBseq_1.0_595_LEFT 1 + GTCCCGGTATTGGCGTTCG

NC_000962.3 1089452 1089471 TBseq_1.0_595_RIGHT 1 - CAGCGTCGACACCATCACC

NC_000962.3 1088387 1088406 TBseq_1.0_596_LEFT 2 + ACTCGCCGATCCTTTCCCA

NC_000962.3 1090311 1090333 TBseq_1.0_596_RIGHT 2 - CGTAGCGAAACGGACTCTACTC

NC_000962.3 1093148 1093167 TBseq_1.0_597_LEFT 1 + CCCTCCCTGGCTCGTGATA

NC_000962.3 1095040 1095061 TBseq_1.0_597_RIGHT 1 - CGTCAGCGCTTATCGAAATCG

NC_000962.3 1094892 1094911 TBseq_1.0_598_LEFT 2 + TGCCCAAGCGCAGAAAGTC

NC_000962.3 1096810 1096829 TBseq_1.0_598_RIGHT 2 - ATTCGCACGGACACCAGTG

NC_000962.3 1096599 1096618 TBseq_1.0_599_LEFT 1 + ACACTGCACCTGGGGTTCT

NC_000962.3 1098632 1098651 TBseq_1.0_599_RIGHT 1 - TCAGCCTGACACCCACGTG

NC_000962.3 1098435 1098454 TBseq_1.0_600_LEFT 2 + CCGGAGAAGTGGTGCACGA

NC_000962.3 1100450 1100470 TBseq_1.0_600_RIGHT 2 - GCGACCTTCATCACTGCTCC

NC_000962.3 1100290 1100309 TBseq_1.0_601_LEFT 1 + CGAAGGGCGTCGTTGTCAC

NC_000962.3 1102296 1102317 TBseq_1.0_601_RIGHT 1 - TCATCGGCTAACACCAGCATG

NC_000962.3 1102150 1102172 TBseq_1.0_602_LEFT 2 + GGAGTTTCTCAGAGGAAAGCGG

NC_000962.3 1104114 1104134 TBseq_1.0_602_RIGHT 2 - GGTCGGCGGAGACACATAGA

NC_000962.3 1103935 1103955 TBseq_1.0_603_LEFT 1 + GCTGCTCGGTCCAATAGGTC

NC_000962.3 1105881 1105901 TBseq_1.0_603_RIGHT 1 - GAGCCCGAACCAATCCCAAC

NC_000962.3 1105737 1105757 TBseq_1.0_604_LEFT 2 + CGGGAAACGCGTCCAACTAC

NC_000962.3 1107674 1107692 TBseq_1.0_604_RIGHT 2 - GGCTGACAGCGCTTTGGT

NC_000962.3 1107451 1107470 TBseq_1.0_605_LEFT 1 + GGTCAGCGTCACCGTCTGA

NC_000962.3 1109436 1109456 TBseq_1.0_605_RIGHT 1 - GTGACGATCACCAGCCGTTC

NC_000962.3 1109249 1109269 TBseq_1.0_606_LEFT 2 + GCTGCGGGTTATCGTGTGAA

NC_000962.3 1111228 1111250 TBseq_1.0_606_RIGHT 2 - GAACCATCACCTCGAAGACCAC

NC_000962.3 1110997 1111017 TBseq_1.0_607_LEFT 1 + ACGGAACTTGGCGAAATCGT

NC_000962.3 1113063 1113082 TBseq_1.0_607_RIGHT 1 - GAAGGTGTACTTGCGGGCG

NC_000962.3 1112866 1112885 TBseq_1.0_608_LEFT 2 + GATGCCGAAGCTGCTGACG

NC_000962.3 1114823 1114843 TBseq_1.0_608_RIGHT 2 - CAATGACACCAGACCCTCGG

NC_000962.3 1114472 1114489 TBseq_1.0_609_LEFT 1 + TGGCCCACGGTTGTCCA

NC_000962.3 1116432 1116452 TBseq_1.0_609_RIGHT 1 - CGTCCAGGGATTACCAGTGG

NC_000962.3 1116228 1116248 TBseq_1.0_610_LEFT 2 + GGGAAAGACTGCAGCAAGGT

NC_000962.3 1118258 1118278 TBseq_1.0_610_RIGHT 2 - TTGGTCTGTACGTTGCGCTC

NC_000962.3 1118054 1118073 TBseq_1.0_611_LEFT 1 + CTCGAGGCGTTCACGATCC

NC_000962.3 1120015 1120035 TBseq_1.0_611_RIGHT 1 - CGACCAGAGGACATGACGGA

NC_000962.3 1119712 1119732 TBseq_1.0_612_LEFT 2 + GGGTTGTCTTCCACCCCATG

NC_000962.3 1121639 1121658 TBseq_1.0_612_RIGHT 2 - TACCCGACCCAATCACCGG

NC_000962.3 1121503 1121523 TBseq_1.0_613_LEFT 1 + CAGGATGCCGGAAGACTCCA

NC_000962.3 1123489 1123507 TBseq_1.0_613_RIGHT 1 - TGCGAATCGACCGACTCG

NC_000962.3 1123251 1123270 TBseq_1.0_614_LEFT 2 + GGGTAGGACAGGTAGCCGA

NC_000962.3 1125289 1125308 TBseq_1.0_614_RIGHT 2 - TTCACCACATGGGAGCCGT

NC_000962.3 1125030 1125048 TBseq_1.0_615_LEFT 1 + TGGACCAACTACGGCGCT

NC_000962.3 1126965 1126984 TBseq_1.0_615_RIGHT 1 - CCACCGCGATCGCATATCC

NC_000962.3 1126743 1126763 TBseq_1.0_616_LEFT 2 + GAGATGTTCAGCGCCTCCTG

NC_000962.3 1128814 1128832 TBseq_1.0_616_RIGHT 2 - TCTCGACGCCGTTGACCT

NC_000962.3 1128247 1128266 TBseq_1.0_617_LEFT 1 + GGTTCTCAGTCGACGACCG

NC_000962.3 1130138 1130157 TBseq_1.0_617_RIGHT 1 - CACCGCAGCAGACTATCGC

NC_000962.3 1129925 1129944 TBseq_1.0_618_LEFT 2 + GGAAGCGAGTCGGCGAATC

NC_000962.3 1131975 1131994 TBseq_1.0_618_RIGHT 2 - TGGGAATGGCCACGAGGAA

NC_000962.3 1131781 1131801 TBseq_1.0_619_LEFT 1 + TTGGTGACGTTGTTGGGGTG

NC_000962.3 1133783 1133803 TBseq_1.0_619_RIGHT 1 - CTAGGCGCGAAGTTCAAGGC

NC_000962.3 1133602 1133622 TBseq_1.0_620_LEFT 2 + GCCGAACTCGAGGTCAAGGT

NC_000962.3 1135542 1135561 TBseq_1.0_620_RIGHT 2 - CACAATCCGGGCGGTCTTC

NC_000962.3 1135330 1135350 TBseq_1.0_621_LEFT 1 + TGGGCTCGTGGACAAAATCG

NC_000962.3 1137329 1137349 TBseq_1.0_621_RIGHT 1 - GTCAACAATCGCGTCCAGCT

NC_000962.3 1137032 1137051 TBseq_1.0_622_LEFT 2 + AGGTAGGTGAAGGGCCCGA

NC_000962.3 1139026 1139043 TBseq_1.0_622_RIGHT 2 - AATGTCGGCGCGCTCAG

NC_000962.3 1138738 1138756 TBseq_1.0_623_LEFT 1 + ACCTGGCACCGCTGTATG

NC_000962.3 1140661 1140683 TBseq_1.0_623_RIGHT 1 - TCGGGACATAGAGCTTGTCAGT

NC_000962.3 1140487 1140504 TBseq_1.0_624_LEFT 2 + AAGCGGCGCAACATCGT

NC_000962.3 1142427 1142448 TBseq_1.0_624_RIGHT 2 - CCGGGATACATTCGCTTCAGC

NC_000962.3 1142021 1142041 TBseq_1.0_625_LEFT 1 + GATTCGACCTGTACGTGCGG

NC_000962.3 1144009 1144030 TBseq_1.0_625_RIGHT 1 - TCCACGCAAGATGACACTTGG

NC_000962.3 1143817 1143838 TBseq_1.0_626_LEFT 2 + TGAGCCTTTTCATCACCGACG

NC_000962.3 1145818 1145837 TBseq_1.0_626_RIGHT 2 - GCGAACCGAGGAAATGCCA

NC_000962.3 1145659 1145678 TBseq_1.0_627_LEFT 1 + GCGAGACGGAGGACACCAT

NC_000962.3 1147572 1147591 TBseq_1.0_627_RIGHT 1 - CGTGGACACCTCCTGCAAC

NC_000962.3 1147389 1147408 TBseq_1.0_628_LEFT 2 + CGAGCTCTCCTTCCGTGGA

NC_000962.3 1149393 1149412 TBseq_1.0_628_RIGHT 2 - AACCTGATCGACAACGCGC

NC_000962.3 1149186 1149205 TBseq_1.0_629_LEFT 1 + CCATAGCCTCGACGAAGCC

NC_000962.3 1151220 1151240 TBseq_1.0_629_RIGHT 1 - GCACTTGGAGGGCCTAAACG

NC_000962.3 1151044 1151064 TBseq_1.0_630_LEFT 2 + GAAGTAGTTCGACAGCGCGG

NC_000962.3 1153042 1153059 TBseq_1.0_630_RIGHT 2 - ACGCCGCCCATAACCCA

NC_000962.3 1152776 1152798 TBseq_1.0_631_LEFT 1 + GGCAATTTCGTCGAAAACTGGG

NC_000962.3 1154683 1154702 TBseq_1.0_631_RIGHT 1 - CGGTCTTGTCCAGCAGCAG

NC_000962.3 1154488 1154505 TBseq_1.0_632_LEFT 2 + CTGCAGCCGTTCGCCAT

NC_000962.3 1156444 1156463 TBseq_1.0_632_RIGHT 2 - TCCCGATGATCGAGCAGCA

NC_000962.3 1156254 1156273 TBseq_1.0_633_LEFT 1 + GTGGTCAATGCCAAGCTGC

NC_000962.3 1158183 1158202 TBseq_1.0_633_RIGHT 1 - GGTACGCCGATCTCGCTCT

NC_000962.3 1157744 1157766 TBseq_1.0_634_LEFT 2 + TGTAAGAGTGGTTCAACGCGTG

NC_000962.3 1159739 1159756 TBseq_1.0_634_RIGHT 2 - GAAACTGCGCTCGCGTG

NC_000962.3 1159374 1159393 TBseq_1.0_635_LEFT 1 + TTACCGAGCCGACCCTTGG

NC_000962.3 1161274 1161294 TBseq_1.0_635_RIGHT 1 - CGGACAAAGGGGGACATCTC

NC_000962.3 1162515 1162532 TBseq_1.0_636_LEFT 2 + TTGGTTGAGCGTGGCCG

NC_000962.3 1164466 1164485 TBseq_1.0_636_RIGHT 2 - TTGATGCCCGAGGTTGTGC

NC_000962.3 1164203 1164220 TBseq_1.0_637_LEFT 1 + AGCGTGAACCCGAGGCT

NC_000962.3 1166277 1166298 TBseq_1.0_637_RIGHT 1 - CAGGTCTGTCCGTCTATCCGT

NC_000962.3 1166113 1166133 TBseq_1.0_638_LEFT 2 + GTAGACCTCATCCGCCCAGT

NC_000962.3 1168030 1168049 TBseq_1.0_638_RIGHT 2 - GGCTTGACCGGCATACCAG

NC_000962.3 1167840 1167859 TBseq_1.0_639_LEFT 1 + CTGCTGTTGGTCAAAGGCG

NC_000962.3 1169784 1169803 TBseq_1.0_639_RIGHT 1 - AAGTGTTTCGACCAGGCGC

NC_000962.3 1169591 1169610 TBseq_1.0_640_LEFT 2 + CCAATCAGCGCAACGGCTA

NC_000962.3 1171596 1171616 TBseq_1.0_640_RIGHT 2 - GTTGTAGAGGCCACCGGTCT

NC_000962.3 1171412 1171432 TBseq_1.0_641_LEFT 1 + CCACAGCACACCAGTCGAAA

NC_000962.3 1173341 1173360 TBseq_1.0_641_RIGHT 1 - GGAGGAGTAGCCGGCGAAT

NC_000962.3 1173185 1173205 TBseq_1.0_642_LEFT 2 + CGCAGAGACCTTCCTTGACG

NC_000962.3 1175156 1175178 TBseq_1.0_642_RIGHT 2 - GGGCCACATTTTAGATCGGACG

NC_000962.3 1174993 1175010 TBseq_1.0_643_LEFT 1 + ATCAGCCGGGCGGGTTT

NC_000962.3 1176974 1176993 TBseq_1.0_643_RIGHT 1 - AGGCACTGGTACGTGACGA

NC_000962.3 1176766 1176786 TBseq_1.0_644_LEFT 2 + CGAACTGTTGATGCTCGCCA

NC_000962.3 1178744 1178763 TBseq_1.0_644_RIGHT 2 - CGAGTGCCTAGCCATGGTC

NC_000962.3 1178497 1178519 TBseq_1.0_645_LEFT 1 + GGCTGTGCCCAATCTAGATTCC

NC_000962.3 1180524 1180545 TBseq_1.0_645_RIGHT 1 - CAGGCGAGAACCACTGCATAG

NC_000962.3 1180052 1180072 TBseq_1.0_646_LEFT 2 + CAGAAGAGCTCCAAACCCCG

NC_000962.3 1182125 1182144 TBseq_1.0_646_RIGHT 2 - ACTAACGGTGGCCCCTTCC

NC_000962.3 1181941 1181961 TBseq_1.0_647_LEFT 1 + ACGAGCAAGGCTTCATCACC

NC_000962.3 1183965 1183984 TBseq_1.0_647_RIGHT 1 - CGCTCAGCCCACCAACAAA

NC_000962.3 1183804 1183823 TBseq_1.0_648_LEFT 2 + TTGGTGGAGGGCAAGTGGA

NC_000962.3 1185732 1185752 TBseq_1.0_648_RIGHT 2 - ATCGAGGGCTGATCAAACGC

NC_000962.3 1185465 1185484 TBseq_1.0_649_LEFT 1 + CCAGCTCGGTCAACCTTGG

NC_000962.3 1187441 1187460 TBseq_1.0_649_RIGHT 1 - GTTGGCGTGACAAGAGGCA

NC_000962.3 1186348 1186367 TBseq_1.0_650_LEFT 2 + CTTCGTGTGGGGCGATCAC

NC_000962.3 1188322 1188341 TBseq_1.0_650_RIGHT 2 - GCACGATAGCCACCGACGA

NC_000962.3 1188381 1188400 TBseq_1.0_651_LEFT 1 + GTGGGCGGGTAGTTTGGCT

NC_000962.3 1190424 1190446 TBseq_1.0_651_RIGHT 1 - AGCAAATCAGGGAGAGGAAACC

NC_000962.3 1190434 1190459 TBseq_1.0_652_LEFT 2 + CCCTGATTTGCTGATATGTAGTTGC

NC_000962.3 1192347 1192369 TBseq_1.0_652_RIGHT 2 - GACACAGTCGACGTCATGAAAC

NC_000962.3 1192199 1192219 TBseq_1.0_653_LEFT 1 + GTGATGGCGCGAGTCTAAGC

NC_000962.3 1194116 1194136 TBseq_1.0_653_RIGHT 1 - CTACACATTCACCGGCACCG

NC_000962.3 1193974 1193993 TBseq_1.0_654_LEFT 2 + ATGTAGCGGACCAGCCAGA

NC_000962.3 1195947 1195967 TBseq_1.0_654_RIGHT 2 - CATGAGCTGGGAGCATGAGG

NC_000962.3 1195762 1195782 TBseq_1.0_655_LEFT 1 + GTCCATCAACGCCACGTAGG

NC_000962.3 1197781 1197801 TBseq_1.0_655_RIGHT 1 - TTATCAGCAAGAGCCGCAGC

NC_000962.3 1197633 1197652 TBseq_1.0_656_LEFT 2 + GACTGGATGCGCTTGCCAA

NC_000962.3 1199525 1199545 TBseq_1.0_656_RIGHT 2 - ATTCATGAAACGCCGTCCCG

NC_000962.3 1199358 1199378 TBseq_1.0_657_LEFT 1 + GACAGCTTCTGGCATGACGG

NC_000962.3 1201278 1201300 TBseq_1.0_657_RIGHT 1 - CTGCTTGTGTTCCTTTGCTAGC

NC_000962.3 1201109 1201128 TBseq_1.0_658_LEFT 2 + ACGACTGTCACGACGGCTA

NC_000962.3 1203062 1203081 TBseq_1.0_658_RIGHT 2 - GCCCAACAAGTCGTACCGG

NC_000962.3 1202799 1202819 TBseq_1.0_659_LEFT 1 + GAGTACGGGGTGTCGCAGAT

NC_000962.3 1204799 1204819 TBseq_1.0_659_RIGHT 1 - GGTCAGGTAGGCGTCGAATG

NC_000962.3 1204483 1204502 TBseq_1.0_660_LEFT 2 + GTGGGTGCCGCGATTACTC

NC_000962.3 1206472 1206495 TBseq_1.0_660_RIGHT 2 - CTGCCCCCTTAAGCCTATAAGTG

NC_000962.3 1206286 1206306 TBseq_1.0_661_LEFT 1 + GATGACGGCAATGACGATGC

NC_000962.3 1208267 1208287 TBseq_1.0_661_RIGHT 1 - GCATTAGCGCTTCCAGCAGT

NC_000962.3 1208121 1208140 TBseq_1.0_662_LEFT 2 + GAGTTGCGCTCGATGGCTT

NC_000962.3 1210119 1210136 TBseq_1.0_662_RIGHT 2 - AGCTACACGCCGTTCGC

NC_000962.3 1209623 1209641 TBseq_1.0_663_LEFT 1 + CGAACTCGCCACCGCTTT

NC_000962.3 1211537 1211558 TBseq_1.0_663_RIGHT 1 - GCCTTACTCCGATCAGTCCAG

NC_000962.3 1213916 1213935 TBseq_1.0_664_LEFT 2 + GGCGAATCCAGTCCGGTCT

NC_000962.3 1215917 1215937 TBseq_1.0_664_RIGHT 2 - GGTGTGGTCGACGAAACTCA

NC_000962.3 1214385 1214410 TBseq_1.0_665_LEFT 1 + GTGATAAAACCGTTATAGGGTCCGT

NC_000962.3 1216365 1216387 TBseq_1.0_665_RIGHT 1 - GTTTCCCATGGACAGAATGCAC

NC_000962.3 1219067 1219084 TBseq_1.0_666_LEFT 2 + GCAAACTTCGCGCACGC

NC_000962.3 1220986 1221006 TBseq_1.0_666_RIGHT 2 - CGACGCCGTAGAAGCCATTC

NC_000962.3 1220722 1220741 TBseq_1.0_667_LEFT 1 + CAAGTACGCCGAGGGACTG

NC_000962.3 1222756 1222777 TBseq_1.0_667_RIGHT 1 - GACGAATTGCTTGAGGGAGGG

NC_000962.3 1222543 1222562 TBseq_1.0_668_LEFT 2 + TTCGCCAACCTCGTTACGC

NC_000962.3 1224575 1224594 TBseq_1.0_668_RIGHT 2 - GTCGGTCAGGATGTGCAGC

NC_000962.3 1224369 1224389 TBseq_1.0_669_LEFT 1 + CGGTAGACTTCCTGGGTGCC

NC_000962.3 1226420 1226440 TBseq_1.0_669_RIGHT 1 - CTGACCAATGTGTCACGGCT

NC_000962.3 1226256 1226276 TBseq_1.0_670_LEFT 2 + GGCGAATCGTTTTGCGTTCC

NC_000962.3 1228158 1228178 TBseq_1.0_670_RIGHT 2 - GGAAAACATCCGAGCGGTCG

NC_000962.3 1227787 1227806 TBseq_1.0_671_LEFT 1 + AGTGGCGCAGAAGAAGACG

NC_000962.3 1229831 1229851 TBseq_1.0_671_RIGHT 1 - ACATCGCCGGATTTCACCAC

NC_000962.3 1229678 1229698 TBseq_1.0_672_LEFT 2 + GGTAACCACCAGCAGATGCC

NC_000962.3 1231659 1231679 TBseq_1.0_672_RIGHT 2 - CCAACCTGCGGCCGTTATAG

NC_000962.3 1231508 1231528 TBseq_1.0_673_LEFT 1 + ACATGCCGCTCGATTTAGGG

NC_000962.3 1233479 1233500 TBseq_1.0_673_RIGHT 1 - CGGTTCAACGACCTCTACACC

NC_000962.3 1233343 1233364 TBseq_1.0_674_LEFT 2 + GCACACTTTCGAACAGCTTGC

NC_000962.3 1235296 1235316 TBseq_1.0_674_RIGHT 2 - ATGGTATTGCGCGATCCTGC

NC_000962.3 1235044 1235061 TBseq_1.0_675_LEFT 1 + TACGGGATCGGCCGCTT

NC_000962.3 1237121 1237139 TBseq_1.0_675_RIGHT 1 - CCGTTGTCACCGGTTGCA

NC_000962.3 1236884 1236903 TBseq_1.0_676_LEFT 2 + CCGAGTGCGAGCTGGTCAT

NC_000962.3 1238950 1238970 TBseq_1.0_676_RIGHT 2 - GTCAACTCCGACTCGATGGC

NC_000962.3 1238697 1238715 TBseq_1.0_677_LEFT 1 + GAAGGAAGCGCGCACCAA

NC_000962.3 1240719 1240740 TBseq_1.0_677_RIGHT 1 - CACAGGTTATGTCTCGGGGTC

NC_000962.3 1240519 1240539 TBseq_1.0_678_LEFT 2 + CCCGTATCCAGTGGTGAAGG

NC_000962.3 1242483 1242503 TBseq_1.0_678_RIGHT 2 - TGATCTGGCATGCCGAACTG

NC_000962.3 1242328 1242347 TBseq_1.0_679_LEFT 1 + TTGTCCTCCTGGTCGCGAT

NC_000962.3 1244239 1244259 TBseq_1.0_679_RIGHT 1 - TCAGGTACTCCAGCTCGACG

NC_000962.3 1244020 1244040 TBseq_1.0_680_LEFT 2 + CTGTCGGCCGCTGTATTACC

NC_000962.3 1245936 1245958 TBseq_1.0_680_RIGHT 2 - GAGAATTCCGCTAGGTCAGGTG

NC_000962.3 1245687 1245710 TBseq_1.0_681_LEFT 1 + ACTTCGTGAAGATGGTCCACAAC

NC_000962.3 1247719 1247737 TBseq_1.0_681_RIGHT 1 - TGATAGCCCGGCGACCAA

NC_000962.3 1247518 1247536 TBseq_1.0_682_LEFT 2 + CGACAGCCCGTTTGCCAA

NC_000962.3 1249457 1249475 TBseq_1.0_682_RIGHT 2 - ACAAGGTCAAAGCGGGCG

NC_000962.3 1249317 1249340 TBseq_1.0_683_LEFT 1 + CTGCAGTGAAAACTACTGGGCAT

NC_000962.3 1251304 1251323 TBseq_1.0_683_RIGHT 1 - TACCCACGCGAGATCCTGG

NC_000962.3 1251093 1251112 TBseq_1.0_684_LEFT 2 + CTGACCAGCAGCGGATTCG

NC_000962.3 1253142 1253161 TBseq_1.0_684_RIGHT 2 - TGCGTTCCCCTATCTCGGT

NC_000962.3 1253000 1253019 TBseq_1.0_685_LEFT 1 + ACAATGCTTGCCCAGAGCC

NC_000962.3 1254986 1255007 TBseq_1.0_685_RIGHT 1 - CACCAGTGGGGGTATGTTGTC

NC_000962.3 1254763 1254782 TBseq_1.0_686_LEFT 2 + CCGCATCATCGACAACGCT

NC_000962.3 1256783 1256803 TBseq_1.0_686_RIGHT 2 - GGATTGGGTCGAGGTCACCA

NC_000962.3 1256590 1256609 TBseq_1.0_687_LEFT 1 + ATCGTGGCGATCGACATGC

NC_000962.3 1258545 1258564 TBseq_1.0_687_RIGHT 1 - GCTTGATGCACCACAGCGT

NC_000962.3 1258216 1258237 TBseq_1.0_688_LEFT 2 + TTATCGAGATGTGTGTGCCCG

NC_000962.3 1260143 1260161 TBseq_1.0_688_RIGHT 2 - AATGGGCAAATCCGGGCG

NC_000962.3 1259797 1259814 TBseq_1.0_689_LEFT 1 + ACTGCACCCAGCCGTTC

NC_000962.3 1261868 1261888 TBseq_1.0_689_RIGHT 1 - GTCTCTGGTGCAGGTGAGTG

NC_000962.3 1262137 1262157 TBseq_1.0_690_LEFT 2 + ACGACGAGGATGACGCTGTA

NC_000962.3 1264180 1264199 TBseq_1.0_690_RIGHT 2 - TCAGCCAGCGGAGGTTTGT

NC_000962.3 1264274 1264293 TBseq_1.0_691_LEFT 1 + CCTCCCACCACCAACACTG

NC_000962.3 1266258 1266280 TBseq_1.0_691_RIGHT 1 - ATGGGATTGCCTACGTTGGTGA

NC_000962.3 1266114 1266135 TBseq_1.0_692_LEFT 2 + GCGTCTTGCTGAATAGTGGCT

NC_000962.3 1268081 1268099 TBseq_1.0_692_RIGHT 2 - CTCCAACGCACACACCGT

NC_000962.3 1267911 1267935 TBseq_1.0_693_LEFT 1 + TCCTGCTGATCACCTTGATTAACG

NC_000962.3 1269929 1269949 TBseq_1.0_693_RIGHT 1 - TACCGGATCGACACTCGGAC

NC_000962.3 1269712 1269732 TBseq_1.0_694_LEFT 2 + CCACACATCGTCAACGCTGA

NC_000962.3 1271639 1271659 TBseq_1.0_694_RIGHT 2 - CAGGGTCATGCCAACTACGC

NC_000962.3 1271465 1271487 TBseq_1.0_695_LEFT 1 + GCAAGATCGTGGACATCAACCT

NC_000962.3 1273405 1273426 TBseq_1.0_695_RIGHT 1 - AGGACCTAGCAACACAATCGC

NC_000962.3 1273225 1273244 TBseq_1.0_696_LEFT 2 + CGAAGCACTGATCCGGACC

NC_000962.3 1275177 1275196 TBseq_1.0_696_RIGHT 2 - GACCGTCTTGTCCGTCACG

NC_000962.3 1274190 1274210 TBseq_1.0_697_LEFT 1 + CCAGGAAGGCCATTTCGGTG

NC_000962.3 1276122 1276146 TBseq_1.0_697_RIGHT 1 - CGGACTTGATTTCTTCGCTATTGG

NC_000962.3 1275944 1275965 TBseq_1.0_698_LEFT 2 + CGAAGGACCAGGTCACCAGTA

NC_000962.3 1277868 1277888 TBseq_1.0_698_RIGHT 2 - CAATCCTCGCTGGCCAGTAC

NC_000962.3 1277727 1277747 TBseq_1.0_699_LEFT 1 + GAGGCAGAACGTTTCGCTCA

NC_000962.3 1279656 1279676 TBseq_1.0_699_RIGHT 1 - GTAACCAATCCCGCAGCTCC

NC_000962.3 1279450 1279471 TBseq_1.0_700_LEFT 2 + CGCCATAGGTACCATCCCCAG

NC_000962.3 1281448 1281468 TBseq_1.0_700_RIGHT 2 - TTACGGCCAACAGCTTGTCG

NC_000962.3 1281196 1281215 TBseq_1.0_701_LEFT 1 + GGTCTTCTCGCGCACTTCC

NC_000962.3 1283135 1283155 TBseq_1.0_701_RIGHT 1 - CGGTTCAGGCAGCATCATGT

NC_000962.3 1282862 1282881 TBseq_1.0_702_LEFT 2 + GGCCAAGGCAGAGGGAAAG

NC_000962.3 1284861 1284881 TBseq_1.0_702_RIGHT 2 - CCGCTAACCAGGGAGAGACA

NC_000962.3 1284710 1284727 TBseq_1.0_703_LEFT 1 + TTGGCGGCCGAGCTGAT

NC_000962.3 1286679 1286698 TBseq_1.0_703_RIGHT 1 - GGAAGTTCCCAACGACCCG

NC_000962.3 1286480 1286501 TBseq_1.0_704_LEFT 2 + TTGACCTACGCAGGACACCAC

NC_000962.3 1288515 1288536 TBseq_1.0_704_RIGHT 2 - GAAACGGAATCCCAGTGAGCC

NC_000962.3 1288289 1288310 TBseq_1.0_705_LEFT 1 + CCACGTGATCCTGTCGGAATG

NC_000962.3 1290227 1290247 TBseq_1.0_705_RIGHT 1 - TCTCCAGCTCGAGGAATCCC

NC_000962.3 1290000 1290020 TBseq_1.0_706_LEFT 2 + GCGATCTACGACAAGTGGCT

NC_000962.3 1292053 1292074 TBseq_1.0_706_RIGHT 2 - GTGTTGATCAACGCGTACACC

NC_000962.3 1291787 1291807 TBseq_1.0_707_LEFT 1 + GCCGAAAAGTGCACCCTGTG

NC_000962.3 1293788 1293810 TBseq_1.0_707_RIGHT 1 - CACCAGGTACATCAGCTTGTCA

NC_000962.3 1293310 1293329 TBseq_1.0_708_LEFT 2 + CAACGCCTAGCTCAGTCGG

NC_000962.3 1295249 1295268 TBseq_1.0_708_RIGHT 2 - ACGAATCGACACGTTGCCC

NC_000962.3 1295067 1295088 TBseq_1.0_709_LEFT 1 + GAGATCATGATCGGCGACACG

NC_000962.3 1297086 1297106 TBseq_1.0_709_RIGHT 1 - CCAGCTTGGGAACGTTTGCC

NC_000962.3 1296691 1296710 TBseq_1.0_710_LEFT 2 + GTTACGTTCGCACAGCCCT

NC_000962.3 1298608 1298627 TBseq_1.0_710_RIGHT 2 - GCTCTACGATGCGACGCTG

NC_000962.3 1298422 1298442 TBseq_1.0_711_LEFT 1 + CAGATTCTCCCTTGCCGACC

NC_000962.3 1300431 1300451 TBseq_1.0_711_RIGHT 1 - CAATGACCTCGCCCTCCTCA

NC_000962.3 1298744 1298764 TBseq_1.0_712_LEFT 2 + ATCAGGTGAGGCCCTTTTGC

NC_000962.3 1300795 1300814 TBseq_1.0_712_RIGHT 2 - GGTGATCTGCGGTACCGGA

NC_000962.3 1300900 1300919 TBseq_1.0_713_LEFT 1 + GACGATCTGCGACCCGAAT

NC_000962.3 1302872 1302891 TBseq_1.0_713_RIGHT 1 - CCCCACCAGGTGAGATGCT

NC_000962.3 1302722 1302741 TBseq_1.0_714_LEFT 2 + GGGGCTTGTTCAGACGCTG

NC_000962.3 1304719 1304739 TBseq_1.0_714_RIGHT 2 - GCCCTGCATGCATACTTCGG

NC_000962.3 1304279 1304298 TBseq_1.0_715_LEFT 1 + CGCAGCGATCGACACTCAG

NC_000962.3 1306311 1306333 TBseq_1.0_715_RIGHT 1 - GTGCGAGATCTGGAGAGTGAAC

NC_000962.3 1306115 1306135 TBseq_1.0_716_LEFT 2 + CTGGTTATCGGGCGACACTG

NC_000962.3 1308125 1308144 TBseq_1.0_716_RIGHT 2 - TGCACACCGGACTGGAAGA

NC_000962.3 1307922 1307942 TBseq_1.0_717_LEFT 1 + GCAGGATCTTTGCACCCGAA

NC_000962.3 1309912 1309931 TBseq_1.0_717_RIGHT 1 - CCAGGTAGCACTCGTCGGA

NC_000962.3 1309708 1309733 TBseq_1.0_718_LEFT 2 + GAATTGGCATATCCGACTTATGACG

NC_000962.3 1311660 1311680 TBseq_1.0_718_RIGHT 2 - CACCCGTTCGCTACTAGGTG

NC_000962.3 1311515 1311535 TBseq_1.0_719_LEFT 1 + CAGCAGACCGACCAGTTGTC

NC_000962.3 1313470 1313490 TBseq_1.0_719_RIGHT 1 - TACCAGACGTCGTAGGCCTG

NC_000962.3 1313262 1313280 TBseq_1.0_720_LEFT 2 + CAAAGGCCCGCGATTCCA

NC_000962.3 1315293 1315313 TBseq_1.0_720_RIGHT 2 - ACTGCGGGCTGATAGGGAAT

NC_000962.3 1315098 1315118 TBseq_1.0_721_LEFT 1 + GAGCAACTACACGTCACGGC

NC_000962.3 1317040 1317059 TBseq_1.0_721_RIGHT 1 - GCCTTCTGGACCTCGGGAT

NC_000962.3 1316894 1316912 TBseq_1.0_722_LEFT 2 + AGGCCTAGTTGCGGTGCA

NC_000962.3 1318833 1318852 TBseq_1.0_722_RIGHT 2 - CCAAACGAATGGCAACGGC

NC_000962.3 1318655 1318674 TBseq_1.0_723_LEFT 1 + CACTCGGCTCGGGTTGTTC

NC_000962.3 1320653 1320673 TBseq_1.0_723_RIGHT 1 - GCAGAAGTCGTCGTAGCTGC

NC_000962.3 1320515 1320538 TBseq_1.0_724_LEFT 2 + GCGACCATTTCACATTCTTTGCA

NC_000962.3 1322406 1322426 TBseq_1.0_724_RIGHT 2 - GGCCAACCGTTGAGAACACA

NC_000962.3 1322179 1322202 TBseq_1.0_725_LEFT 1 + GGTCTAGCCGTATCCAATCAAGC

NC_000962.3 1324108 1324127 TBseq_1.0_725_RIGHT 1 - GCACCACAAACGCTAGGCC

NC_000962.3 1323878 1323898 TBseq_1.0_726_LEFT 2 + GTTACCCGGTCATGCTGAGG

NC_000962.3 1325900 1325920 TBseq_1.0_726_RIGHT 2 - GATATCACGCTCGCACGGTC

NC_000962.3 1325708 1325728 TBseq_1.0_727_LEFT 1 + CGTCCCGGCCGACATTAAAG

NC_000962.3 1327687 1327706 TBseq_1.0_727_RIGHT 1 - ATGGAACGCACGGCTTAGC

NC_000962.3 1327533 1327554 TBseq_1.0_728_LEFT 2 + CGCGATCTCCGATAACCCAAG

NC_000962.3 1329439 1329458 TBseq_1.0_728_RIGHT 2 - CGGGGATTTCGGCGCATAG

NC_000962.3 1329036 1329056 TBseq_1.0_729_LEFT 1 + GACGGGTGCTTGACGAAGAT

NC_000962.3 1331005 1331024 TBseq_1.0_729_RIGHT 1 - CCATCAGTCGACCGCCATG

NC_000962.3 1330821 1330841 TBseq_1.0_730_LEFT 2 + CGGCGGGGAACTTCTATGTC

NC_000962.3 1332764 1332781 TBseq_1.0_730_RIGHT 2 - ACTCCAGTGGCGCAGGA

NC_000962.3 1332535 1332552 TBseq_1.0_731_LEFT 1 + TCCACATGCCGGCAGCT

NC_000962.3 1334566 1334585 TBseq_1.0_731_RIGHT 1 - GCTGCTTGCGACTACCGTT

NC_000962.3 1334155 1334177 TBseq_1.0_732_LEFT 2 + CCGCATCAGTACGAAACCATCT

NC_000962.3 1336158 1336177 TBseq_1.0_732_RIGHT 2 - GGGTGAGGGCTCCGGATAA

NC_000962.3 1335680 1335699 TBseq_1.0_733_LEFT 1 + TGTTGAGCCCGCTGTTTCC

NC_000962.3 1337668 1337685 TBseq_1.0_733_RIGHT 1 - AACGGGGTGGAGGGGTT

NC_000962.3 1337387 1337408 TBseq_1.0_734_LEFT 2 + ACTCGAGTCTGCGCAGAAATG

NC_000962.3 1339324 1339346 TBseq_1.0_734_RIGHT 2 - CGTGTCCCTATTTCTCGTGGTT

NC_000962.3 1338980 1339000 TBseq_1.0_735_LEFT 1 + GCCGTTTGAGGATGGAGTGC

NC_000962.3 1340965 1340989 TBseq_1.0_735_RIGHT 1 - CGCTTGTAAAAGTATTGTGCTGCA

NC_000962.3 1340819 1340839 TBseq_1.0_736_LEFT 2 + AGACACCATGGCCCAGATGA

NC_000962.3 1342750 1342770 TBseq_1.0_736_RIGHT 2 - GGCACCTTCTGTCACTGCTC

NC_000962.3 1342570 1342591 TBseq_1.0_737_LEFT 1 + AGCTGCTCGGTGTCGATAAGA

NC_000962.3 1344593 1344611 TBseq_1.0_737_RIGHT 1 - AACTACAACGCCGGCACC

NC_000962.3 1344290 1344309 TBseq_1.0_738_LEFT 2 + GGAATTGCGGCGAAACAGC

NC_000962.3 1346186 1346203 TBseq_1.0_738_RIGHT 2 - CCCAAAGCGGCAAAGCG

NC_000962.3 1345982 1346002 TBseq_1.0_739_LEFT 1 + ATCAACTACCGCTTTGCCCC

NC_000962.3 1347887 1347906 TBseq_1.0_739_RIGHT 1 - CCGTTGTCGACTACCTCGC

NC_000962.3 1347614 1347633 TBseq_1.0_740_LEFT 2 + GCCAGTGAGGACAGCCGTA

NC_000962.3 1349642 1349662 TBseq_1.0_740_RIGHT 2 - CTGTGCTGGGTGAGTTACGC

NC_000962.3 1349388 1349405 TBseq_1.0_741_LEFT 1 + CGGATGCCGCGAGTGTT

NC_000962.3 1351361 1351381 TBseq_1.0_741_RIGHT 1 - TCGCTGAAAGTCGCACCCTT

NC_000962.3 1351139 1351159 TBseq_1.0_742_LEFT 2 + AGTCTCGATACCCGCACTGG

NC_000962.3 1353065 1353085 TBseq_1.0_742_RIGHT 2 - GTCGACTAGTGATACCGGCC

NC_000962.3 1352881 1352901 TBseq_1.0_743_LEFT 1 + GCAATCGCCCAGGTCAACTT

NC_000962.3 1354844 1354866 TBseq_1.0_743_RIGHT 1 - CGGCTTACCATCGGACAACTAC

NC_000962.3 1354640 1354660 TBseq_1.0_744_LEFT 2 + CCAATCTGGCCTGGTAACCG

NC_000962.3 1356648 1356669 TBseq_1.0_744_RIGHT 2 - CGACACCAGGTCCATATGTGC

NC_000962.3 1356417 1356439 TBseq_1.0_745_LEFT 1 + TTCGTCTCAATGGGCAACTACA

NC_000962.3 1358462 1358483 TBseq_1.0_745_RIGHT 1 - AGCTGAAGTTCTTGCGAGACG

NC_000962.3 1358258 1358277 TBseq_1.0_746_LEFT 2 + CTGCCCGAGAGCGAGTTTG

NC_000962.3 1360278 1360295 TBseq_1.0_746_RIGHT 2 - AACCTGGAGCCGTTCGC

NC_000962.3 1360089 1360108 TBseq_1.0_747_LEFT 1 + GCCGAAAACGACGAGTCCT

NC_000962.3 1362114 1362134 TBseq_1.0_747_RIGHT 1 - GCTGTGCGAAAAGGTGACCA

NC_000962.3 1361926 1361948 TBseq_1.0_748_LEFT 2 + GAGTTCTCTCAGGCTTTCGCTG

NC_000962.3 1363850 1363869 TBseq_1.0_748_RIGHT 2 - GCATCTACGCCTTGCCAGG

NC_000962.3 1363432 1363450 TBseq_1.0_749_LEFT 1 + ACTTTGCCGAGCAGACGC

NC_000962.3 1365373 1365390 TBseq_1.0_749_RIGHT 1 - GAAAACGCGCGCCGGAA

NC_000962.3 1365225 1365245 TBseq_1.0_750_LEFT 2 + CATGGTTGCCAATCGGTCCC

NC_000962.3 1367125 1367144 TBseq_1.0_750_RIGHT 2 - TCTCGTTGACCGGGATCGC

NC_000962.3 1366987 1367009 TBseq_1.0_751_LEFT 1 + GTCATTGACGCAATTCAGACCG

NC_000962.3 1368919 1368939 TBseq_1.0_751_RIGHT 1 - CGCAAGGGTTATCAGGTGCT

NC_000962.3 1368759 1368778 TBseq_1.0_752_LEFT 2 + CGCGGAAAAACCCCAGTGA

NC_000962.3 1370765 1370786 TBseq_1.0_752_RIGHT 2 - CAGAGAAATCACCTCGCGCTC

NC_000962.3 1370585 1370604 TBseq_1.0_753_LEFT 1 + CCCGATGTGCACCACCATC

NC_000962.3 1372529 1372549 TBseq_1.0_753_RIGHT 1 - GTAAGGGCGGAGTCGGAAAG

NC_000962.3 1372356 1372376 TBseq_1.0_754_LEFT 2 + ATGACCTTCACCTGGTGGGC

NC_000962.3 1374325 1374344 TBseq_1.0_754_RIGHT 2 - AGCACGCCAAGAAATCGGG

NC_000962.3 1374192 1374211 TBseq_1.0_755_LEFT 1 + TGCACTCGACCGTCCTCAG

NC_000962.3 1376146 1376166 TBseq_1.0_755_RIGHT 1 - GGGATCGGTCAACAGGGTGT

NC_000962.3 1375917 1375935 TBseq_1.0_756_LEFT 2 + AAGCGGTGCAGTGACACG

NC_000962.3 1377934 1377955 TBseq_1.0_756_RIGHT 2 - GTACAGCTTGTGGTTCCAGCC

NC_000962.3 1377732 1377752 TBseq_1.0_757_LEFT 1 + CAGGTCCCCCAATGAGCAAC

NC_000962.3 1379695 1379716 TBseq_1.0_757_RIGHT 1 - GGTTGTCGTAGCCCAAGATCG

NC_000962.3 1379522 1379541 TBseq_1.0_758_LEFT 2 + AAGAGCAGCGCAGGTTGAC

NC_000962.3 1381473 1381492 TBseq_1.0_758_RIGHT 2 - GCGATCACCCCCTGGACTT

NC_000962.3 1381333 1381353 TBseq_1.0_759_LEFT 1 + CACCCCTGAGGAGCTTTACG

NC_000962.3 1383309 1383328 TBseq_1.0_759_RIGHT 1 - AGCTCGATCGGACGGTCAG

NC_000962.3 1382823 1382843 TBseq_1.0_760_LEFT 2 + GATCCAGACGAACGCCTCCT

NC_000962.3 1384799 1384816 TBseq_1.0_760_RIGHT 2 - GCCCAACGCGATGTCCA

NC_000962.3 1384665 1384685 TBseq_1.0_761_LEFT 1 + GTTCGGGTTCCTACGGATCG

NC_000962.3 1386706 1386723 TBseq_1.0_761_RIGHT 1 - TTCGTGCCGACCCGTTG

NC_000962.3 1384962 1384982 TBseq_1.0_762_LEFT 2 + ATAATGACTGCCGCTCTGCC

NC_000962.3 1387003 1387020 TBseq_1.0_762_RIGHT 2 - AGCGCCGCGACGTAGAT

NC_000962.3 1386835 1386856 TBseq_1.0_763_LEFT 1 + TATCGGCTCAGACACTTCGGT

NC_000962.3 1388832 1388850 TBseq_1.0_763_RIGHT 1 - TAGGTTGGGCAAGCCGCT

NC_000962.3 1388629 1388649 TBseq_1.0_764_LEFT 2 + ACGTACCCTCCCTTGTCTCG

NC_000962.3 1390681 1390701 TBseq_1.0_764_RIGHT 2 - ACGTGGCGACATCTCGATGA

NC_000962.3 1390420 1390440 TBseq_1.0_765_LEFT 1 + CTTCATAGGCCATCTCCCGG

NC_000962.3 1392446 1392465 TBseq_1.0_765_RIGHT 1 - CAATCTCGGCCTGGCGATC

NC_000962.3 1392250 1392268 TBseq_1.0_766_LEFT 2 + AATGCACGGTGCCGATGG

NC_000962.3 1394289 1394309 TBseq_1.0_766_RIGHT 2 - GTCGGATTCGCGATGGCTAC

NC_000962.3 1394138 1394157 TBseq_1.0_767_LEFT 1 + GACCCAATGCCACCGCTAG

NC_000962.3 1396039 1396058 TBseq_1.0_767_RIGHT 1 - CAAATTCCAGGGCGGGCAG

NC_000962.3 1395882 1395905 TBseq_1.0_768_LEFT 2 + GCAGATACTGAGTCAACAGCTCC

NC_000962.3 1397785 1397807 TBseq_1.0_768_RIGHT 2 - CGTGCTCAAAGAGATCGAAGGC

NC_000962.3 1397573 1397590 TBseq_1.0_769_LEFT 1 + AGCAAGGCGACCGCTGT

NC_000962.3 1399624 1399644 TBseq_1.0_769_RIGHT 1 - AATCGCCAAAGTCGTCTCCG

NC_000962.3 1399384 1399403 TBseq_1.0_770_LEFT 2 + ACGTCAATGCCACCGTGTC

NC_000962.3 1401371 1401390 TBseq_1.0_770_RIGHT 2 - ACGCGGTAGGTGGTCAAGT

NC_000962.3 1401173 1401193 TBseq_1.0_771_LEFT 1 + GTCCGGGAATCGAGCTGTTC

NC_000962.3 1403212 1403233 TBseq_1.0_771_RIGHT 1 - TATCGGTACTTCGACAGCCGC

NC_000962.3 1402966 1402985 TBseq_1.0_772_LEFT 2 + TGAGTTGAGGAATGCCGCG

NC_000962.3 1404990 1405013 TBseq_1.0_772_RIGHT 2 - GAGGAGCGGAATCTGATGATCTC

NC_000962.3 1404618 1404643 TBseq_1.0_773_LEFT 1 + CATTCCATCGGTAATATCAATCGGC

NC_000962.3 1406652 1406670 TBseq_1.0_773_RIGHT 1 - TATTGCGCACCCTCGGGA

NC_000962.3 1406438 1406459 TBseq_1.0_774_LEFT 2 + GCCAAACCCAGGGTAAGAACC

NC_000962.3 1408474 1408494 TBseq_1.0_774_RIGHT 2 - GTCCCGGAACAGCTCATTGC

NC_000962.3 1408224 1408245 TBseq_1.0_775_LEFT 1 + CCGGGATTGAGTGACGTGAAG

NC_000962.3 1410285 1410305 TBseq_1.0_775_RIGHT 1 - GCTATCTCGCGATCCTCGAC

NC_000962.3 1410132 1410151 TBseq_1.0_776_LEFT 2 + TGTCGCGTCAGCCAACTTC

NC_000962.3 1412101 1412124 TBseq_1.0_776_RIGHT 2 - GCCATAGTTCTCGCTAATCTCCC

NC_000962.3 1411805 1411825 TBseq_1.0_777_LEFT 1 + CGTCGGTGTCATGACATTGC

NC_000962.3 1413827 1413846 TBseq_1.0_777_RIGHT 1 - ACCAGCTTGACCGACCACG

NC_000962.3 1413639 1413659 TBseq_1.0_778_LEFT 2 + GACTATCGCCGATGAAGCCC

NC_000962.3 1415611 1415631 TBseq_1.0_778_RIGHT 2 - GTTGCAGGAACCTCACGTGG

NC_000962.3 1415383 1415401 TBseq_1.0_779_LEFT 1 + CAAAGTCGTCGCGGGTGA

NC_000962.3 1417381 1417401 TBseq_1.0_779_RIGHT 1 - CCGGTAGCGACACCCTATTG

NC_000962.3 1417165 1417185 TBseq_1.0_780_LEFT 2 + TCCCAGAGGGCGGTGATTAG

NC_000962.3 1419163 1419183 TBseq_1.0_780_RIGHT 2 - GCTCACGTCTGACCTCCGAA

NC_000962.3 1418986 1419010 TBseq_1.0_781_LEFT 1 + GGTTTCAACAAGCAATTAGGACGA

NC_000962.3 1420947 1420967 TBseq_1.0_781_RIGHT 1 - TACGAGATCGGTTCCGGACG

NC_000962.3 1420741 1420761 TBseq_1.0_782_LEFT 2 + CAGCAGGCAGTGTGTTCACA

NC_000962.3 1422669 1422690 TBseq_1.0_782_RIGHT 2 - CCGACGGCTTTGTACAAACAG

NC_000962.3 1422491 1422511 TBseq_1.0_783_LEFT 1 + GATACCTGTCGCAGCGATGC

NC_000962.3 1424471 1424491 TBseq_1.0_783_RIGHT 1 - AGGAGAAGGAGAAGTGCGGG

NC_000962.3 1424283 1424303 TBseq_1.0_784_LEFT 2 + GACAACAAACCGGGAGCGAC

NC_000962.3 1426249 1426269 TBseq_1.0_784_RIGHT 2 - GCATCCCATCCCTCCTGAGT

NC_000962.3 1426037 1426056 TBseq_1.0_785_LEFT 1 + GTTGCGATCGCCTAGCTCG

NC_000962.3 1427948 1427968 TBseq_1.0_785_RIGHT 1 - GAAGTAGCGCGCATACTCGG

NC_000962.3 1427793 1427811 TBseq_1.0_786_LEFT 2 + CGAACTGTGGCATGCCCA

NC_000962.3 1429751 1429770 TBseq_1.0_786_RIGHT 2 - GTTCTGTCGTCCAGGGTGC

NC_000962.3 1429513 1429532 TBseq_1.0_787_LEFT 1 + CGCAAGGGCAAGCTTGATG

NC_000962.3 1431445 1431465 TBseq_1.0_787_RIGHT 1 - GTGGTACAGGGTGTGCGAAC

NC_000962.3 1431247 1431265 TBseq_1.0_788_LEFT 2 + CGCATGCCAAGCCTGTCA

NC_000962.3 1433292 1433311 TBseq_1.0_788_RIGHT 2 - GTCGTGGCTATGGTGCTCA

NC_000962.3 1433134 1433154 TBseq_1.0_789_LEFT 1 + GAAGTTGGGCGGAAAGTCGG

NC_000962.3 1435135 1435154 TBseq_1.0_789_RIGHT 1 - GTGGGCGAATCGGGTTCAG

NC_000962.3 1434948 1434968 TBseq_1.0_790_LEFT 2 + ACGGTGTAGACAGGGGTGAG

NC_000962.3 1436930 1436950 TBseq_1.0_790_RIGHT 2 - CGGCTTGGACAGACCCATAC

NC_000962.3 1436677 1436696 TBseq_1.0_791_LEFT 1 + ACCACAAACGTCGGTGTGC

NC_000962.3 1438611 1438631 TBseq_1.0_791_RIGHT 1 - GGCGAAATAGATGGACGGCA

NC_000962.3 1438362 1438383 TBseq_1.0_792_LEFT 2 + CCATCCGGGAGAACCAATTCG

NC_000962.3 1440379 1440397 TBseq_1.0_792_RIGHT 2 - GCGGTCGGCCATGGAAAA

NC_000962.3 1440240 1440260 TBseq_1.0_793_LEFT 1 + GGTGTGGTTTACCGGACTGT

NC_000962.3 1442165 1442185 TBseq_1.0_793_RIGHT 1 - GGAAGTTCGCCTCGATCCAG

NC_000962.3 1441991 1442010 TBseq_1.0_794_LEFT 2 + CACCGACCAGGATTTCCGC

NC_000962.3 1443985 1444005 TBseq_1.0_794_RIGHT 2 - GGCAGATGGTGGATATCGCG

NC_000962.3 1443839 1443858 TBseq_1.0_795_LEFT 1 + AGCCAGAAGAGGAGCTCCC

NC_000962.3 1445785 1445805 TBseq_1.0_795_RIGHT 1 - GGTTGGCACCACCTTGACTG

NC_000962.3 1445382 1445404 TBseq_1.0_796_LEFT 2 + TTTCGGACTAGGATTTCTGGCC

NC_000962.3 1447387 1447407 TBseq_1.0_796_RIGHT 2 - CTTTAGCCGGGCGATGTAGC

NC_000962.3 1447081 1447104 TBseq_1.0_797_LEFT 1 + TCGACCACATCAAACAGTCTCTG

NC_000962.3 1449084 1449101 TBseq_1.0_797_RIGHT 1 - AGACACCAGCCGGACGT

NC_000962.3 1448760 1448780 TBseq_1.0_798_LEFT 2 + AAAAGACGGCACAGATCGCG

NC_000962.3 1450821 1450842 TBseq_1.0_798_RIGHT 2 - TTGGAGAGATTAGTTGCCGCG

NC_000962.3 1450623 1450643 TBseq_1.0_799_LEFT 1 + CCGTTGATGCACTGGACGAC

NC_000962.3 1452654 1452672 TBseq_1.0_799_RIGHT 1 - GTTCGGTGAGCGCAACCA

NC_000962.3 1452460 1452480 TBseq_1.0_800_LEFT 2 + CGTGGTTTCGTGGACTGACC

NC_000962.3 1454410 1454430 TBseq_1.0_800_RIGHT 2 - CCGGTCGAAAGTTGAAGCGA

NC_000962.3 1454057 1454077 TBseq_1.0_801_LEFT 1 + GCAGAAGTTCAACCCGCTGG

NC_000962.3 1456074 1456093 TBseq_1.0_801_RIGHT 1 - GAAGTATGCACGCGGCCTT

NC_000962.3 1455920 1455939 TBseq_1.0_802_LEFT 2 + ACGGTGTTGGACGAGACCA

NC_000962.3 1457878 1457898 TBseq_1.0_802_RIGHT 2 - TCGCCAAGATCCCATTGCAG

NC_000962.3 1457738 1457758 TBseq_1.0_803_LEFT 1 + GCGATATGCCGGTAGGTGTG

NC_000962.3 1459679 1459698 TBseq_1.0_803_RIGHT 1 - CGTATCGCAGTCGTCGCAC

NC_000962.3 1459449 1459469 TBseq_1.0_804_LEFT 2 + GCGACACTGATCCCCCTGTT

NC_000962.3 1461481 1461501 TBseq_1.0_804_RIGHT 2 - CACGTTCCCGCAAGACCTTC

NC_000962.3 1461281 1461305 TBseq_1.0_805_LEFT 1 + GTCAAGTAATTCGACGGCAAATGG

NC_000962.3 1463302 1463323 TBseq_1.0_805_RIGHT 1 - ACCGACTTCCTCTCTACTGGT

NC_000962.3 1463139 1463159 TBseq_1.0_806_LEFT 2 + TCTCGTCTCGTCTAGCTGCG

NC_000962.3 1465038 1465057 TBseq_1.0_806_RIGHT 2 - GGCCAGGGTGGTAAGCATC

NC_000962.3 1464814 1464836 TBseq_1.0_807_LEFT 1 + GAGGATAAGCTCGCCAAGGAAG

NC_000962.3 1466883 1466904 TBseq_1.0_807_RIGHT 1 - GGGAAGATGCCCTTGGAGAAC

NC_000962.3 1466603 1466624 TBseq_1.0_808_LEFT 2 + AGCAGGGTCAAGACGTATTGC

NC_000962.3 1468586 1468606 TBseq_1.0_808_RIGHT 2 - CTACGCGCTGTGGAAGAACC

NC_000962.3 1468398 1468418 TBseq_1.0_809_LEFT 1 + GACGAATACCGGGATGCGAC

NC_000962.3 1470404 1470423 TBseq_1.0_809_RIGHT 1 - ACAACGTCGCAGCCATGAG

NC_000962.3 1470265 1470283 TBseq_1.0_810_LEFT 2 + TCGCTGACTGGCTTGGCT

NC_000962.3 1472157 1472177 TBseq_1.0_810_RIGHT 2 - GCCGTATCTCAGTCCCAGTG

NC_000962.3 1471966 1471986 TBseq_1.0_811_LEFT 1 + GGTGATCTGCCCTGCACTTC

NC_000962.3 1473983 1474003 TBseq_1.0_811_RIGHT 1 - CCGGGTAGCGCTGAGACATA

NC_000962.3 1473761 1473781 TBseq_1.0_812_LEFT 2 + CCGAGGATTTCCGAATGGGG

NC_000962.3 1475761 1475781 TBseq_1.0_812_RIGHT 2 - ATTCTCCGCTTCACCCTTGC

NC_000962.3 1475565 1475586 TBseq_1.0_813_LEFT 1 + GCAAAATGCCCCCGTAACTTC

NC_000962.3 1477522 1477542 TBseq_1.0_813_RIGHT 1 - GCAGACGTATGAGCCAAGCC

NC_000962.3 1477325 1477345 TBseq_1.0_814_LEFT 2 + GATCTGGTCGGCGATTTCCC

NC_000962.3 1479358 1479378 TBseq_1.0_814_RIGHT 2 - CGATTGTGCGAACTGGCCAA

NC_000962.3 1479145 1479166 TBseq_1.0_815_LEFT 1 + CATGACTGGCGGAAAAACGAC

NC_000962.3 1481082 1481106 TBseq_1.0_815_RIGHT 1 - GCCAAGCAAAGATTCGAATACACA

NC_000962.3 1480940 1480959 TBseq_1.0_816_LEFT 2 + TCACCGTCTCGCCCAAAGA

NC_000962.3 1482873 1482892 TBseq_1.0_816_RIGHT 2 - CGAAATGCCGGAGTGCCAA

NC_000962.3 1482528 1482546 TBseq_1.0_817_LEFT 1 + CGCCCCACGGTAAATCGT

NC_000962.3 1484539 1484559 TBseq_1.0_817_RIGHT 1 - TCGTGGCTACTGTCGTGCTC

NC_000962.3 1484260 1484279 TBseq_1.0_818_LEFT 2 + GTCGTTGCGCGCTCTAAGG

NC_000962.3 1486329 1486347 TBseq_1.0_818_RIGHT 2 - GTCGTTGCGTTGCTCGGT

NC_000962.3 1486146 1486167 TBseq_1.0_819_LEFT 1 + CAACTCATTCGGGCCAGAGAG

NC_000962.3 1488116 1488136 TBseq_1.0_819_RIGHT 1 - ACGCAGAATCGCCTAAACCC

NC_000962.3 1487902 1487922 TBseq_1.0_820_LEFT 2 + TCAACCAGGATGTGAGTGCG

NC_000962.3 1489972 1489990 TBseq_1.0_820_RIGHT 2 - GACGCACAGCTGATCGGA

NC_000962.3 1489966 1489987 TBseq_1.0_821_LEFT 1 + CTTTCCTCCGATCAGCTGTGC

NC_000962.3 1491869 1491888 TBseq_1.0_821_RIGHT 1 - GTGTGGTGAGTGGCGTGTC

NC_000962.3 1491582 1491601 TBseq_1.0_822_LEFT 2 + CAGCCATCCAGTCGTCGTC

NC_000962.3 1493597 1493617 TBseq_1.0_822_RIGHT 2 - GGTTCGACGTGGTGTACCTG

NC_000962.3 1493326 1493345 TBseq_1.0_823_LEFT 1 + GCAGCTCGGTGAACCACTG

NC_000962.3 1495379 1495398 TBseq_1.0_823_RIGHT 1 - GGGTGAGCTTTTCGACGGC

NC_000962.3 1495215 1495235 TBseq_1.0_824_LEFT 2 + AGGTGGGTAGGGTTCCGTTG

NC_000962.3 1497198 1497217 TBseq_1.0_824_RIGHT 2 - GAGCAGACGCAAAAGCCCA

NC_000962.3 1496993 1497012 TBseq_1.0_825_LEFT 1 + GTCGAGATGGCGCATACCG

NC_000962.3 1498982 1499002 TBseq_1.0_825_RIGHT 1 - CGATCATCCGCGCTCTTTGC

NC_000962.3 1498796 1498817 TBseq_1.0_826_LEFT 2 + ACTGGCTGTGACTGAGTTGTG

NC_000962.3 1500761 1500782 TBseq_1.0_826_RIGHT 2 - CTCATCAAGTTGACCGGGTCG

NC_000962.3 1500592 1500611 TBseq_1.0_827_LEFT 1 + TGGGACCCAGTCGTTCACA

NC_000962.3 1502589 1502610 TBseq_1.0_827_RIGHT 1 - ACATGTCACGGTAGGTCGGTA

NC_000962.3 1502271 1502290 TBseq_1.0_828_LEFT 2 + GAGCCTTCAACACCCCGTT

NC_000962.3 1504343 1504364 TBseq_1.0_828_RIGHT 2 - CATGCCCATAGTTGCCCTTCC

NC_000962.3 1504027 1504046 TBseq_1.0_829_LEFT 1 + ACGGTGAGGGGGTATACGC

NC_000962.3 1505941 1505962 TBseq_1.0_829_RIGHT 1 - CTACTGTGCCATGCCCGATAC

NC_000962.3 1505717 1505735 TBseq_1.0_830_LEFT 2 + AAGTGGTCCGGGTGCTCA

NC_000962.3 1507716 1507736 TBseq_1.0_830_RIGHT 2 - TCCTCGAATGTCACACCGGT

NC_000962.3 1507350 1507371 TBseq_1.0_831_LEFT 1 + CCTGGTAGAGATTCAGGGCAC

NC_000962.3 1509321 1509340 TBseq_1.0_831_RIGHT 1 - CATCAAGTCGCCGGCAGAG

NC_000962.3 1508997 1509017 TBseq_1.0_832_LEFT 2 + GCCTTGCCTAATACCCCAGG

NC_000962.3 1511023 1511044 TBseq_1.0_832_RIGHT 2 - GTTCGACGAGTTTACCGACGT

NC_000962.3 1510829 1510850 TBseq_1.0_833_LEFT 1 + TGGAGATGGCGGACTGATGAC

NC_000962.3 1512780 1512801 TBseq_1.0_833_RIGHT 1 - GGGACTCGAACCCTCAAACTC

NC_000962.3 1512591 1512611 TBseq_1.0_834_LEFT 2 + GTGGGTTTGGTCATTGCGGG

NC_000962.3 1514546 1514565 TBseq_1.0_834_RIGHT 2 - AGGCGACCAGGAATCCGAT

NC_000962.3 1514321 1514340 TBseq_1.0_835_LEFT 1 + CTGTTCGTCGTGGACTGGC

NC_000962.3 1516335 1516352 TBseq_1.0_835_RIGHT 1 - ACAACAGCTGGCCCGGT

NC_000962.3 1515997 1516017 TBseq_1.0_836_LEFT 2 + GGCCTGGTGGTTAATCTGGT

NC_000962.3 1518014 1518034 TBseq_1.0_836_RIGHT 2 - CGTTTACCCGAATGCCGAGG

NC_000962.3 1517827 1517850 TBseq_1.0_837_LEFT 1 + TGCATCTGAAGGGAACATGGAAC

NC_000962.3 1519785 1519805 TBseq_1.0_837_RIGHT 1 - GACGATCTGCTCAACGCGAT

NC_000962.3 1519632 1519651 TBseq_1.0_838_LEFT 2 + AGCGCCGCTCGAATCAGTA

NC_000962.3 1521622 1521643 TBseq_1.0_838_RIGHT 2 - GCTCAGGCTGATCTACTTCGC

NC_000962.3 1521441 1521461 TBseq_1.0_839_LEFT 1 + GATGAACCCCAGCAGTCCAG

NC_000962.3 1523387 1523411 TBseq_1.0_839_RIGHT 1 - ATCAAAGTGATACTGCGACAAGCC

NC_000962.3 1523223 1523245 TBseq_1.0_840_LEFT 2 + TGGGGGACTTTGTCTTTTGTGG

NC_000962.3 1525197 1525216 TBseq_1.0_840_RIGHT 2 - TGGGTGGGACCGGTTGAAT

NC_000962.3 1524985 1525005 TBseq_1.0_841_LEFT 1 + CACGCGCGTTCACAAATACC

NC_000962.3 1527002 1527021 TBseq_1.0_841_RIGHT 1 - AGACCGCTACCCACCTCCT

NC_000962.3 1526767 1526788 TBseq_1.0_842_LEFT 2 + CCAGTGAGGCGTCTTGATCTG

NC_000962.3 1528692 1528715 TBseq_1.0_842_RIGHT 2 - CCTTGAGTATTACCTTCTCCGCA

NC_000962.3 1528335 1528356 TBseq_1.0_843_LEFT 1 + GTTGGAAACCGTACGCCAGTA

NC_000962.3 1530347 1530367 TBseq_1.0_843_RIGHT 1 - CACCATCGCGGCAGTTATCG

NC_000962.3 1530111 1530131 TBseq_1.0_844_LEFT 2 + CCCGGAGTTCGAATCGGATG

NC_000962.3 1532002 1532021 TBseq_1.0_844_RIGHT 2 - ATCACCTCGCACGACACCT

NC_000962.3 1531821 1531841 TBseq_1.0_845_LEFT 1 + CCCTATCCCGTTCCGAGAGT

NC_000962.3 1533853 1533871 TBseq_1.0_845_RIGHT 1 - ATCACGGCCCGACCTCAA

NC_000962.3 1533667 1533690 TBseq_1.0_846_LEFT 2 + CAATGATGGACAGCAAACAACGT

NC_000962.3 1535692 1535712 TBseq_1.0_846_RIGHT 2 - GACACCGAGGACATCTTCGC

NC_000962.3 1535510 1535530 TBseq_1.0_847_LEFT 1 + CACCCACCTGAGTTTTCCCC

NC_000962.3 1537507 1537526 TBseq_1.0_847_RIGHT 1 - CAGTGAACGCGGCCTATCG

NC_000962.3 1537373 1537393 TBseq_1.0_848_LEFT 2 + GCCACTCCGATCCTGATTGC

NC_000962.3 1539300 1539319 TBseq_1.0_848_RIGHT 2 - TCGGCTTCCAGCTTCAACC

NC_000962.3 1539116 1539135 TBseq_1.0_849_LEFT 1 + AGGCGAGCAAGAACAAGGC

NC_000962.3 1541011 1541034 TBseq_1.0_849_RIGHT 1 - GATCAAGCCATTCATGTTCAGCC

NC_000962.3 1540795 1540815 TBseq_1.0_850_LEFT 2 + GTGTTACTGTCGCGCTCTCC

NC_000962.3 1542787 1542807 TBseq_1.0_850_RIGHT 2 - CTACGACCACATCAACCGGG

NC_000962.3 1542629 1542648 TBseq_1.0_851_LEFT 1 + GGTCATCAGCCGTTCGACG

NC_000962.3 1544547 1544566 TBseq_1.0_851_RIGHT 1 - GGCAGCACGTGATGGACTC

NC_000962.3 1544366 1544386 TBseq_1.0_852_LEFT 2 + GCGACGCTGTGGGTTAGTAC

NC_000962.3 1546335 1546357 TBseq_1.0_852_RIGHT 2 - GTCCTACGCAGATGTAGCTGAC

NC_000962.3 1546066 1546085 TBseq_1.0_853_LEFT 1 + ACAACCTGCGATGGGATGC

NC_000962.3 1547962 1547983 TBseq_1.0_853_RIGHT 1 - GCAGACCCTAATACACGGCTT

NC_000962.3 1547601 1547621 TBseq_1.0_854_LEFT 2 + GCAGAGAACGGAGGAGGGAA

NC_000962.3 1549551 1549570 TBseq_1.0_854_RIGHT 2 - GGTGTGGCGCATGTTCACC

NC_000962.3 1549410 1549430 TBseq_1.0_855_LEFT 1 + CGCTGAGCTGCATCCATTCG

NC_000962.3 1551318 1551340 TBseq_1.0_855_RIGHT 1 - CCGGGATGTACTATCACTGCAC

NC_000962.3 1551108 1551127 TBseq_1.0_856_LEFT 2 + TGACATTGTCCTTGGGCGC

NC_000962.3 1553135 1553156 TBseq_1.0_856_RIGHT 2 - ACGTTCTTGCCCACATAGTCG

NC_000962.3 1552985 1553003 TBseq_1.0_857_LEFT 1 + TGACGCGCTGGTGATCCT

NC_000962.3 1554909 1554930 TBseq_1.0_857_RIGHT 1 - CCCTGGTCCTTAGCCCATTTC

NC_000962.3 1554649 1554668 TBseq_1.0_858_LEFT 2 + GTGCATGACCCGCTGATCA

NC_000962.3 1556646 1556665 TBseq_1.0_858_RIGHT 2 - CCGTTGGACAAGAACACGC

NC_000962.3 1556259 1556280 TBseq_1.0_859_LEFT 1 + GCACGTTGGAAGACGAACTCA

NC_000962.3 1558200 1558220 TBseq_1.0_859_RIGHT 1 - CACCGGGGAACTTCTCGAAC

NC_000962.3 1557656 1557676 TBseq_1.0_860_LEFT 2 + TAGCGTACTCCACCGACGAG

NC_000962.3 1559650 1559671 TBseq_1.0_860_RIGHT 2 - GTTGGCTTCCAGGACGTAGAG

NC_000962.3 1559476 1559496 TBseq_1.0_861_LEFT 1 + ATGGAGCACATCGAGGAGGC

NC_000962.3 1561408 1561428 TBseq_1.0_861_RIGHT 1 - TTCAACCGGACACTTAGCGC

NC_000962.3 1561408 1561428 TBseq_1.0_862_LEFT 2 + GCGCTAAGTGTCCGGTTGAA

NC_000962.3 1563429 1563449 TBseq_1.0_862_RIGHT 2 - GCCCTGACGATAGCTCTCGA

NC_000962.3 1563473 1563491 TBseq_1.0_863_LEFT 1 + GCGTTTGGGGCACTAGCA

NC_000962.3 1565444 1565465 TBseq_1.0_863_RIGHT 1 - TGGGGATCCGTTTATGGTCCA

NC_000962.3 1565154 1565174 TBseq_1.0_864_LEFT 2 + GTCGTCAGGTGCATCAGGTG

NC_000962.3 1567077 1567097 TBseq_1.0_864_RIGHT 2 - GTCGAAACCCTTGTCCGACG

NC_000962.3 1566879 1566901 TBseq_1.0_865_LEFT 1 + ACAAGATCTGTGACGCCATCAG

NC_000962.3 1568856 1568876 TBseq_1.0_865_RIGHT 1 - TTCGTGGAAGCAACGTTCCC

NC_000962.3 1568488 1568507 TBseq_1.0_866_LEFT 2 + CGTCCCAGAACCGGCTCAA

NC_000962.3 1570475 1570495 TBseq_1.0_866_RIGHT 2 - CTGTTCAGCCAGGACTTCGG

NC_000962.3 1570099 1570124 TBseq_1.0_867_LEFT 1 + CATCAGATACCAGAACCAGGACATG

NC_000962.3 1571996 1572015 TBseq_1.0_867_RIGHT 1 - CGAGACTTCGGTGTAGCCC

NC_000962.3 1572074 1572094 TBseq_1.0_868_LEFT 2 + CGGGTAGACCAGCCCTTTTC

NC_000962.3 1574093 1574110 TBseq_1.0_868_RIGHT 2 - ACCGACGACACCTCGCT

NC_000962.3 1573863 1573883 TBseq_1.0_869_LEFT 1 + CCCCCTGAATCCTGTGGGTA

NC_000962.3 1575932 1575949 TBseq_1.0_869_RIGHT 1 - TGCACAACGCCCAGACG

NC_000962.3 1575239 1575260 TBseq_1.0_870_LEFT 2 + GTACCACAGCAGCTGGAACAC

NC_000962.3 1577243 1577263 TBseq_1.0_870_RIGHT 2 - TCAGGTTGTCCTTCCCCAGG

NC_000962.3 1576901 1576918 TBseq_1.0_871_LEFT 1 + ACCGGGCAGCGAGTTGA

NC_000962.3 1578916 1578935 TBseq_1.0_871_RIGHT 1 - CGTAATCACCGCCGTACCC

NC_000962.3 1578121 1578140 TBseq_1.0_872_LEFT 2 + CACGGTACTGGCGATCGTG

NC_000962.3 1580089 1580109 TBseq_1.0_872_RIGHT 2 - CAGAAGCCCTCCCGTTTAGC

NC_000962.3 1579860 1579880 TBseq_1.0_873_LEFT 1 + CGTAATCCTCGCTTCCCCAC

NC_000962.3 1581777 1581798 TBseq_1.0_873_RIGHT 1 - GCTGGCTCTGGCAATATTTCG

NC_000962.3 1581596 1581614 TBseq_1.0_874_LEFT 2 + TCACGCCGATCGCTGAGA

NC_000962.3 1583547 1583567 TBseq_1.0_874_RIGHT 2 - GGATCGTTCTGCGCATCAGG

NC_000962.3 1583262 1583281 TBseq_1.0_875_LEFT 1 + GCCACCGAGCTGACATACG

NC_000962.3 1585295 1585315 TBseq_1.0_875_RIGHT 1 - TTGGGATCCACAATGACGGC

NC_000962.3 1585114 1585131 TBseq_1.0_876_LEFT 2 + TGTACGGCGCCGATGAC

NC_000962.3 1587080 1587099 TBseq_1.0_876_RIGHT 2 - CCAATCCCGACGGCAAACA

NC_000962.3 1586601 1586621 TBseq_1.0_877_LEFT 1 + CCAGCCCCAACCCTAAACCA

NC_000962.3 1588584 1588604 TBseq_1.0_877_RIGHT 1 - GTCACTTCTCCGCGTTCCTC

NC_000962.3 1588312 1588333 TBseq_1.0_878_LEFT 2 + CTCTTGAGAGCCTTGGTCTGC

NC_000962.3 1590372 1590392 TBseq_1.0_878_RIGHT 2 - GCTACCTAGCTGTCAGGCCT

NC_000962.3 1590181 1590200 TBseq_1.0_879_LEFT 1 + ATTCCCAACCACGTGTGCC

NC_000962.3 1592168 1592186 TBseq_1.0_879_RIGHT 1 - GCAACACGACGTCCCAGT

NC_000962.3 1591752 1591771 TBseq_1.0_880_LEFT 2 + ACGGAAAGATCTGCGACGC

NC_000962.3 1593762 1593784 TBseq_1.0_880_RIGHT 2 - TTCCGATATTCACGCATTCCCC

NC_000962.3 1593392 1593414 TBseq_1.0_881_LEFT 1 + GGGCATACTCAAATGTGTCCCA

NC_000962.3 1595465 1595482 TBseq_1.0_881_RIGHT 1 - TCTGATCGCGCAGGTGC

NC_000962.3 1595240 1595261 TBseq_1.0_882_LEFT 2 + GAGCATTCAGGACTCGTTGGG

NC_000962.3 1597228 1597247 TBseq_1.0_882_RIGHT 2 - TGCCTTTCACCCCGAGGAT

NC_000962.3 1596910 1596930 TBseq_1.0_883_LEFT 1 + GGACACGGCTTGTATGCGAC

NC_000962.3 1598881 1598900 TBseq_1.0_883_RIGHT 1 - CGAAGTAAGTCAGCCCGCT

NC_000962.3 1598741 1598760 TBseq_1.0_884_LEFT 2 + GCTTGCCGATCCTCCGATG

NC_000962.3 1600640 1600660 TBseq_1.0_884_RIGHT 2 - TGAGGGTGCTCTCGTGGATG

NC_000962.3 1600473 1600494 TBseq_1.0_885_LEFT 1 + CGGCAATACCTACAGAAGCGT

NC_000962.3 1602443 1602462 TBseq_1.0_885_RIGHT 1 - CCCGGAAACCCTCAAGCAG

NC_000962.3 1602227 1602247 TBseq_1.0_886_LEFT 2 + TACCGGACAGTGGGCGTAAT

NC_000962.3 1604213 1604234 TBseq_1.0_886_RIGHT 2 - CAGGAAAGCCAGCTCTACCTG

NC_000962.3 1604057 1604078 TBseq_1.0_887_LEFT 1 + CGGGTCTAACACCTGCATGAC

NC_000962.3 1606032 1606052 TBseq_1.0_887_RIGHT 1 - TGTCCGCATAGTTCCATCGC

NC_000962.3 1604363 1604383 TBseq_1.0_888_LEFT 2 + GGGAAAAACGACCACCACAC

NC_000962.3 1606302 1606323 TBseq_1.0_888_RIGHT 2 - GAATTGGGTATACGCCGGGTC

NC_000962.3 1606154 1606172 TBseq_1.0_889_LEFT 1 + TAACCGCCGGTCCGTTCA

NC_000962.3 1608091 1608111 TBseq_1.0_889_RIGHT 1 - TCGGGAAGATCGGGCTTCAG

NC_000962.3 1606366 1606385 TBseq_1.0_890_LEFT 2 + GGTTGGTCGGAAGGTCGGT

NC_000962.3 1608347 1608368 TBseq_1.0_890_RIGHT 2 - CCTCGAACAGCAACGTGAAGA

NC_000962.3 1608788 1608808 TBseq_1.0_891_LEFT 1 + TCATGCTGTGCTTGTGGTGG

NC_000962.3 1610783 1610802 TBseq_1.0_891_RIGHT 1 - GATTTCCGCGAAGGTCCCG

NC_000962.3 1610429 1610449 TBseq_1.0_892_LEFT 2 + GCTGGGGTTGATGATCCTGG

NC_000962.3 1612328 1612347 TBseq_1.0_892_RIGHT 2 - CGCTGACGCTGGTCCTCTT

NC_000962.3 1612167 1612186 TBseq_1.0_893_LEFT 1 + CTGCATAAGCCTGAGCCGC

NC_000962.3 1614164 1614184 TBseq_1.0_893_RIGHT 1 - GTGACGATGTCGCTCGAGAC

NC_000962.3 1613753 1613776 TBseq_1.0_894_LEFT 2 + CAGAACATCATCTCCAATGCGTC

NC_000962.3 1615756 1615779 TBseq_1.0_894_RIGHT 2 - TGACAAGTCTTGTGCACCATAGG

NC_000962.3 1615552 1615572 TBseq_1.0_895_LEFT 1 + GGAGGAGTTTTGTGAGCCGC

NC_000962.3 1617529 1617549 TBseq_1.0_895_RIGHT 1 - ACTTCAACCGAGTTGTCGCG

NC_000962.3 1616131 1616148 TBseq_1.0_896_LEFT 2 + AGGTGTGTGCGGCGATC

NC_000962.3 1618052 1618073 TBseq_1.0_896_RIGHT 2 - GCGCTAGCGGTATTTGATGAG

NC_000962.3 1617726 1617743 TBseq_1.0_897_LEFT 1 + TGACAGCCGCGGTCAGT

NC_000962.3 1619688 1619709 TBseq_1.0_897_RIGHT 1 - TGGCACCAGGTTTTAGACTGC

NC_000962.3 1618182 1618203 TBseq_1.0_898_LEFT 2 + AAACAGTGGTTAGTCGGCCTG

NC_000962.3 1620196 1620215 TBseq_1.0_898_RIGHT 2 - CTGTAGCTGTGCCGGGATG

NC_000962.3 1620024 1620045 TBseq_1.0_899_LEFT 1 + GTCAGCTGGGACGAGTTGATC

NC_000962.3 1621966 1621984 TBseq_1.0_899_RIGHT 1 - ACGTGCCGGAATCGTTGC

NC_000962.3 1621788 1621808 TBseq_1.0_900_LEFT 2 + CTTACTGACGGCGACATCGT

NC_000962.3 1623674 1623695 TBseq_1.0_900_RIGHT 2 - GACCGTCATGGCAGACAGAAG

NC_000962.3 1623450 1623470 TBseq_1.0_901_LEFT 1 + GTTTCGCGAGATCGTCCAGG

NC_000962.3 1625451 1625473 TBseq_1.0_901_RIGHT 1 - CGGAATCATCCTTGGAGATGCT

NC_000962.3 1625252 1625274 TBseq_1.0_902_LEFT 2 + GCAATGATGAGCGTGAGTACCC

NC_000962.3 1627177 1627199 TBseq_1.0_902_RIGHT 2 - CGTGAACACTATGCCGGAAAAG

NC_000962.3 1626998 1627018 TBseq_1.0_903_LEFT 1 + AGTAGCTCGTTCCAGGAGGC

NC_000962.3 1628953 1628972 TBseq_1.0_903_RIGHT 1 - ATAAAGGGCGCCGACTCCT

NC_000962.3 1628682 1628700 TBseq_1.0_904_LEFT 2 + ATCGGTTGATGGGTGGGC

NC_000962.3 1630595 1630614 TBseq_1.0_904_RIGHT 2 - ATGCCGCGATGCTTCTTCC

NC_000962.3 1638265 1638287 TBseq_1.0_905_LEFT 1 + GTCACACTCCTGCCAGATAAGG

NC_000962.3 1640170 1640190 TBseq_1.0_905_RIGHT 1 - CCTGACACAATGGGCCACTC

NC_000962.3 1639840 1639859 TBseq_1.0_906_LEFT 2 + GGGTGAAGTGGAACAGCGA

NC_000962.3 1641780 1641802 TBseq_1.0_906_RIGHT 2 - GCTGAAAGTCGAAATCACCACC

NC_000962.3 1641613 1641635 TBseq_1.0_907_LEFT 1 + GTGAAGTATTGCGTGGTACCGA

NC_000962.3 1643584 1643603 TBseq_1.0_907_RIGHT 1 - ACACCGGCAGAACTGATGC

NC_000962.3 1643167 1643189 TBseq_1.0_908_LEFT 2 + GGGATGAACATGGTCAACAGCA

NC_000962.3 1645127 1645148 TBseq_1.0_908_RIGHT 2 - GGTTCCATCCTGATCACGCTG

NC_000962.3 1644217 1644236 TBseq_1.0_909_LEFT 1 + GAAGCCGCAGTACCACCTC

NC_000962.3 1646247 1646270 TBseq_1.0_909_RIGHT 1 - CCGGGATTTTCACAACATCAGTG

NC_000962.3 1646042 1646062 TBseq_1.0_910_LEFT 2 + GGTGAGCTCGGTAGTGGTCA

NC_000962.3 1647999 1648020 TBseq_1.0_910_RIGHT 2 - GTTGACTTCAGCCTTCCACCC

NC_000962.3 1647670 1647690 TBseq_1.0_911_LEFT 1 + CCGAGAACATGGGCCAGTTC

NC_000962.3 1649567 1649586 TBseq_1.0_911_RIGHT 1 - ACAGCTCGCCCTTGTTGTG

NC_000962.3 1649375 1649395 TBseq_1.0_912_LEFT 2 + GTTCTACCTGATGAGCCGCG

NC_000962.3 1651350 1651370 TBseq_1.0_912_RIGHT 2 - GGTGGATGTAGCGCAGGATG

NC_000962.3 1651211 1651230 TBseq_1.0_913_LEFT 1 + TGCTCAAGCCCAAGATCGC

NC_000962.3 1653168 1653189 TBseq_1.0_913_RIGHT 1 - TCTTTGAACGCCATCCATCCG

NC_000962.3 1652933 1652954 TBseq_1.0_914_LEFT 2 + GGGTCACCGACGTTTCCTATG

NC_000962.3 1654887 1654907 TBseq_1.0_914_RIGHT 2 - GTGGGGATGTCGGCAATACC

NC_000962.3 1654705 1654726 TBseq_1.0_915_LEFT 1 + GTCGGAGAGGACTTTAAGCCC

NC_000962.3 1656723 1656743 TBseq_1.0_915_RIGHT 1 - CTCTTCATTGCGGAGGTGCC

NC_000962.3 1655549 1655567 TBseq_1.0_916_LEFT 2 + TCCGAGCGGGAAGAACGT

NC_000962.3 1657618 1657639 TBseq_1.0_916_RIGHT 2 - TGCAATACACCCGATCCGTTC

NC_000962.3 1657368 1657388 TBseq_1.0_917_LEFT 1 + CGGAATCGGTCAAAGGCCTG

NC_000962.3 1659437 1659458 TBseq_1.0_917_RIGHT 1 - CACCAGGAGGCCCAATAATCG

NC_000962.3 1659291 1659312 TBseq_1.0_918_LEFT 2 + GCATACACCTTCAAGTCCCCG

NC_000962.3 1661235 1661255 TBseq_1.0_918_RIGHT 2 - GTCGAGGTGGTTAGTCGGCT

NC_000962.3 1661015 1661034 TBseq_1.0_919_LEFT 1 + CGTTACGGTCAGCTCGAGG

NC_000962.3 1663016 1663034 TBseq_1.0_919_RIGHT 1 - GCTGTTTTTCGCGCTGGC

NC_000962.3 1662770 1662788 TBseq_1.0_920_LEFT 2 + GGCACATCGTCACGCACT

NC_000962.3 1664665 1664684 TBseq_1.0_920_RIGHT 2 - CGGGTGAAGTCGGACGAAC

NC_000962.3 1664512 1664531 TBseq_1.0_921_LEFT 1 + TGGTCTTCACCCACGGCTT

NC_000962.3 1666408 1666427 TBseq_1.0_921_RIGHT 1 - CGCAGCGGTGTGTCATTGG

NC_000962.3 1666250 1666270 TBseq_1.0_922_LEFT 2 + CATTCCGCAAGACGTCGACA

NC_000962.3 1668202 1668220 TBseq_1.0_922_RIGHT 2 - TGCGGCCCAGGTTGTACT

NC_000962.3 1667928 1667948 TBseq_1.0_923_LEFT 1 + ATCTTGCAGCCCACCGATAC

NC_000962.3 1669917 1669939 TBseq_1.0_923_RIGHT 1 - GATGATTTCGCGCTCTTCTTCG

NC_000962.3 1669701 1669720 TBseq_1.0_924_LEFT 2 + CGACGAGATCAACCGGGCT

NC_000962.3 1671665 1671686 TBseq_1.0_924_RIGHT 2 - CGATCACCAACATCACCACCG

NC_000962.3 1671413 1671433 TBseq_1.0_925_LEFT 1 + GCTTCGCGCATTCATGGTTC

NC_000962.3 1673330 1673350 TBseq_1.0_925_RIGHT 1 - ACTGGGGTTACGCTCGTGAC

NC_000962.3 1673169 1673188 TBseq_1.0_926_LEFT 2 + ATTGCGCGGTCAGTTCCAC

NC_000962.3 1675163 1675184 TBseq_1.0_926_RIGHT 2 - GCCATTGATCGGTGATACCCC

NC_000962.3 1674959 1674980 TBseq_1.0_927_LEFT 1 + CACGGGTGACATCATCTACGC

NC_000962.3 1676958 1676976 TBseq_1.0_927_RIGHT 1 - CAACGCCGCGATAAGCCA

NC_000962.3 1676755 1676775 TBseq_1.0_928_LEFT 2 + GCATCATAAGCTGCGACGGA

NC_000962.3 1678807 1678826 TBseq_1.0_928_RIGHT 2 - TCTCGCACCCGGACATGTG

NC_000962.3 1678661 1678680 TBseq_1.0_929_LEFT 1 + CTTGGACGTTCCGGTCAGC

NC_000962.3 1680551 1680571 TBseq_1.0_929_RIGHT 1 - CAGACGAAGACCTGCACGTT

NC_000962.3 1680359 1680381 TBseq_1.0_930_LEFT 2 + GTCAGAACCTTCTTTGCCGAAC

NC_000962.3 1682249 1682268 TBseq_1.0_930_RIGHT 2 - CGAGTTGTGCTGAGTCGGT

NC_000962.3 1681975 1681995 TBseq_1.0_931_LEFT 1 + GCGTACGGGGTCAACATGAA

NC_000962.3 1683959 1683979 TBseq_1.0_931_RIGHT 1 - TTCGACAAGGCTTGCACCAC

NC_000962.3 1683667 1683686 TBseq_1.0_932_LEFT 2 + CGAGCACAACATCCGGACG

NC_000962.3 1685645 1685665 TBseq_1.0_932_RIGHT 2 - GCCAGGGCCAGCAGATTATT

NC_000962.3 1685420 1685440 TBseq_1.0_933_LEFT 1 + ATCGACTCCGGTCAGCAACC

NC_000962.3 1687441 1687461 TBseq_1.0_933_RIGHT 1 - CCCTTCTTGATGCCCTGCAA

NC_000962.3 1687193 1687216 TBseq_1.0_934_LEFT 2 + CGATTCTTGGTGATAAAACCCGG

NC_000962.3 1689111 1689132 TBseq_1.0_934_RIGHT 2 - GGTGGTAGATGCCGACAAAGC

NC_000962.3 1688859 1688879 TBseq_1.0_935_LEFT 1 + TACGGTTCTTGTCGCGGGAG

NC_000962.3 1690851 1690870 TBseq_1.0_935_RIGHT 1 - CGTTACGATCGAGAGGCGC

NC_000962.3 1690689 1690707 TBseq_1.0_936_LEFT 2 + ATCAGCAGCCGAAACGCC

NC_000962.3 1692697 1692717 TBseq_1.0_936_RIGHT 2 - GCAAAACTAGCCTTGCCCCT

NC_000962.3 1692491 1692511 TBseq_1.0_937_LEFT 1 + AAACACCTCCGGCAAAGACC

NC_000962.3 1694483 1694502 TBseq_1.0_937_RIGHT 1 - AATCGCAGCCGCTTCCTTC

NC_000962.3 1694268 1694288 TBseq_1.0_938_LEFT 2 + GTTGTGAGAGCAGCCCTGTG

NC_000962.3 1696308 1696327 TBseq_1.0_938_RIGHT 2 - CGTCTCCCCCGAAATGGTG

NC_000962.3 1696150 1696169 TBseq_1.0_939_LEFT 1 + AGCGTCGCTTCGATGTGAG

NC_000962.3 1698157 1698177 TBseq_1.0_939_RIGHT 1 - GCACCACCTCATGCTTACCC

NC_000962.3 1698005 1698025 TBseq_1.0_940_LEFT 2 + GTAGGCACTGGTTCGGGTTG

NC_000962.3 1699985 1700009 TBseq_1.0_940_RIGHT 2 - CCCATACAATTCGCGATAATTCCG

NC_000962.3 1699789 1699812 TBseq_1.0_941_LEFT 1 + GACATCTAGGATGAACCCACAGC

NC_000962.3 1701802 1701822 TBseq_1.0_941_RIGHT 1 - CAGTGACCCGTACTGTGTCC

NC_000962.3 1701438 1701461 TBseq_1.0_942_LEFT 2 + GTCTACACGGTCATCTATCTGGC

NC_000962.3 1703446 1703466 TBseq_1.0_942_RIGHT 2 - CGACGAGGACGCCTGATAGA

NC_000962.3 1703289 1703309 TBseq_1.0_943_LEFT 1 + CTGCTGAGCACCATCGAACC

NC_000962.3 1705278 1705296 TBseq_1.0_943_RIGHT 1 - TGCAAGACGGCAAAGGGC

NC_000962.3 1705122 1705141 TBseq_1.0_944_LEFT 2 + TGCCGAACTGGACTGGGAA

NC_000962.3 1707102 1707122 TBseq_1.0_944_RIGHT 2 - CGATACCGTCTTCTGCTGCG

NC_000962.3 1706882 1706903 TBseq_1.0_945_LEFT 1 + GGAAGTCCCTCATGTTCGGTG

NC_000962.3 1708877 1708897 TBseq_1.0_945_RIGHT 1 - CCCCAAAAGGAGCACCATCG

NC_000962.3 1708724 1708743 TBseq_1.0_946_LEFT 2 + TCGAGTCGGTGCTGGTTGG

NC_000962.3 1710696 1710716 TBseq_1.0_946_RIGHT 2 - GATACGCGGACGCTTAAGGC

NC_000962.3 1710479 1710498 TBseq_1.0_947_LEFT 1 + GCTGGCCATGATCGTGGAG

NC_000962.3 1712505 1712524 TBseq_1.0_947_RIGHT 1 - GGCTTTGTGGCGCCAGAAT

NC_000962.3 1712318 1712338 TBseq_1.0_948_LEFT 2 + CTCTCTTCCTGGTGTGCTGC

NC_000962.3 1714343 1714363 TBseq_1.0_948_RIGHT 2 - GATGGCGTGTCGAAACCCAA

NC_000962.3 1714167 1714186 TBseq_1.0_949_LEFT 1 + AGGCTCAGGTGCTCAGCTT

NC_000962.3 1716134 1716154 TBseq_1.0_949_RIGHT 1 - AAGGTCTCGTTTCAGGCTGG

NC_000962.3 1715965 1715986 TBseq_1.0_950_LEFT 2 + CTGCAGGGTGTTGACTATCCC

NC_000962.3 1717900 1717920 TBseq_1.0_950_RIGHT 2 - TAAGAGCCACGGGGAGAGTG

NC_000962.3 1717725 1717746 TBseq_1.0_951_LEFT 1 + CAGATCAGGGGCATTTCGTGG

NC_000962.3 1719645 1719664 TBseq_1.0_951_RIGHT 1 - ACGGCAGGTGGGAAAGACC

NC_000962.3 1719322 1719342 TBseq_1.0_952_LEFT 2 + GAGATCCAAGCCTACGACGC

NC_000962.3 1721375 1721392 TBseq_1.0_952_RIGHT 2 - GTTTGTCGGCCCGCTGA

NC_000962.3 1721208 1721228 TBseq_1.0_953_LEFT 1 + ATAGATCAGGGCTCGCTCGC

NC_000962.3 1723175 1723194 TBseq_1.0_953_RIGHT 1 - TCATCCTCGACGTCCCCCA

NC_000962.3 1722973 1722992 TBseq_1.0_954_LEFT 2 + GCGGACGAGTTCGATGGTC

NC_000962.3 1724983 1725002 TBseq_1.0_954_RIGHT 2 - GTTGCGGTCCTATGGGTCG

NC_000962.3 1724708 1724728 TBseq_1.0_955_LEFT 1 + GCCACCAAATCCGTAGCGTC

NC_000962.3 1726675 1726693 TBseq_1.0_955_RIGHT 1 - ACCATCGCCGCGATCGAA

NC_000962.3 1726360 1726380 TBseq_1.0_956_LEFT 2 + AAGCACTTGTTGAGCAGGCA

NC_000962.3 1728352 1728372 TBseq_1.0_956_RIGHT 2 - GTGTTACGCCGGTAGCTGTC

NC_000962.3 1728216 1728237 TBseq_1.0_957_LEFT 1 + GTTCGGGGTCGTAGTACTCGT

NC_000962.3 1730153 1730170 TBseq_1.0_957_RIGHT 1 - CGGCAGCCAAGACACCA

NC_000962.3 1729991 1730010 TBseq_1.0_958_LEFT 2 + GCAGGGATACGCCGGAAAC

NC_000962.3 1731991 1732010 TBseq_1.0_958_RIGHT 2 - AACCCGGTCACCATCACGA

NC_000962.3 1731841 1731862 TBseq_1.0_959_LEFT 1 + AAGTGGCCACCTTGTTCAACC

NC_000962.3 1733812 1733832 TBseq_1.0_959_RIGHT 1 - CGATGTATTTGGGCGGCAGC

NC_000962.3 1733607 1733626 TBseq_1.0_960_LEFT 2 + CAATGCGGACCAGAGTCGC

NC_000962.3 1735653 1735673 TBseq_1.0_960_RIGHT 2 - ACCCCTCTCGTCAGAGCTTC

NC_000962.3 1734896 1734920 TBseq_1.0_961_LEFT 1 + TGATCTACTGGTACTTCGACTCCA

NC_000962.3 1736896 1736915 TBseq_1.0_961_RIGHT 1 - TGTAGCGCAACACGGATGC

NC_000962.3 1736748 1736769 TBseq_1.0_962_LEFT 2 + CACTATGCGCGGTTACAAGGT

NC_000962.3 1738658 1738678 TBseq_1.0_962_RIGHT 2 - TTCCATCGACTCGCTGAGGT

NC_000962.3 1738391 1738415 TBseq_1.0_963_LEFT 1 + GCAACTATCCAGACGTAACAGAGG

NC_000962.3 1740336 1740355 TBseq_1.0_963_RIGHT 1 - TCTTCAGCGTGCCGGACTA

NC_000962.3 1739820 1739841 TBseq_1.0_964_LEFT 2 + CCCCAAATAGCATCACGGGTG

NC_000962.3 1741869 1741888 TBseq_1.0_964_RIGHT 2 - CTCTGTGGCTGGACCTCAC

NC_000962.3 1741635 1741655 TBseq_1.0_965_LEFT 1 + GACGTGAAGCCAAGCGACTC

NC_000962.3 1743637 1743657 TBseq_1.0_965_RIGHT 1 - CAGCCATTGACGGTCCAACC

NC_000962.3 1743367 1743389 TBseq_1.0_966_LEFT 2 + TTCAAGGACATCCAGATCCGTC

NC_000962.3 1745408 1745428 TBseq_1.0_966_RIGHT 2 - ACGGCAATCGCATCCATGTC

NC_000962.3 1745189 1745208 TBseq_1.0_967_LEFT 1 + CAGGGCCGCAACCTCAATG

NC_000962.3 1747184 1747204 TBseq_1.0_967_RIGHT 1 - CAGAGGCCATCGCATGAGAC

NC_000962.3 1746919 1746936 TBseq_1.0_968_LEFT 2 + TGCCGAACGGAGTGCTG

NC_000962.3 1748986 1749005 TBseq_1.0_968_RIGHT 2 - CGTAGCGCACCATCTCACC

NC_000962.3 1748759 1748784 TBseq_1.0_969_LEFT 1 + GTTTCCCATCGTACTTTCTGATCGT

NC_000962.3 1750653 1750673 TBseq_1.0_969_RIGHT 1 - GATGCCCCGAGACGTACAGT

NC_000962.3 1753405 1753423 TBseq_1.0_970_LEFT 2 + TACGAAAAAGCCGGGCGC

NC_000962.3 1755379 1755399 TBseq_1.0_970_RIGHT 2 - GCTCGTAAACCTGCGGTCTC

NC_000962.3 1755180 1755197 TBseq_1.0_971_LEFT 1 + AGGTGATCGCGCGCATC

NC_000962.3 1757088 1757108 TBseq_1.0_971_RIGHT 1 - CACTGCTTGCCGACCTGTAG

NC_000962.3 1756836 1756858 TBseq_1.0_972_LEFT 2 + TTCGAGTTCTTGTTTTCTGGCC

NC_000962.3 1758825 1758844 TBseq_1.0_972_RIGHT 2 - GCGGTTGGCGCCATTAATG

NC_000962.3 1758598 1758618 TBseq_1.0_973_LEFT 1 + GGCCCCGTCGTCTATCTAGA

NC_000962.3 1760531 1760550 TBseq_1.0_973_RIGHT 1 - ACAGCACCATCCAGGCAAC

NC_000962.3 1760342 1760362 TBseq_1.0_974_LEFT 2 + TTGGACTTCAGCGCCAATCC

NC_000962.3 1762316 1762336 TBseq_1.0_974_RIGHT 2 - GCTATTCCGGCGATCAGGAG

NC_000962.3 1761990 1762010 TBseq_1.0_975_LEFT 1 + CGGCCTTGATCGAAAACGCT

NC_000962.3 1763952 1763972 TBseq_1.0_975_RIGHT 1 - CCAAGCACTTCGACGGCTAT

NC_000962.3 1763813 1763833 TBseq_1.0_976_LEFT 2 + CGGGGAGTTCATCGACCTGA

NC_000962.3 1765730 1765749 TBseq_1.0_976_RIGHT 2 - AGGACCCGCAGACATTCCA

NC_000962.3 1765538 1765557 TBseq_1.0_977_LEFT 1 + CAGGCGATCATCAGCTGGC

NC_000962.3 1767532 1767551 TBseq_1.0_977_RIGHT 1 - AGCGAACTGTGGGACGACA

NC_000962.3 1767384 1767401 TBseq_1.0_978_LEFT 2 + TATGCGCCGCCCAGGAA

NC_000962.3 1769279 1769298 TBseq_1.0_978_RIGHT 2 - CCCCATTTTGACGGCGGTC

NC_000962.3 1768971 1768991 TBseq_1.0_979_LEFT 1 + TCGACTCTGAGGTTGGCAAC

NC_000962.3 1771028 1771048 TBseq_1.0_979_RIGHT 1 - AGACCCATCCCAGTATCCCG

NC_000962.3 1770876 1770896 TBseq_1.0_980_LEFT 2 + CCCCAGTAGTTTCGCAAGCC

NC_000962.3 1772858 1772877 TBseq_1.0_980_RIGHT 2 - CCAATCGCCTGCTAGCCAC

NC_000962.3 1772714 1772733 TBseq_1.0_981_LEFT 1 + AGCAACCCGGATACCAGCA

NC_000962.3 1774628 1774648 TBseq_1.0_981_RIGHT 1 - CCGAGAGTGCCAAGGAGGTT

NC_000962.3 1774281 1774301 TBseq_1.0_982_LEFT 2 + CAGGACCTTTTGCCCAACCT

NC_000962.3 1776226 1776243 TBseq_1.0_982_RIGHT 2 - CGCCTTGCCGACACACA

NC_000962.3 1775985 1776002 TBseq_1.0_983_LEFT 1 + TACAGCGCGGCGTTCGA

NC_000962.3 1777961 1777980 TBseq_1.0_983_RIGHT 1 - GTCTGAACGGGCTTGCACA

NC_000962.3 1777362 1777383 TBseq_1.0_984_LEFT 2 + ACGAGTTAGGTCTAGCGGGTG

NC_000962.3 1779321 1779341 TBseq_1.0_984_RIGHT 2 - GGTTGAAACGTGCTGGTGTG

NC_000962.3 1779164 1779184 TBseq_1.0_985_LEFT 1 + TGTCCAAAGATCTTCGCGGG

NC_000962.3 1781053 1781072 TBseq_1.0_985_RIGHT 1 - GATTGTGGCGGCGGATGTT

NC_000962.3 1780788 1780808 TBseq_1.0_986_LEFT 2 + CAACTCTGTCGCCGATGAGG

NC_000962.3 1782730 1782756 TBseq_1.0_986_RIGHT 2 - GACCTTTTGTGTGTGATACAATCGAG

NC_000962.3 1782579 1782599 TBseq_1.0_987_LEFT 1 + GCCATGCGGAAAAGGATGCT

NC_000962.3 1784549 1784569 TBseq_1.0_987_RIGHT 1 - CACTGACAAGGCCCGTGATG

NC_000962.3 1784201 1784221 TBseq_1.0_988_LEFT 2 + CGGGGATCGTGGACTTGATC

NC_000962.3 1786208 1786228 TBseq_1.0_988_RIGHT 2 - CGTCTAGCGCCGATCCTACC

NC_000962.3 1785801 1785820 TBseq_1.0_989_LEFT 1 + TTGGCCGCGATGAACTTGC

NC_000962.3 1787848 1787868 TBseq_1.0_989_RIGHT 1 - CGTGGACGACTACAACGCTG

NC_000962.3 1786504 1786526 TBseq_1.0_990_LEFT 2 + GCGATGATGGTAGATGGTCGAC

NC_000962.3 1788456 1788478 TBseq_1.0_990_RIGHT 2 - GCTGTCTACCTCCGAATCTCAG

NC_000962.3 1788211 1788232 TBseq_1.0_991_LEFT 1 + TGACATGAAGGCTTCCAGCTC

NC_000962.3 1790109 1790129 TBseq_1.0_991_RIGHT 1 - CCAGCTATCAGCACTCGCAC

NC_000962.3 1789880 1789902 TBseq_1.0_992_LEFT 2 + GCTGCTAGAACTGGTGTTAGCT

NC_000962.3 1791895 1791913 TBseq_1.0_992_RIGHT 2 - AGCCCAATCGACAGCCCA

NC_000962.3 1791760 1791781 TBseq_1.0_993_LEFT 1 + CGAGTATCTGGGCAGCGAATC

NC_000962.3 1793718 1793738 TBseq_1.0_993_RIGHT 1 - GGTAGAGCCCGGCAATTTGG

NC_000962.3 1793264 1793285 TBseq_1.0_994_LEFT 2 + GGTACTGATACCGCCCATCCT

NC_000962.3 1795244 1795263 TBseq_1.0_994_RIGHT 2 - CGGGATCGATGGATGCGAC

NC_000962.3 1794936 1794953 TBseq_1.0_995_LEFT 1 + ACGTTGCCGACCACGTC

NC_000962.3 1796929 1796948 TBseq_1.0_995_RIGHT 1 - GCAAGCTGTTGGAGGCCAG

NC_000962.3 1796500 1796520 TBseq_1.0_996_LEFT 2 + CACCCCACGATGCTTTTTGC

NC_000962.3 1798493 1798511 TBseq_1.0_996_RIGHT 2 - ATGGTGGGCGGGAAGTTG

NC_000962.3 1798043 1798063 TBseq_1.0_997_LEFT 1 + CCTGCTGGACAATTTTGCGG

NC_000962.3 1800043 1800062 TBseq_1.0_997_RIGHT 1 - GCTGGCTACCACCAACGAG

NC_000962.3 1799814 1799833 TBseq_1.0_998_LEFT 2 + GACTGGACTGCGACGTCTG

NC_000962.3 1801814 1801834 TBseq_1.0_998_RIGHT 2 - GAAGTTGGCATCGCTTGGGA

NC_000962.3 1801586 1801605 TBseq_1.0_999_LEFT 1 + GCAAGGCATTCGCTTTCGC

NC_000962.3 1803606 1803625 TBseq_1.0_999_RIGHT 1 - GGGTTTTCCAGGGCAGCAG

NC_000962.3 1803295 1803316 TBseq_1.0_1000_LEFT 2 + GATGCCGCTGATACTTTTGCC

NC_000962.3 1805314 1805334 TBseq_1.0_1000_RIGHT 2 - TGAGTGGTGACCTCCCAACC

NC_000962.3 1805168 1805187 TBseq_1.0_1001_LEFT 1 + CTGGGGCTGACAAAGTCGC

NC_000962.3 1807118 1807138 TBseq_1.0_1001_RIGHT 1 - CCAACTGAATGGCACCGAGG

NC_000962.3 1806820 1806840 TBseq_1.0_1002_LEFT 2 + TGCTGAGCCTTGGATTGCTG

NC_000962.3 1808793 1808817 TBseq_1.0_1002_RIGHT 2 - ACTGGATCCAACAATCGAAAAGTC

NC_000962.3 1808631 1808651 TBseq_1.0_1003_LEFT 1 + GTGGATCAGATTGCAGCCGG

NC_000962.3 1810573 1810592 TBseq_1.0_1003_RIGHT 1 - CGCAGCACCGGAATCGAAA

NC_000962.3 1810411 1810430 TBseq_1.0_1004_LEFT 2 + TCAAGCGCGCTAGTCCTTC

NC_000962.3 1812333 1812351 TBseq_1.0_1004_RIGHT 2 - TGCCCAGCAAGCCAAACC

NC_000962.3 1812102 1812122 TBseq_1.0_1005_LEFT 1 + AACATGCGTGGCTCAAGGAG

NC_000962.3 1814152 1814170 TBseq_1.0_1005_RIGHT 1 - TCACCGCTTCCGCCACAT

NC_000962.3 1813882 1813901 TBseq_1.0_1006_LEFT 2 + TCTGTGTTGAGCTGCTGCG

NC_000962.3 1815863 1815883 TBseq_1.0_1006_RIGHT 2 - CTCACCGTGTAGCAGATCGT

NC_000962.3 1815522 1815542 TBseq_1.0_1007_LEFT 1 + GGGATCGGGCGAATCTACCT

NC_000962.3 1817543 1817560 TBseq_1.0_1007_RIGHT 1 - AACCCACTGTGCCTGGC

NC_000962.3 1817254 1817271 TBseq_1.0_1008_LEFT 2 + ATGCGGCCCGTGACATC

NC_000962.3 1819238 1819257 TBseq_1.0_1008_RIGHT 2 - CAGCCGTGCATGTGACACA

NC_000962.3 1818883 1818900 TBseq_1.0_1009_LEFT 1 + ACGGCCATGCATGCGAC

NC_000962.3 1820830 1820851 TBseq_1.0_1009_RIGHT 1 - CACAACACTGGCCATCCTGAT

NC_000962.3 1820602 1820622 TBseq_1.0_1010_LEFT 2 + TCAATCGTCACCCGGATCGA

NC_000962.3 1822641 1822660 TBseq_1.0_1010_RIGHT 2 - CGGAACTGTCTGCTGACCA

NC_000962.3 1822433 1822453 TBseq_1.0_1011_LEFT 1 + ATGAAACTGGACCCCAACGC

NC_000962.3 1824488 1824506 TBseq_1.0_1011_RIGHT 1 - TGGAACACGACGCGGAAC

NC_000962.3 1824277 1824296 TBseq_1.0_1012_LEFT 2 + GCCCATACCGACATGAGCG

NC_000962.3 1826259 1826278 TBseq_1.0_1012_RIGHT 2 - GTGACGCGGTTCTGTTGCT

NC_000962.3 1826080 1826100 TBseq_1.0_1013_LEFT 1 + CGACCAACGCCAGTATCCAG

NC_000962.3 1828052 1828072 TBseq_1.0_1013_RIGHT 1 - TGGCAGAGGAAACGGACTCA

NC_000962.3 1827783 1827802 TBseq_1.0_1014_LEFT 2 + GAGCCAGGCGGTGATGGTA

NC_000962.3 1829852 1829870 TBseq_1.0_1014_RIGHT 2 - CACGTTCGCCCAGAAGCT

NC_000962.3 1829544 1829567 TBseq_1.0_1015_LEFT 1 + GGGAGTTCTTGACCTTCACTTGA

NC_000962.3 1831549 1831569 TBseq_1.0_1015_RIGHT 1 - AACGTGTCGAACAACCGGTC

NC_000962.3 1831346 1831365 TBseq_1.0_1016_LEFT 2 + TGGTGGACAACGTTGACGC

NC_000962.3 1833295 1833313 TBseq_1.0_1016_RIGHT 2 - GTAAGCGCCGCCCATCTT

NC_000962.3 1832942 1832961 TBseq_1.0_1017_LEFT 1 + ACGAGCAGATGGACGCGTA

NC_000962.3 1834923 1834941 TBseq_1.0_1017_RIGHT 1 - TCGCTGGCCAGTGATCCA

NC_000962.3 1834748 1834769 TBseq_1.0_1018_LEFT 2 + GGCTTGAGGGATTCGAAAAGC

NC_000962.3 1836743 1836764 TBseq_1.0_1018_RIGHT 2 - CGTGCCCAATTCCACTACCTC

NC_000962.3 1836542 1836562 TBseq_1.0_1019_LEFT 1 + GAGTAGTTCGTCGACGTCGG

NC_000962.3 1838499 1838519 TBseq_1.0_1019_RIGHT 1 - TCGACCTCCGAATGCAGGTA

NC_000962.3 1838329 1838348 TBseq_1.0_1020_LEFT 2 + TGGTGGACCCGAAAGTGGT

NC_000962.3 1840268 1840285 TBseq_1.0_1020_RIGHT 2 - GCCCACAGCGCGATGAT

NC_000962.3 1840043 1840064 TBseq_1.0_1021_LEFT 1 + GATACCTACGTGCCGCTGTTC

NC_000962.3 1841980 1841999 TBseq_1.0_1021_RIGHT 1 - TTGTGGTTCGACGAGGGGG

NC_000962.3 1841846 1841866 TBseq_1.0_1022_LEFT 2 + CCATAATTCGGTGGGCGGAA

NC_000962.3 1843778 1843796 TBseq_1.0_1022_RIGHT 2 - TCGACGCTGCGCAGATTG

NC_000962.3 1843641 1843661 TBseq_1.0_1023_LEFT 1 + CGTTACGACGTTACCGGTCC

NC_000962.3 1845603 1845622 TBseq_1.0_1023_RIGHT 1 - TGAGTTGACGACGGGGGTC

NC_000962.3 1845416 1845434 TBseq_1.0_1024_LEFT 2 + GCACGACGAGGACACCAT

NC_000962.3 1847310 1847329 TBseq_1.0_1024_RIGHT 2 - GCAGCTACACCGGACTGTC

NC_000962.3 1847026 1847046 TBseq_1.0_1025_LEFT 1 + GCAACGGGATCTTAGGCACT

NC_000962.3 1849013 1849032 TBseq_1.0_1025_RIGHT 1 - GCGATCTCGGAAGCACTCG

NC_000962.3 1848843 1848866 TBseq_1.0_1026_LEFT 2 + TGTCGGAAAGTCGATGTAGAACG

NC_000962.3 1850851 1850873 TBseq_1.0_1026_RIGHT 2 - CACAAGGGGCTCAGACATATCG

NC_000962.3 1850554 1850574 TBseq_1.0_1027_LEFT 1 + GGATCAATCGCCTCCACCAG

NC_000962.3 1852621 1852643 TBseq_1.0_1027_RIGHT 1 - CCACGGAACATAATGGTGACCT

NC_000962.3 1852441 1852463 TBseq_1.0_1028_LEFT 2 + GCAAGATCATGGACTACGGCAA

NC_000962.3 1854440 1854461 TBseq_1.0_1028_RIGHT 2 - GTCCATTCCACACTTTCGCTG

NC_000962.3 1853833 1853853 TBseq_1.0_1029_LEFT 1 + AAGGCGCTCTCTGATACGGT

NC_000962.3 1855743 1855764 TBseq_1.0_1029_RIGHT 1 - TAGCCCGACTCCTTGTACACG

NC_000962.3 1855527 1855544 TBseq_1.0_1030_LEFT 2 + CCGATATCGCGCCGTGT

NC_000962.3 1857507 1857527 TBseq_1.0_1030_RIGHT 2 - TCATGATGCCCCCTTTGGTG

NC_000962.3 1857371 1857393 TBseq_1.0_1031_LEFT 1 + ATGCTGTGAAGTCGCTTGAAGT

NC_000962.3 1859355 1859375 TBseq_1.0_1031_RIGHT 1 - GATGGAAGATGGGCGTGTGG

NC_000962.3 1859154 1859174 TBseq_1.0_1032_LEFT 2 + CAACTTCGACGCCCTCAACT

NC_000962.3 1861163 1861181 TBseq_1.0_1032_RIGHT 2 - ACAAGGTCTGCGGGTTGC

NC_000962.3 1860143 1860163 TBseq_1.0_1033_LEFT 1 + CGAACTCAATTTGGGCGCAG

NC_000962.3 1862161 1862182 TBseq_1.0_1033_RIGHT 1 - CGTCTTCGGTTAAGGTGCGAT

NC_000962.3 1865483 1865504 TBseq_1.0_1034_LEFT 2 + GACGCAACTTCGAGTCCCATC

NC_000962.3 1867555 1867574 TBseq_1.0_1034_RIGHT 2 - GCGGTCTTGACCAGGCTGT

NC_000962.3 1867228 1867247 TBseq_1.0_1035_LEFT 1 + CCATGCTGTGCGTGCTCAC

NC_000962.3 1869289 1869309 TBseq_1.0_1035_RIGHT 1 - TCCCCCATGATCGGTTCCAG

NC_000962.3 1869036 1869058 TBseq_1.0_1036_LEFT 2 + GCGAGTGTTCTTCTGCAACTCC

NC_000962.3 1870937 1870955 TBseq_1.0_1036_RIGHT 2 - GTTTTGGCTGCGCACCTG

NC_000962.3 1869860 1869879 TBseq_1.0_1037_LEFT 1 + CACAGCTCGACGGCTTTGT

NC_000962.3 1871849 1871869 TBseq_1.0_1037_RIGHT 1 - GGTGACGTTGATCGGGATCG

NC_000962.3 1871413 1871434 TBseq_1.0_1038_LEFT 2 + GCGATCAGCTGGATAGGCAAG

NC_000962.3 1873435 1873456 TBseq_1.0_1038_RIGHT 2 - GTGACGTAGCCGAATTCCGTC

NC_000962.3 1873103 1873121 TBseq_1.0_1039_LEFT 1 + ATGCCCGGCAAAACCCAC

NC_000962.3 1875151 1875171 TBseq_1.0_1039_RIGHT 1 - CGATCATCAACCCGGGACTT

NC_000962.3 1874670 1874690 TBseq_1.0_1040_LEFT 2 + CGTATCCGGGATACAAGCCG

NC_000962.3 1876675 1876692 TBseq_1.0_1040_RIGHT 2 - TTCCACTGCCGGTGCCT

NC_000962.3 1876429 1876450 TBseq_1.0_1041_LEFT 1 + CGAACATCGGTCATACGTCGG

NC_000962.3 1878379 1878401 TBseq_1.0_1041_RIGHT 1 - GCCGAATACACCCACACATTCC

NC_000962.3 1877944 1877961 TBseq_1.0_1042_LEFT 2 + TTGCGGGCGTGGGTGTT

NC_000962.3 1879945 1879966 TBseq_1.0_1042_RIGHT 2 - ATCGCGAGTCGGAGATATGGA

NC_000962.3 1879766 1879786 TBseq_1.0_1043_LEFT 1 + GTTTTACGGGCTGTCGGTGT

NC_000962.3 1881708 1881727 TBseq_1.0_1043_RIGHT 1 - AACATGCGTGGTGGTTCCC

NC_000962.3 1881512 1881531 TBseq_1.0_1044_LEFT 2 + CCGCGAGCTAGTTGTGCTG

NC_000962.3 1883544 1883565 TBseq_1.0_1044_RIGHT 2 - AACTCGGTGCTAGTCAACAGC

NC_000962.3 1883410 1883430 TBseq_1.0_1045_LEFT 1 + CAATGGGTAGGCATGGGAGC

NC_000962.3 1885371 1885391 TBseq_1.0_1045_RIGHT 1 - ACTCCAACTCCCAGACCACG

NC_000962.3 1885226 1885246 TBseq_1.0_1046_LEFT 2 + TGTCGGTACAGGCATTGGTC

NC_000962.3 1887125 1887145 TBseq_1.0_1046_RIGHT 2 - CTGCAGCATAATTGGCCTGG

NC_000962.3 1886827 1886847 TBseq_1.0_1047_LEFT 1 + CAGCATTGGTGGACGAGCTC

NC_000962.3 1888787 1888806 TBseq_1.0_1047_RIGHT 1 - TGATGACGTCCACCTGGCC

NC_000962.3 1888572 1888591 TBseq_1.0_1048_LEFT 2 + AACAAGAATTCGGCGCGGT

NC_000962.3 1890583 1890603 TBseq_1.0_1048_RIGHT 2 - GTGGGGCTAGGCTTTGCAAT

NC_000962.3 1890434 1890454 TBseq_1.0_1049_LEFT 1 + GCGGTTGATTGGTCTTCGGT

NC_000962.3 1892371 1892389 TBseq_1.0_1049_RIGHT 1 - AGGGCCATGCTGCAGTTG

NC_000962.3 1892019 1892040 TBseq_1.0_1050_LEFT 2 + GCTGACCAAAGACGACATCGG

NC_000962.3 1893964 1893982 TBseq_1.0_1050_RIGHT 2 - GTGGGCGGGTACGTTGAT

NC_000962.3 1893652 1893669 TBseq_1.0_1051_LEFT 1 + TGGGTCAGCCGGGTGAT

NC_000962.3 1895697 1895716 TBseq_1.0_1051_RIGHT 1 - TTCAACCGGGTGCAAACCC

NC_000962.3 1895291 1895311 TBseq_1.0_1052_LEFT 2 + CTGAGTCGGATAGGCCAGGT

NC_000962.3 1897196 1897217 TBseq_1.0_1052_RIGHT 2 - CCGATAGTGACGCTGAGTTGG

NC_000962.3 1896999 1897018 TBseq_1.0_1053_LEFT 1 + GTCAGCAACGCGAGATGGT

NC_000962.3 1898998 1899019 TBseq_1.0_1053_RIGHT 1 - AACCTGATGTTGTACAGCCGG

NC_000962.3 1898771 1898790 TBseq_1.0_1054_LEFT 2 + TAGATGCGACGGGCCTGTT

NC_000962.3 1900783 1900804 TBseq_1.0_1054_RIGHT 2 - CAAGTCAAGGAGTTTGCCCCC

NC_000962.3 1900617 1900637 TBseq_1.0_1055_LEFT 1 + CCATCGGCATCTCCAGTAGC

NC_000962.3 1902559 1902580 TBseq_1.0_1055_RIGHT 1 - GCGTGAAGTGATACAGGTCCC

NC_000962.3 1902372 1902393 TBseq_1.0_1056_LEFT 2 + GCGTGTGATACTGCCCTCATG

NC_000962.3 1904375 1904395 TBseq_1.0_1056_RIGHT 2 - GCCCTGCCGTAGAAGTCGTA

NC_000962.3 1904197 1904216 TBseq_1.0_1057_LEFT 1 + CACCACCTTGACCCACGTG

NC_000962.3 1906212 1906231 TBseq_1.0_1057_RIGHT 1 - GGAACAACGCGGCCTCTTC

NC_000962.3 1906051 1906070 TBseq_1.0_1058_LEFT 2 + AATCGCTGGTTCCCGAGGT

NC_000962.3 1908029 1908048 TBseq_1.0_1058_RIGHT 2 - CCCTGCGAATACCCGACGA

NC_000962.3 1907710 1907731 TBseq_1.0_1059_LEFT 1 + CCGTCCCAAATCGTTGAGAGC

NC_000962.3 1909633 1909650 TBseq_1.0_1059_RIGHT 1 - AAGCCGCGGGACAATGC

NC_000962.3 1909479 1909499 TBseq_1.0_1060_LEFT 2 + GATCACCAACTACCGCTGGG

NC_000962.3 1911455 1911475 TBseq_1.0_1060_RIGHT 2 - GTCGTCGTCGTGCTGAGTTT

NC_000962.3 1911314 1911333 TBseq_1.0_1061_LEFT 1 + GGGCACTGGACAGGATTCG

NC_000962.3 1913208 1913229 TBseq_1.0_1061_RIGHT 1 - ACGTTGGCACAGACATGGATC

NC_000962.3 1913072 1913091 TBseq_1.0_1062_LEFT 2 + TCGAGGTCGAGGCGTATGG

NC_000962.3 1915057 1915077 TBseq_1.0_1062_RIGHT 2 - TCATCACCTCCCAATCCCCC

NC_000962.3 1914913 1914933 TBseq_1.0_1063_LEFT 1 + GGTGATATGCGACGTGAGCG

NC_000962.3 1916810 1916830 TBseq_1.0_1063_RIGHT 1 - TGGTGACAAACAGCTTGCGG

NC_000962.3 1916559 1916580 TBseq_1.0_1064_LEFT 2 + CGCCTACGCTGAAATACTGCT

NC_000962.3 1918600 1918617 TBseq_1.0_1064_RIGHT 2 - CTTGACGCCGACGCTGT

NC_000962.3 1918306 1918325 TBseq_1.0_1065_LEFT 1 + GCAACGATCCTCGGGTGGT

NC_000962.3 1920248 1920267 TBseq_1.0_1065_RIGHT 1 - ATCGGCCTCTTGCGCTAGT

NC_000962.3 1920041 1920060 TBseq_1.0_1066_LEFT 2 + CGCCAAATCGTTGAGCGGT

NC_000962.3 1922006 1922026 TBseq_1.0_1066_RIGHT 2 - GGGCTTTCACTGCGGATGAA

NC_000962.3 1921768 1921788 TBseq_1.0_1067_LEFT 1 + CGGTCCAGAGGTGTTGGTCA

NC_000962.3 1923834 1923851 TBseq_1.0_1067_RIGHT 1 - GCGGTTTGCGGGTGCTT

NC_000962.3 1923615 1923634 TBseq_1.0_1068_LEFT 2 + ACCTGATCAACGGAGGCCA

NC_000962.3 1925617 1925636 TBseq_1.0_1068_RIGHT 2 - TGGCTCCCGTATGCAAGGT

NC_000962.3 1925200 1925219 TBseq_1.0_1069_LEFT 1 + ATTCGGTTGTTGCCCAGGC

NC_000962.3 1927145 1927164 TBseq_1.0_1069_RIGHT 1 - ACCGCTACCGCTAGTGACG

NC_000962.3 1926966 1926989 TBseq_1.0_1070_LEFT 2 + CATATGTTGAGGCATTCGTTCGC

NC_000962.3 1928993 1929013 TBseq_1.0_1070_RIGHT 2 - CGATGACGCCCCAATGTGAT

NC_000962.3 1928911 1928933 TBseq_1.0_1071_LEFT 1 + GAATAGGTTTGAGGTCCAGCCC

NC_000962.3 1930884 1930904 TBseq_1.0_1071_RIGHT 1 - TTCGTCTCCCCTCATGGTGT

NC_000962.3 1930593 1930616 TBseq_1.0_1072_LEFT 2 + GCTAGGGAGAACATCGTCACAAA

NC_000962.3 1932654 1932677 TBseq_1.0_1072_RIGHT 2 - GACGAGGTAAGGAGTTCATAGCG

NC_000962.3 1933878 1933899 TBseq_1.0_1073_LEFT 1 + CGAAAACCGCCTCGTTATGGG

NC_000962.3 1935775 1935798 TBseq_1.0_1073_RIGHT 1 - GCAACGAAAACGAACAAAACGAC

NC_000962.3 1935640 1935660 TBseq_1.0_1074_LEFT 2 + GAACTGATCGGCCAGGGGAT

NC_000962.3 1937622 1937641 TBseq_1.0_1074_RIGHT 2 - TCCAGAAGCGCGAGGTCTT

NC_000962.3 1936966 1936985 TBseq_1.0_1075_LEFT 1 + GCACGGACGGCGTGATAAT

NC_000962.3 1938890 1938912 TBseq_1.0_1075_RIGHT 1 - GATTCCAGCCTGAGACAACACT

NC_000962.3 1938720 1938740 TBseq_1.0_1076_LEFT 2 + TCCCGACGTCGACACAATTG

NC_000962.3 1940698 1940717 TBseq_1.0_1076_RIGHT 2 - CCTTGTTGGCGGCCAAGAA

NC_000962.3 1940271 1940289 TBseq_1.0_1077_LEFT 1 + GAAGTGAGGCAGTGCGGT

NC_000962.3 1942218 1942237 TBseq_1.0_1077_RIGHT 1 - CGTTCGCGTCCATCACAGC

NC_000962.3 1941730 1941751 TBseq_1.0_1078_LEFT 2 + TTCTGCTGTCCCATAATGCGG

NC_000962.3 1943664 1943683 TBseq_1.0_1078_RIGHT 2 - CACGTCCTCGAAGTACGGC

NC_000962.3 1943533 1943550 TBseq_1.0_1079_LEFT 1 + CGCGCAACTCCAAGCCA

NC_000962.3 1945472 1945493 TBseq_1.0_1079_RIGHT 1 - GGTACAAGGTGTCCTCCAAGC

NC_000962.3 1945262 1945279 TBseq_1.0_1080_LEFT 2 + TGCGACTGTGGGCGGAA

NC_000962.3 1947305 1947324 TBseq_1.0_1080_RIGHT 2 - CGCCCCTGCACATTTCGAT

NC_000962.3 1947049 1947069 TBseq_1.0_1081_LEFT 1 + CCGCATTATGGCCATGGGAT

NC_000962.3 1949053 1949071 TBseq_1.0_1081_RIGHT 1 - TGGTTCAGGCGCCACATC

NC_000962.3 1948881 1948902 TBseq_1.0_1082_LEFT 2 + CACTGTTCCTCTTCCACCTGC

NC_000962.3 1950871 1950893 TBseq_1.0_1082_RIGHT 2 - AGCACTAGCCAAACACTTCCAG

NC_000962.3 1950713 1950732 TBseq_1.0_1083_LEFT 1 + TTTTCGATGGCACGACCGG

NC_000962.3 1952625 1952642 TBseq_1.0_1083_RIGHT 1 - TTGCCGCGCTGGTCAAC

NC_000962.3 1952311 1952329 TBseq_1.0_1084_LEFT 2 + TCGGACTGAGCTGCGACA

NC_000962.3 1954335 1954353 TBseq_1.0_1084_RIGHT 2 - TCCCACGGAAGCGGACTT

NC_000962.3 1953843 1953862 TBseq_1.0_1085_LEFT 1 + CGTTTGGTTGTCGCGTTCG

NC_000962.3 1955813 1955832 TBseq_1.0_1085_RIGHT 1 - GCCTTCTATCGGCCCGACT

NC_000962.3 1955536 1955557 TBseq_1.0_1086_LEFT 2 + CAGATCCCAGTTGTCGTCGTC

NC_000962.3 1957543 1957561 TBseq_1.0_1086_RIGHT 2 - TACGGGTTGTGGGGCCAA

NC_000962.3 1957344 1957363 TBseq_1.0_1087_LEFT 1 + GTCGCGTCCACATGAGTGT

NC_000962.3 1959275 1959294 TBseq_1.0_1087_RIGHT 1 - AGTCAACCCCGACCAACGA

NC_000962.3 1959089 1959112 TBseq_1.0_1088_LEFT 2 + TTGCTCAAGTACACCGAATCACA

NC_000962.3 1961023 1961044 TBseq_1.0_1088_RIGHT 2 - CTCGGAGCTATCGGACCTCAC

NC_000962.3 1960804 1960827 TBseq_1.0_1089_LEFT 1 + TCGTTGATCGACAGGAAGAACAG

NC_000962.3 1962775 1962796 TBseq_1.0_1089_RIGHT 1 - CCAGCCCGATGTTTCATTTCG

NC_000962.3 1961567 1961586 TBseq_1.0_1090_LEFT 2 + AACACGACGCTCCACAACG

NC_000962.3 1963489 1963509 TBseq_1.0_1090_RIGHT 2 - GTTCTACGACTGGTACGCCG

NC_000962.3 1963076 1963095 TBseq_1.0_1091_LEFT 1 + GGCGAATTCGAGCACCACC

NC_000962.3 1965079 1965098 TBseq_1.0_1091_RIGHT 1 - GGCAACCATGGGCTCCTAC

NC_000962.3 1964945 1964965 TBseq_1.0_1092_LEFT 2 + ACCATGCCCATACCGAACAC

NC_000962.3 1966833 1966853 TBseq_1.0_1092_RIGHT 2 - CCGGGATCGCGATTGTTACC

NC_000962.3 1966534 1966554 TBseq_1.0_1093_LEFT 1 + CGTAGCGCGGCATATACCAC

NC_000962.3 1968549 1968568 TBseq_1.0_1093_RIGHT 1 - CAACAGGTACGGCGTGGTG

NC_000962.3 1968344 1968363 TBseq_1.0_1094_LEFT 2 + CGGTCTTGGATCCGTTCCC

NC_000962.3 1970333 1970353 TBseq_1.0_1094_RIGHT 2 - GTTCTTGCTGTGCGACTGGT

NC_000962.3 1970194 1970218 TBseq_1.0_1095_LEFT 1 + CCGACAACGATCGACATTTTCAAC

NC_000962.3 1972092 1972110 TBseq_1.0_1095_RIGHT 1 - AGGGCTTGCGACACTCGA

NC_000962.3 1971931 1971950 TBseq_1.0_1096_LEFT 2 + GCGGTCGTTGAGCAAAACC

NC_000962.3 1973885 1973905 TBseq_1.0_1096_RIGHT 2 - TCCGATATGGACTCGCTGGG

NC_000962.3 1972400 1972419 TBseq_1.0_1097_LEFT 1 + CATGGACTTCGTCGACGGC

NC_000962.3 1974330 1974350 TBseq_1.0_1097_RIGHT 1 - CGATGTCGTTGTCGTTCGCC

NC_000962.3 1974065 1974082 TBseq_1.0_1098_LEFT 2 + AACAGCTTCCACCGGCC

NC_000962.3 1976040 1976057 TBseq_1.0_1098_RIGHT 2 - ACGCCGCGAAACCCCAT

NC_000962.3 1975720 1975741 TBseq_1.0_1099_LEFT 1 + GCGAAGGTCTGTGTCTACACC

NC_000962.3 1977689 1977708 TBseq_1.0_1099_RIGHT 1 - ATCGTGGGCGTGGTATCGA

NC_000962.3 1977494 1977514 TBseq_1.0_1100_LEFT 2 + CGACGTACTGGATGGTGAGC

NC_000962.3 1979479 1979500 TBseq_1.0_1100_RIGHT 2 - GTGGTCTGCAATGGACTTGGA

NC_000962.3 1979160 1979179 TBseq_1.0_1101_LEFT 1 + GCCACCAATTCAGCCCACC

NC_000962.3 1981188 1981206 TBseq_1.0_1101_RIGHT 1 - GCTTGCGCCGAAACCACT

NC_000962.3 1979566 1979587 TBseq_1.0_1102_LEFT 2 + TGAATTCGCTCCCATGTCGAG

NC_000962.3 1981533 1981554 TBseq_1.0_1102_RIGHT 2 - ACCATATTCAGCTCGGGTTGC

NC_000962.3 1984836 1984855 TBseq_1.0_1103_LEFT 1 + TTGGCCCACAAGCTAGACG

NC_000962.3 1986813 1986834 TBseq_1.0_1103_RIGHT 1 - CGGTCAGGTACACGGTATTCC

NC_000962.3 1986662 1986683 TBseq_1.0_1104_LEFT 2 + CGGTACATTCAAGGCCCATCG

NC_000962.3 1988622 1988639 TBseq_1.0_1104_RIGHT 2 - CCCCGATGGTTTGCGGT

NC_000962.3 1987571 1987592 TBseq_1.0_1105_LEFT 1 + CGGACAAGATCTGACCTGGGA

NC_000962.3 1989474 1989493 TBseq_1.0_1105_RIGHT 1 - GCCCAATTGCCTCCGCTAG

NC_000962.3 1992697 1992716 TBseq_1.0_1106_LEFT 2 + CCCGACTCATACCCGCTGT

NC_000962.3 1994609 1994629 TBseq_1.0_1106_RIGHT 2 - GGTCATCACTGCACTCCAGC

NC_000962.3 1994379 1994403 TBseq_1.0_1107_LEFT 1 + GCCTACAATCTGATCCTGTCGAAT

NC_000962.3 1996411 1996433 TBseq_1.0_1107_RIGHT 1 - GGTCTTTAAAATCGCGTTCGCC

NC_000962.3 1995805 1995825 TBseq_1.0_1108_LEFT 2 + GTTCGCTGGCTACTGGATCG

NC_000962.3 1997722 1997740 TBseq_1.0_1108_RIGHT 2 - TGCTCAGCGAGTCCGAAC

NC_000962.3 1997525 1997545 TBseq_1.0_1109_LEFT 1 + GTCCTGCCAATGTCGGATGT

NC_000962.3 1999512 1999531 TBseq_1.0_1109_RIGHT 1 - CACCAGGCCATGAAACGCA

NC_000962.3 1998301 1998320 TBseq_1.0_1110_LEFT 2 + ACGAGCAGCCCATCTTCCA

NC_000962.3 2000214 2000234 TBseq_1.0_1110_RIGHT 2 - GCCACAAGCTCCTTGACCTT

NC_000962.3 2000552 2000572 TBseq_1.0_1111_LEFT 1 + TTGCCGAGGGTTCAATGGTG

NC_000962.3 2002497 2002519 TBseq_1.0_1111_RIGHT 1 - CCAATTGATGTGCAGATTGCCC

NC_000962.3 2002536 2002558 TBseq_1.0_1112_LEFT 2 + CGCACACATCTACGAGATTGGT

NC_000962.3 2004461 2004481 TBseq_1.0_1112_RIGHT 2 - TGTCGATGCGCTCCACAATC

NC_000962.3 2004154 2004173 TBseq_1.0_1113_LEFT 1 + TGCACCTCAAGCTGTCGGT

NC_000962.3 2006205 2006226 TBseq_1.0_1113_RIGHT 1 - GAACCGCGATGTAGCAAGTGT

NC_000962.3 2006029 2006049 TBseq_1.0_1114_LEFT 2 + CCAACGCAAGGTCAGGTTCA

NC_000962.3 2007993 2008014 TBseq_1.0_1114_RIGHT 2 - CACCTTATGGCCATTGACCGT

NC_000962.3 2007722 2007743 TBseq_1.0_1115_LEFT 1 + CCTTCGTCGCGATTCGTTGTC

NC_000962.3 2009627 2009648 TBseq_1.0_1115_RIGHT 1 - GACGAATTGTATTGCGGCACC

NC_000962.3 2009229 2009251 TBseq_1.0_1116_LEFT 2 + AGTTCTTCAAACCCGAGATGGC

NC_000962.3 2011265 2011286 TBseq_1.0_1116_RIGHT 2 - ACCCTTCGAGTAGCTGAGTGG

NC_000962.3 2010962 2010981 TBseq_1.0_1117_LEFT 1 + GCACCGCTAAACCGTTCGA

NC_000962.3 2012948 2012969 TBseq_1.0_1117_RIGHT 1 - CCCACAAGGTGACGGTCAATG

NC_000962.3 2012728 2012747 TBseq_1.0_1118_LEFT 2 + AGTTCATGCGGACCCTCCC

NC_000962.3 2014729 2014749 TBseq_1.0_1118_RIGHT 2 - GAAGAATCTGCGGCCGAACT

NC_000962.3 2014573 2014594 TBseq_1.0_1119_LEFT 1 + CGAGAGATGACGCAGATCACG

NC_000962.3 2016565 2016584 TBseq_1.0_1119_RIGHT 1 - CACTCAAGCTCGTGCACCG

NC_000962.3 2016191 2016210 TBseq_1.0_1120_LEFT 2 + CACCTGCGGTCACACCTTG

NC_000962.3 2018242 2018261 TBseq_1.0_1120_RIGHT 2 - CAGCGACCCGATGCTTGAC

NC_000962.3 2018051 2018070 TBseq_1.0_1121_LEFT 1 + TACCCGGCGCTGAATTTGG

NC_000962.3 2019992 2020012 TBseq_1.0_1121_RIGHT 1 - CATCTGGACATGGTCAGGCC

NC_000962.3 2019846 2019866 TBseq_1.0_1122_LEFT 2 + GGAATTCGGGCGCTACCAAA

NC_000962.3 2021771 2021789 TBseq_1.0_1122_RIGHT 2 - ACACGAGATTGCACGCCG

NC_000962.3 2021571 2021591 TBseq_1.0_1123_LEFT 1 + GTCGGACGACATTGAAGGCG

NC_000962.3 2023579 2023599 TBseq_1.0_1123_RIGHT 1 - GGTTCGCATCTAGCCCGTTT

NC_000962.3 2023368 2023390 TBseq_1.0_1124_LEFT 2 + CCTGTTGATGGCAGAAGACACC

NC_000962.3 2025279 2025299 TBseq_1.0_1124_RIGHT 2 - CAGTCCTCCCTCTCCACACA

NC_000962.3 2024478 2024500 TBseq_1.0_1125_LEFT 1 + CAGGTAGTACCAGCCGTTCATG

NC_000962.3 2026453 2026476 TBseq_1.0_1125_RIGHT 1 - GCGATCTCCTGCTTAGTTGTCTT

NC_000962.3 2026446 2026467 TBseq_1.0_1126_LEFT 2 + GCACCCGAAGACAACTAAGCA

NC_000962.3 2028374 2028395 TBseq_1.0_1126_RIGHT 2 - CAAACGACATCTGAGGTGCCT

NC_000962.3 2028329 2028348 TBseq_1.0_1127_LEFT 1 + CTAGCTCGCGTTTCCCAGC

NC_000962.3 2030311 2030337 TBseq_1.0_1127_RIGHT 1 - CTCCTTAGTACTGAATGCCTAGATCG

NC_000962.3 2029874 2029897 TBseq_1.0_1128_LEFT 2 + GCTGGGATTAATTGGGAGAGGAA

NC_000962.3 2031858 2031876 TBseq_1.0_1128_RIGHT 2 - AGATAGCCAGCCACGCCT

NC_000962.3 2031588 2031610 TBseq_1.0_1129_LEFT 1 + CAATGGAGGAGATGCTAGAGGC

NC_000962.3 2033638 2033658 TBseq_1.0_1129_RIGHT 1 - CTTGCGGAATAGCGGACTGT

NC_000962.3 2033271 2033289 TBseq_1.0_1130_LEFT 2 + TCGGTGCGTGATGTGCTG

NC_000962.3 2035187 2035209 TBseq_1.0_1130_RIGHT 2 - CGATTGATGATTTGGTACGCCG

NC_000962.3 2034848 2034871 TBseq_1.0_1131_LEFT 1 + AGCAGAACCCGATTTTTGATCCC

NC_000962.3 2036838 2036859 TBseq_1.0_1131_RIGHT 1 - CACACATCTTGGGATCCAGCC

NC_000962.3 2036605 2036625 TBseq_1.0_1132_LEFT 2 + TGGTCATCCCCAGTCGGAAC

NC_000962.3 2038528 2038549 TBseq_1.0_1132_RIGHT 2 - ACCGACGAAAAGAGTGCCTAC

NC_000962.3 2037516 2037534 TBseq_1.0_1133_LEFT 1 + AAACGATTGAGGCGCGCA

NC_000962.3 2039405 2039425 TBseq_1.0_1133_RIGHT 1 - GCACACTTTCATCCGATGCG

NC_000962.3 2044843 2044864 TBseq_1.0_1134_LEFT 2 + TGCTTGAGTTGGTTGATGCCC

NC_000962.3 2046864 2046883 TBseq_1.0_1134_RIGHT 2 - GCGGTCGTATGGATTCCCC

NC_000962.3 2044896 2044918 TBseq_1.0_1135_LEFT 1 + AATTAGTTTCGTCCCCAGACCG

NC_000962.3 2046963 2046983 TBseq_1.0_1135_RIGHT 1 - GGTGACACGGGGTTCACATC

NC_000962.3 2047621 2047641 TBseq_1.0_1136_LEFT 2 + CGCGGATATAGGCCACTGAC

NC_000962.3 2049656 2049676 TBseq_1.0_1136_RIGHT 2 - CGCAGAGAAAAGCACTGTCG

NC_000962.3 2047875 2047895 TBseq_1.0_1137_LEFT 1 + ATCCGAGATCGACGACCGTT

NC_000962.3 2049850 2049871 TBseq_1.0_1137_RIGHT 1 - CGTAGTTCTGCCCACCAACTG

NC_000962.3 2051151 2051173 TBseq_1.0_1138_LEFT 2 + AGGACTTTCGAGATGGCACTTG

NC_000962.3 2053141 2053159 TBseq_1.0_1138_RIGHT 2 - ACGCCGTTAGCACACAGC

NC_000962.3 2052923 2052945 TBseq_1.0_1139_LEFT 1 + CAGGACGCGATGCAATTACAAC

NC_000962.3 2054934 2054954 TBseq_1.0_1139_RIGHT 1 - CAGCATCTGCAAGGACTGGC

NC_000962.3 2054698 2054717 TBseq_1.0_1140_LEFT 2 + TTCTGGTCTCGACGCCGTT

NC_000962.3 2056595 2056615 TBseq_1.0_1140_RIGHT 2 - GCGGTCTCGATGCTTTCCAG

NC_000962.3 2056379 2056399 TBseq_1.0_1141_LEFT 1 + TCTAACAGCACCGACCGGAT

NC_000962.3 2058409 2058432 TBseq_1.0_1141_RIGHT 1 - ACTGGACACATAGCGGTATACGG

NC_000962.3 2058025 2058045 TBseq_1.0_1142_LEFT 2 + AGTGAGAAGGGGGATTCGGG

NC_000962.3 2059963 2059985 TBseq_1.0_1142_RIGHT 2 - CGGGAAAGTAACTACGCTCGAA

NC_000962.3 2059224 2059244 TBseq_1.0_1143_LEFT 1 + GCACGACACCTGTAGCAGTC

NC_000962.3 2061138 2061157 TBseq_1.0_1143_RIGHT 1 - CTCCCGGCGTCTTACCTGA

NC_000962.3 2060943 2060963 TBseq_1.0_1144_LEFT 2 + GTCCAATATCGCCCAGGACG

NC_000962.3 2062955 2062976 TBseq_1.0_1144_RIGHT 2 - CTGGAGTTTGCGCTCTACCAA

NC_000962.3 2062730 2062750 TBseq_1.0_1145_LEFT 1 + TCACTCCCCAGAGATCGACG

NC_000962.3 2064783 2064804 TBseq_1.0_1145_RIGHT 1 - GCTCATATCGGCAGCCTATCG

NC_000962.3 2064630 2064651 TBseq_1.0_1146_LEFT 2 + CAGATGGCACTGATCGTCCAG

NC_000962.3 2066612 2066632 TBseq_1.0_1146_RIGHT 2 - CTCCGGTTCTTTTCGGTGCT

NC_000962.3 2066464 2066484 TBseq_1.0_1147_LEFT 1 + GCACGGTTGTCCACGAATTG

NC_000962.3 2068443 2068461 TBseq_1.0_1147_RIGHT 1 - CGGTTACGGTGCGCAACA

NC_000962.3 2068271 2068290 TBseq_1.0_1148_LEFT 2 + CCGATGGCAACCGACGAAA

NC_000962.3 2070275 2070295 TBseq_1.0_1148_RIGHT 2 - GCGGTCGAGGATGATTTGCT

NC_000962.3 2069831 2069848 TBseq_1.0_1149_LEFT 1 + CTGGATACGCGGCGGTT

NC_000962.3 2071785 2071804 TBseq_1.0_1149_RIGHT 1 - CCTGTGCACCACCAGGAAT

NC_000962.3 2071422 2071440 TBseq_1.0_1150_LEFT 2 + CTGAACGCGTTGCAGGCA

NC_000962.3 2073363 2073385 TBseq_1.0_1150_RIGHT 2 - CGCGGTAGAACGTAAGGAGATC

NC_000962.3 2072931 2072950 TBseq_1.0_1151_LEFT 1 + GTCGGGAGTCTCAACGGCA

NC_000962.3 2074993 2075012 TBseq_1.0_1151_RIGHT 1 - CGGAATCGTCGGGGAACAG

NC_000962.3 2074789 2074811 TBseq_1.0_1152_LEFT 2 + GCGTATGCTCTCTAACACTCGG

NC_000962.3 2076830 2076850 TBseq_1.0_1152_RIGHT 2 - ATGTGTTGCTCGCGAGTCTG

NC_000962.3 2076449 2076468 TBseq_1.0_1153_LEFT 1 + AAGCCGCTGGGTATCGAGA

NC_000962.3 2078354 2078374 TBseq_1.0_1153_RIGHT 1 - GCCACCGGAAAACTCATCGT

NC_000962.3 2078201 2078222 TBseq_1.0_1154_LEFT 2 + CGCCTTGACGAGTATTACCCG

NC_000962.3 2080181 2080200 TBseq_1.0_1154_RIGHT 2 - AACCGGCCGTCCAAATCAC

NC_000962.3 2079955 2079975 TBseq_1.0_1155_LEFT 1 + TCCAGTTCGTTCGACTTCCG

NC_000962.3 2081933 2081950 TBseq_1.0_1155_RIGHT 1 - GCAACGCCCTGCACGAA

NC_000962.3 2081680 2081700 TBseq_1.0_1156_LEFT 2 + AATTGTGCGGGCTGATCAGG

NC_000962.3 2083612 2083630 TBseq_1.0_1156_RIGHT 2 - TGATGGGCGCACGACCTA

NC_000962.3 2083341 2083361 TBseq_1.0_1157_LEFT 1 + GGCTTGTGCAAATACCTGGC

NC_000962.3 2085244 2085265 TBseq_1.0_1157_RIGHT 1 - GCCACCATCGAACAATTGCTG

NC_000962.3 2084767 2084784 TBseq_1.0_1158_LEFT 2 + ATCGTCACCGGCCCTGT

NC_000962.3 2086727 2086746 TBseq_1.0_1158_RIGHT 2 - ATGCCTACCGCCAGTTCCT

NC_000962.3 2085748 2085767 TBseq_1.0_1159_LEFT 1 + TTCGCAGGTAAACGCCACC

NC_000962.3 2087717 2087735 TBseq_1.0_1159_RIGHT 1 - CGCCATGAGTTCCCGACA

NC_000962.3 2087535 2087554 TBseq_1.0_1160_LEFT 2 + GGTGACTAGGCGTTCACCG

NC_000962.3 2089548 2089567 TBseq_1.0_1160_RIGHT 2 - TACGCTGTAACCGCTGGGA

NC_000962.3 2087934 2087955 TBseq_1.0_1161_LEFT 1 + ACGGGTGTATGAATCCAGCTC

NC_000962.3 2089909 2089932 TBseq_1.0_1161_RIGHT 1 - GGATATCTGCACATCAAGGACGT

NC_000962.3 2089675 2089695 TBseq_1.0_1162_LEFT 2 + GTCAGTCAACGATGCGTCCC

NC_000962.3 2091588 2091605 TBseq_1.0_1162_RIGHT 2 - AACGGAGCGGCGAACTG

NC_000962.3 2090667 2090685 TBseq_1.0_1163_LEFT 1 + GTTGGCGCCGATCAGCAA

NC_000962.3 2092571 2092590 TBseq_1.0_1163_RIGHT 1 - GAGACCTGATGCGTGACCG

NC_000962.3 2092203 2092224 TBseq_1.0_1164_LEFT 2 + GCGATACTGCGGGTTTGAAGT

NC_000962.3 2094167 2094188 TBseq_1.0_1164_RIGHT 2 - CGTTGTACGCCTCCAAGATCG

NC_000962.3 2093815 2093832 TBseq_1.0_1165_LEFT 1 + AAGAAGTCGCGCTGGGC

NC_000962.3 2095888 2095909 TBseq_1.0_1165_RIGHT 1 - CCTACATCACCGTCTTTGCGT

NC_000962.3 2095562 2095583 TBseq_1.0_1166_LEFT 2 + AAACGCCTCCAGGACAAGATC

NC_000962.3 2097542 2097562 TBseq_1.0_1166_RIGHT 2 - CCCTCCATCACATCGTCACG

NC_000962.3 2097291 2097310 TBseq_1.0_1167_LEFT 1 + GGCCTTAACCCGCTCGAAA

NC_000962.3 2099329 2099348 TBseq_1.0_1167_RIGHT 1 - ATCGCCCCACCTTTGAGCA

NC_000962.3 2099141 2099161 TBseq_1.0_1168_LEFT 2 + ACTGGAAGGTGACCCGTCTG

NC_000962.3 2101142 2101163 TBseq_1.0_1168_RIGHT 2 - CGATCACCCGGATTCTCATGG

NC_000962.3 2100845 2100866 TBseq_1.0_1169_LEFT 1 + CAAGACTGACCTGGCTGCATT

NC_000962.3 2102853 2102874 TBseq_1.0_1169_RIGHT 1 - CAAGTGGCCACCGGGATTATC

NC_000962.3 2102573 2102594 TBseq_1.0_1170_LEFT 2 + GCTCGAAAGCGCTCAATATGC

NC_000962.3 2104463 2104481 TBseq_1.0_1170_RIGHT 2 - GTGCAATCCGTGGGCGAT

NC_000962.3 2104142 2104161 TBseq_1.0_1171_LEFT 1 + GGTTCAGCCGAAGTGAGCC

NC_000962.3 2106185 2106204 TBseq_1.0_1171_RIGHT 1 - CGGCAGCGAGACAAAGGTC

NC_000962.3 2105823 2105843 TBseq_1.0_1172_LEFT 2 + GGGATCGTGTTCGTGGCAAT

NC_000962.3 2107813 2107830 TBseq_1.0_1172_RIGHT 2 - GCAACGGTCACCAGGCT

NC_000962.3 2107671 2107690 TBseq_1.0_1173_LEFT 1 + GCCAGTTCATGAGCCGACC

NC_000962.3 2109567 2109585 TBseq_1.0_1173_RIGHT 1 - CTGCCACGCACTCATCGT

NC_000962.3 2109207 2109227 TBseq_1.0_1174_LEFT 2 + ACGGCAAGACGGGTATAGGT

NC_000962.3 2111232 2111253 TBseq_1.0_1174_RIGHT 2 - CTGGTGCTGGTACTGACGAAC

NC_000962.3 2111087 2111106 TBseq_1.0_1175_LEFT 1 + AGACGAGCCCGGATTTCCA

NC_000962.3 2113042 2113063 TBseq_1.0_1175_RIGHT 1 - ACACCGTAGCTGTTTCATCGG

NC_000962.3 2112722 2112744 TBseq_1.0_1176_LEFT 2 + CGAGGAATCCCGAAAACGAATC

NC_000962.3 2114691 2114711 TBseq_1.0_1176_RIGHT 2 - ATCGGATGTCCAAACACGGG

NC_000962.3 2114549 2114571 TBseq_1.0_1177_LEFT 1 + ACCTCACTTGGGTTTTCCTACG

NC_000962.3 2116456 2116477 TBseq_1.0_1177_RIGHT 1 - CATAAGCTTGGTGTAGGGCCA

NC_000962.3 2116160 2116179 TBseq_1.0_1178_LEFT 2 + AGTGGACTGTGCAGGACGA

NC_000962.3 2118210 2118230 TBseq_1.0_1178_RIGHT 2 - TCTCCCTCCAACCCTTCTGG

NC_000962.3 2117979 2117998 TBseq_1.0_1179_LEFT 1 + CGCAGCTGGGAGCAATTGA

NC_000962.3 2120008 2120027 TBseq_1.0_1179_RIGHT 1 - GCTCAAGATGCGTGACGGG

NC_000962.3 2119321 2119341 TBseq_1.0_1180_LEFT 2 + ACGAACTGATTACCACGCCG

NC_000962.3 2121344 2121363 TBseq_1.0_1180_RIGHT 2 - TTAAACTCGCCGGCACCAC

NC_000962.3 2121107 2121128 TBseq_1.0_1181_LEFT 1 + GTGGCTGAGCTCTCGTCATAC

NC_000962.3 2123086 2123106 TBseq_1.0_1181_RIGHT 1 - CTGCTCCAGTTCAACAGGCC

NC_000962.3 2122896 2122915 TBseq_1.0_1182_LEFT 2 + GTCACGCAGGATTGTCGGG

NC_000962.3 2124827 2124845 TBseq_1.0_1182_RIGHT 2 - CGACGCCGTTTGCTGAGT

NC_000962.3 2124595 2124612 TBseq_1.0_1183_LEFT 1 + CCGCAACGGTTGGCAGT

NC_000962.3 2126634 2126654 TBseq_1.0_1183_RIGHT 1 - TCAACAGCCCGACAATGGTC

NC_000962.3 2126462 2126480 TBseq_1.0_1184_LEFT 2 + ACGTGCCGGTTTCGATCG

NC_000962.3 2128423 2128444 TBseq_1.0_1184_RIGHT 2 - GAGGAATTCGACTTCGTGGCC

NC_000962.3 2128265 2128284 TBseq_1.0_1185_LEFT 1 + GCATCGATCTGTCCGCCTT

NC_000962.3 2130244 2130264 TBseq_1.0_1185_RIGHT 1 - CCCCAGGTAGTGCACACTCA

NC_000962.3 2130097 2130117 TBseq_1.0_1186_LEFT 2 + ATCTGCACAAGGCCAATCCG

NC_000962.3 2132052 2132072 TBseq_1.0_1186_RIGHT 2 - TAACGTCGACGGGTTCAACG

NC_000962.3 2131711 2131732 TBseq_1.0_1187_LEFT 1 + CGGACTTCGTCGTAAAAGGGC

NC_000962.3 2133613 2133637 TBseq_1.0_1187_RIGHT 1 - CGGACAAGATCTGGACATTGATCG

NC_000962.3 2133413 2133433 TBseq_1.0_1188_LEFT 2 + ATTGTTCACCGGTCTGTCGC

NC_000962.3 2135308 2135328 TBseq_1.0_1188_RIGHT 2 - CCAGCAGTTCATCTACGCCG

NC_000962.3 2135145 2135164 TBseq_1.0_1189_LEFT 1 + ACCAGCTTGGGGATCTGCT

NC_000962.3 2137124 2137142 TBseq_1.0_1189_RIGHT 1 - GATACGTGGCGTCGGCTT

NC_000962.3 2136869 2136889 TBseq_1.0_1190_LEFT 2 + CAAACGATGGCGCAGGTTAC

NC_000962.3 2138860 2138880 TBseq_1.0_1190_RIGHT 2 - GAGAGCCGTGGGGATTGACT

NC_000962.3 2138545 2138565 TBseq_1.0_1191_LEFT 1 + GGGTTGGTTGGGGTCAAAGC

NC_000962.3 2140579 2140597 TBseq_1.0_1191_RIGHT 1 - TGTCGCCGCGGATGATGT

NC_000962.3 2140368 2140387 TBseq_1.0_1192_LEFT 2 + CTCGGAATCGCGATGGCAG

NC_000962.3 2142302 2142324 TBseq_1.0_1192_RIGHT 2 - GAGGGTTGATCAGGGAGTAGGT

NC_000962.3 2142150 2142169 TBseq_1.0_1193_LEFT 1 + GGCAGCTGCAGTCCAAACT

NC_000962.3 2144145 2144165 TBseq_1.0_1193_RIGHT 1 - CGCTGATGAGGCTGGCATAC

NC_000962.3 2143875 2143895 TBseq_1.0_1194_LEFT 2 + CGGTCAGGTAGGGCAGTAGT

NC_000962.3 2145948 2145967 TBseq_1.0_1194_RIGHT 2 - TGTTCGCACCCTCGGTACA

NC_000962.3 2145806 2145826 TBseq_1.0_1195_LEFT 1 + GCCCACACAATCGAACCCTC

NC_000962.3 2147781 2147801 TBseq_1.0_1195_RIGHT 1 - CCGCAGATCGTGATGTGTGC

NC_000962.3 2147607 2147627 TBseq_1.0_1196_LEFT 2 + CTGTGAATGTGGGGAGCCTG

NC_000962.3 2149515 2149535 TBseq_1.0_1196_RIGHT 2 - CCGCGACTACCGATCATGGT

NC_000962.3 2149287 2149308 TBseq_1.0_1197_LEFT 1 + GGCACCCTGTACGAAGACTTG

NC_000962.3 2151198 2151219 TBseq_1.0_1197_RIGHT 1 - ACAGCGTATGCACATGATCCC

NC_000962.3 2151018 2151037 TBseq_1.0_1198_LEFT 2 + TGACCTGCATAGCGGCCTA

NC_000962.3 2152974 2152993 TBseq_1.0_1198_RIGHT 2 - TGGGGATCGAACAAGCCCA

NC_000962.3 2152657 2152677 TBseq_1.0_1199_LEFT 1 + GGTTACCCATCCGCTTCCAA

NC_000962.3 2154679 2154699 TBseq_1.0_1199_RIGHT 1 - GCTAGTTTCGACCGCATGGG

NC_000962.3 2154492 2154514 TBseq_1.0_1200_LEFT 2 + AAGGACACTTTGATGTTCCCCG

NC_000962.3 2156461 2156481 TBseq_1.0_1200_RIGHT 2 - GAAACGATTTTCGGTGCCGT

NC_000962.3 2156178 2156201 TBseq_1.0_1201_LEFT 1 + CGAACAATCAGGACATAGACCCC

NC_000962.3 2158142 2158162 TBseq_1.0_1201_RIGHT 1 - GTTCGCCGAAGCTCCTGAAG

NC_000962.3 2157884 2157904 TBseq_1.0_1202_LEFT 2 + GTGTCTCTCGACTGTCACCG

NC_000962.3 2159852 2159870 TBseq_1.0_1202_RIGHT 2 - TGGCGACGGACAAACTGC

NC_000962.3 2159427 2159447 TBseq_1.0_1203_LEFT 1 + GACCAAGCGGAGGTGTTCTG

NC_000962.3 2161431 2161450 TBseq_1.0_1203_RIGHT 1 - CCGAAAATGCCTTGGCGCT

NC_000962.3 2160924 2160943 TBseq_1.0_1204_LEFT 2 + CTGCAGATGAGCGAGCGAC

NC_000962.3 2162832 2162851 TBseq_1.0_1204_RIGHT 2 - GACGCCGTCTGACAGCATC

NC_000962.3 2170674 2170695 TBseq_1.0_1205_LEFT 1 + CTCGTTCGTAGCGCATGAGAG

NC_000962.3 2172597 2172617 TBseq_1.0_1205_RIGHT 1 - CGGGGAGGCATTCATCAACC

NC_000962.3 2172117 2172137 TBseq_1.0_1206_LEFT 2 + TATCCGATTGTGCCGTTCGC

NC_000962.3 2174146 2174163 TBseq_1.0_1206_RIGHT 2 - TGCGTGTCGTGTGCTGG

NC_000962.3 2173875 2173898 TBseq_1.0_1207_LEFT 1 + CTGAACGCTGAGCAAATTCGATG

NC_000962.3 2175923 2175943 TBseq_1.0_1207_RIGHT 1 - GAAATCGAGCAGTGGGGTGG

NC_000962.3 2175575 2175596 TBseq_1.0_1208_LEFT 2 + GAGCTAACGGCAAGTCTGAGG

NC_000962.3 2177479 2177500 TBseq_1.0_1208_RIGHT 2 - AAGTGCCGTATCAAGACGCTC

NC_000962.3 2177201 2177222 TBseq_1.0_1209_LEFT 1 + TCATCGAGCGCAACATCAAGT

NC_000962.3 2179187 2179205 TBseq_1.0_1209_RIGHT 1 - GCCACTGCCACGGTCAAT

NC_000962.3 2178972 2178991 TBseq_1.0_1210_LEFT 2 + AGCAGATCCTCCATGCCGT

NC_000962.3 2180945 2180965 TBseq_1.0_1210_RIGHT 2 - GAGCTGGGTGGGATCGACAT

NC_000962.3 2180566 2180586 TBseq_1.0_1211_LEFT 1 + CAACGGAATCTTGGGCTCCC

NC_000962.3 2182513 2182530 TBseq_1.0_1211_RIGHT 1 - AAACCATGCGCCGCAGC

NC_000962.3 2182233 2182253 TBseq_1.0_1212_LEFT 2 + TTGTCCTGGTGCACAATCCG

NC_000962.3 2184170 2184189 TBseq_1.0_1212_RIGHT 2 - GGTTCCTTCCAGGCGGTCA

NC_000962.3 2183994 2184014 TBseq_1.0_1213_LEFT 1 + GGCACCGTGGATCTGAAGAG

NC_000962.3 2185892 2185911 TBseq_1.0_1213_RIGHT 1 - ACCTGCTCGGAATCGACCT

NC_000962.3 2185727 2185747 TBseq_1.0_1214_LEFT 2 + CATTCGTCACCGTCGAGCAC

NC_000962.3 2187679 2187697 TBseq_1.0_1214_RIGHT 2 - ACGCCGAGCATGAACCGA

NC_000962.3 2187228 2187250 TBseq_1.0_1215_LEFT 1 + AGCCAATACGCACATGAGAGTG

NC_000962.3 2189201 2189221 TBseq_1.0_1215_RIGHT 1 - CCCGTAGAGCAGGTAGACCG

NC_000962.3 2188990 2189012 TBseq_1.0_1216_LEFT 2 + TGCGAGTTCATCATCAGGTTGC

NC_000962.3 2191029 2191049 TBseq_1.0_1216_RIGHT 2 - AGGATTCGATGGACCTGCGA

NC_000962.3 2190780 2190798 TBseq_1.0_1217_LEFT 1 + GTACATGGACCGCGTGCT

NC_000962.3 2192818 2192838 TBseq_1.0_1217_RIGHT 1 - GTGTCTCGGTCACACATGGG

NC_000962.3 2192411 2192433 TBseq_1.0_1218_LEFT 2 + GTGTTCTGGAGCGAATTGTCCA

NC_000962.3 2194459 2194479 TBseq_1.0_1218_RIGHT 2 - GGCAATGTCTCGCGTGATCT

NC_000962.3 2193974 2193994 TBseq_1.0_1219_LEFT 1 + GGACTTCGATCGGGTCATCG

NC_000962.3 2195926 2195949 TBseq_1.0_1219_RIGHT 1 - CGTCATAGGCTCGGAATGATCAG

NC_000962.3 2195612 2195632 TBseq_1.0_1220_LEFT 2 + GCTTTTGCTGCATCCTCCGG

NC_000962.3 2197598 2197617 TBseq_1.0_1220_RIGHT 2 - TGCACGCACCTGCTCTATG

NC_000962.3 2197390 2197410 TBseq_1.0_1221_LEFT 1 + CAAGTACTGCGACCGTAGCC

NC_000962.3 2199410 2199433 TBseq_1.0_1221_RIGHT 1 - CGGACAAGGGTTCTCTCTTTGAA

NC_000962.3 2199264 2199283 TBseq_1.0_1222_LEFT 2 + GTTAGGTTGCTCTCGGCGG

NC_000962.3 2201183 2201203 TBseq_1.0_1222_RIGHT 2 - GCTAGGGGCTGCATGATGAG

NC_000962.3 2200958 2200977 TBseq_1.0_1223_LEFT 1 + AACGTGGTGTCCGCTTTGC

NC_000962.3 2202997 2203018 TBseq_1.0_1223_RIGHT 1 - GACGTATTCGCGGATGTACGG

NC_000962.3 2202778 2202800 TBseq_1.0_1224_LEFT 2 + GCATTTGTGGTGCGGATTTCTT

NC_000962.3 2204825 2204846 TBseq_1.0_1224_RIGHT 2 - CTATCGCGCTGGCTATCGTAC

NC_000962.3 2204085 2204104 TBseq_1.0_1225_LEFT 1 + AGCTGGGTTTCACGGTCCT

NC_000962.3 2206005 2206028 TBseq_1.0_1225_RIGHT 1 - TTTTCTTCCAAACAGGCCATCCT

NC_000962.3 2205588 2205607 TBseq_1.0_1226_LEFT 2 + CGCAAGAGGTTACCTGCCC

NC_000962.3 2207564 2207586 TBseq_1.0_1226_RIGHT 2 - TGATGTCCATAGCCAATACGCG

NC_000962.3 2207217 2207236 TBseq_1.0_1227_LEFT 1 + CTGCAGACAAAGCGCGAGT

NC_000962.3 2209142 2209164 TBseq_1.0_1227_RIGHT 1 - ACACCAGCATGATCATCAGAGC

NC_000962.3 2208801 2208819 TBseq_1.0_1228_LEFT 2 + ACACCCGCGAGATTCAGC

NC_000962.3 2210803 2210823 TBseq_1.0_1228_RIGHT 2 - CGGGGTTGATGGAGATCCTG

NC_000962.3 2210430 2210448 TBseq_1.0_1229_LEFT 1 + ACCAACACCGGATGGGGA

NC_000962.3 2212505 2212524 TBseq_1.0_1229_RIGHT 1 - GACGACAGCGACTCACCCA

NC_000962.3 2212110 2212129 TBseq_1.0_1230_LEFT 2 + GACCTTTGCCGATACGCCG

NC_000962.3 2214134 2214154 TBseq_1.0_1230_RIGHT 2 - GCGATCATCACCAGGGTCAG

NC_000962.3 2213883 2213903 TBseq_1.0_1231_LEFT 1 + TTGTGCGTGCAATACCTGGC

NC_000962.3 2215949 2215968 TBseq_1.0_1231_RIGHT 1 - CGTGTGTCTGCAATTGGCC

NC_000962.3 2215714 2215735 TBseq_1.0_1232_LEFT 2 + GGCAATACCTCACGAGAACCT

NC_000962.3 2217766 2217786 TBseq_1.0_1232_RIGHT 2 - ACCAGATAGGCGACCACGTC

NC_000962.3 2217520 2217542 TBseq_1.0_1233_LEFT 1 + CTGCTGTTCGTAAACCAGACCA

NC_000962.3 2219544 2219564 TBseq_1.0_1233_RIGHT 1 - GCTCACAACTCGCAGCGTAG

NC_000962.3 2219345 2219366 TBseq_1.0_1234_LEFT 2 + GCTCTTGCTGGAACTTCTGGT

NC_000962.3 2221329 2221349 TBseq_1.0_1234_RIGHT 2 - GGTCATAGCTGTGGTCGTGG

NC_000962.3 2221106 2221127 TBseq_1.0_1235_LEFT 1 + CCCGGATAGCCCTGAAACTTG

NC_000962.3 2223113 2223133 TBseq_1.0_1235_RIGHT 1 - TCCACAAGCTGGGTTTCTGC

NC_000962.3 2222719 2222738 TBseq_1.0_1236_LEFT 2 + ACAGCACGCCGATAAAGCC

NC_000962.3 2224788 2224809 TBseq_1.0_1236_RIGHT 2 - GGTCGGAACAGAACCCTTACC

NC_000962.3 2224097 2224120 TBseq_1.0_1237_LEFT 1 + TCTGAGTCGACGACAAACATAGG

NC_000962.3 2226080 2226103 TBseq_1.0_1237_RIGHT 1 - CTGTGGCGCTGAATATCAAAGAC

NC_000962.3 2225975 2225995 TBseq_1.0_1238_LEFT 2 + CGACTCCAAAAACGCCAGCT

NC_000962.3 2227956 2227978 TBseq_1.0_1238_RIGHT 2 - CGTATGTTCAGTCGGGGATGAC

NC_000962.3 2226175 2226195 TBseq_1.0_1239_LEFT 1 + AGATGTCAAAGGCAGCGTCT

NC_000962.3 2228174 2228196 TBseq_1.0_1239_RIGHT 1 - GGTCATCGATTTGTCCACCTCG

NC_000962.3 2227985 2228006 TBseq_1.0_1240_LEFT 2 + CGCCATAATATTGCCGCCTCC

NC_000962.3 2229992 2230012 TBseq_1.0_1240_RIGHT 2 - ACGCAATCGACACTAGGCAG

NC_000962.3 2229721 2229741 TBseq_1.0_1241_LEFT 1 + CCCGACATGGCTTTTCCCTG

NC_000962.3 2231614 2231632 TBseq_1.0_1241_RIGHT 1 - CACCGGCACTGCGATCAA

NC_000962.3 2231268 2231288 TBseq_1.0_1242_LEFT 2 + TCCGTCTTGCACACATGGAA

NC_000962.3 2233301 2233320 TBseq_1.0_1242_RIGHT 2 - GATGGCGGAGGGAGTGTTC

NC_000962.3 2233090 2233110 TBseq_1.0_1243_LEFT 1 + ACCTCAACCATGCAGGCTTG

NC_000962.3 2235007 2235027 TBseq_1.0_1243_RIGHT 1 - GCCACCCAAGACACCTGATC

NC_000962.3 2234737 2234756 TBseq_1.0_1244_LEFT 2 + TGTCGATCTGGCCCGTGAG

NC_000962.3 2236812 2236832 TBseq_1.0_1244_RIGHT 2 - GCATTCTCCGGAGTGTTGCT

NC_000962.3 2236560 2236580 TBseq_1.0_1245_LEFT 1 + GTCGCGTATTCCTCCAAGCC

NC_000962.3 2238541 2238561 TBseq_1.0_1245_RIGHT 1 - AGGTGATGTCCGTGAGCTTG

NC_000962.3 2238173 2238195 TBseq_1.0_1246_LEFT 2 + ACCGTCATGAAACAGACACCAC

NC_000962.3 2240151 2240171 TBseq_1.0_1246_RIGHT 2 - CTGACGCCGACAATCACCTC

NC_000962.3 2240002 2240021 TBseq_1.0_1247_LEFT 1 + CCCGTCCGAACCGTGATTG

NC_000962.3 2241990 2242009 TBseq_1.0_1247_RIGHT 1 - GGCAAACACGCTGGCTGTA

NC_000962.3 2241823 2241841 TBseq_1.0_1248_LEFT 2 + AGTGCCGGCATTGCGGTA

NC_000962.3 2243743 2243765 TBseq_1.0_1248_RIGHT 2 - GTCACACATACAATCAGGCCCA

NC_000962.3 2243382 2243402 TBseq_1.0_1249_LEFT 1 + GGGGTCGATGAGCTTTTCGG

NC_000962.3 2245369 2245388 TBseq_1.0_1249_RIGHT 1 - GTCGTTCATGGGCCCTCCT

NC_000962.3 2245208 2245227 TBseq_1.0_1250_LEFT 2 + ATGCGTCCAGGGTTTGTGG

NC_000962.3 2247167 2247186 TBseq_1.0_1250_RIGHT 2 - ACCCTTCGGTTTCGATGCG

NC_000962.3 2247010 2247029 TBseq_1.0_1251_LEFT 1 + GGCAGAGGTCCATCCCCAT

NC_000962.3 2248962 2248981 TBseq_1.0_1251_RIGHT 1 - CTTTCGTCAGCCGGCACTT

NC_000962.3 2248467 2248486 TBseq_1.0_1252_LEFT 2 + CCGGTCTGGCGATGACGAT

NC_000962.3 2250428 2250451 TBseq_1.0_1252_RIGHT 2 - GGATTGAACACATGGTCGACGAA

NC_000962.3 2250200 2250222 TBseq_1.0_1253_LEFT 1 + GTAGTCCCCAAGATCTTTGCGG

NC_000962.3 2252170 2252189 TBseq_1.0_1253_RIGHT 1 - CAATGGTCTTCGCCGGTCC

NC_000962.3 2252018 2252037 TBseq_1.0_1254_LEFT 2 + CGTCAATGTGACCGGACCG

NC_000962.3 2254015 2254034 TBseq_1.0_1254_RIGHT 2 - CGAGTTTGCAGTGCGACCA

NC_000962.3 2253817 2253836 TBseq_1.0_1255_LEFT 1 + TCCCCAACTGGTTGTCGCT

NC_000962.3 2255809 2255832 TBseq_1.0_1255_RIGHT 1 - CACAAACGGAAACTCAAGTGGTC

NC_000962.3 2255655 2255674 TBseq_1.0_1256_LEFT 2 + TCCGATATCGCCGACGTCC

NC_000962.3 2257565 2257585 TBseq_1.0_1256_RIGHT 2 - GGCCTTAGCGGGTTGATCAC

NC_000962.3 2257400 2257421 TBseq_1.0_1257_LEFT 1 + GACAACTCCACCAACTCGACG

NC_000962.3 2259358 2259378 TBseq_1.0_1257_RIGHT 1 - CCGATGCGAAGGGTTTCACG

NC_000962.3 2258991 2259011 TBseq_1.0_1258_LEFT 2 + GCGACCATTCTCGGTTAGCA

NC_000962.3 2260879 2260898 TBseq_1.0_1258_RIGHT 2 - GTGGGCGACGAGGTCTTTG

NC_000962.3 2260563 2260582 TBseq_1.0_1259_LEFT 1 + CCACGATCGAGCACAACGC

NC_000962.3 2262555 2262574 TBseq_1.0_1259_RIGHT 1 - TGGCGAATCAAGCTTCCGC

NC_000962.3 2262226 2262246 TBseq_1.0_1260_LEFT 2 + CCCGTCACGTTCAAACCAGG

NC_000962.3 2264186 2264206 TBseq_1.0_1260_RIGHT 2 - CCTCGAAACCTGTTCCCGTG

NC_000962.3 2263799 2263821 TBseq_1.0_1261_LEFT 1 + CTATCGGTGGGACCCTGATACC

NC_000962.3 2265816 2265835 TBseq_1.0_1261_RIGHT 1 - CGAGGACGACGTTGGCATC

NC_000962.3 2265672 2265694 TBseq_1.0_1262_LEFT 2 + AGTCCTGATGATCTGGTGGTCA

NC_000962.3 2267625 2267645 TBseq_1.0_1262_RIGHT 2 - GGCTACTGTTTCAACGGCCC

NC_000962.3 2267289 2267308 TBseq_1.0_1263_LEFT 1 + CAGAATGCGGACCTCGGAC

NC_000962.3 2269187 2269209 TBseq_1.0_1263_RIGHT 1 - GCACGACGGTCAGTATCTGAAG

NC_000962.3 2268942 2268961 TBseq_1.0_1264_LEFT 2 + ACCCCGGAGAGTTCCTGCT

NC_000962.3 2271006 2271023 TBseq_1.0_1264_RIGHT 2 - AGCGATGGTCGATGCCG

NC_000962.3 2270785 2270805 TBseq_1.0_1265_LEFT 1 + GCCATGTTCAGCCGGATAGG

NC_000962.3 2272824 2272843 TBseq_1.0_1265_RIGHT 1 - GTCGAGAACATGCCGACCG

NC_000962.3 2272685 2272705 TBseq_1.0_1266_LEFT 2 + TTGGATTGCGCTGATCCGTC

NC_000962.3 2274610 2274628 TBseq_1.0_1266_RIGHT 2 - AACGCCGTGTTGCAGGAG

NC_000962.3 2274476 2274496 TBseq_1.0_1267_LEFT 1 + GGGTTAACGTTCGCCCTGTC

NC_000962.3 2276363 2276388 TBseq_1.0_1267_RIGHT 1 - CGAATCATCACTTTGACCATGAACC

NC_000962.3 2276072 2276093 TBseq_1.0_1268_LEFT 2 + GCACGAAACGATACTGCTTGG

NC_000962.3 2278149 2278166 TBseq_1.0_1268_RIGHT 2 - GCAACTGCGCGACATCG

NC_000962.3 2277685 2277702 TBseq_1.0_1269_LEFT 1 + TGCATCGCCGACCAGCT

NC_000962.3 2279692 2279714 TBseq_1.0_1269_RIGHT 1 - TGCCCTCAGAAGTCTCAAAAGC

NC_000962.3 2279198 2279217 TBseq_1.0_1270_LEFT 2 + TCCACAACAGCCAGCCCTG

NC_000962.3 2281137 2281161 TBseq_1.0_1270_RIGHT 2 - GACTGGTACAAGCATTTTCCGAGA

NC_000962.3 2280160 2280180 TBseq_1.0_1271_LEFT 1 + GAATCGGGCGGATGATGTGT

NC_000962.3 2282164 2282185 TBseq_1.0_1271_RIGHT 1 - GGTAGTCAGAAACGCCGCTAG

NC_000962.3 2281869 2281888 TBseq_1.0_1272_LEFT 2 + GGGATATCGGCCCGACTTG

NC_000962.3 2283876 2283896 TBseq_1.0_1272_RIGHT 2 - GCGGACGCCTACTTGTATGG

NC_000962.3 2283642 2283662 TBseq_1.0_1273_LEFT 1 + CCGCTCCAACCAACCCTATC

NC_000962.3 2285666 2285686 TBseq_1.0_1273_RIGHT 1 - TGCTGCAGTTGCGAATTACG

NC_000962.3 2285473 2285490 TBseq_1.0_1274_LEFT 2 + ATGCGACGGGAACAGCG

NC_000962.3 2287433 2287452 TBseq_1.0_1274_RIGHT 2 - TCGGGGCCCTGTATGAGAC

NC_000962.3 2287062 2287081 TBseq_1.0_1275_LEFT 1 + GGGCCACCTTGTGCTTGTT

NC_000962.3 2288980 2289000 TBseq_1.0_1275_RIGHT 1 - TCCATCCCAGTCTGGACACG

NC_000962.3 2288814 2288832 TBseq_1.0_1276_LEFT 2 + CCGTCTGGCGCACACAAT

NC_000962.3 2290782 2290800 TBseq_1.0_1276_RIGHT 2 - TGCGTGTACGTGTCGGTC

NC_000962.3 2290338 2290355 TBseq_1.0_1277_LEFT 1 + ACCAATCGGGAAGGCGC

NC_000962.3 2292230 2292248 TBseq_1.0_1277_RIGHT 1 - ATTTGCAGCCCGGCGAGA

NC_000962.3 2292066 2292085 TBseq_1.0_1278_LEFT 2 + TGGCAGAAACCGGGTAGCT

NC_000962.3 2294044 2294065 TBseq_1.0_1278_RIGHT 2 - CGTACAAGTCGTCCACTCCGA

NC_000962.3 2293827 2293848 TBseq_1.0_1279_LEFT 1 + CAGGAGCGTGACATCCATCAA

NC_000962.3 2295771 2295789 TBseq_1.0_1279_RIGHT 1 - ATGGGGTGCGCAATCTGG

NC_000962.3 2295629 2295651 TBseq_1.0_1280_LEFT 2 + GGAATATCGGCGATCACCTTGG

NC_000962.3 2297524 2297541 TBseq_1.0_1280_RIGHT 2 - AGCGTGGCCAAGGTTCG

NC_000962.3 2297292 2297313 TBseq_1.0_1281_LEFT 1 + ACCAGGTGACCTCGAATAGCC

NC_000962.3 2299192 2299211 TBseq_1.0_1281_RIGHT 1 - GCTGGGCATGGGAATGGGA

NC_000962.3 2298991 2299010 TBseq_1.0_1282_LEFT 2 + CGGTCGTACACCCCAGGAT

NC_000962.3 2300992 2301013 TBseq_1.0_1282_RIGHT 2 - CAGGACCTGGGTTTTGACTCG

NC_000962.3 2300380 2300399 TBseq_1.0_1283_LEFT 1 + GGACAATTGGCCGGTCAGC

NC_000962.3 2302361 2302381 TBseq_1.0_1283_RIGHT 1 - GGCTTTGACGACGACCACAT

NC_000962.3 2301862 2301881 TBseq_1.0_1284_LEFT 2 + CAGATTGCGCACCCCATGA

NC_000962.3 2303829 2303847 TBseq_1.0_1284_RIGHT 2 - AACCAGGCGCGGACCTAT

NC_000962.3 2303619 2303638 TBseq_1.0_1285_LEFT 1 + AGACGGCGTCCAACAAAGC

NC_000962.3 2305637 2305660 TBseq_1.0_1285_RIGHT 1 - TCGTTTGGGATTAGTGGCACTAA

NC_000962.3 2305265 2305285 TBseq_1.0_1286_LEFT 2 + ATTCCCATGCCCAGCCATTG

NC_000962.3 2307189 2307208 TBseq_1.0_1286_RIGHT 2 - GTACAGTCGCGCCATGGAC

NC_000962.3 2306961 2306982 TBseq_1.0_1287_LEFT 1 + TCGCATGCTGGAGTTGATCAA

NC_000962.3 2308989 2309010 TBseq_1.0_1287_RIGHT 1 - TTTCGGACGACACAGAGCAAC

NC_000962.3 2308508 2308529 TBseq_1.0_1288_LEFT 2 + GGTTTTGGACAGGACTAGCCG

NC_000962.3 2310507 2310527 TBseq_1.0_1288_RIGHT 2 - GGCCTGGTGTTCTACGTCTC

NC_000962.3 2309638 2309656 TBseq_1.0_1289_LEFT 1 + ACGGCATGGGCAGGTACT

NC_000962.3 2311599 2311618 TBseq_1.0_1289_RIGHT 1 - CATCGAAGCGCACGTACGA

NC_000962.3 2311576 2311595 TBseq_1.0_1290_LEFT 2 + GTCACTTCGGCCTTGGTGC

NC_000962.3 2313550 2313570 TBseq_1.0_1290_RIGHT 2 - CATGCCCCGTTTAGCGTCTC

NC_000962.3 2313415 2313434 TBseq_1.0_1291_LEFT 1 + GCCATGCCAGAATGCTCCG

NC_000962.3 2315384 2315401 TBseq_1.0_1291_RIGHT 1 - AAGACGACGGGGGTGGT

NC_000962.3 2315151 2315175 TBseq_1.0_1292_LEFT 2 + ACTGTTATCGATAAGGAGGACGGT

NC_000962.3 2317162 2317182 TBseq_1.0_1292_RIGHT 2 - ACCTGGAAGCCTGAGTACGG

NC_000962.3 2316700 2316721 TBseq_1.0_1293_LEFT 1 + CCACTTTGAGCTCTAGGCCAA

NC_000962.3 2318714 2318734 TBseq_1.0_1293_RIGHT 1 - GGGGAGCAAGAACTCGACCT

NC_000962.3 2318464 2318483 TBseq_1.0_1294_LEFT 2 + GGGTATCACTTCGGTGGCG

NC_000962.3 2320439 2320459 TBseq_1.0_1294_RIGHT 2 - CTGCAGGCCCACATCTACCT

NC_000962.3 2320034 2320053 TBseq_1.0_1295_LEFT 1 + AGGTGCTCAACGTTCCAGC

NC_000962.3 2322096 2322115 TBseq_1.0_1295_RIGHT 1 - GTTTTGGTGACGGGCCGAA

NC_000962.3 2321359 2321379 TBseq_1.0_1296_LEFT 2 + GCAATCGAAAACGCCCTGAC

NC_000962.3 2323290 2323308 TBseq_1.0_1296_RIGHT 2 - ATGCTGTGACCGTGTGGG

NC_000962.3 2323038 2323057 TBseq_1.0_1297_LEFT 1 + GTTTCGTCGGATCGGCACA

NC_000962.3 2325109 2325129 TBseq_1.0_1297_RIGHT 1 - CAGGGATGGTTCCACAAGGC

NC_000962.3 2324502 2324522 TBseq_1.0_1298_LEFT 2 + CGGACCGAATGAGGACGTTC

NC_000962.3 2326479 2326503 TBseq_1.0_1298_RIGHT 2 - CTGGACAAACTGATCACCTACACC

NC_000962.3 2326304 2326324 TBseq_1.0_1299_LEFT 1 + GGAGGTAGCCGGTAAATGCC

NC_000962.3 2328209 2328228 TBseq_1.0_1299_RIGHT 1 - ACAGTGACCCGGGTGTTGT

NC_000962.3 2327902 2327921 TBseq_1.0_1300_LEFT 2 + ATGATGGCGGTACCGGGAT

NC_000962.3 2329928 2329948 TBseq_1.0_1300_RIGHT 2 - GACCTACACGTGGTTGCCAG

NC_000962.3 2329079 2329098 TBseq_1.0_1301_LEFT 1 + CCGAGGCGCGAATATGCAT

NC_000962.3 2331135 2331153 TBseq_1.0_1301_RIGHT 1 - ATGACCCGCGCGATGTGA

NC_000962.3 2330984 2331003 TBseq_1.0_1302_LEFT 2 + TAGCGTGAGTGGCGATGGT

NC_000962.3 2332890 2332909 TBseq_1.0_1302_RIGHT 2 - AAGCGGTGTATCGACGTGC

NC_000962.3 2332717 2332734 TBseq_1.0_1303_LEFT 1 + CACGCAGGGGATCGCAA

NC_000962.3 2334679 2334701 TBseq_1.0_1303_RIGHT 1 - CTGTGTGATCGTTCCTGTCTCA

NC_000962.3 2334525 2334544 TBseq_1.0_1304_LEFT 2 + AAGGCCCTGCGATTGACTG

NC_000962.3 2336481 2336499 TBseq_1.0_1304_RIGHT 2 - TGCAACCAGCCGCCTTTC

NC_000962.3 2336120 2336139 TBseq_1.0_1305_LEFT 1 + GCAATCTCAACGGCGTTCC

NC_000962.3 2338081 2338101 TBseq_1.0_1305_RIGHT 1 - CCGCAGTAGGTCGGTTTCGT

NC_000962.3 2337712 2337733 TBseq_1.0_1306_LEFT 2 + CATAACCCTGCACAAGAACGG

NC_000962.3 2339629 2339649 TBseq_1.0_1306_RIGHT 2 - AACGACTGTGGGGATAACGG

NC_000962.3 2338862 2338884 TBseq_1.0_1307_LEFT 1 + CCCTGTTAGCGGTAAACCAAGG

NC_000962.3 2340818 2340839 TBseq_1.0_1307_RIGHT 1 - CCCAGGCAAGGTGATAGTTCG

NC_000962.3 2340243 2340261 TBseq_1.0_1308_LEFT 2 + ACAACAACGCCTACGCCG

NC_000962.3 2342244 2342264 TBseq_1.0_1308_RIGHT 2 - GGCATACGGAATTGCACTGC

NC_000962.3 2342102 2342124 TBseq_1.0_1309_LEFT 1 + CACTGCTGATCGATGTGCTGAA

NC_000962.3 2344052 2344072 TBseq_1.0_1309_RIGHT 1 - GCCCTGTCGTGGTTTCACAT

NC_000962.3 2343546 2343567 TBseq_1.0_1310_LEFT 2 + GTACGGCAGCCTGAAGATGTG

NC_000962.3 2345542 2345562 TBseq_1.0_1310_RIGHT 2 - GGACGAGGTGGGAGAAAGGG

NC_000962.3 2345192 2345212 TBseq_1.0_1311_LEFT 1 + CATGCGTCGGTTCACCTCAG

NC_000962.3 2347224 2347244 TBseq_1.0_1311_RIGHT 1 - GGCGGGTCTGGTGATAACTC

NC_000962.3 2346874 2346895 TBseq_1.0_1312_LEFT 2 + GGTTTCCTCCTTGACCATCCG

NC_000962.3 2348826 2348846 TBseq_1.0_1312_RIGHT 2 - GTTCATCGGCGCGGTTCTAA

NC_000962.3 2348249 2348268 TBseq_1.0_1313_LEFT 1 + AGACCGCCGCTACCCTACT

NC_000962.3 2350256 2350276 TBseq_1.0_1313_RIGHT 1 - GATCTCCTCGGCCGACTACT

NC_000962.3 2350058 2350078 TBseq_1.0_1314_LEFT 2 + GATGACGGCGCAATTGTTCG

NC_000962.3 2351998 2352018 TBseq_1.0_1314_RIGHT 2 - GAACTACCGTTCTCGCTCGA

NC_000962.3 2351446 2351465 TBseq_1.0_1315_LEFT 1 + TTGCCCACCAAGACGTGTT

NC_000962.3 2353483 2353504 TBseq_1.0_1315_RIGHT 1 - CCTCTGAGGATTGGATGACGC

NC_000962.3 2353311 2353331 TBseq_1.0_1316_LEFT 2 + GACGTTGACCCGATGCTACC

NC_000962.3 2355250 2355270 TBseq_1.0_1316_RIGHT 2 - TAGCCTTGCTGTCCACTCGT

NC_000962.3 2354061 2354081 TBseq_1.0_1317_LEFT 1 + GTGAGCTTTAACGGCCGGTC

NC_000962.3 2355967 2355990 TBseq_1.0_1317_RIGHT 1 - GTGCTGGAAATGATCGAAAGTGG

NC_000962.3 2356299 2356319 TBseq_1.0_1318_LEFT 2 + GTAGTTTTCGTGGCAGCCGT

NC_000962.3 2358310 2358330 TBseq_1.0_1318_RIGHT 2 - CTGAGCTGCGACGAATCTGG

NC_000962.3 2356672 2356694 TBseq_1.0_1319_LEFT 1 + TGCACGTATTTGAGGGTACTGG

NC_000962.3 2358712 2358731 TBseq_1.0_1319_RIGHT 1 - TGATAGTCAACGCCGCAGC

NC_000962.3 2358373 2358393 TBseq_1.0_1320_LEFT 2 + GAGTGGTCGAAGGTGATGGC

NC_000962.3 2360413 2360433 TBseq_1.0_1320_RIGHT 2 - AACGACGGCAACAGCAAAAC

NC_000962.3 2360081 2360100 TBseq_1.0_1321_LEFT 1 + ATGTCACGCCCCTTCTGGA

NC_000962.3 2362142 2362162 TBseq_1.0_1321_RIGHT 1 - ATAGGTGCTCACGACCAGGT

NC_000962.3 2362001 2362021 TBseq_1.0_1322_LEFT 2 + CACTGCTACTGTGCCCGATG

NC_000962.3 2363920 2363941 TBseq_1.0_1322_RIGHT 2 - GCGTAATAGCGGTCTAGGCAG

NC_000962.3 2363777 2363798 TBseq_1.0_1323_LEFT 1 + TCCTTCGACGAGGATCCGTTC

NC_000962.3 2365724 2365746 TBseq_1.0_1323_RIGHT 1 - GGTCTTTAAAATCGCGTTCGCC

NC_000962.3 2365297 2365318 TBseq_1.0_1324_LEFT 2 + CTACTCGGGGTAACACTTCGG

NC_000962.3 2367315 2367336 TBseq_1.0_1324_RIGHT 2 - CAAAGAACCCGAGCTACTGCA

NC_000962.3 2366907 2366925 TBseq_1.0_1325_LEFT 1 + ACGCACTTCCACGTTCCG

NC_000962.3 2368854 2368873 TBseq_1.0_1325_RIGHT 1 - CGACTCTGCGGGGCTATGA

NC_000962.3 2368626 2368647 TBseq_1.0_1326_LEFT 2 + ACAGATTTCATTGTCCCGGCC

NC_000962.3 2370597 2370617 TBseq_1.0_1326_RIGHT 2 - TCCAAAAGGGCGGACAGTGA

NC_000962.3 2370354 2370373 TBseq_1.0_1327_LEFT 1 + TCATGTTGCCCTGCGTCGA

NC_000962.3 2372376 2372396 TBseq_1.0_1327_RIGHT 1 - CGAGTACGGCATTTCCTCGC

NC_000962.3 2372133 2372152 TBseq_1.0_1328_LEFT 2 + CGTGGTCGACATACAGCCG

NC_000962.3 2374029 2374047 TBseq_1.0_1328_RIGHT 2 - ACCAATAGCGCGGACCGT

NC_000962.3 2372871 2372889 TBseq_1.0_1329_LEFT 1 + ATGGGTGATCCTGGCGCT

NC_000962.3 2374827 2374848 TBseq_1.0_1329_RIGHT 1 - GGTTGTCGACCGGATGTATGC

NC_000962.3 2374559 2374578 TBseq_1.0_1330_LEFT 2 + CTTCTTGCCCGAGATCCGC

NC_000962.3 2376577 2376599 TBseq_1.0_1330_RIGHT 2 - CCCAGGGTTACACGGATGTTTC

NC_000962.3 2376042 2376066 TBseq_1.0_1331_LEFT 1 + TGTTGACGGGCTTCTTTAAGAGTT

NC_000962.3 2378031 2378049 TBseq_1.0_1331_RIGHT 1 - GGCCCAGATCGTGCATGA

NC_000962.3 2377427 2377449 TBseq_1.0_1332_LEFT 2 + GCACTGACGACTAAGTGGACTG

NC_000962.3 2379383 2379400 TBseq_1.0_1332_RIGHT 2 - GTTATCGGCGCCGTGCT

NC_000962.3 2379091 2379111 TBseq_1.0_1333_LEFT 1 + CAGGCGAGACAGGCGATTTC

NC_000962.3 2381038 2381061 TBseq_1.0_1333_RIGHT 1 - CCTCCTTCGACCACAGGAATAAC

NC_000962.3 2380712 2380731 TBseq_1.0_1334_LEFT 2 + GCACCTGCGTCCAGTACAG

NC_000962.3 2382680 2382705 TBseq_1.0_1334_RIGHT 2 - GGCGAAAGAAGACTATTTCAAGCTC

NC_000962.3 2382545 2382567 TBseq_1.0_1335_LEFT 1 + TTCGGGATGCAGCTGTAATTCC

NC_000962.3 2384532 2384555 TBseq_1.0_1335_RIGHT 1 - TCCATCCTCATCGACACCTTGAC

NC_000962.3 2384388 2384408 TBseq_1.0_1336_LEFT 2 + GCTGCGGGATTGAGACCAAA

NC_000962.3 2386354 2386373 TBseq_1.0_1336_RIGHT 2 - GTCGTTCCAGCCCTCGAAC

NC_000962.3 2386152 2386173 TBseq_1.0_1337_LEFT 1 + CCACAAGAGTCTCAGCTGAGG

NC_000962.3 2388088 2388108 TBseq_1.0_1337_RIGHT 1 - CGTATCTCGCGCAGTTTGTC

NC_000962.3 2386449 2386470 TBseq_1.0_1338_LEFT 2 + ACGAGGCCTACTACGACTACC

NC_000962.3 2388497 2388518 TBseq_1.0_1338_RIGHT 2 - CAACTCCACCGGAATCGTACC

NC_000962.3 2388600 2388621 TBseq_1.0_1339_LEFT 1 + CCCGAGACTCCTGAATTGAGC

NC_000962.3 2390576 2390594 TBseq_1.0_1339_RIGHT 1 - CTGCGTTCGCCGAATTGC

NC_000962.3 2390387 2390405 TBseq_1.0_1340_LEFT 2 + GCTTGGCAACGGTGTTGC

NC_000962.3 2392322 2392343 TBseq_1.0_1340_RIGHT 2 - CATGTACGTCTGCGGGATCAC

NC_000962.3 2391984 2392004 TBseq_1.0_1341_LEFT 1 + ACTCTCCCATTTCCCGGTCT

NC_000962.3 2394022 2394044 TBseq_1.0_1341_RIGHT 1 - CGATTGACGACCAAATTCTGCA

NC_000962.3 2393851 2393869 TBseq_1.0_1342_LEFT 2 + TAAAACGCCGGCCAGGGA

NC_000962.3 2395837 2395857 TBseq_1.0_1342_RIGHT 2 - CTTCTCCAATGCTGCGGTGT

NC_000962.3 2395271 2395289 TBseq_1.0_1343_LEFT 1 + GCGTTCTTCGTCGACGGT

NC_000962.3 2397271 2397293 TBseq_1.0_1343_RIGHT 1 - CTCACGAATCCGAATCTGGCAA

NC_000962.3 2397083 2397104 TBseq_1.0_1344_LEFT 2 + TGTGGAAGGAGAAACTGCGTC

NC_000962.3 2399064 2399086 TBseq_1.0_1344_RIGHT 2 - CGTGATTGTTGAACCCCATCCG

NC_000962.3 2398920 2398938 TBseq_1.0_1345_LEFT 1 + TTCGACAAGGACGGCACC

NC_000962.3 2400822 2400843 TBseq_1.0_1345_RIGHT 1 - AAGGCAGTGCTACATGACACC

NC_000962.3 2400344 2400364 TBseq_1.0_1346_LEFT 2 + TGATCGACGTTGCGACTAGC

NC_000962.3 2402385 2402406 TBseq_1.0_1346_RIGHT 2 - CTTGTTTGTCGATGCCGTGAC

NC_000962.3 2402033 2402053 TBseq_1.0_1347_LEFT 1 + CTACTAATGGGCGTGGGGGT

NC_000962.3 2404083 2404103 TBseq_1.0_1347_RIGHT 1 - CCAGCTCCGGATCTAGACCC

NC_000962.3 2403947 2403969 TBseq_1.0_1348_LEFT 2 + CGGCCAGGTCTATAACGACTTT

NC_000962.3 2405900 2405921 TBseq_1.0_1348_RIGHT 2 - GTTCATCTTCTGGCTGCTGCT

NC_000962.3 2405680 2405700 TBseq_1.0_1349_LEFT 1 + AAACGCCAGTTGCATACCGA

NC_000962.3 2407732 2407750 TBseq_1.0_1349_RIGHT 1 - CAGCTCCGCGATTTGGGT

NC_000962.3 2407297 2407316 TBseq_1.0_1350_LEFT 2 + TGTTGCGTTGAATCCGGCC

NC_000962.3 2409264 2409283 TBseq_1.0_1350_RIGHT 2 - ACGCCAAGGACGAGATCGA

NC_000962.3 2409002 2409020 TBseq_1.0_1351_LEFT 1 + TACCGCGGCATCTCCCAT

NC_000962.3 2410915 2410933 TBseq_1.0_1351_RIGHT 1 - TTGGGCGTGCGCTGAATG

NC_000962.3 2410613 2410634 TBseq_1.0_1352_LEFT 2 + TCTGTGGGTCCTCGTTGTGTT

NC_000962.3 2412549 2412568 TBseq_1.0_1352_RIGHT 2 - GTGCTGCATGCCCATGGAC

NC_000962.3 2412342 2412361 TBseq_1.0_1353_LEFT 1 + TCAGCCGCTGTTCACCGTT

NC_000962.3 2414390 2414409 TBseq_1.0_1353_RIGHT 1 - ATCTGGGGAGCGCATCTGC

NC_000962.3 2414237 2414257 TBseq_1.0_1354_LEFT 2 + CATACCACAGCAGGCCCAAC

NC_000962.3 2416200 2416219 TBseq_1.0_1354_RIGHT 2 - ACGCGGTGCAGCAGATAAC

NC_000962.3 2415826 2415844 TBseq_1.0_1355_LEFT 1 + CGGCAATGTTGAGCACCG

NC_000962.3 2417737 2417759 TBseq_1.0_1355_RIGHT 1 - CGCTTAGATGTGTCTCGACTCG

NC_000962.3 2417391 2417414 TBseq_1.0_1356_LEFT 2 + CTTAGTGAACAACCGGATCAGCA

NC_000962.3 2419430 2419449 TBseq_1.0_1356_RIGHT 2 - TTCTCGCGCTGGTCGACTA

NC_000962.3 2417776 2417794 TBseq_1.0_1357_LEFT 1 + TGCGATCGTGCTCGGCTA

NC_000962.3 2419818 2419837 TBseq_1.0_1357_RIGHT 1 - TACTTCGAGGCCAAGGCGT

NC_000962.3 2420059 2420077 TBseq_1.0_1358_LEFT 2 + GATGCCGATGGTGCCGAT

NC_000962.3 2421975 2421995 TBseq_1.0_1358_RIGHT 2 - GATACCTGGAACGCGACGTG

NC_000962.3 2421171 2421190 TBseq_1.0_1359_LEFT 1 + GTTTCGTCCAGCAGCACCA

NC_000962.3 2423137 2423155 TBseq_1.0_1359_RIGHT 1 - TAATGAAAGCCGCCGCCG

NC_000962.3 2422960 2422977 TBseq_1.0_1360_LEFT 2 + GCAGCGGCGATACCAGT

NC_000962.3 2424846 2424864 TBseq_1.0_1360_RIGHT 2 - TGTGCTGAGCTGGTCGGA

NC_000962.3 2424847 2424866 TBseq_1.0_1361_LEFT 1 + CCGACCAGCTCAGCACAAC

NC_000962.3 2426786 2426809 TBseq_1.0_1361_RIGHT 1 - GGTCATCTTGGTGTTGATGTTGG

NC_000962.3 2426356 2426377 TBseq_1.0_1362_LEFT 2 + TGACGCAGATCCTGTCTTTCC

NC_000962.3 2428389 2428408 TBseq_1.0_1362_RIGHT 2 - GAACTTCCCGGCCATGAGC

NC_000962.3 2428172 2428191 TBseq_1.0_1363_LEFT 1 + AGGACTGTCGGCTCTCCTG

NC_000962.3 2430237 2430256 TBseq_1.0_1363_RIGHT 1 - TCAACCATCGCCGCCTCTA

NC_000962.3 2429996 2430018 TBseq_1.0_1364_LEFT 2 + CTACGACCTCAGAAAACCGCAT

NC_000962.3 2432027 2432047 TBseq_1.0_1364_RIGHT 2 - ATCTGAGTTAGTTGCCCGCG

NC_000962.3 2431281 2431301 TBseq_1.0_1365_LEFT 1 + CGCAGCCAACACCAAGTAGA

NC_000962.3 2433321 2433343 TBseq_1.0_1365_RIGHT 1 - GGATACCGATGTCAGATCCGCT

NC_000962.3 2432491 2432510 TBseq_1.0_1366_LEFT 2 + AGTGGCGTACGGCTATCCC

NC_000962.3 2434451 2434472 TBseq_1.0_1366_RIGHT 2 - CAATCGAAGATGCTCGCAAGG

NC_000962.3 2434258 2434278 TBseq_1.0_1367_LEFT 1 + TCCATGAACGCGGTTACCTG

NC_000962.3 2436149 2436171 TBseq_1.0_1367_RIGHT 1 - GTCCACAACAGAATCGACGACA

NC_000962.3 2435905 2435925 TBseq_1.0_1368_LEFT 2 + CCGATGACTACTCCGAGCCA

NC_000962.3 2437886 2437904 TBseq_1.0_1368_RIGHT 2 - AACGGAAGCCTAACGCGC

NC_000962.3 2437641 2437660 TBseq_1.0_1369_LEFT 1 + GGCAGGCTCTTGACCACCT

NC_000962.3 2439626 2439644 TBseq_1.0_1369_RIGHT 1 - CACGAATCGGCCGGCAAA

NC_000962.3 2439469 2439488 TBseq_1.0_1370_LEFT 2 + TCTTGTTGGCAGCGGGACT

NC_000962.3 2441446 2441466 TBseq_1.0_1370_RIGHT 2 - CGACTGCGCTGAGACATTCA

NC_000962.3 2441022 2441041 TBseq_1.0_1371_LEFT 1 + ACCCCACAGGCACTCATGA

NC_000962.3 2443040 2443058 TBseq_1.0_1371_RIGHT 1 - TGGCAGCCGCGAAATGTC

NC_000962.3 2442833 2442854 TBseq_1.0_1372_LEFT 2 + TCCGTCTTCCTCCCAGTAGAC

NC_000962.3 2444874 2444894 TBseq_1.0_1372_RIGHT 2 - GTGATTCCGGTGGCGATGAT

NC_000962.3 2443789 2443811 TBseq_1.0_1373_LEFT 1 + CGCTTTCGGTCAGATCAATGTG

NC_000962.3 2445741 2445762 TBseq_1.0_1373_RIGHT 1 - CAGGCAATCCTCGATGGGATC

NC_000962.3 2445571 2445591 TBseq_1.0_1374_LEFT 2 + ATCACCTCCAGCAGAGCCAG

NC_000962.3 2447477 2447497 TBseq_1.0_1374_RIGHT 2 - GCGGACAAGACGACACAGAC

NC_000962.3 2447181 2447201 TBseq_1.0_1375_LEFT 1 + TGACCTCAGTGCCAGAACCC

NC_000962.3 2449119 2449136 TBseq_1.0_1375_RIGHT 1 - TTGGCGCGCAGTAGCAG

NC_000962.3 2448842 2448862 TBseq_1.0_1376_LEFT 2 + GGTTTTCCTGCCGCTAGCTC

NC_000962.3 2450765 2450784 TBseq_1.0_1376_RIGHT 2 - GTGGGCTGGTCGATGCTTC

NC_000962.3 2449515 2449535 TBseq_1.0_1377_LEFT 1 + GGGTTCTTGACGATCACCGG

NC_000962.3 2451476 2451496 TBseq_1.0_1377_RIGHT 1 - GTCTGTGGCGCTGTCGTTAC

NC_000962.3 2451240 2451260 TBseq_1.0_1378_LEFT 2 + CGGAAGCTAGCCCATTAGCC

NC_000962.3 2453265 2453290 TBseq_1.0_1378_RIGHT 2 - CGTTTAAGGAAGCAAATTTTGAGGC

NC_000962.3 2452879 2452898 TBseq_1.0_1379_LEFT 1 + CAACAGTTGCGGGGACTCC

NC_000962.3 2454784 2454805 TBseq_1.0_1379_RIGHT 1 - CTTCGACCTGCGGTTATACGG

NC_000962.3 2454127 2454147 TBseq_1.0_1380_LEFT 2 + ACTACGGCGATGGTGGGTAA

NC_000962.3 2456186 2456205 TBseq_1.0_1380_RIGHT 2 - CGCGGTTCCATCCCTCCTA

NC_000962.3 2455615 2455635 TBseq_1.0_1381_LEFT 1 + AGCAGAGGTTGGCAGTCAGA

NC_000962.3 2457576 2457596 TBseq_1.0_1381_RIGHT 1 - CCACTCTTACTGCCACCGGA

NC_000962.3 2457383 2457403 TBseq_1.0_1382_LEFT 2 + CTGGTACGCACTGGGATGAG

NC_000962.3 2459351 2459372 TBseq_1.0_1382_RIGHT 2 - ACTGGGTTTGATCCGGATGAG

NC_000962.3 2458705 2458723 TBseq_1.0_1383_LEFT 1 + GTTCGGACTGGCGCTGTT

NC_000962.3 2460722 2460742 TBseq_1.0_1383_RIGHT 1 - CAGGAATGGGTAGGCGGGTA

NC_000962.3 2460309 2460329 TBseq_1.0_1384_LEFT 2 + GCTCTACGCCCTGCACATTT

NC_000962.3 2462268 2462287 TBseq_1.0_1384_RIGHT 2 - CCCCGATCTCGCAATCCGA

NC_000962.3 2462064 2462083 TBseq_1.0_1385_LEFT 1 + ACATGCCCCAACCAGGATG

NC_000962.3 2464084 2464108 TBseq_1.0_1385_RIGHT 1 - GGACCTACCTGAATTTCGACAAGG

NC_000962.3 2463885 2463907 TBseq_1.0_1386_LEFT 2 + CCGGTCTTGGTGATTTCTTCGA

NC_000962.3 2465926 2465943 TBseq_1.0_1386_RIGHT 2 - AAGCCCTCGCGCTCGAA

NC_000962.3 2465783 2465802 TBseq_1.0_1387_LEFT 1 + CGGCAGTGCTTGAGGACTC

NC_000962.3 2467696 2467717 TBseq_1.0_1387_RIGHT 1 - GTACCACCGATGTCGACATGG

NC_000962.3 2467424 2467447 TBseq_1.0_1388_LEFT 2 + CAGATCCCATTCGTAGTCGTTGG

NC_000962.3 2469404 2469423 TBseq_1.0_1388_RIGHT 2 - GCGACTCGGGAATAGCGGT

NC_000962.3 2469258 2469275 TBseq_1.0_1389_LEFT 1 + TTGGCGCTCGGCTCGTT

NC_000962.3 2471193 2471212 TBseq_1.0_1389_RIGHT 1 - CTTCGTGTCAACCAGCCGG

NC_000962.3 2470540 2470559 TBseq_1.0_1390_LEFT 2 + AGAGTAGGTCTGCGCACCC

NC_000962.3 2472521 2472542 TBseq_1.0_1390_RIGHT 2 - GTGGGTATCACCGTTGCGAAT

NC_000962.3 2471269 2471288 TBseq_1.0_1391_LEFT 1 + TCCCAGATACGCCGGTTGC

NC_000962.3 2473303 2473323 TBseq_1.0_1391_RIGHT 1 - TAATCTGGGGACACGGAGGG

NC_000962.3 2473510 2473530 TBseq_1.0_1392_LEFT 2 + GTGGTACTGCCGGTCATCTG

NC_000962.3 2475465 2475488 TBseq_1.0_1392_RIGHT 2 - CACCCAATACCGTTACCTGTTGA

NC_000962.3 2475227 2475249 TBseq_1.0_1393_LEFT 1 + AAGAAGATGTTCATGCCACCCA

NC_000962.3 2477293 2477313 TBseq_1.0_1393_RIGHT 1 - CGGCTTGCACCATCTCTTCG

NC_000962.3 2477113 2477133 TBseq_1.0_1394_LEFT 2 + GGTCCGTGTATCAGCTCTGG

NC_000962.3 2479131 2479151 TBseq_1.0_1394_RIGHT 2 - TGGCCTTTTGGGGGTTCTTG

NC_000962.3 2478929 2478948 TBseq_1.0_1395_LEFT 1 + TCAACACTCCCCCAAGCCA

NC_000962.3 2480970 2480992 TBseq_1.0_1395_RIGHT 1 - CCATTCCGATAGTTGATGTGCC

NC_000962.3 2480560 2480579 TBseq_1.0_1396_LEFT 2 + CGAGCACATAGGGGTACGC

NC_000962.3 2482480 2482501 TBseq_1.0_1396_RIGHT 2 - GCTGATACTGACCAAGACCCC

NC_000962.3 2482022 2482041 TBseq_1.0_1397_LEFT 1 + CCCGCTGGCTCAAACAGGA

NC_000962.3 2483974 2483993 TBseq_1.0_1397_RIGHT 1 - CGGTCCTTGGTGTTTCCGT

NC_000962.3 2483608 2483630 TBseq_1.0_1398_LEFT 2 + GCCGATTTAGGACTGTGATGGC

NC_000962.3 2485608 2485625 TBseq_1.0_1398_RIGHT 2 - GCATAGCGCAGGCCCAT

NC_000962.3 2485256 2485276 TBseq_1.0_1399_LEFT 1 + CCGTACCATCGCCGTTATGA

NC_000962.3 2487162 2487181 TBseq_1.0_1399_RIGHT 1 - GATTCACACCCGGGCAGTT

NC_000962.3 2486753 2486774 TBseq_1.0_1400_LEFT 2 + GACGTGCCGATCTACGACATT

NC_000962.3 2488735 2488755 TBseq_1.0_1400_RIGHT 2 - AGAACGCCAGATACGGGTTG

NC_000962.3 2488057 2488077 TBseq_1.0_1401_LEFT 1 + CAACGGCTCCTTCTACGAGG

NC_000962.3 2490007 2490025 TBseq_1.0_1401_RIGHT 1 - AATTTGCGACCCGAGGGC

NC_000962.3 2489695 2489715 TBseq_1.0_1402_LEFT 2 + CAACTGCACGGTCCATTCGA

NC_000962.3 2491672 2491691 TBseq_1.0_1402_RIGHT 2 - TGCGCGCGAACTGAACTAC

NC_000962.3 2491413 2491431 TBseq_1.0_1403_LEFT 1 + AACGCTCGCCAAGTTCCG

NC_000962.3 2493318 2493337 TBseq_1.0_1403_RIGHT 1 - TTCCTGCTCAAGCCCGGAC

NC_000962.3 2493079 2493102 TBseq_1.0_1404_LEFT 2 + GACTTCTTTGATGACGTAGCGGA

NC_000962.3 2494972 2494990 TBseq_1.0_1404_RIGHT 2 - ACTTCGACCTGGTGGGCT

NC_000962.3 2494709 2494732 TBseq_1.0_1405_LEFT 1 + TCCGAGGTAGTTGATCTGATCGT

NC_000962.3 2496712 2496732 TBseq_1.0_1405_RIGHT 1 - GGAGACAAGATCGGTTCGCT

NC_000962.3 2496428 2496451 TBseq_1.0_1406_LEFT 2 + CCAGAAAGTTCTTGCCCATCTTG

NC_000962.3 2498321 2498341 TBseq_1.0_1406_RIGHT 2 - CATCTCCATCACGACGGCAA

NC_000962.3 2497870 2497890 TBseq_1.0_1407_LEFT 1 + TATTCGACGGCCCGGATCTT

NC_000962.3 2499945 2499962 TBseq_1.0_1407_RIGHT 1 - AGAAGTGGCATGGGCGC

NC_000962.3 2499398 2499418 TBseq_1.0_1408_LEFT 2 + CTACTGGACCGGCTAGCCAA

NC_000962.3 2501462 2501482 TBseq_1.0_1408_RIGHT 2 - AAGTTGCATTGCGGTACCCC

NC_000962.3 2501243 2501263 TBseq_1.0_1409_LEFT 1 + GCCTTGATGACTGGTTGGCG

NC_000962.3 2503183 2503202 TBseq_1.0_1409_RIGHT 1 - ACGGACGCCAAGCAATTGG

NC_000962.3 2502972 2502991 TBseq_1.0_1410_LEFT 2 + GAATGTTGGTGCCGGGCTT

NC_000962.3 2504927 2504948 TBseq_1.0_1410_RIGHT 2 - TGGGAACATTGCAACTGACGG

NC_000962.3 2504619 2504636 TBseq_1.0_1411_LEFT 1 + ACGCTTTGCCCACTCGG

NC_000962.3 2506650 2506669 TBseq_1.0_1411_RIGHT 1 - CGCAGCGTCTCATGCATGG

NC_000962.3 2506188 2506206 TBseq_1.0_1412_LEFT 2 + GCAGCGGCACAAGCTTTG

NC_000962.3 2508189 2508209 TBseq_1.0_1412_RIGHT 2 - CAGATAGGACCCAGCCAGCT

NC_000962.3 2507754 2507775 TBseq_1.0_1413_LEFT 1 + AAACGTCACGAGAGAACCAGC

NC_000962.3 2509746 2509765 TBseq_1.0_1413_RIGHT 1 - CCACCCACAGCTGCAACTT

NC_000962.3 2509394 2509419 TBseq_1.0_1414_LEFT 2 + TGGTCTACGCTCAGCTATTTATGAC

NC_000962.3 2511388 2511407 TBseq_1.0_1414_RIGHT 2 - TGATGGCGACGGCGATTTG

NC_000962.3 2510697 2510718 TBseq_1.0_1415_LEFT 1 + GCCAAGCACCCAAAACCTTAG

NC_000962.3 2512686 2512706 TBseq_1.0_1415_RIGHT 1 - GGTAGACGTCAATGCCGGGA

NC_000962.3 2511933 2511952 TBseq_1.0_1416_LEFT 2 + TTGCGGCCCTGATTGAACG

NC_000962.3 2513895 2513915 TBseq_1.0_1416_RIGHT 2 - CCGGTCGAGCATGTAACGAA

NC_000962.3 2513288 2513311 TBseq_1.0_1417_LEFT 1 + TTGGACAACTTGACCTTCGTGAT

NC_000962.3 2515361 2515379 TBseq_1.0_1417_RIGHT 1 - GCGAATCGGGCACAGTGT

NC_000962.3 2514593 2514617 TBseq_1.0_1418_LEFT 2 + AACCCGGAGAACATCTTCTTCTAC

NC_000962.3 2516645 2516666 TBseq_1.0_1418_RIGHT 2 - GCGACCTGTTGTCTGGATCAC

NC_000962.3 2516355 2516377 TBseq_1.0_1419_LEFT 1 + AGACGCATATCTGGATTGTGGC

NC_000962.3 2518310 2518328 TBseq_1.0_1419_RIGHT 1 - TGTGGCTGTCGACCGGAT

NC_000962.3 2518111 2518132 TBseq_1.0_1420_LEFT 2 + CAAGTGAGTCAGCCTTCCACC

NC_000962.3 2520085 2520104 TBseq_1.0_1420_RIGHT 2 - TCACGATGCGCATCTGAGC

NC_000962.3 2519649 2519670 TBseq_1.0_1421_LEFT 1 + TGAGGAATTCGACCACCAGCT

NC_000962.3 2521620 2521640 TBseq_1.0_1421_RIGHT 1 - CGCTTTCGGAGTTCAGGCAG

NC_000962.3 2520661 2520678 TBseq_1.0_1422_LEFT 2 + TCGTTCGGATTCGGCGG

NC_000962.3 2522567 2522587 TBseq_1.0_1422_RIGHT 2 - CCGCATCCTTACTCCGGGAT

NC_000962.3 2522421 2522440 TBseq_1.0_1423_LEFT 1 + GGTCTACGCGGCACAAGAG

NC_000962.3 2524399 2524416 TBseq_1.0_1423_RIGHT 1 - TTGGTGCGTGCCGGTTT

NC_000962.3 2523661 2523681 TBseq_1.0_1424_LEFT 2 + CCCGACGGCGATTCAAAAAC

NC_000962.3 2525590 2525609 TBseq_1.0_1424_RIGHT 2 - CGGCGGTGCGGAAATACTC

NC_000962.3 2525405 2525422 TBseq_1.0_1425_LEFT 1 + AATGGGACGCGTGGGGA

NC_000962.3 2527363 2527383 TBseq_1.0_1425_RIGHT 1 - TCAATGGTTTCCGTCCAGCC

NC_000962.3 2526393 2526412 TBseq_1.0_1426_LEFT 2 + AAACCCAAATCACCGGCGG

NC_000962.3 2528348 2528367 TBseq_1.0_1426_RIGHT 2 - GATCGATTTGGCTGGGGCA

NC_000962.3 2527969 2527990 TBseq_1.0_1427_LEFT 1 + TACGGGAGGAACAGATGTCCG

NC_000962.3 2529949 2529971 TBseq_1.0_1427_RIGHT 1 - ACAAGGCACAATAGAAACGCAC

NC_000962.3 2529793 2529812 TBseq_1.0_1428_LEFT 2 + CGAGGCGTGGATGTACACC

NC_000962.3 2531734 2531754 TBseq_1.0_1428_RIGHT 2 - CATGGAGATCGCTGAGGCTG

NC_000962.3 2531478 2531498 TBseq_1.0_1429_LEFT 1 + TTGTGGAAGCGCGGGTATTC

NC_000962.3 2533398 2533418 TBseq_1.0_1429_RIGHT 1 - CCGACCAGCCAGATGTTGTT

NC_000962.3 2533184 2533203 TBseq_1.0_1430_LEFT 2 + ACTTCCCCACGCTGATCGA

NC_000962.3 2535097 2535117 TBseq_1.0_1430_RIGHT 2 - CTGGGTGCATCGCAATGGAG

NC_000962.3 2534808 2534825 TBseq_1.0_1431_LEFT 1 + AACGGCCGCGGTGAACT

NC_000962.3 2536764 2536785 TBseq_1.0_1431_RIGHT 1 - GACCACCGATGTCATCACTCC

NC_000962.3 2536578 2536597 TBseq_1.0_1432_LEFT 2 + CCCGAAATCGCGCTGAACT

NC_000962.3 2538610 2538630 TBseq_1.0_1432_RIGHT 2 - CCGACGGCCGAACTCATTTC

NC_000962.3 2538404 2538428 TBseq_1.0_1433_LEFT 1 + CGTCATACCTATAGCCAATGTGGG

NC_000962.3 2540350 2540375 TBseq_1.0_1433_RIGHT 1 - TACGAACTGAAAATGTCCGGATGAC

NC_000962.3 2539868 2539889 TBseq_1.0_1434_LEFT 2 + TGAACTGATGGCCCTGGTGAT

NC_000962.3 2541939 2541959 TBseq_1.0_1434_RIGHT 2 - CCGAAAGTTGGACGAAGGCC

NC_000962.3 2541560 2541581 TBseq_1.0_1435_LEFT 1 + CAGGATGATGTTGGCGAGACG

NC_000962.3 2543458 2543477 TBseq_1.0_1435_RIGHT 1 - TTTCGCTTGTGCAGGGCTT

NC_000962.3 2543094 2543111 TBseq_1.0_1436_LEFT 2 + TGAGCCTGCCGGGATGA

NC_000962.3 2545113 2545134 TBseq_1.0_1436_RIGHT 2 - TCCTGATGCGAGTCGATGAGA

NC_000962.3 2544878 2544902 TBseq_1.0_1437_LEFT 1 + AGACCTGGATTAACCTTCAAGTCG

NC_000962.3 2546858 2546881 TBseq_1.0_1437_RIGHT 1 - GTTTTCCTTGGACCATAGGGACG

NC_000962.3 2546656 2546676 TBseq_1.0_1438_LEFT 2 + TGCTCAGCCTGCAAGTCAAC

NC_000962.3 2548569 2548590 TBseq_1.0_1438_RIGHT 2 - GCGAAGGAGAGATTGATCCGC

NC_000962.3 2548230 2548253 TBseq_1.0_1439_LEFT 1 + CCGTAGCTTGAGTATCGCTTTCA

NC_000962.3 2550207 2550226 TBseq_1.0_1439_RIGHT 1 - CACCCACTTACGCACCGTC

NC_000962.3 2549580 2549604 TBseq_1.0_1440_LEFT 2 + GGAGTCTGCTTGAGGTCTAGAAAC

NC_000962.3 2551539 2551559 TBseq_1.0_1440_RIGHT 2 - CGGAGGCCTCACTTTCCAAC

NC_000962.3 2551103 2551126 TBseq_1.0_1441_LEFT 1 + CTCCTATGACAATGCACTAGCCG

NC_000962.3 2553100 2553119 TBseq_1.0_1441_RIGHT 1 - ACGGGTAAACGCCGGATGA

NC_000962.3 2552291 2552315 TBseq_1.0_1442_LEFT 2 + CCTGACATCCTGGAGTACATTGAC

NC_000962.3 2554225 2554250 TBseq_1.0_1442_RIGHT 2 - AGTAGTTATTCATCGTGGTAAGGCC

NC_000962.3 2554075 2554095 TBseq_1.0_1443_LEFT 1 + TACTACCGCGAGCTACACGA

NC_000962.3 2555970 2555989 TBseq_1.0_1443_RIGHT 1 - TTTTCGAGGCACCGCAGTC

NC_000962.3 2555751 2555772 TBseq_1.0_1444_LEFT 2 + CGCCTTGACACAGCTTGTTGA

NC_000962.3 2557805 2557824 TBseq_1.0_1444_RIGHT 2 - GGTGCAACTCCCATAGCGG

NC_000962.3 2557623 2557642 TBseq_1.0_1445_LEFT 1 + CGTTCCTGCCCTTCCCGTT

NC_000962.3 2559604 2559624 TBseq_1.0_1445_RIGHT 1 - GCACGGCACAATGTCATCGA

NC_000962.3 2559389 2559409 TBseq_1.0_1446_LEFT 2 + GCCATAGGACCAGTCTCGCT

NC_000962.3 2561292 2561314 TBseq_1.0_1446_RIGHT 2 - GGTCCAAGAGTTGCACTTCCTC

NC_000962.3 2561063 2561082 TBseq_1.0_1447_LEFT 1 + CGCCCCCGATCTCGTCAAA

NC_000962.3 2563089 2563110 TBseq_1.0_1447_RIGHT 1 - GGACAGGTTACATGTACCCCG

NC_000962.3 2562851 2562872 TBseq_1.0_1448_LEFT 2 + GGGTTAACGCACACGACGATT

NC_000962.3 2564776 2564799 TBseq_1.0_1448_RIGHT 2 - CAAGAAGGTGATTGTGGCGATGA

NC_000962.3 2564383 2564404 TBseq_1.0_1449_LEFT 1 + CGTTCGGGATGCAAGGAAAAG

NC_000962.3 2566338 2566359 TBseq_1.0_1449_RIGHT 1 - CTTCGGTCATCTCGTCGCTAG

NC_000962.3 2566039 2566059 TBseq_1.0_1450_LEFT 2 + GTGGAATCTCGGCGGACTCA

NC_000962.3 2568086 2568106 TBseq_1.0_1450_RIGHT 2 - GGGGAAAGGTGCATCGTAGC

NC_000962.3 2567836 2567856 TBseq_1.0_1451_LEFT 1 + CTCGACCTGCACGACGTTAC

NC_000962.3 2569792 2569809 TBseq_1.0_1451_RIGHT 1 - ATGGCGCGCAACGTAGC

NC_000962.3 2569608 2569628 TBseq_1.0_1452_LEFT 2 + CCCTGATGCGCTCGAAGATC

NC_000962.3 2571568 2571588 TBseq_1.0_1452_RIGHT 2 - AAGGTCCAACTGCTTACCCG

NC_000962.3 2571395 2571418 TBseq_1.0_1453_LEFT 1 + CCAGGTTACGGATCTTCCATTCC

NC_000962.3 2573321 2573341 TBseq_1.0_1453_RIGHT 1 - GGCCATGCTCTGAATGTGGG

NC_000962.3 2573073 2573093 TBseq_1.0_1454_LEFT 2 + CGCGGTGCTTCTGCTTACTC

NC_000962.3 2575041 2575061 TBseq_1.0_1454_RIGHT 2 - CGATGACTTCCTGCACGGTC

NC_000962.3 2574904 2574924 TBseq_1.0_1455_LEFT 1 + AATACGACAGCAGCTCGACG

NC_000962.3 2576864 2576884 TBseq_1.0_1455_RIGHT 1 - CCCAGGTTGGACAGACAGAC

NC_000962.3 2576723 2576744 TBseq_1.0_1456_LEFT 2 + GGGATCACCGGACCATTGAAG

NC_000962.3 2578679 2578702 TBseq_1.0_1456_RIGHT 2 - GGCTAGATGTCGCTAAAGAGGTG

NC_000962.3 2578496 2578515 TBseq_1.0_1457_LEFT 1 + CCACCCAAGCGCATTCCAT

NC_000962.3 2580467 2580489 TBseq_1.0_1457_RIGHT 1 - AGCTTATCGACCTCGGCGTATA

NC_000962.3 2579645 2579668 TBseq_1.0_1458_LEFT 2 + CTGATTCCGTCACAATATCCCCA

NC_000962.3 2581656 2581676 TBseq_1.0_1458_RIGHT 2 - CTGTCGTCCTACCACTGCGA

NC_000962.3 2581330 2581349 TBseq_1.0_1459_LEFT 1 + AACGACAGGCATCTCCGGA

NC_000962.3 2583314 2583333 TBseq_1.0_1459_RIGHT 1 - CTCATGGGCCGACAGGAAG

NC_000962.3 2583067 2583089 TBseq_1.0_1460_LEFT 2 + CACCATAAATCGACACCCTCCG

NC_000962.3 2585042 2585060 TBseq_1.0_1460_RIGHT 2 - TTCGGTTGACGGCGGTCT

NC_000962.3 2584695 2584715 TBseq_1.0_1461_LEFT 1 + GGCGGGATCGACCTAACTGA

NC_000962.3 2586609 2586629 TBseq_1.0_1461_RIGHT 1 - GGCGCTACCAGACCATCATG

NC_000962.3 2586062 2586085 TBseq_1.0_1462_LEFT 2 + GGCTTTCGTTAAACCGGAAGTTG

NC_000962.3 2588118 2588139 TBseq_1.0_1462_RIGHT 2 - CGACGAGATCTGGAACTGGAC

NC_000962.3 2587911 2587930 TBseq_1.0_1463_LEFT 1 + CCGAGTTTGTCTGGGGTGG

NC_000962.3 2589939 2589960 TBseq_1.0_1463_RIGHT 1 - CGAAAATGTCAGCTGGCCCAG

NC_000962.3 2589755 2589775 TBseq_1.0_1464_LEFT 2 + GGTAATCACGCTGTCCCCCT

NC_000962.3 2591783 2591802 TBseq_1.0_1464_RIGHT 2 - GCCTCAGGGTTGTCGTGAC

NC_000962.3 2591621 2591643 TBseq_1.0_1465_LEFT 1 + CCGGTCTACTTCGACTACTGGT

NC_000962.3 2593569 2593588 TBseq_1.0_1465_RIGHT 1 - CGAGGGTCAACGCGATCAT

NC_000962.3 2592860 2592881 TBseq_1.0_1466_LEFT 2 + CGAAAGAACAGGCGTCACAGG

NC_000962.3 2594919 2594943 TBseq_1.0_1466_RIGHT 2 - CAAACAACAACTTTCATGGCCACA

NC_000962.3 2594668 2594688 TBseq_1.0_1467_LEFT 1 + CCAGCCCCGATTGGATTTCG

NC_000962.3 2596594 2596615 TBseq_1.0_1467_RIGHT 1 - GACCACCTCGGGGATATTCAC

NC_000962.3 2596311 2596331 TBseq_1.0_1468_LEFT 2 + TGCTTAGAACGGAGGAGGGC

NC_000962.3 2598322 2598341 TBseq_1.0_1468_RIGHT 2 - GAGCCTGCCTACTTGACGG

NC_000962.3 2597845 2597867 TBseq_1.0_1469_LEFT 1 + AGTTGTGGTCATGACATTCCCC

NC_000962.3 2599778 2599799 TBseq_1.0_1469_RIGHT 1 - CGATCATCTCCGTCGTCGTTC

NC_000962.3 2598709 2598726 TBseq_1.0_1470_LEFT 2 + AGCAAAGCGTCCCTGCC

NC_000962.3 2600703 2600724 TBseq_1.0_1470_RIGHT 2 - TCCTCTCGACTCACTACGGTT

NC_000962.3 2600509 2600528 TBseq_1.0_1471_LEFT 1 + CGGTGTGGGTCGGATCGAT

NC_000962.3 2602443 2602468 TBseq_1.0_1471_RIGHT 1 - CGCTTGACCCTTATAGTCTTTGTGG

NC_000962.3 2602197 2602220 TBseq_1.0_1472_LEFT 2 + TGACTACTCCTGAGATGATGCGT

NC_000962.3 2604183 2604202 TBseq_1.0_1472_RIGHT 2 - CGTCCCGATTCTGGCACTG

NC_000962.3 2603991 2604013 TBseq_1.0_1473_LEFT 1 + GGTGATTTCGACCCATTTGGAG

NC_000962.3 2605966 2605987 TBseq_1.0_1473_RIGHT 1 - CGACTATTGTCTGATCGCGCA

NC_000962.3 2605449 2605469 TBseq_1.0_1474_LEFT 2 + CGCGGACTGTTTCTGAGCAT

NC_000962.3 2607410 2607429 TBseq_1.0_1474_RIGHT 2 - CCGGCCTGATGATCCTTCC

NC_000962.3 2606451 2606471 TBseq_1.0_1475_LEFT 1 + GTCATCGGTCAGGCCAACAA

NC_000962.3 2608480 2608500 TBseq_1.0_1475_RIGHT 1 - CGGGATCGGTTCACCTAGCT

NC_000962.3 2608211 2608230 TBseq_1.0_1476_LEFT 2 + AAACTGCGCTGGATGTCGG

NC_000962.3 2610122 2610142 TBseq_1.0_1476_RIGHT 2 - CTTGATCGGACCGAGGACCT

NC_000962.3 2609648 2609671 TBseq_1.0_1477_LEFT 1 + GGAAGCTAATCGTCGTAGTGCTC

NC_000962.3 2611604 2611627 TBseq_1.0_1477_RIGHT 1 - GGAACCAGCAAGAGATCATTCGA

NC_000962.3 2611387 2611408 TBseq_1.0_1478_LEFT 2 + GGAGATTGCGGCATTATCGGT

NC_000962.3 2613308 2613330 TBseq_1.0_1478_RIGHT 2 - CCGAGGTGTTGAAGATTCTGCT

NC_000962.3 2613067 2613093 TBseq_1.0_1479_LEFT 1 + GGCAGTATCTGAGTACACCATTCTAG

NC_000962.3 2615101 2615121 TBseq_1.0_1479_RIGHT 1 - GTACGCCGCCTTACCATCTG

NC_000962.3 2614904 2614923 TBseq_1.0_1480_LEFT 2 + ACGTGCCGGAAAGGTGTTC

NC_000962.3 2616941 2616961 TBseq_1.0_1480_RIGHT 2 - CAGCCGTGCCACCGATATAG

NC_000962.3 2615284 2615305 TBseq_1.0_1481_LEFT 1 + GCATGAAGCTGATCACCGGAT

NC_000962.3 2617224 2617244 TBseq_1.0_1481_RIGHT 1 - CGGGACACCAAAAGGAGGTT

NC_000962.3 2617055 2617075 TBseq_1.0_1482_LEFT 2 + TACCCAGAGCCTGATTGCGT

NC_000962.3 2619021 2619041 TBseq_1.0_1482_RIGHT 2 - TCAGTGTGAGGCGCAGTAGG

NC_000962.3 2617569 2617590 TBseq_1.0_1483_LEFT 1 + CACCGGGGTTGATATGCACTT

NC_000962.3 2619552 2619574 TBseq_1.0_1483_RIGHT 1 - CTCCCATCGGTAAGGAATCTCC

NC_000962.3 2619340 2619363 TBseq_1.0_1484_LEFT 2 + CGAACCTTAGCTCATCAGAACCC

NC_000962.3 2621376 2621396 TBseq_1.0_1484_RIGHT 2 - CCCTGCGACCTACGACTGAA

NC_000962.3 2620749 2620769 TBseq_1.0_1485_LEFT 1 + TCGACCTGAATCGCCTCCAC

NC_000962.3 2622745 2622764 TBseq_1.0_1485_RIGHT 1 - AGGTGCCCGATCTGGTACG

NC_000962.3 2622500 2622518 TBseq_1.0_1486_LEFT 2 + TGCGTTCCAACCGACCCT

NC_000962.3 2624420 2624441 TBseq_1.0_1486_RIGHT 2 - TGCCATTACGTTGGTAGGGTC

NC_000962.3 2624044 2624062 TBseq_1.0_1487_LEFT 1 + CGGTCAGAGTGCGCTCAA

NC_000962.3 2626067 2626087 TBseq_1.0_1487_RIGHT 1 - AGGCCATCGTTCGTGATGTG

NC_000962.3 2625912 2625933 TBseq_1.0_1488_LEFT 2 + CTGTCGGTTTGTGCCATGTTG

NC_000962.3 2627833 2627853 TBseq_1.0_1488_RIGHT 2 - CCGCGACGTCATCAACAACA

NC_000962.3 2627657 2627677 TBseq_1.0_1489_LEFT 1 + GAAGAAGCCGCCATGTTCGT

NC_000962.3 2629645 2629665 TBseq_1.0_1489_RIGHT 1 - CACTACAGCTGGCGCATCAT

NC_000962.3 2629437 2629460 TBseq_1.0_1490_LEFT 2 + GGGTAATCGGTTGTTTCTGACGT

NC_000962.3 2631409 2631431 TBseq_1.0_1490_RIGHT 2 - GCAATTCAGTTGGCGCATCATG

NC_000962.3 2630656 2630676 TBseq_1.0_1491_LEFT 1 + CACGTTAGGGATGCACTGCG

NC_000962.3 2632594 2632613 TBseq_1.0_1491_RIGHT 1 - CAGCCAGGGCAACACTTGT

NC_000962.3 2632188 2632208 TBseq_1.0_1492_LEFT 2 + TTTTCGCGTTCCAGCAACCG

NC_000962.3 2634181 2634202 TBseq_1.0_1492_RIGHT 2 - GCGGATTTTCGGTGTGGATTG

NC_000962.3 2634144 2634164 TBseq_1.0_1493_LEFT 1 + CTCCAGACACAAACCAGCCC

NC_000962.3 2636156 2636177 TBseq_1.0_1493_RIGHT 1 - CCGTAAACACCGTAGTTGGCG

NC_000962.3 2634493 2634515 TBseq_1.0_1494_LEFT 2 + CCAACTACATCGCACTACCGAA

NC_000962.3 2636554 2636579 TBseq_1.0_1494_RIGHT 2 - GTATGGTGGATAACGTCTTTCAGGT

NC_000962.3 2637520 2637542 TBseq_1.0_1495_LEFT 1 + GAACCAACTCAGGTCTTACGCT

NC_000962.3 2639539 2639563 TBseq_1.0_1495_RIGHT 1 - AGAATGATTTGTTAGAGGAGGGGC

NC_000962.3 2637597 2637617 TBseq_1.0_1496_LEFT 2 + CTGAAGGTGAGCCAGAGACG

NC_000962.3 2639545 2639570 TBseq_1.0_1496_RIGHT 2 - GCGGTTGAGAATGATTTGTTAGAGG

NC_000962.3 2639868 2639891 TBseq_1.0_1497_LEFT 1 + TCGATGTTCCAGCATTTCCGTAA

NC_000962.3 2641873 2641892 TBseq_1.0_1497_RIGHT 1 - GTGGTGATGGTGCTCCGAG

NC_000962.3 2641241 2641262 TBseq_1.0_1498_LEFT 2 + CAATATGGTGACGTCCCCCTC

NC_000962.3 2643243 2643264 TBseq_1.0_1498_RIGHT 2 - AACCATGTCGCCATTGTCATG

NC_000962.3 2642657 2642684 TBseq_1.0_1499_LEFT 1 + AGAGCTTGTCCTGGAATATGTATTCAG

NC_000962.3 2644656 2644676 TBseq_1.0_1499_RIGHT 1 - CTCACAGTCGTCGGTAGCAG

NC_000962.3 2644431 2644451 TBseq_1.0_1500_LEFT 2 + AAGAGTTGGTGCGACGTTCC

NC_000962.3 2646439 2646458 TBseq_1.0_1500_RIGHT 2 - CAAGCGGCTCAACGACTTG

NC_000962.3 2646005 2646026 TBseq_1.0_1501_LEFT 1 + CTGACCTCGTCAATCACCACC

NC_000962.3 2647941 2647959 TBseq_1.0_1501_RIGHT 1 - TTGATGCCGATCAGCCGC

NC_000962.3 2647613 2647633 TBseq_1.0_1502_LEFT 2 + CTTTCAGGTACACCACGCCG

NC_000962.3 2649540 2649559 TBseq_1.0_1502_RIGHT 2 - CGATCGACGCCAACACCAT

NC_000962.3 2649041 2649061 TBseq_1.0_1503_LEFT 1 + GGTCAGCTCCGCGATATGGA

NC_000962.3 2651018 2651038 TBseq_1.0_1503_RIGHT 1 - TCGAACTGATCCTCGACGGC

NC_000962.3 2650771 2650790 TBseq_1.0_1504_LEFT 2 + CGAGCACCTGGTGGATTCG

NC_000962.3 2652740 2652760 TBseq_1.0_1504_RIGHT 2 - CGACGAAGGATTTCACGCCG

NC_000962.3 2652261 2652282 TBseq_1.0_1505_LEFT 1 + CGCAGCGATATCCACAATCCG

NC_000962.3 2654230 2654250 TBseq_1.0_1505_RIGHT 1 - CGCATAGGTCATGAGACGGC

NC_000962.3 2654019 2654042 TBseq_1.0_1506_LEFT 2 + CTTCTCAAGCGGCTATCCACAAA

NC_000962.3 2655998 2656019 TBseq_1.0_1506_RIGHT 2 - GTCGTATACCAGATGCAGCCG

NC_000962.3 2655329 2655349 TBseq_1.0_1507_LEFT 1 + GTCTCAGATACCGCCGCAAC

NC_000962.3 2657331 2657352 TBseq_1.0_1507_RIGHT 1 - TGGAGTCAGTACCTAGCCTGG

NC_000962.3 2657136 2657157 TBseq_1.0_1508_LEFT 2 + GTCCCAGAACTGTGCGATTGA

NC_000962.3 2659103 2659125 TBseq_1.0_1508_RIGHT 2 - GGGCTTTTTGCAGATGGGTCTA

NC_000962.3 2658925 2658944 TBseq_1.0_1509_LEFT 1 + TGTTGGGTAGCACCGGGAT

NC_000962.3 2660905 2660926 TBseq_1.0_1509_RIGHT 1 - CGCTAGAGAGTTTGTCGCACT

NC_000962.3 2659423 2659442 TBseq_1.0_1510_LEFT 2 + CCGTCACATGGCAACCGTG

NC_000962.3 2661419 2661438 TBseq_1.0_1510_RIGHT 2 - CGGCCCGACTCTGTTATCG

NC_000962.3 2661156 2661178 TBseq_1.0_1511_LEFT 1 + GGCACCGTATTGATGAACAGGC

NC_000962.3 2663157 2663175 TBseq_1.0_1511_RIGHT 1 - TCGTGGTGTTGCGCAACG

NC_000962.3 2662426 2662444 TBseq_1.0_1512_LEFT 2 + CGTTGGCAGCCGTTCGAT

NC_000962.3 2664391 2664411 TBseq_1.0_1512_RIGHT 2 - CGGCCTATGTTGTGCTGGAT

NC_000962.3 2664121 2664142 TBseq_1.0_1513_LEFT 1 + GGCGAATTCGAAGATCTCCCG

NC_000962.3 2666090 2666107 TBseq_1.0_1513_RIGHT 1 - GACGGCGGCGGATTCAA

NC_000962.3 2664965 2664986 TBseq_1.0_1514_LEFT 2 + GGACCGTAGAAGTTGTGCAGC

NC_000962.3 2666964 2666984 TBseq_1.0_1514_RIGHT 2 - ACTGCGTACCACCTACCCTG

NC_000962.3 2666665 2666685 TBseq_1.0_1515_LEFT 1 + TAGGTCAGCTCGCGAGTACG

NC_000962.3 2668695 2668714 TBseq_1.0_1515_RIGHT 1 - GGGACGCCGAGTTGATCCT

NC_000962.3 2668272 2668289 TBseq_1.0_1516_LEFT 2 + AGCACGCCGGAGTCCAA

NC_000962.3 2670267 2670287 TBseq_1.0_1516_RIGHT 2 - CGAGTGTGGGGAGGTGTGAT

NC_000962.3 2669956 2669975 TBseq_1.0_1517_LEFT 1 + CCTGGCCGGGAAAGACGAA

NC_000962.3 2671991 2672008 TBseq_1.0_1517_RIGHT 1 - GCAATGGCGTGCCGGTA

NC_000962.3 2671758 2671776 TBseq_1.0_1518_LEFT 2 + TGGATGTTGGCGCGGATG

NC_000962.3 2673742 2673764 TBseq_1.0_1518_RIGHT 2 - CTCGCCTATGTGTTGTTCACCT

NC_000962.3 2673494 2673516 TBseq_1.0_1519_LEFT 1 + CTCGTAAGTGTCGATAAGCCGG

NC_000962.3 2675555 2675574 TBseq_1.0_1519_RIGHT 1 - AGGGGATGCCGGGTTATCC

NC_000962.3 2675306 2675327 TBseq_1.0_1520_LEFT 2 + GATGGGAAAGTCGCGAGATCC

NC_000962.3 2677198 2677217 TBseq_1.0_1520_RIGHT 2 - GATCGAAGCAGCGCTCGTT

NC_000962.3 2677039 2677058 TBseq_1.0_1521_LEFT 1 + CCCGAAGTGGTGGAGCACA

NC_000962.3 2678956 2678975 TBseq_1.0_1521_RIGHT 1 - ATCCATCGAGCGACCGGAT

NC_000962.3 2678704 2678723 TBseq_1.0_1522_LEFT 2 + TCCTCGAATTCGCGCTCTG

NC_000962.3 2680698 2680718 TBseq_1.0_1522_RIGHT 2 - CAGGGGAGCGACCAGGAATA

NC_000962.3 2680092 2680110 TBseq_1.0_1523_LEFT 1 + TCGTCGGGAGCAAATGGC

NC_000962.3 2682000 2682019 TBseq_1.0_1523_RIGHT 1 - GCTAACCGATCGAAGCCCC

NC_000962.3 2681727 2681746 TBseq_1.0_1524_LEFT 2 + CTGTTTGCGACGCTTGGGA

NC_000962.3 2683793 2683812 TBseq_1.0_1524_RIGHT 2 - GCAACCGGAACAGCCCAAC

NC_000962.3 2683618 2683638 TBseq_1.0_1525_LEFT 1 + CAACGGCGGTTTCGATGAAC

NC_000962.3 2685561 2685580 TBseq_1.0_1525_RIGHT 1 - AATTTCAGCCGGGCCTTGG

NC_000962.3 2684827 2684850 TBseq_1.0_1526_LEFT 2 + CGAAAACATCTACGCCAAACAGG

NC_000962.3 2686893 2686914 TBseq_1.0_1526_RIGHT 2 - AACGTCTCATCGAAGCTGACC

NC_000962.3 2686651 2686673 TBseq_1.0_1527_LEFT 1 + GAAACAATCGGCACCAGAGATG

NC_000962.3 2688717 2688737 TBseq_1.0_1527_RIGHT 1 - AAATACTTGCGAGCCTCCGG

NC_000962.3 2688386 2688408 TBseq_1.0_1528_LEFT 2 + CGGCGGATATCTGGTGTACTTC

NC_000962.3 2690402 2690424 TBseq_1.0_1528_RIGHT 2 - GAGTACATGACGCCAAGGATCC

NC_000962.3 2690120 2690140 TBseq_1.0_1529_LEFT 1 + TCCTGGGCGCATTGATTACC

NC_000962.3 2692105 2692126 TBseq_1.0_1529_RIGHT 1 - TGTCAACCGTGAAGTTGCTGT

NC_000962.3 2690825 2690847 TBseq_1.0_1530_LEFT 2 + CCAGAATTTTCTCGACGGACGT

NC_000962.3 2692775 2692796 TBseq_1.0_1530_RIGHT 2 - GGCACCTCCAAAATGTCAGGA

NC_000962.3 2692363 2692381 TBseq_1.0_1531_LEFT 1 + TGCCGAACCAGCAGACGA

NC_000962.3 2694255 2694278 TBseq_1.0_1531_RIGHT 1 - CTTCGTGATGTCCTTCTTGGGAG

NC_000962.3 2694121 2694140 TBseq_1.0_1532_LEFT 2 + CACCCGATCCACAACAGCC

NC_000962.3 2696034 2696053 TBseq_1.0_1532_RIGHT 2 - ACGCCGGCATTGTTATCCG

NC_000962.3 2695735 2695755 TBseq_1.0_1533_LEFT 1 + GCACAAAGACGTACCCCAGC

NC_000962.3 2697692 2697714 TBseq_1.0_1533_RIGHT 1 - ATGCTCTCCTTGACGCTTTCTG

NC_000962.3 2696989 2697012 TBseq_1.0_1534_LEFT 2 + TTCTCGTAGCTGATCAACACGTC

NC_000962.3 2698934 2698952 TBseq_1.0_1534_RIGHT 2 - GTTCAACGGTGCCGCTGA

NC_000962.3 2698632 2698651 TBseq_1.0_1535_LEFT 1 + TTCGGTGGAGTGGCTGTGT

NC_000962.3 2700619 2700639 TBseq_1.0_1535_RIGHT 1 - TTCTTTGGCATCACCCACCC

NC_000962.3 2700414 2700434 TBseq_1.0_1536_LEFT 2 + CGACAGCTCGGGGATGTTTC

NC_000962.3 2702325 2702345 TBseq_1.0_1536_RIGHT 2 - GTGGGTTACCTGATCACCGG

NC_000962.3 2701767 2701786 TBseq_1.0_1537_LEFT 1 + GTGTAGCGCAGTTCCACCC

NC_000962.3 2703770 2703789 TBseq_1.0_1537_RIGHT 1 - ACCTCGCGTTCCAGAATCG

NC_000962.3 2703340 2703360 TBseq_1.0_1538_LEFT 2 + GAAACGCTTCGCACAATCCG

NC_000962.3 2705248 2705267 TBseq_1.0_1538_RIGHT 2 - TCACCGTGTGTACCAACGC

NC_000962.3 2704080 2704101 TBseq_1.0_1539_LEFT 1 + GTCACCGATGCTAACGATGCC

NC_000962.3 2705990 2706009 TBseq_1.0_1539_RIGHT 1 - CTATCGGTGTGGCGCCATG

NC_000962.3 2705756 2705774 TBseq_1.0_1540_LEFT 2 + AATACGTGCAGCGCGACC

NC_000962.3 2707824 2707843 TBseq_1.0_1540_RIGHT 2 - CTTCATGTTGCTTGGGCGC

NC_000962.3 2707330 2707353 TBseq_1.0_1541_LEFT 1 + CATCAGGATTCCTCTTGGCTTCC

NC_000962.3 2709370 2709390 TBseq_1.0_1541_RIGHT 1 - GTCGGGGGTGTCGTATGTGA

NC_000962.3 2708186 2708206 TBseq_1.0_1542_LEFT 2 + CGGGATCGACACTGGAATCC

NC_000962.3 2710191 2710209 TBseq_1.0_1542_RIGHT 2 - TGCCTTGTCGCCTGCATG

NC_000962.3 2709506 2709528 TBseq_1.0_1543_LEFT 1 + GTGAAAGTGCTCACAGGAGGTC

NC_000962.3 2711432 2711451 TBseq_1.0_1543_RIGHT 1 - GTGTTCACGGTTGCGCAGT

NC_000962.3 2710909 2710927 TBseq_1.0_1544_LEFT 2 + ATGAAGTCGGCGCGCTCA

NC_000962.3 2712972 2712991 TBseq_1.0_1544_RIGHT 2 - GGCAGCGCAATGGTAGGTT

NC_000962.3 2712577 2712597 TBseq_1.0_1545_LEFT 1 + AATGCCACGGTGATTGGGTG

NC_000962.3 2714597 2714616 TBseq_1.0_1545_RIGHT 1 - CGAACTCAGGGCGGTAACC

NC_000962.3 2713458 2713479 TBseq_1.0_1546_LEFT 2 + GCTGACATTACCGGCTCAGTC

NC_000962.3 2715527 2715546 TBseq_1.0_1546_RIGHT 2 - CCGGTACTTGCTCTGCACG

NC_000962.3 2715314 2715335 TBseq_1.0_1547_LEFT 1 + ACACAGGGTCACAGTCACAGA

NC_000962.3 2717243 2717260 TBseq_1.0_1547_RIGHT 1 - TTTGGGCGGCATGGGCA

NC_000962.3 2716984 2717006 TBseq_1.0_1548_LEFT 2 + GGCCAATCAGCTCTAAATCCCA

NC_000962.3 2718918 2718941 TBseq_1.0_1548_RIGHT 2 - GGCTTAGGAATAACCAATCGTGC

NC_000962.3 2718677 2718698 TBseq_1.0_1549_LEFT 1 + GAGACCTGTCGACCCTTTTGC

NC_000962.3 2720669 2720687 TBseq_1.0_1549_RIGHT 1 - ACCAGCCCCCAAATCCGA

NC_000962.3 2720408 2720430 TBseq_1.0_1550_LEFT 2 + GATGTATACCGGGATGTGCACG

NC_000962.3 2722405 2722425 TBseq_1.0_1550_RIGHT 2 - CGATCGACCTGGTGTTGCAC

NC_000962.3 2721424 2721445 TBseq_1.0_1551_LEFT 1 + GGGATCTTCGAGCGCATACTG

NC_000962.3 2723491 2723512 TBseq_1.0_1551_RIGHT 1 - GGAGTTAGTGCGCATCATCGG

NC_000962.3 2722727 2722747 TBseq_1.0_1552_LEFT 2 + ATCTGTTCCTGGGTGGCTGT

NC_000962.3 2724714 2724733 TBseq_1.0_1552_RIGHT 2 - GACGTGGCCGAGCGTATCT

NC_000962.3 2724277 2724299 TBseq_1.0_1553_LEFT 1 + CGTCAATTCCGGTAGTCCCATC

NC_000962.3 2726276 2726293 TBseq_1.0_1553_RIGHT 1 - TAGTCGCCGGGCTGCTT

NC_000962.3 2725220 2725239 TBseq_1.0_1554_LEFT 2 + ACTCGTTGCGGGTTCAAGG

NC_000962.3 2727288 2727306 TBseq_1.0_1554_RIGHT 2 - CGCTTGTGCAACGCCTGA

NC_000962.3 2726986 2727004 TBseq_1.0_1555_LEFT 1 + ACCATCTGTCGGCTGCAG

NC_000962.3 2728922 2728947 TBseq_1.0_1555_RIGHT 1 - TGATCAATTATTTGGTCCCGTTCCT

NC_000962.3 2728746 2728764 TBseq_1.0_1556_LEFT 2 + GCTGCGACGTCGTGGAAA

NC_000962.3 2730727 2730747 TBseq_1.0_1556_RIGHT 2 - TCGTCTACGACATGTGGGGC

NC_000962.3 2730502 2730522 TBseq_1.0_1557_LEFT 1 + GCAATCCGATCGCAATGCCA

NC_000962.3 2732448 2732471 TBseq_1.0_1557_RIGHT 1 - GACGAATTCGCTGGTCATTTACG

NC_000962.3 2732206 2732227 TBseq_1.0_1558_LEFT 2 + ATCTTGGTCCGACGTGAATGG

NC_000962.3 2734151 2734170 TBseq_1.0_1558_RIGHT 2 - GGGTCTCCTTCGGTGCGTA

NC_000962.3 2733769 2733788 TBseq_1.0_1559_LEFT 1 + CAGGACTTGGCCGCTATCG

NC_000962.3 2735766 2735786 TBseq_1.0_1559_RIGHT 1 - CTGGACTCGAGCAACGGTTG

NC_000962.3 2735476 2735497 TBseq_1.0_1560_LEFT 2 + ACGTCGTACACTTTTTCGCCA

NC_000962.3 2737393 2737411 TBseq_1.0_1560_RIGHT 2 - AACCGGCGCACTGACTCT

NC_000962.3 2737062 2737087 TBseq_1.0_1561_LEFT 1 + GCCATGTTGATCGAAATCATTTGGG

NC_000962.3 2739077 2739094 TBseq_1.0_1561_RIGHT 1 - TCGGTGTGGTCTCGGCT

NC_000962.3 2738675 2738695 TBseq_1.0_1562_LEFT 2 + CTGCGACAGCCCAAAGATCA

NC_000962.3 2740686 2740706 TBseq_1.0_1562_RIGHT 2 - GCGGTTCCTCCAGATCGACA

NC_000962.3 2740511 2740530 TBseq_1.0_1563_LEFT 1 + ACCTGCAACTACGGGGTCA

NC_000962.3 2742489 2742508 TBseq_1.0_1563_RIGHT 1 - GTGACGAGGAATCCGGGGA

NC_000962.3 2741929 2741947 TBseq_1.0_1564_LEFT 2 + AACGCCGTCGCAACCATC

NC_000962.3 2743829 2743852 TBseq_1.0_1564_RIGHT 2 - TCTACCTGGGAATTGTGCAGAAC

NC_000962.3 2743591 2743614 TBseq_1.0_1565_LEFT 1 + GCACATAAACCAGAAATCGACCG

NC_000962.3 2745659 2745678 TBseq_1.0_1565_RIGHT 1 - GGCAGCTGATCGGCGAGAT

NC_000962.3 2745517 2745537 TBseq_1.0_1566_LEFT 2 + ACCCGACGTGATGAACTCCA

NC_000962.3 2747487 2747507 TBseq_1.0_1566_RIGHT 2 - AGCATCTACTCGACCAACGC

NC_000962.3 2746093 2746113 TBseq_1.0_1567_LEFT 1 + GAATCCCTTCCACGGGTCAG

NC_000962.3 2748041 2748063 TBseq_1.0_1567_RIGHT 1 - CGCGCAACGGATTAACGATATG

NC_000962.3 2747933 2747953 TBseq_1.0_1568_LEFT 2 + GTTGCTCAGATCCGAGTCCC

NC_000962.3 2749918 2749939 TBseq_1.0_1568_RIGHT 2 - CGGTCGACGGCAAGACTAAAG

NC_000962.3 2749611 2749631 TBseq_1.0_1569_LEFT 1 + AGCGAGCCGTACCTAAACGA

NC_000962.3 2751546 2751565 TBseq_1.0_1569_RIGHT 1 - CTACGCCGCGTGAATTCGA

NC_000962.3 2751184 2751203 TBseq_1.0_1570_LEFT 2 + CGGCCTGCTTGTGGTACAA

NC_000962.3 2753096 2753115 TBseq_1.0_1570_RIGHT 2 - CTTCGGATGCACTGCGGAT

NC_000962.3 2752839 2752861 TBseq_1.0_1571_LEFT 1 + CAGCAGACTCAAATGTCCTCCA

NC_000962.3 2754806 2754826 TBseq_1.0_1571_RIGHT 1 - GGGAGATTGGCCGAGCTTGA

NC_000962.3 2754283 2754303 TBseq_1.0_1572_LEFT 2 + TCGTCACGTTGATGTTGCGG

NC_000962.3 2756354 2756374 TBseq_1.0_1572_RIGHT 2 - GCCAATATCGGCGACCTACC

NC_000962.3 2756092 2756111 TBseq_1.0_1573_LEFT 1 + GATGAAGGCCTCGCTGTGC

NC_000962.3 2758037 2758055 TBseq_1.0_1573_RIGHT 1 - AGCCTCGATCGGGTTCGT

NC_000962.3 2757762 2757782 TBseq_1.0_1574_LEFT 2 + CTTCGTTACGCCCAATCCGT

NC_000962.3 2759772 2759792 TBseq_1.0_1574_RIGHT 2 - CTGACCAATTCCACAACGCC

NC_000962.3 2759595 2759620 TBseq_1.0_1575_LEFT 1 + GTCCTAGAGATTTCAGGGTGTTCAC

NC_000962.3 2761603 2761625 TBseq_1.0_1575_RIGHT 1 - ACAGTTTGAGATCGACCAACGG

NC_000962.3 2761080 2761099 TBseq_1.0_1576_LEFT 2 + GTTCGGGGTCACGCTCTTC

NC_000962.3 2763079 2763100 TBseq_1.0_1576_RIGHT 2 - GGGGTCAAGGAGTCCAATCCA

NC_000962.3 2762595 2762615 TBseq_1.0_1577_LEFT 1 + CCTCTTCCGCGGTCAAGATC

NC_000962.3 2764533 2764553 TBseq_1.0_1577_RIGHT 1 - GACGAATTCGCGCAGTTAGC

NC_000962.3 2764381 2764400 TBseq_1.0_1578_LEFT 2 + GCACATACGACTCCGGCAA

NC_000962.3 2766350 2766372 TBseq_1.0_1578_RIGHT 2 - CAGCCTTGGGATGTTCGTTTAG

NC_000962.3 2766146 2766166 TBseq_1.0_1579_LEFT 1 + GGTTTCATCCTCGGCGAAGT

NC_000962.3 2768192 2768212 TBseq_1.0_1579_RIGHT 1 - GAATTCACTATCGGCGGGCG

NC_000962.3 2767699 2767719 TBseq_1.0_1580_LEFT 2 + CTTCGTGGGTCCGTTCGTAC

NC_000962.3 2769729 2769748 TBseq_1.0_1580_RIGHT 2 - CGTACTTGCCGAACGCGTA

NC_000962.3 2769410 2769429 TBseq_1.0_1581_LEFT 1 + CGACCAACCCGACCTCAAG

NC_000962.3 2771411 2771431 TBseq_1.0_1581_RIGHT 1 - TGTCCCAGTGCGGATACAGG

NC_000962.3 2770981 2771000 TBseq_1.0_1582_LEFT 2 + GGCATGTGCAGACCCTAGG

NC_000962.3 2773050 2773070 TBseq_1.0_1582_RIGHT 2 - CCGCGAATCATGGTGGACTG

NC_000962.3 2772620 2772639 TBseq_1.0_1583_LEFT 1 + CACAACAGAACCGGTCCCG

NC_000962.3 2774676 2774693 TBseq_1.0_1583_RIGHT 1 - ACCACCGCCATCGCCTT

NC_000962.3 2774469 2774487 TBseq_1.0_1584_LEFT 2 + TATCTGCGGCCCGACGAA

NC_000962.3 2776416 2776434 TBseq_1.0_1584_RIGHT 2 - AAACGATCAGCGCCGACG

NC_000962.3 2776192 2776212 TBseq_1.0_1585_LEFT 1 + ACTATCTCGCTGGAGCTCCC

NC_000962.3 2778192 2778212 TBseq_1.0_1585_RIGHT 1 - GATACCGCCGGCATCACCTA

NC_000962.3 2777986 2778005 TBseq_1.0_1586_LEFT 2 + GCGGACGGTAGTTGAGCAG

NC_000962.3 2779914 2779934 TBseq_1.0_1586_RIGHT 2 - TTGATCGACGAACTTCCGCT

NC_000962.3 2779446 2779466 TBseq_1.0_1587_LEFT 1 + CCCAGCCAGAACCCATAGGA

NC_000962.3 2781369 2781389 TBseq_1.0_1587_RIGHT 1 - GCCTATCCCTATGCCATCGC

NC_000962.3 2780782 2780799 TBseq_1.0_1588_LEFT 2 + AACGGTCGGCCCAGGTT

NC_000962.3 2782824 2782844 TBseq_1.0_1588_RIGHT 2 - AGACCGTCTGGGAGGTTGTC

NC_000962.3 2782626 2782645 TBseq_1.0_1589_LEFT 1 + GTCGTTCGTCGGTTCGTCG

NC_000962.3 2784668 2784687 TBseq_1.0_1589_RIGHT 1 - CGCTCAACGCCAGAGACCA

NC_000962.3 2784393 2784413 TBseq_1.0_1590_LEFT 2 + TGTCCCACCACAATCACCGG

NC_000962.3 2786434 2786454 TBseq_1.0_1590_RIGHT 2 - CTATTTCCGGAGTGGGAGGC

NC_000962.3 2786239 2786259 TBseq_1.0_1591_LEFT 1 + GGGTGACGGCGATAACATCC

NC_000962.3 2788190 2788208 TBseq_1.0_1591_RIGHT 1 - CGCGCACCGGAAAGATGT

NC_000962.3 2787721 2787744 TBseq_1.0_1592_LEFT 2 + TCCAGATAGTCCAGTGAGTCCTG

NC_000962.3 2789707 2789726 TBseq_1.0_1592_RIGHT 2 - ATCGTGCCGATCGTGATCC

NC_000962.3 2789373 2789393 TBseq_1.0_1593_LEFT 1 + GCCTTCTTTGTAGCCGCCTT

NC_000962.3 2791412 2791432 TBseq_1.0_1593_RIGHT 1 - GAGCCCGCGATGAACATCAT

NC_000962.3 2791042 2791062 TBseq_1.0_1594_LEFT 2 + CACTGAACCGGCTCCTTGAG

NC_000962.3 2793081 2793102 TBseq_1.0_1594_RIGHT 2 - TGCACGAAATGATGATGCCGT

NC_000962.3 2792945 2792965 TBseq_1.0_1595_LEFT 1 + GGCACCATCGGATCCTTCTC

NC_000962.3 2794833 2794854 TBseq_1.0_1595_RIGHT 1 - CGAAGCACATGCCGAACAATA

NC_000962.3 2797512 2797531 TBseq_1.0_1596_LEFT 2 + TGCTGCGCGAGTTGAAGAC

NC_000962.3 2799575 2799594 TBseq_1.0_1596_RIGHT 2 - GGCGATCGAACTTGCAGCG

NC_000962.3 2798626 2798645 TBseq_1.0_1597_LEFT 1 + CGAACCCGACATAGGCCTG

NC_000962.3 2800644 2800663 TBseq_1.0_1597_RIGHT 1 - CTCAGCTTGACACGACGGC

NC_000962.3 2806280 2806300 TBseq_1.0_1598_LEFT 2 + TGCGACTAGTGACCCCTTGG

NC_000962.3 2808261 2808281 TBseq_1.0_1598_RIGHT 2 - GGACCATCTCCGGTGTGATC

NC_000962.3 2808067 2808086 TBseq_1.0_1599_LEFT 1 + GCTAGCATCAGGGCTGTGC

NC_000962.3 2810051 2810071 TBseq_1.0_1599_RIGHT 1 - CTACCAATTGGAGGCTCCGG

NC_000962.3 2809864 2809883 TBseq_1.0_1600_LEFT 2 + CGTCACCGTCACTTCCTGC

NC_000962.3 2811866 2811885 TBseq_1.0_1600_RIGHT 2 - CGTTGTACACGCCCTGTCG

NC_000962.3 2811492 2811514 TBseq_1.0_1601_LEFT 1 + ACTGGTTGTTCTGCACGTAGAA

NC_000962.3 2813410 2813429 TBseq_1.0_1601_RIGHT 1 - TGTCGGTTGCGCAGTTGAC

NC_000962.3 2812906 2812927 TBseq_1.0_1602_LEFT 2 + CAGCATCACCGTGGTATAGGC

NC_000962.3 2814968 2814988 TBseq_1.0_1602_RIGHT 2 - ACGGGTACAGGTGTTGGTGT

NC_000962.3 2814736 2814756 TBseq_1.0_1603_LEFT 1 + ACAGGCCCATCTCTCCCATC

NC_000962.3 2816739 2816756 TBseq_1.0_1603_RIGHT 1 - TCTTGGAGGCGGACGCT

NC_000962.3 2816496 2816515 TBseq_1.0_1604_LEFT 2 + AACATCGAAAGCGGCGACC

NC_000962.3 2818448 2818469 TBseq_1.0_1604_RIGHT 2 - CACCCCGTCGATAGCAATAGC

NC_000962.3 2817734 2817752 TBseq_1.0_1605_LEFT 1 + TCGTCGTCGTCAGCCAGA

NC_000962.3 2819668 2819691 TBseq_1.0_1605_RIGHT 1 - TCTGGGACTATTGTTGCAACACA

NC_000962.3 2819501 2819521 TBseq_1.0_1606_LEFT 2 + GACCCCTGCCGGTGTATAGA

NC_000962.3 2821470 2821489 TBseq_1.0_1606_RIGHT 2 - TGCCAGGTACGGACATCGG

NC_000962.3 2821015 2821037 TBseq_1.0_1607_LEFT 1 + TGGATGTTTACCGGATCACTGC

NC_000962.3 2823048 2823069 TBseq_1.0_1607_RIGHT 1 - ACGTTGCCGCTATCCATGTAC

NC_000962.3 2822465 2822484 TBseq_1.0_1608_LEFT 2 + TTGGTCCCCCTCTATCCGG

NC_000962.3 2824512 2824531 TBseq_1.0_1608_RIGHT 2 - GTGGGATTGCGGTTCCACC

NC_000962.3 2824147 2824167 TBseq_1.0_1609_LEFT 1 + GTGCTGGGTGTGGACAGTTG

NC_000962.3 2826125 2826145 TBseq_1.0_1609_RIGHT 1 - GCAGACCGTGAAGCTGATCC

NC_000962.3 2825547 2825567 TBseq_1.0_1610_LEFT 2 + GTTGAGGACGCTCTTGACGG

NC_000962.3 2827438 2827455 TBseq_1.0_1610_RIGHT 2 - GCGGTCTTGGGCTGCTT

NC_000962.3 2827240 2827261 TBseq_1.0_1611_LEFT 1 + GTCACCGATGCCGATCTGAAC

NC_000962.3 2829201 2829221 TBseq_1.0_1611_RIGHT 1 - TACCGAGAGATCCTGGGCAT

NC_000962.3 2828884 2828903 TBseq_1.0_1612_LEFT 2 + CGGGGAGTTTGTCGGTCAG

NC_000962.3 2830873 2830895 TBseq_1.0_1612_RIGHT 2 - GCAGGGATGGACTTTCTGAGAG

NC_000962.3 2830146 2830169 TBseq_1.0_1613_LEFT 1 + CAGAGAGAAACAAGGTGGACGAC

NC_000962.3 2832090 2832110 TBseq_1.0_1613_RIGHT 1 - AGGCCTTGATCGAGGTCAGC

NC_000962.3 2831923 2831943 TBseq_1.0_1614_LEFT 2 + ACGATCACCGGCAATTCGTC

NC_000962.3 2833818 2833840 TBseq_1.0_1614_RIGHT 2 - CGAATGAGTCCGGCTAAAACCT

NC_000962.3 2833572 2833592 TBseq_1.0_1615_LEFT 1 + CAGGCCCGCTTTCTTCAACG

NC_000962.3 2835635 2835652 TBseq_1.0_1615_RIGHT 1 - GTAACCGGCGTGCACCT

NC_000962.3 2835481 2835501 TBseq_1.0_1616_LEFT 2 + TAGGTAATCGAGGCGCCGTT

NC_000962.3 2837406 2837425 TBseq_1.0_1616_RIGHT 2 - TCGTCGTCGTGGTCATCCA

NC_000962.3 2837290 2837310 TBseq_1.0_1617_LEFT 1 + CCATAAGGGCGTCACGTTCG

NC_000962.3 2839321 2839339 TBseq_1.0_1617_RIGHT 1 - TGTTGTCGCAGGCCGGTT

NC_000962.3 2838662 2838683 TBseq_1.0_1618_LEFT 2 + GCCGATTTCCGATACTCCGTC

NC_000962.3 2840721 2840739 TBseq_1.0_1618_RIGHT 2 - ACGTGGCGGTCATCTCCA

NC_000962.3 2840981 2841000 TBseq_1.0_1619_LEFT 1 + TTGGGCCGGGAAAACTTCG

NC_000962.3 2842965 2842984 TBseq_1.0_1619_RIGHT 1 - GAAGTTGCCGTGGTGACCG

NC_000962.3 2842707 2842727 TBseq_1.0_1620_LEFT 2 + TGATGTGAATCGACTGCGGC

NC_000962.3 2844657 2844677 TBseq_1.0_1620_RIGHT 2 - ACGGTCGTTCATTTTGGTGC

NC_000962.3 2843179 2843199 TBseq_1.0_1621_LEFT 1 + CCACAGCTTCACCAGGTCCT

NC_000962.3 2845167 2845187 TBseq_1.0_1621_RIGHT 1 - CAACCCGACCAGCATCATCG

NC_000962.3 2844875 2844892 TBseq_1.0_1622_LEFT 2 + GCCGCCAGCCGATAGTT

NC_000962.3 2846935 2846956 TBseq_1.0_1622_RIGHT 2 - CAGACGCTATTCACCGATGCT

NC_000962.3 2845398 2845418 TBseq_1.0_1623_LEFT 1 + CCACGTCCACAATCTCTGCG

NC_000962.3 2847394 2847413 TBseq_1.0_1623_RIGHT 1 - GGCCACCAAGGAATCCACC

NC_000962.3 2847072 2847091 TBseq_1.0_1624_LEFT 2 + TCGAGTTGCCTTCCCCGAT

NC_000962.3 2849040 2849058 TBseq_1.0_1624_RIGHT 2 - AATGGGTACGCGCACTGG

NC_000962.3 2848825 2848843 TBseq_1.0_1625_LEFT 1 + AAACAGCTCGACGTCGCG

NC_000962.3 2850880 2850899 TBseq_1.0_1625_RIGHT 1 - CACGTCCAGGTTCTGCCAG

NC_000962.3 2850702 2850722 TBseq_1.0_1626_LEFT 2 + CTGCGACCACTACTACGGTG

NC_000962.3 2852677 2852699 TBseq_1.0_1626_RIGHT 2 - TGACAAGCCAGCTGGATAATGG

NC_000962.3 2851740 2851764 TBseq_1.0_1627_LEFT 1 + GCGATAATCCTTACAGGCCATCAA

NC_000962.3 2853713 2853733 TBseq_1.0_1627_RIGHT 1 - AGGTGTAGGGGTAGCGTTCG

NC_000962.3 2852713 2852735 TBseq_1.0_1628_LEFT 2 + CCAAGTTGGGTAAGTCCATCCG

NC_000962.3 2854764 2854784 TBseq_1.0_1628_RIGHT 2 - GGGATTGTCGAGGTTGACGG

NC_000962.3 2854450 2854469 TBseq_1.0_1629_LEFT 1 + GTGTGCCCGGCAAGAGAAG

NC_000962.3 2856511 2856531 TBseq_1.0_1629_RIGHT 1 - GTACCCGATGTACCTCGACG

NC_000962.3 2856176 2856199 TBseq_1.0_1630_LEFT 2 + ACACCAATTCCGGTATTCCTCAG

NC_000962.3 2858155 2858175 TBseq_1.0_1630_RIGHT 2 - CCTACGGGCTGACCATTGTC

NC_000962.3 2857066 2857086 TBseq_1.0_1631_LEFT 1 + GCAACTCACGGATCCACTCG

NC_000962.3 2859114 2859132 TBseq_1.0_1631_RIGHT 1 - GTTCAACGCCGGGGTGAA

NC_000962.3 2859180 2859197 TBseq_1.0_1632_LEFT 2 + ACGAACGCCGGACCCTT

NC_000962.3 2861159 2861179 TBseq_1.0_1632_RIGHT 2 - TGCGGTACCTAGCTGAGCAT

NC_000962.3 2860026 2860046 TBseq_1.0_1633_LEFT 1 + AGTGACTCCACAGTTCCGGA

NC_000962.3 2862062 2862085 TBseq_1.0_1633_RIGHT 1 - ATCCTGGATCTCATCGAAGCTGA

NC_000962.3 2861778 2861796 TBseq_1.0_1634_LEFT 2 + AGGACAGGATGGTGCGGT

NC_000962.3 2863759 2863779 TBseq_1.0_1634_RIGHT 2 - GCCAAGAAAGACGGCGACAC

NC_000962.3 2862891 2862911 TBseq_1.0_1635_LEFT 1 + GGCGCTGATCTCCAGGTAGA

NC_000962.3 2864949 2864967 TBseq_1.0_1635_RIGHT 1 - ATCGAATCGCGCACCTCG

NC_000962.3 2865575 2865594 TBseq_1.0_1636_LEFT 2 + CATCCAAGAGGCGCTGAGG

NC_000962.3 2867537 2867558 TBseq_1.0_1636_RIGHT 2 - TTCCTCTCGCACGATATTGGC

NC_000962.3 2867296 2867316 TBseq_1.0_1637_LEFT 1 + CCACAAAGGCTCCTACGAGG

NC_000962.3 2869230 2869250 TBseq_1.0_1637_RIGHT 1 - CCCCAGGGAGTGCTCAGTAC

NC_000962.3 2868527 2868548 TBseq_1.0_1638_LEFT 2 + CCAACCTACGGAGTGGATCGT

NC_000962.3 2870514 2870534 TBseq_1.0_1638_RIGHT 2 - GCTTCGCCCCTGTACAGAAG

NC_000962.3 2870034 2870052 TBseq_1.0_1639_LEFT 1 + TGCTGGCCCACAACCTCT

NC_000962.3 2872096 2872116 TBseq_1.0_1639_RIGHT 1 - ACCATCGATTCCCAGGGCAC

NC_000962.3 2870769 2870788 TBseq_1.0_1640_LEFT 2 + CAATCTCAGCCCAGCAGCG

NC_000962.3 2872799 2872818 TBseq_1.0_1640_RIGHT 2 - CACAACCGGGTGGGGAAGT

NC_000962.3 2872362 2872382 TBseq_1.0_1641_LEFT 1 + ATCCTGGGTGTTGGCTTCCT

NC_000962.3 2874425 2874445 TBseq_1.0_1641_RIGHT 1 - CCGTTCTCGCTGGAGCTATG

NC_000962.3 2873655 2873673 TBseq_1.0_1642_LEFT 2 + TGCAAGCCACGCCGATAC

NC_000962.3 2875699 2875719 TBseq_1.0_1642_RIGHT 2 - TCATCGATACCGTGGCCAGG

NC_000962.3 2875318 2875338 TBseq_1.0_1643_LEFT 1 + GGTGGCTCCGGATTTCTTGG

NC_000962.3 2877301 2877320 TBseq_1.0_1643_RIGHT 1 - GCTTGCATGCATGGCTTCC

NC_000962.3 2877129 2877147 TBseq_1.0_1644_LEFT 2 + ACGCGCGCTCTACCACTA

NC_000962.3 2879092 2879110 TBseq_1.0_1644_RIGHT 2 - AAATCGGTGCGCGGTTCC

NC_000962.3 2878784 2878802 TBseq_1.0_1645_LEFT 1 + AGATGAGCCGGCACCAGA

NC_000962.3 2880830 2880849 TBseq_1.0_1645_RIGHT 1 - CGATGTTGGACCCGACCGT

NC_000962.3 2880579 2880598 TBseq_1.0_1646_LEFT 2 + TGCACGCCGGAATTCTGAC

NC_000962.3 2882597 2882615 TBseq_1.0_1646_RIGHT 2 - GTGACGTTTCGCGCTGAC

NC_000962.3 2882306 2882325 TBseq_1.0_1647_LEFT 1 + GCGTGATGTCCAATGGCGA

NC_000962.3 2884213 2884230 TBseq_1.0_1647_RIGHT 1 - TGCGAGCCCGTACCGTT

NC_000962.3 2883834 2883852 TBseq_1.0_1648_LEFT 2 + TCCTTGCCGACGAACCGA

NC_000962.3 2885743 2885764 TBseq_1.0_1648_RIGHT 2 - GTGAGGGCGGATATTGGGATC

NC_000962.3 2885561 2885579 TBseq_1.0_1649_LEFT 1 + GGACTGGCGCATTTCGGT

NC_000962.3 2887548 2887566 TBseq_1.0_1649_RIGHT 1 - GGATGCCCGTTCGCGTTT

NC_000962.3 2887380 2887399 TBseq_1.0_1650_LEFT 2 + TTGCCCTACACCGACGAGT

NC_000962.3 2889392 2889411 TBseq_1.0_1650_RIGHT 2 - GCCGTTACAGGTCACCACG

NC_000962.3 2889120 2889144 TBseq_1.0_1651_LEFT 1 + CACTTCTTGATTCATGACATCGCC

NC_000962.3 2891027 2891047 TBseq_1.0_1651_RIGHT 1 - CCGACAAGAGTTTCCCCGCT

NC_000962.3 2890650 2890669 TBseq_1.0_1652_LEFT 2 + TAGTGGAAAGCGCGGACCT

NC_000962.3 2892540 2892562 TBseq_1.0_1652_RIGHT 2 - GACCTATACCCCTTTGTGCTGC

NC_000962.3 2891152 2891170 TBseq_1.0_1653_LEFT 1 + CAACCATGCCGGGGTGTT

NC_000962.3 2893051 2893070 TBseq_1.0_1653_RIGHT 1 - GACCACCGGACACCAGAAC

NC_000962.3 2893317 2893337 TBseq_1.0_1654_LEFT 2 + GGAGATTGGCGCAGAGTTGG

NC_000962.3 2895292 2895311 TBseq_1.0_1654_RIGHT 2 - ATCAGCAGCTGAACGGGCT

NC_000962.3 2894958 2894975 TBseq_1.0_1655_LEFT 1 + GACGATATCGGCGCGCA

NC_000962.3 2897008 2897027 TBseq_1.0_1655_RIGHT 1 - GTGGGCGCTGATAGGCTAC

NC_000962.3 2896857 2896878 TBseq_1.0_1656_LEFT 2 + CATACGGTGCCTGGAAGACAC

NC_000962.3 2898842 2898861 TBseq_1.0_1656_RIGHT 2 - AAGAGGTAAGGCGCGGTCT

NC_000962.3 2899036 2899058 TBseq_1.0_1657_LEFT 1 + GAAGAGTTCATTTCGTGGGAGC

NC_000962.3 2900947 2900966 TBseq_1.0_1657_RIGHT 1 - CTTTCGGGGGTGAATCGGC

NC_000962.3 2900226 2900246 TBseq_1.0_1658_LEFT 2 + TACCAGGGACGAGTCGAGCA

NC_000962.3 2902298 2902319 TBseq_1.0_1658_RIGHT 2 - GCACGAATATGGACGGCTTAC

NC_000962.3 2901715 2901737 TBseq_1.0_1659_LEFT 1 + GCCTATCAGACCTACTTTGCGG

NC_000962.3 2903633 2903653 TBseq_1.0_1659_RIGHT 1 - GCCGAATGCTGTCATAAGGC

NC_000962.3 2903400 2903419 TBseq_1.0_1660_LEFT 2 + TCGTGGAAGGTGATGCCGT

NC_000962.3 2905331 2905354 TBseq_1.0_1660_RIGHT 2 - CTTGGAGTGCCCTATGTCATCAA

NC_000962.3 2904894 2904913 TBseq_1.0_1661_LEFT 1 + AGTCCTTCACTGCGACCGT

NC_000962.3 2906887 2906907 TBseq_1.0_1661_RIGHT 1 - CGAGTTTGCGTTTGGCTGTG

NC_000962.3 2906718 2906738 TBseq_1.0_1662_LEFT 2 + ACAGTTACACGCCAGCAACC

NC_000962.3 2908661 2908681 TBseq_1.0_1662_RIGHT 2 - TGTCGAGGTTTTCACGTCCA

NC_000962.3 2908484 2908502 TBseq_1.0_1663_LEFT 1 + TGACCAAGCGCTGCAACG

NC_000962.3 2910373 2910397 TBseq_1.0_1663_RIGHT 1 - GATCATTGACGACGTGTTAGCAAC

NC_000962.3 2909970 2909990 TBseq_1.0_1664_LEFT 2 + GGGATAGATCTCCCGGTGCA

NC_000962.3 2912033 2912053 TBseq_1.0_1664_RIGHT 2 - CAGATTGCGCGACTGTGGAA

NC_000962.3 2911895 2911915 TBseq_1.0_1665_LEFT 1 + GTGCCCCACCAACGATCATT

NC_000962.3 2913863 2913884 TBseq_1.0_1665_RIGHT 1 - GGCACCATAGCTTCCTTTCGC

NC_000962.3 2913581 2913601 TBseq_1.0_1666_LEFT 2 + GCGTCTCCGAACGAATCTGC

NC_000962.3 2915505 2915523 TBseq_1.0_1666_RIGHT 2 - CAAATCATCAGCGCGCGG

NC_000962.3 2914949 2914968 TBseq_1.0_1667_LEFT 1 + GCAGGTACGCGGTCTTGTG

NC_000962.3 2916935 2916955 TBseq_1.0_1667_RIGHT 1 - AGTTGCTTATCGAGGAGGCC

NC_000962.3 2916763 2916782 TBseq_1.0_1668_LEFT 2 + GATCGCACGCTCCTACACC

NC_000962.3 2918717 2918738 TBseq_1.0_1668_RIGHT 2 - GGCTCATCGGCATGAAGTTCA

NC_000962.3 2918222 2918243 TBseq_1.0_1669_LEFT 1 + GCTTCAACAGCGTCGACTACA

NC_000962.3 2920199 2920218 TBseq_1.0_1669_RIGHT 1 - TAGCGACCCAAAAAGCCGG

NC_000962.3 2919525 2919544 TBseq_1.0_1670_LEFT 2 + GCTTTCTCGCCATGGCATC

NC_000962.3 2921487 2921511 TBseq_1.0_1670_RIGHT 2 - GCCCTGATGATCAAGACTCTTTCA

NC_000962.3 2921292 2921313 TBseq_1.0_1671_LEFT 1 + CGGTCCGGACAAAGACATTCC

NC_000962.3 2923209 2923226 TBseq_1.0_1671_RIGHT 1 - AGCGAGTCAACCGGGGT

NC_000962.3 2923803 2923822 TBseq_1.0_1672_LEFT 2 + GACCTTTGCCGACCACCAC

NC_000962.3 2925842 2925863 TBseq_1.0_1672_RIGHT 2 - CCGAATAGGTTTCCACAGCCG

NC_000962.3 2925639 2925658 TBseq_1.0_1673_LEFT 1 + TTCGGGAAGGCTCGGTTCT

NC_000962.3 2927588 2927609 TBseq_1.0_1673_RIGHT 1 - GGGATTCTTGGCGATAGTGCG

NC_000962.3 2927234 2927256 TBseq_1.0_1674_LEFT 2 + TACCACCTCCAATCCCATACCG

NC_000962.3 2929252 2929272 TBseq_1.0_1674_RIGHT 2 - GACCAGGCTTTCGGTGTAGC

NC_000962.3 2928857 2928876 TBseq_1.0_1675_LEFT 1 + CCTCAACGCGGCCGACTAT

NC_000962.3 2930866 2930886 TBseq_1.0_1675_RIGHT 1 - AGAAGACAGTGACGACGTGC

NC_000962.3 2930415 2930437 TBseq_1.0_1676_LEFT 2 + GCATGGCTAGACACCATCAACG

NC_000962.3 2932460 2932480 TBseq_1.0_1676_RIGHT 2 - GTGGTTCATGCGACCCTTCC

NC_000962.3 2932165 2932183 TBseq_1.0_1677_LEFT 1 + CCACCGCGTCAAGTTCGT

NC_000962.3 2934226 2934251 TBseq_1.0_1677_RIGHT 1 - CATCCTTGCCAATTGGTCTTTATCG

NC_000962.3 2932919 2932939 TBseq_1.0_1678_LEFT 2 + GAAAAACCGTGCGCTCCTTG

NC_000962.3 2934907 2934927 TBseq_1.0_1678_RIGHT 2 - GCAAGATCTGCGAGGTTCCG

NC_000962.3 2934904 2934924 TBseq_1.0_1679_LEFT 1 + TGACGGAACCTCGCAGATCT

NC_000962.3 2936890 2936910 TBseq_1.0_1679_RIGHT 1 - TGCAACTAGGCGAGCTGTTG

NC_000962.3 2936888 2936908 TBseq_1.0_1680_LEFT 2 + GGCAACAGCTCGCCTAGTTG

NC_000962.3 2938839 2938862 TBseq_1.0_1680_RIGHT 2 - TGCCTGATTACTTTGTCTCCGGT

NC_000962.3 2938524 2938543 TBseq_1.0_1681_LEFT 1 + CAACGCCTCCATCTGCCAG

NC_000962.3 2940421 2940441 TBseq_1.0_1681_RIGHT 1 - GCGTGTGTGGTGTGGTTCTT

NC_000962.3 2940274 2940295 TBseq_1.0_1682_LEFT 2 + CGCATGTGAAACGCTATCCAC

NC_000962.3 2942245 2942265 TBseq_1.0_1682_RIGHT 2 - GGACATCTGGACTGGTACGC

NC_000962.3 2941481 2941501 TBseq_1.0_1683_LEFT 1 + GATAGGCGACGTGCTCATCG

NC_000962.3 2943451 2943470 TBseq_1.0_1683_RIGHT 1 - TCGACGTCCTGCTGGGTAG

NC_000962.3 2942995 2943012 TBseq_1.0_1684_LEFT 2 + ACGTGCGCGGTCGAATG

NC_000962.3 2944993 2945011 TBseq_1.0_1684_RIGHT 2 - ACATCGTGGCGGTCAGGA

NC_000962.3 2943546 2943568 TBseq_1.0_1685_LEFT 1 + TACCGTAGCAACTCGCAAATCC

NC_000962.3 2945530 2945547 TBseq_1.0_1685_RIGHT 1 - ACGACGGCGGTTTCGGA

NC_000962.3 2945227 2945247 TBseq_1.0_1686_LEFT 2 + CCCCAGTGAATCTCCGGACG

NC_000962.3 2947282 2947301 TBseq_1.0_1686_RIGHT 2 - TGTCCGAGCATGACAGCCC

NC_000962.3 2947121 2947141 TBseq_1.0_1687_LEFT 1 + GGGATTTCAGCACGGTCAGC

NC_000962.3 2949025 2949044 TBseq_1.0_1687_RIGHT 1 - TGTGTTTAGCCGCGTTGCC

NC_000962.3 2948629 2948650 TBseq_1.0_1688_LEFT 2 + GGGTCTATGGCCAACAAACGT

NC_000962.3 2950707 2950725 TBseq_1.0_1688_RIGHT 2 - CCGGACGGCGAATTCGAA

NC_000962.3 2949863 2949884 TBseq_1.0_1689_LEFT 1 + TCCACAGTGAAATCGTTCCGG

NC_000962.3 2951879 2951899 TBseq_1.0_1689_RIGHT 1 - TGGGATTGGCCGAGTTTGTG

NC_000962.3 2951566 2951586 TBseq_1.0_1690_LEFT 2 + CACCTACGCTGGTAGTGCTG

NC_000962.3 2953599 2953619 TBseq_1.0_1690_RIGHT 2 - GCGGTCACACTGGCTTCTTC

NC_000962.3 2952725 2952745 TBseq_1.0_1691_LEFT 1 + TCTCCTGGATGCTTGCGTTC

NC_000962.3 2954631 2954652 TBseq_1.0_1691_RIGHT 1 - GGTCTGTACGTCGCTCAATCG

NC_000962.3 2954438 2954460 TBseq_1.0_1692_LEFT 2 + TGCGATTCCCATAGAACTCCAG

NC_000962.3 2956390 2956409 TBseq_1.0_1692_RIGHT 2 - GCTGCGGCAATGTGGAAAG

NC_000962.3 2955835 2955855 TBseq_1.0_1693_LEFT 1 + TCGCACGACACTCTTGATGC

NC_000962.3 2957736 2957756 TBseq_1.0_1693_RIGHT 1 - CCGACCAAGAGTCTTACGCC

NC_000962.3 2957329 2957348 TBseq_1.0_1694_LEFT 2 + GCTAGTTCAGGTGGGTGCC

NC_000962.3 2959325 2959345 TBseq_1.0_1694_RIGHT 2 - AGGCCATCTAGCGGTTGTCA

NC_000962.3 2962491 2962511 TBseq_1.0_1695_LEFT 1 + CACTCGGCAGCAACAAATCC

NC_000962.3 2964419 2964440 TBseq_1.0_1695_RIGHT 1 - GGGACTAGAGCGTGGTTCTGT

NC_000962.3 2964143 2964160 TBseq_1.0_1696_LEFT 2 + GGCATTACGGCCGCGAT

NC_000962.3 2966128 2966146 TBseq_1.0_1696_RIGHT 2 - TGCTCGAGCCGACTTCCA

NC_000962.3 2965871 2965893 TBseq_1.0_1697_LEFT 1 + AAGAGATGTTGCACACTGGGTT

NC_000962.3 2967925 2967946 TBseq_1.0_1697_RIGHT 1 - GACACATAGACCAACCCCACC

NC_000962.3 2967620 2967642 TBseq_1.0_1698_LEFT 2 + CTCTACGGTTTGCTGTTCACCA

NC_000962.3 2969657 2969676 TBseq_1.0_1698_RIGHT 2 - TACGTTGCGGCTCTAGGCT

NC_000962.3 2969470 2969492 TBseq_1.0_1699_LEFT 1 + CCAACGAATAATGCTCCCTGAC

NC_000962.3 2971481 2971503 TBseq_1.0_1699_RIGHT 1 - GCTGTGTAGCGTTCTGTTGTCA

NC_000962.3 2971295 2971314 TBseq_1.0_1700_LEFT 2 + ACGGCTACCTGTTCCCCAA

NC_000962.3 2973239 2973261 TBseq_1.0_1700_RIGHT 2 - GGTTTGATCAGCTCGGTCTTGT

NC_000962.3 2973102 2973124 TBseq_1.0_1701_LEFT 1 + CCACCATACGGATAGGGGATCT

NC_000962.3 2975089 2975108 TBseq_1.0_1701_RIGHT 1 - CGTTTTCAGGCGCTGACAC

NC_000962.3 2974846 2974864 TBseq_1.0_1702_LEFT 2 + CGGTGCGGCACAAGGTTT

NC_000962.3 2976874 2976893 TBseq_1.0_1702_RIGHT 2 - CTAAACGCCAGCCAGCCAT

NC_000962.3 2976580 2976602 TBseq_1.0_1703_LEFT 1 + GGTTTTCACTGTTTGCTGTCGG

NC_000962.3 2978545 2978568 TBseq_1.0_1703_RIGHT 1 - CTCGACGACATTAGGGCATTCTT

NC_000962.3 2978382 2978402 TBseq_1.0_1704_LEFT 2 + CGGTTCCGTGACTTCGAGTG

NC_000962.3 2980329 2980349 TBseq_1.0_1704_RIGHT 2 - TGATCGACTCCAACCCCTGC

NC_000962.3 2979942 2979961 TBseq_1.0_1705_LEFT 1 + TAGGTGACGGTTGGGGTCG

NC_000962.3 2981994 2982016 TBseq_1.0_1705_RIGHT 1 - GGGATGACCAGCTCTAAAAGGC

NC_000962.3 2981718 2981739 TBseq_1.0_1706_LEFT 2 + GTTTGCGCTGGTGAGAAACAG

NC_000962.3 2983700 2983717 TBseq_1.0_1706_RIGHT 2 - AAGAACGCCAGCCAGCC

NC_000962.3 2983461 2983482 TBseq_1.0_1707_LEFT 1 + AGCTTTCCAAGTCGCAAGTGT

NC_000962.3 2985525 2985545 TBseq_1.0_1707_RIGHT 1 - CCATGATAACCCGGCAGCAG

NC_000962.3 2985285 2985302 TBseq_1.0_1708_LEFT 2 + ACCGACGCCGACGAGTT

NC_000962.3 2987357 2987377 TBseq_1.0_1708_RIGHT 2 - GACACGAGCACTGCCTCATC

NC_000962.3 2986927 2986950 TBseq_1.0_1709_LEFT 1 + GCTTTACGACTATCCGGATGACG

NC_000962.3 2988982 2989002 TBseq_1.0_1709_RIGHT 1 - AGTGCACGCATTTCACCAGG

NC_000962.3 2988840 2988860 TBseq_1.0_1710_LEFT 2 + CCGATACCACCCGGGATACG

NC_000962.3 2990831 2990850 TBseq_1.0_1710_RIGHT 2 - ACTGGTAGATGCCCGCTGT

NC_000962.3 2989900 2989921 TBseq_1.0_1711_LEFT 1 + TTCTCAAACCCCTGCTCGGTC

NC_000962.3 2991966 2991984 TBseq_1.0_1711_RIGHT 1 - TTTCACCGGTCCGCGAGT

NC_000962.3 2991166 2991189 TBseq_1.0_1712_LEFT 2 + GTCAGTCTCGGTGAAAGTCAGTC

NC_000962.3 2993228 2993245 TBseq_1.0_1712_RIGHT 2 - GTGCTGCGCGACGAAAC

NC_000962.3 2992863 2992883 TBseq_1.0_1713_LEFT 1 + CGAACACCGTGACCAAGTCG

NC_000962.3 2994892 2994911 TBseq_1.0_1713_RIGHT 1 - CCTGTTTCGAACCGGACGT

NC_000962.3 2994308 2994327 TBseq_1.0_1714_LEFT 2 + GCAATTCGGCGGTCCCAAC

NC_000962.3 2996344 2996362 TBseq_1.0_1714_RIGHT 2 - CATGATCAGCCGGCCGAA

NC_000962.3 2995481 2995498 TBseq_1.0_1715_LEFT 1 + TGGTTGTCGCGCTGCTC

NC_000962.3 2997440 2997460 TBseq_1.0_1715_RIGHT 1 - TGGATACCCGAGGTTCGTCG

NC_000962.3 2996949 2996969 TBseq_1.0_1716_LEFT 2 + ACCTGATTCAGATCCGGCGG

NC_000962.3 2998966 2998986 TBseq_1.0_1716_RIGHT 2 - CCCAGAAACGCCGTGACATC

NC_000962.3 2998453 2998474 TBseq_1.0_1717_LEFT 1 + CAACAGGACGTCGTGGTTCAA

NC_000962.3 3000525 3000542 TBseq_1.0_1717_RIGHT 1 - ACCACCGCGAGCAATGG

NC_000962.3 3000250 3000272 TBseq_1.0_1718_LEFT 2 + ATCGTCTGCCTGGACTATTGGT

NC_000962.3 3002224 3002243 TBseq_1.0_1718_RIGHT 2 - GCGTTTGACGGCCCAAATC

NC_000962.3 3001920 3001940 TBseq_1.0_1719_LEFT 1 + AGACCTGTAGCACCAGACGA

NC_000962.3 3003960 3003979 TBseq_1.0_1719_RIGHT 1 - CATTCGAGGCACCTACCGC

NC_000962.3 3003239 3003258 TBseq_1.0_1720_LEFT 2 + CTGTGGCTGCGGTACTTCG

NC_000962.3 3005203 3005225 TBseq_1.0_1720_RIGHT 2 - CCTAGTGGGCAAGTACTCCAAG

NC_000962.3 3004818 3004836 TBseq_1.0_1721_LEFT 1 + CGTGGTGGTTTCGCAGCA

NC_000962.3 3006898 3006916 TBseq_1.0_1721_RIGHT 1 - CGCGCAACGTGGCTCTTA

NC_000962.3 3006588 3006607 TBseq_1.0_1722_LEFT 2 + GTCGGTTACCAGCTCGCCA

NC_000962.3 3008567 3008589 TBseq_1.0_1722_RIGHT 2 - GGCTTTTGAAATGCACGCAGAG

NC_000962.3 3007963 3007982 TBseq_1.0_1723_LEFT 1 + TGCCGACCTGACTCATGGT

NC_000962.3 3009994 3010014 TBseq_1.0_1723_RIGHT 1 - CCCGACTCGAAGTCCTCACT

NC_000962.3 3009742 3009760 TBseq_1.0_1724_LEFT 2 + ACCGAAACCGCCAAGTGG

NC_000962.3 3011787 3011811 TBseq_1.0_1724_RIGHT 2 - TTGCACGAAACTTCCATAACGTAG

NC_000962.3 3011397 3011420 TBseq_1.0_1725_LEFT 1 + GTCACCGCTGAATTTCGTAATGC

NC_000962.3 3013454 3013474 TBseq_1.0_1725_RIGHT 1 - AGACCAGGAGGAGCTTCAGG

NC_000962.3 3013178 3013201 TBseq_1.0_1726_LEFT 2 + CATCCTTGATCGAAACTTTGGCC

NC_000962.3 3015226 3015245 TBseq_1.0_1726_RIGHT 2 - CGTGCTTGTCGAAAGCGGT

NC_000962.3 3014966 3014987 TBseq_1.0_1727_LEFT 1 + CCTATCCCCTCACATGCCTCA

NC_000962.3 3016908 3016927 TBseq_1.0_1727_RIGHT 1 - CGATGCCGAAGCCATGACG

NC_000962.3 3016388 3016410 TBseq_1.0_1728_LEFT 2 + ATGCCGTAGACGAAATTCACCG

NC_000962.3 3018411 3018431 TBseq_1.0_1728_RIGHT 2 - GGAGGCCTTGTCCTTTTCGG

NC_000962.3 3017297 3017317 TBseq_1.0_1729_LEFT 1 + CACATTCGGAACCGGGATCG

NC_000962.3 3019337 3019356 TBseq_1.0_1729_RIGHT 1 - ATTCGATCTGGCGGATGCG

NC_000962.3 3018823 3018842 TBseq_1.0_1730_LEFT 2 + CGCGGTGGAGAAGTTCGAC

NC_000962.3 3020782 3020802 TBseq_1.0_1730_RIGHT 2 - GTCGAAAGCGCGCTCTTTTC

NC_000962.3 3020636 3020656 TBseq_1.0_1731_LEFT 1 + CCAAAAGCTGGGACGACTCG

NC_000962.3 3022534 3022553 TBseq_1.0_1731_RIGHT 1 - CAGATAGACGCGCACGAGG

NC_000962.3 3022389 3022410 TBseq_1.0_1732_LEFT 2 + GCTGGGTGGGAACTCAAAGTC

NC_000962.3 3024315 3024335 TBseq_1.0_1732_RIGHT 2 - CAACGGGCTGAACAACTCGG

NC_000962.3 3024135 3024154 TBseq_1.0_1733_LEFT 1 + CCAACGCACGAGTAACCGT

NC_000962.3 3026104 3026124 TBseq_1.0_1733_RIGHT 1 - GTCGCGCAGGTGGAATTTCA

NC_000962.3 3025845 3025866 TBseq_1.0_1734_LEFT 2 + GTCACCGGCGACTACATCATC

NC_000962.3 3027881 3027901 TBseq_1.0_1734_RIGHT 2 - ATCGTACTGGCGCTCAAGGG

NC_000962.3 3027626 3027646 TBseq_1.0_1735_LEFT 1 + CGGGTAGCGCTTCCAACCTA

NC_000962.3 3029615 3029635 TBseq_1.0_1735_RIGHT 1 - TCGGTCCAGGTCCAGAGGTA

NC_000962.3 3029241 3029261 TBseq_1.0_1736_LEFT 2 + TGATCAACGCCAGCAAGGTC

NC_000962.3 3031247 3031267 TBseq_1.0_1736_RIGHT 2 - CAGATGATCACCGGCGGTTC

NC_000962.3 3030681 3030702 TBseq_1.0_1737_LEFT 1 + CGCTGATCACCTTTTCCCTGC

NC_000962.3 3032658 3032679 TBseq_1.0_1737_RIGHT 1 - GGACCCATACCAGATGCCGAA

NC_000962.3 3032396 3032416 TBseq_1.0_1738_LEFT 2 + GGTCAGGTGTGGTTGATGCC

NC_000962.3 3034332 3034352 TBseq_1.0_1738_RIGHT 2 - GCCAAATACCTCTACGGCCC

NC_000962.3 3033454 3033476 TBseq_1.0_1739_LEFT 1 + CGGGATCCGTAGTGAATGTGTT

NC_000962.3 3035481 3035501 TBseq_1.0_1739_RIGHT 1 - AACGGGGTCATCATTCGCTT

NC_000962.3 3035233 3035250 TBseq_1.0_1740_LEFT 2 + CGTTTGCGCTCGTCACG

NC_000962.3 3037219 3037240 TBseq_1.0_1740_RIGHT 2 - TTGTTTCGCGAGTCTTACCGG

NC_000962.3 3036803 3036825 TBseq_1.0_1741_LEFT 1 + AACGTCTTTGACCCGTTTAGCA

NC_000962.3 3038811 3038831 TBseq_1.0_1741_RIGHT 1 - GGGCCTTTCGGACTTCACAC

NC_000962.3 3038664 3038681 TBseq_1.0_1742_LEFT 2 + TGCCCTCGGTCCACACT

NC_000962.3 3040656 3040673 TBseq_1.0_1742_RIGHT 2 - ATCGTGAACGCCGACGC

NC_000962.3 3040386 3040404 TBseq_1.0_1743_LEFT 1 + TCCCATCTCGCGCGTACT

NC_000962.3 3042390 3042409 TBseq_1.0_1743_RIGHT 1 - GTGACACTTCAGCGGTCCG

NC_000962.3 3041908 3041927 TBseq_1.0_1744_LEFT 2 + CTCCGTCACGACCCCTTTG

NC_000962.3 3043941 3043960 TBseq_1.0_1744_RIGHT 2 - GCATTGCGAGCCGTGAAGA

NC_000962.3 3043361 3043381 TBseq_1.0_1745_LEFT 1 + GACGCACGCAAGATCAAAGC

NC_000962.3 3045426 3045447 TBseq_1.0_1745_RIGHT 1 - ACCTTCCAATACTCCAAGCGG

NC_000962.3 3044834 3044855 TBseq_1.0_1746_LEFT 2 + GGCAGGATAAACGACCCCAAC

NC_000962.3 3046908 3046928 TBseq_1.0_1746_RIGHT 2 - CGCTAACGTCATTGCGTAGC

NC_000962.3 3046410 3046430 TBseq_1.0_1747_LEFT 1 + GACGTTCATCTGACAGCCGT

NC_000962.3 3048410 3048430 TBseq_1.0_1747_RIGHT 1 - CCAGCCCGAGAACATAGACC

NC_000962.3 3048149 3048168 TBseq_1.0_1748_LEFT 2 + GGACTTCAGCGGCCTGTTG

NC_000962.3 3050073 3050095 TBseq_1.0_1748_RIGHT 2 - TGGGAGAAGACGATTCCGAATT

NC_000962.3 3049822 3049842 TBseq_1.0_1749_LEFT 1 + ACGTTTGCTCTGGATCCGTC

NC_000962.3 3051826 3051844 TBseq_1.0_1749_RIGHT 1 - TTGTGCGCCGACTTCGAC

NC_000962.3 3050871 3050890 TBseq_1.0_1750_LEFT 2 + TATTCAGCGCGCCGGTCAT

NC_000962.3 3052859 3052879 TBseq_1.0_1750_RIGHT 2 - CAGCCGAACTGCTAGGGATC

NC_000962.3 3055856 3055874 TBseq_1.0_1751_LEFT 1 + ACCGAAACGCCGACCTTC

NC_000962.3 3057877 3057898 TBseq_1.0_1751_RIGHT 1 - CGTCAATTGGAGATGCGACTC

NC_000962.3 3057703 3057724 TBseq_1.0_1752_LEFT 2 + CTTGAGGTCTTCGACGCTCTG

NC_000962.3 3059709 3059728 TBseq_1.0_1752_RIGHT 2 - TCCAGGAATTCGGCGACCC

NC_000962.3 3059343 3059365 TBseq_1.0_1753_LEFT 1 + ACACCTATGCCGGAAAGATCTT

NC_000962.3 3061319 3061338 TBseq_1.0_1753_RIGHT 1 - TCGAGGGTCCCTACACGCT

NC_000962.3 3060277 3060296 TBseq_1.0_1754_LEFT 2 + GCCCATGCCGATCAGCTTT

NC_000962.3 3062236 3062256 TBseq_1.0_1754_RIGHT 2 - CCAGAAAGAGGTCGAGGCCC

NC_000962.3 3061963 3061981 TBseq_1.0_1755_LEFT 1 + CAACAAGGCGTCGACCCA

NC_000962.3 3063939 3063959 TBseq_1.0_1755_RIGHT 1 - GGTAGGCACGGGTGTCCAAT

NC_000962.3 3063739 3063759 TBseq_1.0_1756_LEFT 2 + GCGGACTTGTTCTTGCCCAG

NC_000962.3 3065676 3065694 TBseq_1.0_1756_RIGHT 2 - GCACACCGGCGACATCAA

NC_000962.3 3065451 3065470 TBseq_1.0_1757_LEFT 1 + TGCTGTACCCGATCCACGT

NC_000962.3 3067472 3067491 TBseq_1.0_1757_RIGHT 1 - CCAAGTTCGCCGACCAACC

NC_000962.3 3066474 3066493 TBseq_1.0_1758_LEFT 2 + AGGTGGGCAATCACGCTGA

NC_000962.3 3068548 3068568 TBseq_1.0_1758_RIGHT 2 - CCGATCGACGAGAAGATGGC

NC_000962.3 3068311 3068331 TBseq_1.0_1759_LEFT 1 + ACCATCGTACGCAGCTGTGT

NC_000962.3 3070211 3070228 TBseq_1.0_1759_RIGHT 1 - GCATCGCCGCCATCACA

NC_000962.3 3070019 3070040 TBseq_1.0_1760_LEFT 2 + GCGTGTCTTTGAGCTCCTTCA

NC_000962.3 3071936 3071955 TBseq_1.0_1760_RIGHT 2 - AGCCGTGTGTGTTGTTCCC

NC_000962.3 3070844 3070869 TBseq_1.0_1761_LEFT 1 + TGCATATAACGGATGTATCTCAGGC

NC_000962.3 3072878 3072900 TBseq_1.0_1761_RIGHT 1 - AAACGCCGGCACTTGTATTACG

NC_000962.3 3072605 3072629 TBseq_1.0_1762_LEFT 2 + AGCCTTACCTTTTCGACTTTCTCG

NC_000962.3 3074683 3074702 TBseq_1.0_1762_RIGHT 2 - CGGGAGTGAACAGACGCAC

NC_000962.3 3074345 3074367 TBseq_1.0_1763_LEFT 1 + GAAACCGGCCGACAAATCATAG

NC_000962.3 3076363 3076384 TBseq_1.0_1763_RIGHT 1 - CGAAGGCAAGCCAAATGACCT

NC_000962.3 3074800 3074821 TBseq_1.0_1764_LEFT 2 + TACGTGGTTCTGCTTCCCGAC

NC_000962.3 3076699 3076718 TBseq_1.0_1764_RIGHT 2 - GGTTGGTTACGAGGGTGCC

NC_000962.3 3079157 3079177 TBseq_1.0_1765_LEFT 1 + GGCACCGGCAGTCTTAACAC

NC_000962.3 3081144 3081164 TBseq_1.0_1765_RIGHT 1 - GACGGCCCTACAGCTAGAGG

NC_000962.3 3080830 3080852 TBseq_1.0_1766_LEFT 2 + CCACTGATGTACCCCAGATTGG

NC_000962.3 3082787 3082807 TBseq_1.0_1766_RIGHT 2 - CCTAGATAGGTCGTGGGCGC

NC_000962.3 3082447 3082467 TBseq_1.0_1767_LEFT 1 + CACGGTAGTTCTGTCGCAGC

NC_000962.3 3084479 3084497 TBseq_1.0_1767_RIGHT 1 - AGCGTCGCACTGATCGGT

NC_000962.3 3084342 3084364 TBseq_1.0_1768_LEFT 2 + CAATCCTCCCAATAACGCTCGT

NC_000962.3 3086279 3086300 TBseq_1.0_1768_RIGHT 2 - TCGAAAACCTCAATGCCGACG

NC_000962.3 3086096 3086118 TBseq_1.0_1769_LEFT 1 + CAGATCAGTGATCAGCCCGTAT

NC_000962.3 3088032 3088052 TBseq_1.0_1769_RIGHT 1 - GCACGGAACAAACCTTTGGG

NC_000962.3 3087746 3087764 TBseq_1.0_1770_LEFT 2 + ACCTACGCGCTGACCAAC

NC_000962.3 3089799 3089820 TBseq_1.0_1770_RIGHT 2 - GGGCTCAACTGCAATCGTTTC

NC_000962.3 3088773 3088793 TBseq_1.0_1771_LEFT 1 + GCACAAACAGATTGGCTCCG

NC_000962.3 3090700 3090720 TBseq_1.0_1771_RIGHT 1 - CGCGCAGATCTCCATCGAAG

NC_000962.3 3090423 3090443 TBseq_1.0_1772_LEFT 2 + TCGATGTCGGCGATCTCCAC

NC_000962.3 3092398 3092421 TBseq_1.0_1772_RIGHT 2 - CAAGAACCCCAAAGAACACTTCG

NC_000962.3 3091866 3091884 TBseq_1.0_1773_LEFT 1 + CACAGCGCGGCGATAAAC

NC_000962.3 3093831 3093851 TBseq_1.0_1773_RIGHT 1 - GTCAACCCGCTGCTAGACTG

NC_000962.3 3093581 3093599 TBseq_1.0_1774_LEFT 2 + AGTCCTCGCCGCGAATGA

NC_000962.3 3095550 3095570 TBseq_1.0_1774_RIGHT 2 - TGGACCAGGAGCGAGTTTTG

NC_000962.3 3095031 3095048 TBseq_1.0_1775_LEFT 1 + AGAATTGGCGCGCGCAT

NC_000962.3 3096953 3096972 TBseq_1.0_1775_RIGHT 1 - TCGTCAGCCCTCACAACCA

NC_000962.3 3096756 3096775 TBseq_1.0_1776_LEFT 2 + CAACGCCGTGCTGGATTTG

NC_000962.3 3098719 3098737 TBseq_1.0_1776_RIGHT 2 - AAGCGGGAAAAGCGGGAC

NC_000962.3 3098303 3098323 TBseq_1.0_1777_LEFT 1 + GCATCGAGCACAAAGACCAG

NC_000962.3 3100349 3100368 TBseq_1.0_1777_RIGHT 1 - TCGCACGCTACGAGGACAC

NC_000962.3 3100119 3100137 TBseq_1.0_1778_LEFT 2 + ATGCCCACGCCAATGACG

NC_000962.3 3102185 3102207 TBseq_1.0_1778_RIGHT 2 - CTGAGTGGTCGGTAGTTGTCAG

NC_000962.3 3101551 3101571 TBseq_1.0_1779_LEFT 1 + CCAGCCCTCAGGAATCTCGA

NC_000962.3 3103561 3103581 TBseq_1.0_1779_RIGHT 1 - GTTGCCCAATGGTGTGCTGG

NC_000962.3 3103096 3103116 TBseq_1.0_1780_LEFT 2 + CGTTCGATGCCGATCACCAA

NC_000962.3 3105018 3105037 TBseq_1.0_1780_RIGHT 2 - CGAGCAGACGCAAAAGCCC

NC_000962.3 3104585 3104605 TBseq_1.0_1781_LEFT 1 + CCCATCTCGTCGCACATGTT

NC_000962.3 3106581 3106598 TBseq_1.0_1781_RIGHT 1 - TGGCAGCGCTTACCGAC

NC_000962.3 3105958 3105978 TBseq_1.0_1782_LEFT 2 + ATGACATAAGCGTGCCCGTG

NC_000962.3 3107995 3108012 TBseq_1.0_1782_RIGHT 2 - CACGGGTCAAACGGGCT

NC_000962.3 3107693 3107715 TBseq_1.0_1783_LEFT 1 + CGCAGAATTCACCGCATCAAAG

NC_000962.3 3109737 3109754 TBseq_1.0_1783_RIGHT 1 - TGTGCACCAGCTCGACG

NC_000962.3 3109375 3109395 TBseq_1.0_1784_LEFT 2 + CGGGAATCGTTGGACTGGTT

NC_000962.3 3111316 3111333 TBseq_1.0_1784_RIGHT 2 - TGGACTGCGCGGACCTT

NC_000962.3 3110751 3110772 TBseq_1.0_1785_LEFT 1 + CCAGGGTCGTACCTAGGTAGT

NC_000962.3 3112814 3112832 TBseq_1.0_1785_RIGHT 1 - GATCGATCGGCAGCACGT

NC_000962.3 3112461 3112479 TBseq_1.0_1786_LEFT 2 + GTTCTACGCGCTCGCCAT

NC_000962.3 3114420 3114442 TBseq_1.0_1786_RIGHT 2 - GTGTCATAGCGCCAGTAGAACG

NC_000962.3 3114047 3114067 TBseq_1.0_1787_LEFT 1 + CACACCTGTTCGGATGAGGC

NC_000962.3 3115954 3115975 TBseq_1.0_1787_RIGHT 1 - CGGTGTCCCTGTCGTACTAGA

NC_000962.3 3115591 3115610 TBseq_1.0_1788_LEFT 2 + CAGTGGAGCGGCTATGTCG

NC_000962.3 3117592 3117615 TBseq_1.0_1788_RIGHT 2 - GAAGAACCTCTCTATCTTGCCCC

NC_000962.3 3117682 3117701 TBseq_1.0_1789_LEFT 1 + TCGCTGATCTGGCCGAGTT

NC_000962.3 3119670 3119692 TBseq_1.0_1789_RIGHT 1 - CATCTGTGCCTCATACAGGTCC

NC_000962.3 3119393 3119410 TBseq_1.0_1790_LEFT 2 + TGTGGCGGGCGATATGC

NC_000962.3 3121443 3121460 TBseq_1.0_1790_RIGHT 2 - CCCCGATGGTTTGCGGT

NC_000962.3 3121236 3121255 TBseq_1.0_1791_LEFT 1 + CGATGCCCTCACGGTTCAG

NC_000962.3 3123212 3123232 TBseq_1.0_1791_RIGHT 1 - CGTCATACGCCGACCAATCA

NC_000962.3 3122564 3122585 TBseq_1.0_1792_LEFT 2 + TGGCAATGATGGTCGACGAAG

NC_000962.3 3124622 3124641 TBseq_1.0_1792_RIGHT 2 - CAGCAGGCCTTGATTCGGG

NC_000962.3 3124414 3124434 TBseq_1.0_1793_LEFT 1 + GAGTTGAAGGCGTCCAACGG

NC_000962.3 3126403 3126425 TBseq_1.0_1793_RIGHT 1 - AAGACCTTTGTCACCGACCAAG

NC_000962.3 3126129 3126153 TBseq_1.0_1794_LEFT 2 + GCTGAGGAATAGCACGACAACTAT

NC_000962.3 3128170 3128189 TBseq_1.0_1794_RIGHT 2 - CGCGGACACCCTCTACTCT

NC_000962.3 3127695 3127714 TBseq_1.0_1795_LEFT 1 + TAAACGCTCCGAACCCGCT

NC_000962.3 3129677 3129696 TBseq_1.0_1795_RIGHT 1 - ACGGGGTTGCACTCTTCGA

NC_000962.3 3129425 3129447 TBseq_1.0_1796_LEFT 2 + AACCATTGGTGTAGCCGGTTAG

NC_000962.3 3131469 3131488 TBseq_1.0_1796_RIGHT 2 - CCGGCCTACATCGCCTACA

NC_000962.3 3131053 3131075 TBseq_1.0_1797_LEFT 1 + GGCTTGTAGGTAGTGCCAGATG

NC_000962.3 3132995 3133012 TBseq_1.0_1797_RIGHT 1 - CGAGTACGGCGGTTGCA

NC_000962.3 3132448 3132468 TBseq_1.0_1798_LEFT 2 + GACTCCATGAGGACGCCATG

NC_000962.3 3134423 3134442 TBseq_1.0_1798_RIGHT 2 - GCGGTACCAGCTTACGGAA

NC_000962.3 3133649 3133666 TBseq_1.0_1799_LEFT 1 + ATTCAGCCGCGTCCAGC

NC_000962.3 3135685 3135705 TBseq_1.0_1799_RIGHT 1 - GACGTTCTTGGCGACTCTCC

NC_000962.3 3136636 3136655 TBseq_1.0_1800_LEFT 2 + GTCGTGGGTGCCGATGACT

NC_000962.3 3138604 3138624 TBseq_1.0_1800_RIGHT 2 - CACCACCACCGTCTACGTCA

NC_000962.3 3137196 3137216 TBseq_1.0_1801_LEFT 1 + TCTTCGCCTTCACCTCCGTA

NC_000962.3 3139158 3139180 TBseq_1.0_1801_RIGHT 1 - TGGCTAACGTTCAGTACTCTGC

NC_000962.3 3138814 3138833 TBseq_1.0_1802_LEFT 2 + GCATGTCGGCGACTTCCAG

NC_000962.3 3140830 3140849 TBseq_1.0_1802_RIGHT 2 - CGCAACCACTTTCTGTCGC

NC_000962.3 3140619 3140639 TBseq_1.0_1803_LEFT 1 + TGCTGCAGAAACGTCAGACC

NC_000962.3 3142637 3142656 TBseq_1.0_1803_RIGHT 1 - GCGTTAGGGCTAGGCTCCT

NC_000962.3 3142226 3142249 TBseq_1.0_1804_LEFT 2 + GCAGTGTAGCTCCGTATTCTGTC

NC_000962.3 3144274 3144295 TBseq_1.0_1804_RIGHT 2 - CGATCTAACTGATTCCGGGCG

NC_000962.3 3143118 3143137 TBseq_1.0_1805_LEFT 1 + GAAGCCGGCGACCACATAC

NC_000962.3 3145073 3145093 TBseq_1.0_1805_RIGHT 1 - AAGGATCCTGGATTGGCCGG

NC_000962.3 3144851 3144871 TBseq_1.0_1806_LEFT 2 + TGCACACTGTCCGAGATCGT

NC_000962.3 3146896 3146914 TBseq_1.0_1806_RIGHT 2 - CGCGGACAAAATCGACGC

NC_000962.3 3146433 3146456 TBseq_1.0_1807_LEFT 1 + GACCACCAAAATGGCGATATCGG

NC_000962.3 3148357 3148378 TBseq_1.0_1807_RIGHT 1 - GCGTTATCCCACGGCAGATAG

NC_000962.3 3147805 3147828 TBseq_1.0_1808_LEFT 2 + AGAACTTCCTTGCTGGTTACACC

NC_000962.3 3149813 3149833 TBseq_1.0_1808_RIGHT 2 - CCCTCTCGACCTGGATACGA

NC_000962.3 3149453 3149472 TBseq_1.0_1809_LEFT 1 + GCCCCATCTCGGAAGACTG

NC_000962.3 3151453 3151470 TBseq_1.0_1809_RIGHT 1 - ACAAGGACGCGCAGGCT

NC_000962.3 3150638 3150656 TBseq_1.0_1810_LEFT 2 + GGGTTGCTCGCCTCCATT

NC_000962.3 3152565 3152586 TBseq_1.0_1810_RIGHT 2 - GCGAGTACAGCTCCTACAAGG

NC_000962.3 3152266 3152286 TBseq_1.0_1811_LEFT 1 + CTCGAGGCACCGGACAAATG

NC_000962.3 3154233 3154254 TBseq_1.0_1811_RIGHT 1 - GCTATTCACCATCTCGTCGGT

NC_000962.3 3153659 3153679 TBseq_1.0_1812_LEFT 2 + GGTCAGGCTGAACATGACGC

NC_000962.3 3155649 3155667 TBseq_1.0_1812_RIGHT 2 - CTTGACGGCGCCTGGTAT

NC_000962.3 3155163 3155182 TBseq_1.0_1813_LEFT 1 + GGATCTGGCCCGTTCGATC

NC_000962.3 3157227 3157245 TBseq_1.0_1813_RIGHT 1 - CTGTTCGACGGGCGCATT

NC_000962.3 3157055 3157075 TBseq_1.0_1814_LEFT 2 + TGAGGATGACACCGGCGATC

NC_000962.3 3159060 3159080 TBseq_1.0_1814_RIGHT 2 - ACCTGCCTCACAACCGAACT

NC_000962.3 3157952 3157974 TBseq_1.0_1815_LEFT 1 + CCACTTAGCGCTCTTGACAAAC

NC_000962.3 3159929 3159949 TBseq_1.0_1815_RIGHT 1 - GAGAAGGGCACGGCGAAATC

NC_000962.3 3159380 3159401 TBseq_1.0_1816_LEFT 2 + AACACCACATCGTCTACCGTG

NC_000962.3 3161289 3161312 TBseq_1.0_1816_RIGHT 2 - GCAGAAATCCGGGATCAAAGAGG

NC_000962.3 3160252 3160269 TBseq_1.0_1817_LEFT 1 + CGGGCGTCGCGTTTAGT

NC_000962.3 3162221 3162241 TBseq_1.0_1817_RIGHT 1 - GGGCGGATAGTGCGGATTCT

NC_000962.3 3162142 3162161 TBseq_1.0_1818_LEFT 2 + CCAGTAACCCTGTGCAGCG

NC_000962.3 3164182 3164204 TBseq_1.0_1818_RIGHT 2 - GGAATTGTGCACTGCCAATAGC

NC_000962.3 3164183 3164205 TBseq_1.0_1819_LEFT 1 + CTATTGGCAGTGCACAATTCCG

NC_000962.3 3166257 3166277 TBseq_1.0_1819_RIGHT 1 - ACGGCAGCGATCTGAGGATC

NC_000962.3 3166093 3166110 TBseq_1.0_1820_LEFT 2 + CGCGTGGGGTTTTTGCG

NC_000962.3 3168092 3168112 TBseq_1.0_1820_RIGHT 2 - TGGTTGTTCAGCAGCGACAC

NC_000962.3 3167792 3167811 TBseq_1.0_1821_LEFT 1 + CACAACTTGACCTCGGGCG

NC_000962.3 3169713 3169730 TBseq_1.0_1821_RIGHT 1 - GCTGGGCGCGGATTTTG

NC_000962.3 3169368 3169387 TBseq_1.0_1822_LEFT 2 + CCGACGGCGATGAACACAT

NC_000962.3 3171325 3171344 TBseq_1.0_1822_RIGHT 2 - GGGTGTTGCGGTACTGCTG

NC_000962.3 3171167 3171184 TBseq_1.0_1823_LEFT 1 + AACTCCCAAGCGTCGCG

NC_000962.3 3173148 3173168 TBseq_1.0_1823_RIGHT 1 - TGTCTGTAGCGCGAGTAGGC

NC_000962.3 3172672 3172689 TBseq_1.0_1824_LEFT 2 + CGATCACCAGCGCCGTT

NC_000962.3 3174720 3174740 TBseq_1.0_1824_RIGHT 2 - AACTGCTAGGTTTTCGGCGG

NC_000962.3 3174005 3174025 TBseq_1.0_1825_LEFT 1 + ACGACTAGGCATCCCGCTAG

NC_000962.3 3176002 3176022 TBseq_1.0_1825_RIGHT 1 - CCAGTTCCTGCAACACCACC

NC_000962.3 3175537 3175555 TBseq_1.0_1826_LEFT 2 + CAGCGACGCAAATGCCAG

NC_000962.3 3177522 3177542 TBseq_1.0_1826_RIGHT 2 - CCGCATACCGCCATACTAGG

NC_000962.3 3177270 3177295 TBseq_1.0_1827_LEFT 1 + CTGATGTTAAGAACTGTGACGGAGA

NC_000962.3 3179301 3179319 TBseq_1.0_1827_RIGHT 1 - TTCGCAGATGTCGGCTCC

NC_000962.3 3178818 3178837 TBseq_1.0_1828_LEFT 2 + ATGAACATGTCGACGGCGG

NC_000962.3 3180771 3180790 TBseq_1.0_1828_RIGHT 2 - AACCTCATCCTGGCTGCGA

NC_000962.3 3179323 3179343 TBseq_1.0_1829_LEFT 1 + CTTTGGTGGGAACTGACGGC

NC_000962.3 3181344 3181364 TBseq_1.0_1829_RIGHT 1 - CGTAATCGGCGAAACTGGCT

NC_000962.3 3181456 3181476 TBseq_1.0_1830_LEFT 2 + GGCGAATAACACTGCGACCC

NC_000962.3 3183517 3183537 TBseq_1.0_1830_RIGHT 2 - GGTAACCACCCGGATGAAGC

NC_000962.3 3183277 3183297 TBseq_1.0_1831_LEFT 1 + CCGTTATCGAGTCCAGCCGT

NC_000962.3 3185331 3185348 TBseq_1.0_1831_RIGHT 1 - CACGTTTGCGCCGGACT

NC_000962.3 3184536 3184555 TBseq_1.0_1832_LEFT 2 + CGATACGGTGCTGATGCCC

NC_000962.3 3186542 3186565 TBseq_1.0_1832_RIGHT 2 - GAAGTACGTCTCGGGAGTAAGTG

NC_000962.3 3186214 3186233 TBseq_1.0_1833_LEFT 1 + AGTGGTTTGGCGGTCATCG

NC_000962.3 3188247 3188266 TBseq_1.0_1833_RIGHT 1 - GCCGTTCGTCAACCGGATC

NC_000962.3 3187828 3187847 TBseq_1.0_1834_LEFT 2 + GCCTGTGCATCTTCGGCTT

NC_000962.3 3189829 3189849 TBseq_1.0_1834_RIGHT 2 - TACGCGCTGATCCGTGATGT

NC_000962.3 3189411 3189435 TBseq_1.0_1835_LEFT 1 + GTTCGTGTTTTGACCTAATGACGT

NC_000962.3 3191442 3191459 TBseq_1.0_1835_RIGHT 1 - GTTCCGCGCGTTTGGGT

NC_000962.3 3190355 3190377 TBseq_1.0_1836_LEFT 2 + ACCGATTCGAACATGGTACCGT

NC_000962.3 3192371 3192392 TBseq_1.0_1836_RIGHT 2 - GAACGCTGGTCACCACCTAAG

NC_000962.3 3192085 3192105 TBseq_1.0_1837_LEFT 1 + CGAGAGAACATGCCAGGGTT

NC_000962.3 3194166 3194185 TBseq_1.0_1837_RIGHT 1 - ACGGGGTGCAAGTCAAGTG

NC_000962.3 3193964 3193983 TBseq_1.0_1838_LEFT 2 + GGTGTGGGGCTATGAGTGC

NC_000962.3 3195926 3195949 TBseq_1.0_1838_RIGHT 2 - GATCAGAAGGTGGATCTGGATCG

NC_000962.3 3195684 3195703 TBseq_1.0_1839_LEFT 1 + CATCGGCCGAATCGACCAC

NC_000962.3 3197653 3197672 TBseq_1.0_1839_RIGHT 1 - CGGAGTATCGCAGGTTGCC

NC_000962.3 3196832 3196853 TBseq_1.0_1840_LEFT 2 + AAGCTCGGGTCCGACTAGATC

NC_000962.3 3198771 3198790 TBseq_1.0_1840_RIGHT 2 - GCTCAAGGGTGCCAGCATC

NC_000962.3 3197912 3197931 TBseq_1.0_1841_LEFT 1 + TCTTGCCCAGGGACACCAG

NC_000962.3 3199894 3199916 TBseq_1.0_1841_RIGHT 1 - TGGAATCCCAAGATGAAGCGTT

NC_000962.3 3200172 3200192 TBseq_1.0_1842_LEFT 2 + GCTGCGCCGAGTAGTTTACA

NC_000962.3 3202175 3202195 TBseq_1.0_1842_RIGHT 2 - CAGGGATGAGGGCACAGGAT

NC_000962.3 3200322 3200343 TBseq_1.0_1843_LEFT 1 + AGCGTACTGATCCTTTGCTGG

NC_000962.3 3202288 3202307 TBseq_1.0_1843_RIGHT 1 - AGCTGGCCGGGAATCAAAC

NC_000962.3 3202305 3202328 TBseq_1.0_1844_LEFT 2 + CTACCGTTCACCTGAAGGAAGTC

NC_000962.3 3204231 3204248 TBseq_1.0_1844_RIGHT 2 - CAATGCGGTCGGTCGGT

NC_000962.3 3203866 3203885 TBseq_1.0_1845_LEFT 1 + GCTCGACGATCAACCGGTC

NC_000962.3 3205877 3205894 TBseq_1.0_1845_RIGHT 1 - CTGGCCGGCGGATTTGA

NC_000962.3 3205596 3205616 TBseq_1.0_1846_LEFT 2 + GAAGCAGCGTATGACAGCCC

NC_000962.3 3207627 3207647 TBseq_1.0_1846_RIGHT 2 - AACGTCTGGAGAACACGCTG

NC_000962.3 3207024 3207044 TBseq_1.0_1847_LEFT 1 + GCACTCGTCCAGGAACAACA

NC_000962.3 3209023 3209041 TBseq_1.0_1847_RIGHT 1 - TAAGGCGTCGCTTCAGGC

NC_000962.3 3208314 3208333 TBseq_1.0_1848_LEFT 2 + TTCAGGGTTGTCATGCGGC

NC_000962.3 3210228 3210245 TBseq_1.0_1848_RIGHT 2 - AGGTGCGCGCTGACTGT

NC_000962.3 3210021 3210041 TBseq_1.0_1849_LEFT 1 + CAGATGATCTGCACCTCGCT

NC_000962.3 3212061 3212082 TBseq_1.0_1849_RIGHT 1 - TGAAACCGAGATGGAGCTCTC

NC_000962.3 3210814 3210834 TBseq_1.0_1850_LEFT 2 + CGAACAATGCCATGTCCCCG

NC_000962.3 3212749 3212769 TBseq_1.0_1850_RIGHT 2 - CAAGTCTTGCCGCCCTTGAT

NC_000962.3 3212559 3212578 TBseq_1.0_1851_LEFT 1 + CGCTGAGCACATAACCGGG

NC_000962.3 3214557 3214577 TBseq_1.0_1851_RIGHT 1 - CGCATTGCCCTCTTAAGTGC

NC_000962.3 3214244 3214267 TBseq_1.0_1852_LEFT 2 + CCGGTTCATTGGAAATGACCATC

NC_000962.3 3216319 3216336 TBseq_1.0_1852_RIGHT 2 - ATCTTCCCCGCCTGCCT

NC_000962.3 3216101 3216120 TBseq_1.0_1853_LEFT 1 + CGGGCGTGGGAACAATCAA

NC_000962.3 3218103 3218123 TBseq_1.0_1853_RIGHT 1 - ATCGACTATCGCGCGCTAGG

NC_000962.3 3217831 3217856 TBseq_1.0_1854_LEFT 2 + TCATTTTCTGGAAGCACTTGGTTTC

NC_000962.3 3219737 3219755 TBseq_1.0_1854_RIGHT 2 - TGATCACCAAGCGGGCTG

NC_000962.3 3219264 3219281 TBseq_1.0_1855_LEFT 1 + TGCTGGCGCTCATAGGC

NC_000962.3 3221289 3221309 TBseq_1.0_1855_RIGHT 1 - CGAATTTGTCCTGGGTGCCC

NC_000962.3 3220882 3220902 TBseq_1.0_1856_LEFT 2 + CAAGAAAAACAGCAGCGCGG

NC_000962.3 3222880 3222897 TBseq_1.0_1856_RIGHT 2 - CTGCAGCCACCGAAGCT

NC_000962.3 3222523 3222543 TBseq_1.0_1857_LEFT 1 + GTGGAGAAGTTGTCCAGGCG

NC_000962.3 3224587 3224606 TBseq_1.0_1857_RIGHT 1 - CGAGACGGCGATCCAACTG

NC_000962.3 3223565 3223585 TBseq_1.0_1858_LEFT 2 + AGCTAGGGCCCAAAAGTCCT

NC_000962.3 3225525 3225543 TBseq_1.0_1858_RIGHT 2 - TCACCGGTGTGGCCTTGA

NC_000962.3 3225423 3225442 TBseq_1.0_1859_LEFT 1 + AGTCCTCCAGCTTCTCCCC

NC_000962.3 3227424 3227441 TBseq_1.0_1859_RIGHT 1 - TCGGAGAGCACGACCGT

NC_000962.3 3227021 3227039 TBseq_1.0_1860_LEFT 2 + CGACAGCCCAATCCCGTT

NC_000962.3 3229053 3229071 TBseq_1.0_1860_RIGHT 2 - TTTTGCACGCCCTGTCGG

NC_000962.3 3228865 3228884 TBseq_1.0_1861_LEFT 1 + GTCGCGCGGACTAATCTCC

NC_000962.3 3230773 3230792 TBseq_1.0_1861_RIGHT 1 - GGTAGACACCATCGTGCGG

NC_000962.3 3230372 3230393 TBseq_1.0_1862_LEFT 2 + AACGGATACCACAACCTGTCG

NC_000962.3 3232412 3232430 TBseq_1.0_1862_RIGHT 2 - TGTTGATGACGCCGGGTC

NC_000962.3 3232121 3232141 TBseq_1.0_1863_LEFT 1 + ACATCAGCTGGAATGCCACA

NC_000962.3 3234131 3234151 TBseq_1.0_1863_RIGHT 1 - AGGATTGTCAGCGTGTGGGA

NC_000962.3 3233077 3233098 TBseq_1.0_1864_LEFT 2 + GTTCTGTCCGATCGTGGCATC

NC_000962.3 3235082 3235102 TBseq_1.0_1864_RIGHT 2 - GGGAACTCACCGATTCGCTG

NC_000962.3 3234261 3234281 TBseq_1.0_1865_LEFT 1 + TCGTTCTGCATGGTTACGCC

NC_000962.3 3236258 3236276 TBseq_1.0_1865_RIGHT 1 - AGTGGCGCAGGGCTTTTC

NC_000962.3 3236124 3236143 TBseq_1.0_1866_LEFT 2 + TGTTTGAGTGCGCTGACGG

NC_000962.3 3238085 3238102 TBseq_1.0_1866_RIGHT 2 - TCGATGTCGGCGCCTGA

NC_000962.3 3237920 3237937 TBseq_1.0_1867_LEFT 1 + AGCTTCTGGCACGCAGC

NC_000962.3 3239914 3239933 TBseq_1.0_1867_RIGHT 1 - GGAGTGGGCCGGTCCAAAA

NC_000962.3 3238749 3238773 TBseq_1.0_1868_LEFT 2 + CGTTGACGTTGACATACAAAGAGT

NC_000962.3 3240775 3240794 TBseq_1.0_1868_RIGHT 2 - CGAGGAAGACGAGGTCGGA

NC_000962.3 3240821 3240842 TBseq_1.0_1869_LEFT 1 + TAGGCAAACAGTTCGGTCAGG

NC_000962.3 3242786 3242810 TBseq_1.0_1869_RIGHT 1 - CGATAAAGGCATAAATCGGACACG

NC_000962.3 3242419 3242440 TBseq_1.0_1870_LEFT 2 + GAGAGTATTCCCACCCTCGCT

NC_000962.3 3244374 3244392 TBseq_1.0_1870_RIGHT 2 - CGGTGCGCAAATTCCGAG

NC_000962.3 3244195 3244218 TBseq_1.0_1871_LEFT 1 + CGGCTTATCTCCAATACACGTCC

NC_000962.3 3246155 3246177 TBseq_1.0_1871_RIGHT 1 - TATTGCTCCAACCATCGACCTG

NC_000962.3 3245825 3245843 TBseq_1.0_1872_LEFT 2 + TGTGAACGCCGTTCCTCG

NC_000962.3 3247885 3247906 TBseq_1.0_1872_RIGHT 2 - GGGGCCACTTCGATATTGACC

NC_000962.3 3246927 3246948 TBseq_1.0_1873_LEFT 1 + CACAAACTGAATGGCCGGCAA

NC_000962.3 3248886 3248906 TBseq_1.0_1873_RIGHT 1 - CCATGAGAAAGGCAGGCCAT

NC_000962.3 3248206 3248225 TBseq_1.0_1874_LEFT 2 + ATACACCAGCCTCGGGACC

NC_000962.3 3250231 3250253 TBseq_1.0_1874_RIGHT 2 - GCTCGTCACCAATTGATCGTTG

NC_000962.3 3249774 3249792 TBseq_1.0_1875_LEFT 1 + AATTCGGGCCGGCACTTG

NC_000962.3 3251802 3251821 TBseq_1.0_1875_RIGHT 1 - CAGCAATCCCCAGGCAGAG

NC_000962.3 3251597 3251617 TBseq_1.0_1876_LEFT 2 + GGACCGGAAATGCACACAGC

NC_000962.3 3253625 3253648 TBseq_1.0_1876_RIGHT 2 - GCCAATGCTCAAGTATTTCGCTG

NC_000962.3 3253389 3253410 TBseq_1.0_1877_LEFT 1 + CCCCACCATCGGAATCATCTC

NC_000962.3 3255428 3255448 TBseq_1.0_1877_RIGHT 1 - TAGCGAGTCCATGCCGAGTT

NC_000962.3 3254934 3254953 TBseq_1.0_1878_LEFT 2 + GCTGGGTTCTCGATGGCTC

NC_000962.3 3256935 3256953 TBseq_1.0_1878_RIGHT 2 - TGCGGCCTCACTTGCATG

NC_000962.3 3256748 3256772 TBseq_1.0_1879_LEFT 1 + CGAACTCGATTCACTGAGTAAGGT

NC_000962.3 3258745 3258766 TBseq_1.0_1879_RIGHT 1 - TGCGTATTTCGACCTGGCATC

NC_000962.3 3258049 3258069 TBseq_1.0_1880_LEFT 2 + TCATCTCCACCACCTACGCC

NC_000962.3 3260094 3260116 TBseq_1.0_1880_RIGHT 2 - TCACCTCGTGAACGTTTAGTGC

NC_000962.3 3259850 3259870 TBseq_1.0_1881_LEFT 1 + AGATATCGAACCGGACGGCA

NC_000962.3 3261753 3261774 TBseq_1.0_1881_RIGHT 1 - GGGGAACGATTGGAACCACTG

NC_000962.3 3261252 3261272 TBseq_1.0_1882_LEFT 2 + GTGATCACCGGCGACATCAC

NC_000962.3 3263262 3263280 TBseq_1.0_1882_RIGHT 2 - CGGTGCCGTGTGTTTCGA

NC_000962.3 3262692 3262712 TBseq_1.0_1883_LEFT 1 + CTCGAACCGGCGTGATGATG

NC_000962.3 3264708 3264731 TBseq_1.0_1883_RIGHT 1 - GCTGATTTCGATGAAGGTGTTGT

NC_000962.3 3264662 3264680 TBseq_1.0_1884_LEFT 2 + AACCCGGTGCGATTCCAC

NC_000962.3 3266679 3266696 TBseq_1.0_1884_RIGHT 2 - GGCCAGGACGTTGCACT

NC_000962.3 3265923 3265941 TBseq_1.0_1885_LEFT 1 + AGACGACGCGGGGAAGTT

NC_000962.3 3267904 3267923 TBseq_1.0_1885_RIGHT 1 - GACGCACATACGCCGGATC

NC_000962.3 3267634 3267654 TBseq_1.0_1886_LEFT 2 + CGCTGACACTATTCGCGACC

NC_000962.3 3269680 3269697 TBseq_1.0_1886_RIGHT 2 - AATACGCCGGCCAGGGT

NC_000962.3 3268866 3268889 TBseq_1.0_1887_LEFT 1 + CCTAAAGAACAAGGCACTACCCG

NC_000962.3 3270898 3270919 TBseq_1.0_1887_RIGHT 1 - GGTCAGCACCGCTCGAATATC

NC_000962.3 3270047 3270067 TBseq_1.0_1888_LEFT 2 + TCACCGGTAGCTGGATGTCC

NC_000962.3 3271996 3272017 TBseq_1.0_1888_RIGHT 2 - CCAATCGAGATGCAGTGAGCC

NC_000962.3 3271800 3271819 TBseq_1.0_1889_LEFT 1 + CTACGTTTACGCCCCGACC

NC_000962.3 3273726 3273746 TBseq_1.0_1889_RIGHT 1 - CCCCGATCGCGATAACCAGT

NC_000962.3 3273396 3273416 TBseq_1.0_1890_LEFT 2 + TATCCCGTTCGCCATACCGT

NC_000962.3 3275357 3275376 TBseq_1.0_1890_RIGHT 2 - TAGCGGGAGAACAGCTCGT

NC_000962.3 3275081 3275101 TBseq_1.0_1891_LEFT 1 + CCCTCTTGGAAACCCACCCA

NC_000962.3 3277075 3277095 TBseq_1.0_1891_RIGHT 1 - CCAAGGTGTTCGGATCCTGG

NC_000962.3 3276701 3276722 TBseq_1.0_1892_LEFT 2 + GTGGATGCGAACATTTCACCC

NC_000962.3 3278706 3278726 TBseq_1.0_1892_RIGHT 2 - GCACGACTCGGGAGAAAGAG

NC_000962.3 3277805 3277824 TBseq_1.0_1893_LEFT 1 + CCGGTCAGCGAGTTCAGCA

NC_000962.3 3279730 3279751 TBseq_1.0_1893_RIGHT 1 - GTCAACTTCACCGTGGAGACC

NC_000962.3 3278929 3278949 TBseq_1.0_1894_LEFT 2 + GAGGCCAGCTTGATCGTTGG

NC_000962.3 3280930 3280950 TBseq_1.0_1894_RIGHT 2 - GAATCGACAAAGTGCAGCCG

NC_000962.3 3281090 3281109 TBseq_1.0_1895_LEFT 1 + GACCCTTGCCCGGAGAAGA

NC_000962.3 3283080 3283101 TBseq_1.0_1895_RIGHT 1 - TGCAACGGATGTAGTGCTTCG

NC_000962.3 3282858 3282878 TBseq_1.0_1896_LEFT 2 + GTTACACGACGCCAAGACCC

NC_000962.3 3284916 3284933 TBseq_1.0_1896_RIGHT 2 - AGCCGACGTGACTTCGC

NC_000962.3 3284689 3284712 TBseq_1.0_1897_LEFT 1 + TGGCAAAATGTTCATCATCGGTC

NC_000962.3 3286686 3286707 TBseq_1.0_1897_RIGHT 1 - GGGATCGAGGTATTGCTGTGC

NC_000962.3 3286270 3286291 TBseq_1.0_1898_LEFT 2 + TCTGCATGCTACCCATCATCG

NC_000962.3 3288253 3288273 TBseq_1.0_1898_RIGHT 2 - GACAATTTTCGACAGCGCGG

NC_000962.3 3287919 3287936 TBseq_1.0_1899_LEFT 1 + TTGGCCGGGTGAGTGGT

NC_000962.3 3289972 3289990 TBseq_1.0_1899_RIGHT 1 - TGTCGCGTTTGAGGCCAC

NC_000962.3 3289660 3289678 TBseq_1.0_1900_LEFT 2 + TACGATGACGCGCTGGGA

NC_000962.3 3291626 3291650 TBseq_1.0_1900_RIGHT 2 - ACCTACCGTGATTTTCGATCATCC

NC_000962.3 3291330 3291354 TBseq_1.0_1901_LEFT 1 + CTCACTACCTCGATTGTCAAATGG

NC_000962.3 3293341 3293361 TBseq_1.0_1901_RIGHT 1 - ATCAGCAACTGGTTACCCGG

NC_000962.3 3292101 3292120 TBseq_1.0_1902_LEFT 2 + TGCTCAAATGCGCGGTCAT

NC_000962.3 3294063 3294082 TBseq_1.0_1902_RIGHT 2 - TCTTGGGCGGACTTTTGCG

NC_000962.3 3293993 3294011 TBseq_1.0_1903_LEFT 1 + ACCGAGGATGCTTGGCCA

NC_000962.3 3296023 3296041 TBseq_1.0_1903_RIGHT 1 - TTGCCCGTGTTTGCGCAG

NC_000962.3 3295828 3295848 TBseq_1.0_1904_LEFT 2 + AACGGAGTGACCCATGACGA

NC_000962.3 3297827 3297851 TBseq_1.0_1904_RIGHT 2 - GAACTTGGGAAGTGATAGAGGAGC

NC_000962.3 3297454 3297474 TBseq_1.0_1905_LEFT 1 + TCACTAGGAGCGATGCCGAA

NC_000962.3 3299382 3299405 TBseq_1.0_1905_RIGHT 1 - CTCACTCCTGAAGATACTGGGCT

NC_000962.3 3299175 3299199 TBseq_1.0_1906_LEFT 2 + CGGAAGGAATCAGGAGAACATGAG

NC_000962.3 3301114 3301133 TBseq_1.0_1906_RIGHT 2 - CTGGGTGCAGGGAGACAAC

NC_000962.3 3300491 3300516 TBseq_1.0_1907_LEFT 1 + TGCCATTAGCTGCTATCAGTATTCG

NC_000962.3 3302429 3302453 TBseq_1.0_1907_RIGHT 1 - GAAAACCAACTCGTCATTTCATGC

NC_000962.3 3301816 3301840 TBseq_1.0_1908_LEFT 2 + ACCGGATGTGTACTGTAGATATGC

NC_000962.3 3303741 3303764 TBseq_1.0_1908_RIGHT 2 - GGGAGCTTATCTCACGTGAATCG

NC_000962.3 3303434 3303458 TBseq_1.0_1909_LEFT 1 + TCATCTTCACAACGACAGATTCCA

NC_000962.3 3305431 3305448 TBseq_1.0_1909_RIGHT 1 - GCGTTCGGGCCCAACAT

NC_000962.3 3304571 3304591 TBseq_1.0_1910_LEFT 2 + GGCCTATGAGGAAGATCCGC

NC_000962.3 3306617 3306638 TBseq_1.0_1910_RIGHT 2 - CAGGAGTTTGATTCTCGGCGG

NC_000962.3 3306198 3306219 TBseq_1.0_1911_LEFT 1 + GTATTTCGACCGACTACCCCG

NC_000962.3 3308151 3308170 TBseq_1.0_1911_RIGHT 1 - TACCCAGACCGCTGCATCC

NC_000962.3 3307602 3307623 TBseq_1.0_1912_LEFT 2 + AGAAGTAGCAGTACCGGTCGT

NC_000962.3 3309540 3309563 TBseq_1.0_1912_RIGHT 2 - AGGTGGGGATGATGATCGAAAAC

NC_000962.3 3309223 3309244 TBseq_1.0_1913_LEFT 1 + TGCAACTCGAACTGTCCTTCC

NC_000962.3 3311129 3311147 TBseq_1.0_1913_RIGHT 1 - TTTTGAACGCCCTGGCCG

NC_000962.3 3310614 3310634 TBseq_1.0_1914_LEFT 2 + AGTCGTTGCGGTACTTCAGC

NC_000962.3 3312653 3312678 TBseq_1.0_1914_RIGHT 2 - CCACTTATATGGGGATCAATTGCTG

NC_000962.3 3311645 3311664 TBseq_1.0_1915_LEFT 1 + GGATCTTCCTGTCAGCCGC

NC_000962.3 3313546 3313568 TBseq_1.0_1915_RIGHT 1 - CGTAGTTTCGTTTGAGGGTGCT

NC_000962.3 3313116 3313136 TBseq_1.0_1916_LEFT 2 + ACTGCCAAAGGTAGCCAGCT

NC_000962.3 3315129 3315149 TBseq_1.0_1916_RIGHT 2 - CCCCACTCTTGCCGAGGTTT

NC_000962.3 3313955 3313974 TBseq_1.0_1917_LEFT 1 + CTCAGTTCGCAGCAGCACG

NC_000962.3 3315951 3315970 TBseq_1.0_1917_RIGHT 1 - CGACCAGGCCGGATGATTG

NC_000962.3 3316279 3316303 TBseq_1.0_1918_LEFT 2 + GCAGAAATATCGTGGAACTACACC

NC_000962.3 3318269 3318289 TBseq_1.0_1918_RIGHT 2 - CGACTAGCTGGGGTCAAAGC

NC_000962.3 3318083 3318106 TBseq_1.0_1919_LEFT 1 + CGACAGAGAGGTACCCAATGTTC

NC_000962.3 3320123 3320144 TBseq_1.0_1919_RIGHT 1 - AGGAATTCAATGAGCACCGGG

NC_000962.3 3319777 3319797 TBseq_1.0_1920_LEFT 2 + TCGATGGTGGCGATGGTTTG

NC_000962.3 3321706 3321727 TBseq_1.0_1920_RIGHT 2 - CACGTCCTTCATTGATGAGCG

NC_000962.3 3321518 3321540 TBseq_1.0_1921_LEFT 1 + CCAACTTGACTAGTCGCTGCTT

NC_000962.3 3323450 3323470 TBseq_1.0_1921_RIGHT 1 - CTTTCCCAACCCGCTTCTCG

NC_000962.3 3323155 3323175 TBseq_1.0_1922_LEFT 2 + CGCCATTGATAGAGCAGCCG

NC_000962.3 3325173 3325193 TBseq_1.0_1922_RIGHT 2 - ATCGACTACCGGTTGGCTCC

NC_000962.3 3324941 3324963 TBseq_1.0_1923_LEFT 1 + GGTGAAATCGGTCCTGGGATAC

NC_000962.3 3326871 3326892 TBseq_1.0_1923_RIGHT 1 - GCCCAATGCATCCATGTGTTC

NC_000962.3 3326628 3326647 TBseq_1.0_1924_LEFT 2 + CAGGACGAACTGCGCAAAG

NC_000962.3 3328617 3328636 TBseq_1.0_1924_RIGHT 2 - AGATCAGTTGCGCGCCAAG

NC_000962.3 3329537 3329557 TBseq_1.0_1925_LEFT 1 + GGATGAGAAACGCCGGATGC

NC_000962.3 3331559 3331576 TBseq_1.0_1925_RIGHT 1 - TTGCGGGACTGGGCACA

NC_000962.3 3330838 3330862 TBseq_1.0_1926_LEFT 2 + CATCACATACCGCCAACAGATACA

NC_000962.3 3332834 3332854 TBseq_1.0_1926_RIGHT 2 - AGGAGTGGACTGGATACGCG

NC_000962.3 3332588 3332606 TBseq_1.0_1927_LEFT 1 + GGAAAGTGAAGGCGCGCA

NC_000962.3 3334547 3334565 TBseq_1.0_1927_RIGHT 1 - TGTGCGGGTGCTTGTCCA

NC_000962.3 3333574 3333593 TBseq_1.0_1928_LEFT 2 + AGTCCAGCCGGAAGTGACT

NC_000962.3 3335485 3335505 TBseq_1.0_1928_RIGHT 2 - CAGATGCCAGTCGACAAGGT

NC_000962.3 3335007 3335027 TBseq_1.0_1929_LEFT 1 + CCTGGATGTCGGCTTTCAGG

NC_000962.3 3336907 3336927 TBseq_1.0_1929_RIGHT 1 - ATCAACACGATGCCGGGATT

NC_000962.3 3335931 3335953 TBseq_1.0_1930_LEFT 2 + GTATGAGAGCAAAGAGGTGGCC

NC_000962.3 3337950 3337970 TBseq_1.0_1930_RIGHT 2 - AAAACTGCAGCTCGTGGGAG

NC_000962.3 3337671 3337692 TBseq_1.0_1931_LEFT 1 + GGTGCCCGATCCTGATTTGAC

NC_000962.3 3339642 3339661 TBseq_1.0_1931_RIGHT 1 - AGGCTCCTGTCAGCTCGAC

NC_000962.3 3339293 3339313 TBseq_1.0_1932_LEFT 2 + CACTGTTATCACCCCCGACG

NC_000962.3 3341213 3341234 TBseq_1.0_1932_RIGHT 2 - GTGCCTTGATCTCGACCAGTG

NC_000962.3 3340958 3340979 TBseq_1.0_1933_LEFT 1 + CATTCGTCCCAGCTACCCATC

NC_000962.3 3342949 3342968 TBseq_1.0_1933_RIGHT 1 - ATCGGGAATGACCTTGCCC

NC_000962.3 3342561 3342581 TBseq_1.0_1934_LEFT 2 + AGCTTGACTACGCCCAGGAT

NC_000962.3 3344588 3344607 TBseq_1.0_1934_RIGHT 2 - CTGGTATTGGCGTGCCGTT

NC_000962.3 3344360 3344381 TBseq_1.0_1935_LEFT 1 + CGGAATCCGTAGTCCATGAGC

NC_000962.3 3346430 3346452 TBseq_1.0_1935_RIGHT 1 - GCGATATACCTGCACGCTTTCG

NC_000962.3 3345959 3345983 TBseq_1.0_1936_LEFT 2 + ACCAGATGCAGATCGATGTAGATC

NC_000962.3 3347900 3347922 TBseq_1.0_1936_RIGHT 2 - CGCGGTGCCTAGTTTGAAAATG

NC_000962.3 3347648 3347668 TBseq_1.0_1937_LEFT 1 + CGGGCGTGTAGGTCTTCGAT

NC_000962.3 3349650 3349670 TBseq_1.0_1937_RIGHT 1 - ACGACGCGCTGATGAAGATC

NC_000962.3 3348359 3348381 TBseq_1.0_1938_LEFT 2 + CAGATGATGAACAAGCGGGTCG

NC_000962.3 3350328 3350347 TBseq_1.0_1938_RIGHT 2 - GCGACACCGTCTCGATCAC

NC_000962.3 3350103 3350123 TBseq_1.0_1939_LEFT 1 + ATAGCTTTCCTCGCTGTCGC

NC_000962.3 3352083 3352102 TBseq_1.0_1939_RIGHT 1 - CATGCGTGACCCGACATGG

NC_000962.3 3351383 3351401 TBseq_1.0_1940_LEFT 2 + TGGAAAATGCGCGCGGAA

NC_000962.3 3353359 3353382 TBseq_1.0_1940_RIGHT 2 - CGTCCAGAAAACCAGCTATGACC

NC_000962.3 3352458 3352475 TBseq_1.0_1941_LEFT 1 + TAGAGCGCGGCGGCAAT

NC_000962.3 3354419 3354439 TBseq_1.0_1941_RIGHT 1 - GGACTGATCGACAAGGAGGC

NC_000962.3 3354602 3354619 TBseq_1.0_1942_LEFT 2 + ACCAACTGCCCGATGCG

NC_000962.3 3356534 3356555 TBseq_1.0_1942_RIGHT 2 - GATCGCAAGCTTCGTTATGGC

NC_000962.3 3355768 3355786 TBseq_1.0_1943_LEFT 1 + ATCTACGCGCGCATGGTG

NC_000962.3 3357785 3357806 TBseq_1.0_1943_RIGHT 1 - TGTTGTTGTCTCCGAGCTTGC

NC_000962.3 3357609 3357629 TBseq_1.0_1944_LEFT 2 + CGCCAAACATGCTGGGAGAA

NC_000962.3 3359610 3359629 TBseq_1.0_1944_RIGHT 2 - GACCTGATGAGCTGGGTGG

NC_000962.3 3359401 3359420 TBseq_1.0_1945_LEFT 1 + GTGACGAGCACCCTCATCG

NC_000962.3 3361384 3361403 TBseq_1.0_1945_RIGHT 1 - CGACCTGGCCACTCATTCG

NC_000962.3 3360896 3360919 TBseq_1.0_1946_LEFT 2 + CTCGACGATCTTGATGACGTTGA

NC_000962.3 3362956 3362976 TBseq_1.0_1946_RIGHT 2 - CAACCAAGCCACACTCACCG

NC_000962.3 3362778 3362796 TBseq_1.0_1947_LEFT 1 + GGTCATACACCGGCAGCA

NC_000962.3 3364760 3364778 TBseq_1.0_1947_RIGHT 1 - AGCCGCTCGATACCAGCA

NC_000962.3 3364509 3364532 TBseq_1.0_1948_LEFT 2 + TGTGAGTCATTCGAACTGGTCAC

NC_000962.3 3366533 3366553 TBseq_1.0_1948_RIGHT 2 - TGATTCCGTGCCCAGATCGT

NC_000962.3 3365324 3365344 TBseq_1.0_1949_LEFT 1 + CCGGTAAGGTCCTGCGTATC

NC_000962.3 3367276 3367297 TBseq_1.0_1949_RIGHT 1 - CGAACTCGTTCTAGAGGCCTG

NC_000962.3 3366660 3366684 TBseq_1.0_1950_LEFT 2 + TGTCATAGGCATACTCGAATGTGG

NC_000962.3 3368602 3368621 TBseq_1.0_1950_RIGHT 2 - AGGTGTGTCCGGTGTGTCT

NC_000962.3 3368400 3368419 TBseq_1.0_1951_LEFT 1 + CAAAGGCGCGTCCAGGTAG

NC_000962.3 3370324 3370343 TBseq_1.0_1951_RIGHT 1 - CAAGCGGCGCATCATGATC

NC_000962.3 3369676 3369695 TBseq_1.0_1952_LEFT 2 + GGTCATTGCGGTCGTCGTT

NC_000962.3 3371720 3371738 TBseq_1.0_1952_RIGHT 2 - TTGCGGTCGTGTCCCAGA

NC_000962.3 3373708 3373728 TBseq_1.0_1953_LEFT 1 + CACCTCGTCGACTTTGACCA

NC_000962.3 3375743 3375763 TBseq_1.0_1953_RIGHT 1 - CACCATCTGCCACCCTTCTG

NC_000962.3 3375000 3375019 TBseq_1.0_1954_LEFT 2 + GCAAATCCGGACTGCAGCT

NC_000962.3 3376917 3376937 TBseq_1.0_1954_RIGHT 2 - GATCTGACGGCCGTGAACTC

NC_000962.3 3376561 3376582 TBseq_1.0_1955_LEFT 1 + CACCCTAATCGTTCCGCAGTC

NC_000962.3 3378583 3378602 TBseq_1.0_1955_RIGHT 1 - GAGGAGCTTGGTGTGTGGC

NC_000962.3 3381703 3381722 TBseq_1.0_1956_LEFT 2 + CGGGGAGTTTGTCGGTCAG

NC_000962.3 3383659 3383682 TBseq_1.0_1956_RIGHT 2 - TGTTTGGGATTTCGCAGAGAAGT

NC_000962.3 3383226 3383244 TBseq_1.0_1957_LEFT 1 + ACACCATCCGCGTCAACC

NC_000962.3 3385162 3385179 TBseq_1.0_1957_RIGHT 1 - ACGTGCCGGCGACCTAA

NC_000962.3 3384824 3384849 TBseq_1.0_1958_LEFT 2 + CAATAGATACCTTTGACAGCCAGGT

NC_000962.3 3386874 3386894 TBseq_1.0_1958_RIGHT 2 - CCAGTGACAAACCGCATGGC

NC_000962.3 3386408 3386428 TBseq_1.0_1959_LEFT 1 + CGTCTCCCCCGATTGGTACT

NC_000962.3 3388397 3388417 TBseq_1.0_1959_RIGHT 1 - CGCAAAGTGTCGATCGAGGG

NC_000962.3 3387653 3387674 TBseq_1.0_1960_LEFT 2 + ACCCACTCCACCTTCTCTCAC

NC_000962.3 3389682 3389702 TBseq_1.0_1960_RIGHT 2 - CACATGGCCACATCGACGAA

NC_000962.3 3389317 3389337 TBseq_1.0_1961_LEFT 1 + GATATGGCGCCGACCTGATC

NC_000962.3 3391260 3391280 TBseq_1.0_1961_RIGHT 1 - GCGCAGGATCTGATCAGCAA

NC_000962.3 3390554 3390573 TBseq_1.0_1962_LEFT 2 + ATGGAGGTCGACTACGCCA

NC_000962.3 3392443 3392460 TBseq_1.0_1962_RIGHT 2 - TGCCACCAGCCCAAACG

NC_000962.3 3391819 3391841 TBseq_1.0_1963_LEFT 1 + GCTTGAAGAAACTTGGCACCGA

NC_000962.3 3393845 3393867 TBseq_1.0_1963_RIGHT 1 - ACAATCACCGAGATGCCATACG

NC_000962.3 3393603 3393629 TBseq_1.0_1964_LEFT 2 + GAATTGCGAAATGAAGATCAAGGACC

NC_000962.3 3395540 3395560 TBseq_1.0_1964_RIGHT 2 - ACGTAGAGGTTGTCGAAGCC

NC_000962.3 3395242 3395261 TBseq_1.0_1965_LEFT 1 + TGCCGACTCGTGGGTAGAA

NC_000962.3 3397136 3397157 TBseq_1.0_1965_RIGHT 1 - CGAGGACGATTAGACGATGCG

NC_000962.3 3396713 3396731 TBseq_1.0_1966_LEFT 2 + AAGGTGATGGGCTGCGAG

NC_000962.3 3398681 3398700 TBseq_1.0_1966_RIGHT 2 - CACACATTCACGCCCCAGG

NC_000962.3 3397476 3397498 TBseq_1.0_1967_LEFT 1 + ACAGGTAAGCGATTTGGGGATC

NC_000962.3 3399389 3399414 TBseq_1.0_1967_RIGHT 1 - GCCTATATGACGAGGAGTTCAAACA

NC_000962.3 3399380 3399400 TBseq_1.0_1968_LEFT 2 + CGGCGGGGATGTTTGAACTC

NC_000962.3 3401431 3401452 TBseq_1.0_1968_RIGHT 2 - GCACATACGGAACACTGTCGG

NC_000962.3 3401230 3401248 TBseq_1.0_1969_LEFT 1 + TGAAGCCGGGCGGAATCT

NC_000962.3 3403278 3403298 TBseq_1.0_1969_RIGHT 1 - GCGTTCGAACTGCACTACCC

NC_000962.3 3402918 3402938 TBseq_1.0_1970_LEFT 2 + CGATGGCGACGTCTAGACCC

NC_000962.3 3404912 3404931 TBseq_1.0_1970_RIGHT 2 - GAGGAGTGCGTTGACAGCC

NC_000962.3 3404627 3404648 TBseq_1.0_1971_LEFT 1 + AATCGGCGTGGCATAGAACAG

NC_000962.3 3406625 3406646 TBseq_1.0_1971_RIGHT 1 - TTGCCGATCGAGTTGTAGGTG

NC_000962.3 3406389 3406410 TBseq_1.0_1972_LEFT 2 + AAGTTCGCCGGAATCTGTCAC

NC_000962.3 3408412 3408432 TBseq_1.0_1972_RIGHT 2 - TCACCGAGGACGATGACTGG

NC_000962.3 3407862 3407882 TBseq_1.0_1973_LEFT 1 + TTCACCCCGCTCGATTAGGT

NC_000962.3 3409802 3409825 TBseq_1.0_1973_RIGHT 1 - TCGAATCGCAGATCCATTACGTG

NC_000962.3 3409036 3409059 TBseq_1.0_1974_LEFT 2 + GAAGATCTGGCTGTAGCTCTTGG

NC_000962.3 3411111 3411131 TBseq_1.0_1974_RIGHT 2 - GGTGACTGAAGCGCCAAACT

NC_000962.3 3410783 3410803 TBseq_1.0_1975_LEFT 1 + GCCCAAGATTTCGTCCCAGT

NC_000962.3 3412803 3412825 TBseq_1.0_1975_RIGHT 1 - CTAGGACAGATGAACCTGCACG

NC_000962.3 3412182 3412204 TBseq_1.0_1976_LEFT 2 + CGTAAATCTGCGCCTTGTTCAC

NC_000962.3 3414178 3414198 TBseq_1.0_1976_RIGHT 2 - CGGGCGAAACGGACTATCAC

NC_000962.3 3414041 3414064 TBseq_1.0_1977_LEFT 1 + AGCTTCTCGTCCTGATTATGGAA

NC_000962.3 3416027 3416044 TBseq_1.0_1977_RIGHT 1 - TGGTCCGGTTGGCTCGT

NC_000962.3 3415240 3415266 TBseq_1.0_1978_LEFT 2 + CATTTTCGAAATTCCCTGGTCGTAAG

NC_000962.3 3417232 3417252 TBseq_1.0_1978_RIGHT 2 - GTACGGTCACCCATGATGGC

NC_000962.3 3416680 3416701 TBseq_1.0_1979_LEFT 1 + CTGTGCGGACGATGATCGATC

NC_000962.3 3418646 3418666 TBseq_1.0_1979_RIGHT 1 - GACCATGTTGCAGAGGGGAG

NC_000962.3 3417988 3418007 TBseq_1.0_1980_LEFT 2 + CCACTGAACCGTTCGACCC

NC_000962.3 3419912 3419934 TBseq_1.0_1980_RIGHT 2 - TGGAACATGTGCTCTTCGAAGT

NC_000962.3 3419158 3419177 TBseq_1.0_1981_LEFT 1 + AAGATCTGGGCGAGCACCG

NC_000962.3 3421170 3421190 TBseq_1.0_1981_RIGHT 1 - AGGGTTTTCTGCAACAGGGG

NC_000962.3 3420959 3420979 TBseq_1.0_1982_LEFT 2 + GTCCCAGGTAGGCCCTCTTC

NC_000962.3 3422964 3422985 TBseq_1.0_1982_RIGHT 2 - TTCATCTGCGAGCCAAATGCC

NC_000962.3 3422723 3422744 TBseq_1.0_1983_LEFT 1 + CAGGGTTTTTCGATTGCTCCG

NC_000962.3 3424684 3424704 TBseq_1.0_1983_RIGHT 1 - ACCATCGCCACGTTTCAAGC

NC_000962.3 3424480 3424502 TBseq_1.0_1984_LEFT 2 + TGCTCCCAGGTAAAGCCAATAC

NC_000962.3 3426481 3426501 TBseq_1.0_1984_RIGHT 2 - ATCCCGATGCAGGATGTCGA

NC_000962.3 3425561 3425581 TBseq_1.0_1985_LEFT 1 + GCGAGACATGCCAAGCTGAA

NC_000962.3 3427505 3427526 TBseq_1.0_1985_RIGHT 1 - ACTTTCATTTCGATCAGCCGC

NC_000962.3 3426941 3426960 TBseq_1.0_1986_LEFT 2 + CCAACTTAGGCCCGAGCAG

NC_000962.3 3428972 3428989 TBseq_1.0_1986_RIGHT 2 - TGTTGATGCCGCCCAGC

NC_000962.3 3428544 3428563 TBseq_1.0_1987_LEFT 1 + CATACCCACGACAGCGCAG

NC_000962.3 3430486 3430507 TBseq_1.0_1987_RIGHT 1 - TGCCATAACCCACTAGACAGC

NC_000962.3 3430096 3430118 TBseq_1.0_1988_LEFT 2 + AGGATCCAAATTCGACGGGTTG

NC_000962.3 3432054 3432076 TBseq_1.0_1988_RIGHT 2 - ACGTCTACAAGATCTACGCGGA

NC_000962.3 3431900 3431920 TBseq_1.0_1989_LEFT 1 + CGCCATAGCCCCATGAAGTG

NC_000962.3 3433833 3433852 TBseq_1.0_1989_RIGHT 1 - TAACCCACCAGGAAGGCGC

NC_000962.3 3433497 3433516 TBseq_1.0_1990_LEFT 2 + AAAGCGACCTGTTGAGCGA

NC_000962.3 3435530 3435551 TBseq_1.0_1990_RIGHT 2 - GGGGTTTCACCGGTCTTTTGC

NC_000962.3 3434201 3434221 TBseq_1.0_1991_LEFT 1 + GGACTTTTCCCTACGGCACA

NC_000962.3 3436138 3436158 TBseq_1.0_1991_RIGHT 1 - GTCGCGTTATCCCTACTCCC

NC_000962.3 3436282 3436301 TBseq_1.0_1992_LEFT 2 + CAGTCCCAGTCACATCGCA

NC_000962.3 3438225 3438245 TBseq_1.0_1992_RIGHT 2 - CGAAATCGTCTGGGCGAAGG

NC_000962.3 3436395 3436412 TBseq_1.0_1993_LEFT 1 + ACCGGGGTCACAACGCT

NC_000962.3 3438350 3438368 TBseq_1.0_1993_RIGHT 1 - CTCCAACGCGCTCAAGCT

NC_000962.3 3439723 3439742 TBseq_1.0_1994_LEFT 2 + CCGACGATTTTCACCGGCC

NC_000962.3 3441621 3441641 TBseq_1.0_1994_RIGHT 2 - CGATGGGCGCGAATTTACGG

NC_000962.3 3441388 3441408 TBseq_1.0_1995_LEFT 1 + TTCCTGATCGGCCTGCTTAC

NC_000962.3 3443450 3443472 TBseq_1.0_1995_RIGHT 1 - GTTCGCCGAGGTATATCCACTG

NC_000962.3 3443015 3443036 TBseq_1.0_1996_LEFT 2 + GATCCGCTCGTTGTTGATTCG

NC_000962.3 3444980 3444999 TBseq_1.0_1996_RIGHT 2 - GACACCCGCGACCAAGTAC

NC_000962.3 3445359 3445379 TBseq_1.0_1997_LEFT 1 + AGGGAGTACACGTCAGAGGC

NC_000962.3 3447375 3447392 TBseq_1.0_1997_RIGHT 1 - AAACCCCAGCCCCGACT

NC_000962.3 3446871 3446892 TBseq_1.0_1998_LEFT 2 + TGCTACTCGCCTACAACTCCC

NC_000962.3 3448905 3448925 TBseq_1.0_1998_RIGHT 2 - CCGGTGCAGCTGATGAGGTA

NC_000962.3 3448450 3448470 TBseq_1.0_1999_LEFT 1 + AGGCTGTCGCAAAGTGTCAA

NC_000962.3 3450355 3450374 TBseq_1.0_1999_RIGHT 1 - CACCGCAGCCATCTCTTCG

NC_000962.3 3450027 3450046 TBseq_1.0_2000_LEFT 2 + TATTACGTGGTGCCCGGCC

NC_000962.3 3452086 3452104 TBseq_1.0_2000_RIGHT 2 - CCCCATGTCGCACAGGTT

NC_000962.3 3451750 3451774 TBseq_1.0_2001_LEFT 1 + GCCCATAGAGTTCTAGAAAGGGAC

NC_000962.3 3453711 3453733 TBseq_1.0_2001_RIGHT 1 - GCCAAAAAGACGTCGTTGATGG

NC_000962.3 3452880 3452899 TBseq_1.0_2002_LEFT 2 + GCACTGACCAGCTTCCACC

NC_000962.3 3454896 3454918 TBseq_1.0_2002_RIGHT 2 - CATTCCTTGCAGTTGTCCTTGC

NC_000962.3 3454563 3454585 TBseq_1.0_2003_LEFT 1 + CGACATGGGCTATCACATTCGA

NC_000962.3 3456611 3456633 TBseq_1.0_2003_RIGHT 1 - GGCATAGATCTTGATCAGGGCC

NC_000962.3 3455474 3455491 TBseq_1.0_2004_LEFT 2 + TGCCGAGCGCGAATGTG

NC_000962.3 3457445 3457466 TBseq_1.0_2004_RIGHT 2 - GTGCAAGAGGAGATCGACGAG

NC_000962.3 3457180 3457198 TBseq_1.0_2005_LEFT 1 + TGATCTTCGCCGAGGCCA

NC_000962.3 3459106 3459126 TBseq_1.0_2005_RIGHT 1 - GGGATCGGCATGGGGTCAAT

NC_000962.3 3458146 3458166 TBseq_1.0_2006_LEFT 2 + TGCGGCGCAGTATTCTTCAC

NC_000962.3 3460140 3460159 TBseq_1.0_2006_RIGHT 2 - CCGCAAGTCGATGAAGGCC

NC_000962.3 3459763 3459782 TBseq_1.0_2007_LEFT 1 + TCGATCATCGGCAGCCTGG

NC_000962.3 3461746 3461767 TBseq_1.0_2007_RIGHT 1 - GACGGTAACCTCGATCGTGAT

NC_000962.3 3460833 3460852 TBseq_1.0_2008_LEFT 2 + GCGGTCGAAAGCAAACCAG

NC_000962.3 3462906 3462926 TBseq_1.0_2008_RIGHT 2 - CATGTATGATCTGGCGGGCG

NC_000962.3 3462403 3462421 TBseq_1.0_2009_LEFT 1 + TGGTTTCGCGCATGACCG

NC_000962.3 3464349 3464368 TBseq_1.0_2009_RIGHT 1 - GGTACAGATCAGGCGTGGG

NC_000962.3 3463442 3463461 TBseq_1.0_2010_LEFT 2 + CAGCCGGCGAACATGATGT

NC_000962.3 3465413 3465432 TBseq_1.0_2010_RIGHT 2 - ACTCGGTGCACAGGATTGG

NC_000962.3 3465161 3465183 TBseq_1.0_2011_LEFT 1 + CGCAAGGTGGAAAGGAAAGACA

NC_000962.3 3467186 3467206 TBseq_1.0_2011_RIGHT 1 - TGGGAATTCGCGCAAACTGG

NC_000962.3 3465453 3465473 TBseq_1.0_2012_LEFT 2 + GCACTGTCGAGGGAATCCTG

NC_000962.3 3467526 3467547 TBseq_1.0_2012_RIGHT 2 - GCTTGATCCACACTTCCCGTT

NC_000962.3 3467248 3467265 TBseq_1.0_2013_LEFT 1 + AGCCGCCGGGAATCGAA

NC_000962.3 3469230 3469250 TBseq_1.0_2013_RIGHT 1 - CCGTCCCCTTACCACACTGG

NC_000962.3 3468524 3468544 TBseq_1.0_2014_LEFT 2 + GTGAACTGCAGTCCGTCCAG

NC_000962.3 3470417 3470440 TBseq_1.0_2014_RIGHT 2 - GTTACGGGAGAAGATCGAAACGC

NC_000962.3 3469872 3469894 TBseq_1.0_2015_LEFT 1 + GAGGATGTCCGCATAGTCAACC

NC_000962.3 3471833 3471852 TBseq_1.0_2015_RIGHT 1 - GCGTGAAGCTCAGCAACCA

NC_000962.3 3471046 3471066 TBseq_1.0_2016_LEFT 2 + ATGACCTCCAATGCGAAGGC

NC_000962.3 3473030 3473052 TBseq_1.0_2016_RIGHT 2 - GAGAAGTCCCAACTGCAGAACA

NC_000962.3 3472498 3472518 TBseq_1.0_2017_LEFT 1 + TGAGGACGATCGACACCCAC

NC_000962.3 3474532 3474551 TBseq_1.0_2017_RIGHT 1 - GCGTGATCGGCGATATCGG

NC_000962.3 3474203 3474223 TBseq_1.0_2018_LEFT 2 + ATTCGAAAAGACGGCCGAGG

NC_000962.3 3476169 3476189 TBseq_1.0_2018_RIGHT 2 - TCATCACCGAGGCGTGGATG

NC_000962.3 3475866 3475885 TBseq_1.0_2019_LEFT 1 + TGCGTCGCTTGTAGTTCCG

NC_000962.3 3477906 3477926 TBseq_1.0_2019_RIGHT 1 - GGCAAATCTGGGCGGATCAG

NC_000962.3 3477587 3477608 TBseq_1.0_2020_LEFT 2 + CTCTCTTGAGGCGCTCTAACC

NC_000962.3 3479635 3479656 TBseq_1.0_2020_RIGHT 2 - AGCACTTCGGCGATAAACTCC

NC_000962.3 3479382 3479404 TBseq_1.0_2021_LEFT 1 + ACGTACGGGTGATCTTATTCCG

NC_000962.3 3481431 3481455 TBseq_1.0_2021_RIGHT 1 - GTCATTGCGTCATTTCCTTCGATT

NC_000962.3 3480892 3480914 TBseq_1.0_2022_LEFT 2 + TGTTACGACACTCCGAGCATTC

NC_000962.3 3482882 3482900 TBseq_1.0_2022_RIGHT 2 - GCATCCTTCAGCCGCTGT

NC_000962.3 3482556 3482575 TBseq_1.0_2023_LEFT 1 + CCTCGCCGAACAACACGAC

NC_000962.3 3484590 3484613 TBseq_1.0_2023_RIGHT 1 - ACTCCTCATCGGACTTGAAGGTG

NC_000962.3 3484442 3484463 TBseq_1.0_2024_LEFT 2 + TCAAGAACCTCATCGACGTGC

NC_000962.3 3486384 3486404 TBseq_1.0_2024_RIGHT 2 - CACCCAACCTCGCTCGAATC

NC_000962.3 3486208 3486226 TBseq_1.0_2025_LEFT 1 + AGAACCCGTTTCGCACCG

NC_000962.3 3488108 3488130 TBseq_1.0_2025_RIGHT 1 - CGTCTCCCCATTCTGGTTGTTT

NC_000962.3 3487671 3487691 TBseq_1.0_2026_LEFT 2 + GGTGGGGATCAAGGAACTCC

NC_000962.3 3489694 3489715 TBseq_1.0_2026_RIGHT 2 - GCGAAGGTTGCAAATGTACGT

NC_000962.3 3488297 3488317 TBseq_1.0_2027_LEFT 1 + GCGATATTTGTGGACGCGCA

NC_000962.3 3490315 3490337 TBseq_1.0_2027_RIGHT 1 - CGGTCAGTTCCTCTACCTTAGC

NC_000962.3 3490117 3490141 TBseq_1.0_2028_LEFT 2 + GGCTACTACGTGAACGAATACCAG

NC_000962.3 3492080 3492104 TBseq_1.0_2028_RIGHT 2 - CAAATAGGGTCATTGGTCCTCTCA

NC_000962.3 3491685 3491712 TBseq_1.0_2029_LEFT 1 + CTCGTACAAGGGTTAACTAAACAATCG

NC_000962.3 3493671 3493692 TBseq_1.0_2029_RIGHT 1 - GGCACCGTGTTTCTCAACAAG

NC_000962.3 3493311 3493332 TBseq_1.0_2030_LEFT 2 + GTTGAGGCTGTCTCGGTAGGT

NC_000962.3 3495249 3495267 TBseq_1.0_2030_RIGHT 2 - GTGTTGTCACCGTGGCGA

NC_000962.3 3494917 3494936 TBseq_1.0_2031_LEFT 1 + CACTTCAGGTTCGGTCCGG

NC_000962.3 3496840 3496860 TBseq_1.0_2031_RIGHT 1 - CAAGCGGTTGTACCCCGATG

NC_000962.3 3496650 3496670 TBseq_1.0_2032_LEFT 2 + TATGCCCGACGAGTCTGGAG

NC_000962.3 3498633 3498653 TBseq_1.0_2032_RIGHT 2 - CTCTACCAGCAGGCTAAGGC

NC_000962.3 3498324 3498344 TBseq_1.0_2033_LEFT 1 + CCTTCCAAATCGACCCGGTC

NC_000962.3 3500241 3500259 TBseq_1.0_2033_RIGHT 1 - ACGCATTCGAGGAGGCCA

NC_000962.3 3498969 3498991 TBseq_1.0_2034_LEFT 2 + ACAAAGTGCAATACCCGATGCT

NC_000962.3 3500947 3500969 TBseq_1.0_2034_RIGHT 2 - GGGACTTTGCTGTGAAAAGCTG

NC_000962.3 3501119 3501141 TBseq_1.0_2035_LEFT 1 + TCAGTCGACTGCCATACAACCT

NC_000962.3 3503099 3503117 TBseq_1.0_2035_RIGHT 1 - TCACCATCGCCGGGATCA

NC_000962.3 3503218 3503240 TBseq_1.0_2036_LEFT 2 + GCAAGAAATACCGCTAGGACCG

NC_000962.3 3505146 3505168 TBseq_1.0_2036_RIGHT 2 - GTGAGGGCGTAATGTCGTATCG

NC_000962.3 3504860 3504881 TBseq_1.0_2037_LEFT 1 + CTGCTGATTCCCGGACGTAAC

NC_000962.3 3506857 3506877 TBseq_1.0_2037_RIGHT 1 - CTATCGGCCGCATCATCTCC

NC_000962.3 3505992 3506014 TBseq_1.0_2038_LEFT 2 + CAGCAGGCCACCTATCTCAAAG

NC_000962.3 3508040 3508059 TBseq_1.0_2038_RIGHT 2 - CCAGCAGACGCAAAAGCCC

NC_000962.3 3507814 3507833 TBseq_1.0_2039_LEFT 1 + CAGCGACGTCACCTGCAAA

NC_000962.3 3509788 3509808 TBseq_1.0_2039_RIGHT 1 - TGGCGTATCACCATCGGACC

NC_000962.3 3509599 3509618 TBseq_1.0_2040_LEFT 2 + CTCGTGCGGGATTACACCC

NC_000962.3 3511576 3511598 TBseq_1.0_2040_RIGHT 2 - CCGTCACAGTGACCTTCAACAT

NC_000962.3 3511368 3511390 TBseq_1.0_2041_LEFT 1 + CCAACGATTAACCGGCAAATGC

NC_000962.3 3513448 3513467 TBseq_1.0_2041_RIGHT 1 - CCATGTTGACGACGATGCG

NC_000962.3 3512221 3512241 TBseq_1.0_2042_LEFT 2 + GGTTTGACATTGCGCGGTTC

NC_000962.3 3514211 3514234 TBseq_1.0_2042_RIGHT 2 - CCACATCTCTTTGACGCGAATCA

NC_000962.3 3513869 3513889 TBseq_1.0_2043_LEFT 1 + TCACCGGTTTGCGGATGAAC

NC_000962.3 3515807 3515827 TBseq_1.0_2043_RIGHT 1 - ACCTCACCGCGTACGTAGAC

NC_000962.3 3515271 3515290 TBseq_1.0_2044_LEFT 2 + CAAACATCGCGCATCCTGG

NC_000962.3 3517197 3517216 TBseq_1.0_2044_RIGHT 2 - CACCTGCGCGGAGATGTTG

NC_000962.3 3517781 3517799 TBseq_1.0_2045_LEFT 1 + CCAAGTTCGCGCGGATCA

NC_000962.3 3519858 3519880 TBseq_1.0_2045_RIGHT 1 - AACAACAGGAAGGCATACCAGA

NC_000962.3 3519142 3519162 TBseq_1.0_2046_LEFT 2 + GTGAAAATCGGAGCGGGCTC

NC_000962.3 3521055 3521079 TBseq_1.0_2046_RIGHT 2 - GCCTAGGTAGTAGTCCTTATCGGT

NC_000962.3 3520848 3520865 TBseq_1.0_2047_LEFT 1 + TACGGCCGGGTGTACCA

NC_000962.3 3522807 3522831 TBseq_1.0_2047_RIGHT 1 - CGAAAGTGCTAAACGTCAAGAACA

NC_000962.3 3522197 3522219 TBseq_1.0_2048_LEFT 2 + ACGACGCGAATCTACTCAAAGG

NC_000962.3 3524227 3524246 TBseq_1.0_2048_RIGHT 2 - CAACCATACCGGCCCACTT

NC_000962.3 3523526 3523547 TBseq_1.0_2049_LEFT 1 + GTCACCGCGTTCTACATGACG

NC_000962.3 3525428 3525450 TBseq_1.0_2049_RIGHT 1 - AGCAGAACCAGGAATTCGCTGA

NC_000962.3 3525173 3525193 TBseq_1.0_2050_LEFT 2 + TCTCGCACTTCGGGTTCATC

NC_000962.3 3527160 3527181 TBseq_1.0_2050_RIGHT 2 - GGAGATCACGCCGACGATTAC

NC_000962.3 3527200 3527219 TBseq_1.0_2051_LEFT 1 + TCTACGTGCGGGTGATCGT

NC_000962.3 3529232 3529257 TBseq_1.0_2051_RIGHT 1 - GCCGTTTTCCTACAGTTTGTTCTAC

NC_000962.3 3529188 3529207 TBseq_1.0_2052_LEFT 2 + CGTCCCGGCCAATGACTCA

NC_000962.3 3531230 3531250 TBseq_1.0_2052_RIGHT 2 - GTTGGCTACCTACGTGCTGG

NC_000962.3 3530823 3530844 TBseq_1.0_2053_LEFT 1 + GGGTTCCACCATCAGCATCAA

NC_000962.3 3532863 3532883 TBseq_1.0_2053_RIGHT 1 - TGGCGTGCATCACAACTGAC

NC_000962.3 3532713 3532733 TBseq_1.0_2054_LEFT 2 + TCACCGTGCACCTGGATCAC

NC_000962.3 3534625 3534645 TBseq_1.0_2054_RIGHT 2 - CGTGTTACGTGGCGATGGAG

NC_000962.3 3534467 3534488 TBseq_1.0_2055_LEFT 1 + TTCATCACGTGCGGGCTAAAA

NC_000962.3 3536395 3536415 TBseq_1.0_2055_RIGHT 1 - ACCAGCCGGATCACTTCGAA

NC_000962.3 3536233 3536253 TBseq_1.0_2056_LEFT 2 + GTGGAAAGTGGCGTGGACTC

NC_000962.3 3538215 3538232 TBseq_1.0_2056_RIGHT 2 - TCGATGACGCCGAAGCC

NC_000962.3 3537537 3537558 TBseq_1.0_2057_LEFT 1 + CTGCGAAAACTGCGTATCGTG

NC_000962.3 3539505 3539524 TBseq_1.0_2057_RIGHT 1 - CCCCATCAGAATGCCTGGC

NC_000962.3 3539109 3539129 TBseq_1.0_2058_LEFT 2 + CAAAAATGGTGCCCAGCAGG

NC_000962.3 3541155 3541174 TBseq_1.0_2058_RIGHT 2 - AGCACGCATGGGTAGAGGC

NC_000962.3 3540915 3540939 TBseq_1.0_2059_LEFT 1 + GGATCACAGAATAATAACGTCGCG

NC_000962.3 3542957 3542980 TBseq_1.0_2059_RIGHT 1 - TTCGGGAAAGATACAGCTCGACT

NC_000962.3 3542333 3542352 TBseq_1.0_2060_LEFT 2 + AGGCAGCCGACTTAGCAAC

NC_000962.3 3544357 3544376 TBseq_1.0_2060_RIGHT 2 - GAGGTTATTGCGGCCCTGC

NC_000962.3 3544020 3544039 TBseq_1.0_2061_LEFT 1 + TGGGCCGGATTCTTCAACG

NC_000962.3 3546031 3546053 TBseq_1.0_2061_RIGHT 1 - GCACTGTAATCGATTTGTCGCG

NC_000962.3 3545875 3545896 TBseq_1.0_2062_LEFT 2 + GGCATCGGCAAGGTTACTTTC

NC_000962.3 3547827 3547847 TBseq_1.0_2062_RIGHT 2 - GCGGACATGATGTACCCCTC

NC_000962.3 3547191 3547210 TBseq_1.0_2063_LEFT 1 + AGCCGGTCCCTCAAGAACC

NC_000962.3 3549162 3549181 TBseq_1.0_2063_RIGHT 1 - CGTCGGTTGTTCAGCTCCC

NC_000962.3 3548672 3548693 TBseq_1.0_2064_LEFT 2 + CGACTGCATTCTTCAGACCCC

NC_000962.3 3550607 3550624 TBseq_1.0_2064_RIGHT 2 - TTGTCACCGCCCAGCAG

NC_000962.3 3550406 3550426 TBseq_1.0_2065_LEFT 1 + TGGTTCTTCGCGCTCAACAG

NC_000962.3 3552360 3552382 TBseq_1.0_2065_RIGHT 1 - GGTTTGATCAGCTCGGTCTTGT

NC_000962.3 3552081 3552101 TBseq_1.0_2066_LEFT 2 + CCTTTGTCACCGACGCCTAC

NC_000962.3 3554118 3554138 TBseq_1.0_2066_RIGHT 2 - ATTTCGGCCTGGGATTGCTG

NC_000962.3 3553927 3553946 TBseq_1.0_2067_LEFT 1 + TCAACCATCGCCGCCTCTA

NC_000962.3 3555862 3555882 TBseq_1.0_2067_RIGHT 1 - AGCTCGACGGGGAAGAAATC

NC_000962.3 3555613 3555632 TBseq_1.0_2068_LEFT 2 + CGCGGTCACGTCTTTGTTG

NC_000962.3 3557575 3557593 TBseq_1.0_2068_RIGHT 2 - CGAAATGGCCCGGCACAT

NC_000962.3 3557390 3557410 TBseq_1.0_2069_LEFT 1 + GGCGGGGTCGGTTATTGATC

NC_000962.3 3559405 3559425 TBseq_1.0_2069_RIGHT 1 - GGTAGCTCGCTGGGCTCATA

NC_000962.3 3559071 3559090 TBseq_1.0_2070_LEFT 2 + GCATCACCGACGAGACCTC

NC_000962.3 3561048 3561067 TBseq_1.0_2070_RIGHT 2 - GTTCAAGGTGCAGCGCATG

NC_000962.3 3560677 3560697 TBseq_1.0_2071_LEFT 1 + CGGATGCGGTTTTGGTTGTC

NC_000962.3 3562623 3562645 TBseq_1.0_2071_RIGHT 1 - TTCGGTATTTCTGGCATTCGTG

NC_000962.3 3562231 3562252 TBseq_1.0_2072_LEFT 2 + GGTTTGACGCTGATTTGCTCG

NC_000962.3 3564233 3564253 TBseq_1.0_2072_RIGHT 2 - CTGGTGCCGATCGTGGTATT

NC_000962.3 3563882 3563905 TBseq_1.0_2073_LEFT 1 + GCTTTCGGATACTTCAGGTACCC

NC_000962.3 3565832 3565853 TBseq_1.0_2073_RIGHT 1 - CAACGAATACAGAGCAGACGC

NC_000962.3 3565140 3565159 TBseq_1.0_2074_LEFT 2 + CTGTTCAGCCACGTTCCCT

NC_000962.3 3567159 3567178 TBseq_1.0_2074_RIGHT 2 - ATCAGCTCGGCGGTAACCT

NC_000962.3 3566620 3566640 TBseq_1.0_2075_LEFT 1 + GTTCGATCTCAACGCTGGCT

NC_000962.3 3568552 3568574 TBseq_1.0_2075_RIGHT 1 - CTCGAGGTAGCCAAGACACTGT

NC_000962.3 3568145 3568169 TBseq_1.0_2076_LEFT 2 + CGCAAGTGGTTACAGAAAATGACC

NC_000962.3 3570179 3570200 TBseq_1.0_2076_RIGHT 2 - TTCCATGAGCATTCCGACGAG

NC_000962.3 3570010 3570027 TBseq_1.0_2077_LEFT 1 + AAAACCCCTCGCCACCG

NC_000962.3 3571991 3572008 TBseq_1.0_2077_RIGHT 1 - ACGACGGCGCTGATCGT

NC_000962.3 3571389 3571409 TBseq_1.0_2078_LEFT 2 + GACTCAAAACAGCGCTCACG

NC_000962.3 3573340 3573363 TBseq_1.0_2078_RIGHT 2 - GCGATCAACATCTTCGTCATCAC

NC_000962.3 3575843 3575860 TBseq_1.0_2079_LEFT 1 + TCGGGAAGCAACGCGCA

NC_000962.3 3577815 3577834 TBseq_1.0_2079_RIGHT 1 - CCCGGTCAGCACGAGTGAT

NC_000962.3 3576581 3576600 TBseq_1.0_2080_LEFT 2 + ACCACATCGAATGCCAGCT

NC_000962.3 3578622 3578641 TBseq_1.0_2080_RIGHT 2 - CTCAGGAAGTCTGGGTGGC

NC_000962.3 3578621 3578640 TBseq_1.0_2081_LEFT 1 + GGCCACCCAGACTTCCTGA

NC_000962.3 3580537 3580557 TBseq_1.0_2081_RIGHT 1 - CCGTAGCGCTGTACGTGAAG

NC_000962.3 3580214 3580234 TBseq_1.0_2082_LEFT 2 + ACGAGGGTCTGACAAGCTCG

NC_000962.3 3582180 3582200 TBseq_1.0_2082_RIGHT 2 - GCATGATGAGGTCGTCTCGG

NC_000962.3 3581853 3581872 TBseq_1.0_2083_LEFT 1 + AGTAGGGCGTGATGTCGGT

NC_000962.3 3583785 3583805 TBseq_1.0_2083_RIGHT 1 - CCTAACCCGGGGAAGCAAAG

NC_000962.3 3583590 3583609 TBseq_1.0_2084_LEFT 2 + GAGCCTCTTCTGCCCGTCA

NC_000962.3 3585630 3585647 TBseq_1.0_2084_RIGHT 2 - AACTGCACGGTGCCCTC

NC_000962.3 3585204 3585223 TBseq_1.0_2085_LEFT 1 + CGGGCGGGAGTCAGTAAAC

NC_000962.3 3587220 3587238 TBseq_1.0_2085_RIGHT 1 - TGACGACGCCGAGCACAT

NC_000962.3 3586924 3586943 TBseq_1.0_2086_LEFT 2 + ACCACTACCGGACACCACC

NC_000962.3 3588822 3588843 TBseq_1.0_2086_RIGHT 2 - TCGGGGCACTGATAGTTGATC

NC_000962.3 3587863 3587882 TBseq_1.0_2087_LEFT 1 + CGCGCATTAGGGGAAGAGG

NC_000962.3 3589895 3589913 TBseq_1.0_2087_RIGHT 1 - ACAGTTCCAGGCGCGTGT

NC_000962.3 3589250 3589269 TBseq_1.0_2088_LEFT 2 + CCGCATCAGATTCCGGGTC

NC_000962.3 3591259 3591276 TBseq_1.0_2088_RIGHT 2 - AACGCCGTGCTGGTCAC

NC_000962.3 3589941 3589960 TBseq_1.0_2089_LEFT 1 + CTACGGCGAGATCGATGCC

NC_000962.3 3592009 3592029 TBseq_1.0_2089_RIGHT 1 - GCTGTCAGCGCGATCGTTTA

NC_000962.3 3591608 3591629 TBseq_1.0_2090_LEFT 2 + GACAGGCCTAGGATGTCGTCG

NC_000962.3 3593601 3593620 TBseq_1.0_2090_RIGHT 2 - TCCACATAGATGCCCGCGA

NC_000962.3 3593050 3593070 TBseq_1.0_2091_LEFT 1 + CTCGACGACGGCAATCGATC

NC_000962.3 3595092 3595112 TBseq_1.0_2091_RIGHT 1 - CGGACTGGAGTTGGACACGA

NC_000962.3 3593906 3593925 TBseq_1.0_2092_LEFT 2 + CCAAGCGCGATGATTCCCA

NC_000962.3 3595804 3595827 TBseq_1.0_2092_RIGHT 2 - GATTACAGACCAGTTTCGCGTCA

NC_000962.3 3596245 3596264 TBseq_1.0_2093_LEFT 1 + CGTTCCGCTCGAATCGTCA

NC_000962.3 3598248 3598266 TBseq_1.0_2093_RIGHT 1 - TGGACCCTGCTTGACGGT

NC_000962.3 3597765 3597786 TBseq_1.0_2094_LEFT 2 + TCATCACCCCACTCTTTTCGG

NC_000962.3 3599774 3599796 TBseq_1.0_2094_RIGHT 2 - CGATTCAGCGCTGGAAATAACC

NC_000962.3 3599076 3599095 TBseq_1.0_2095_LEFT 1 + CGTAGTAGACCGCCATCCG

NC_000962.3 3601094 3601116 TBseq_1.0_2095_RIGHT 1 - CGTACTACCAAGACACCAACCC

NC_000962.3 3600324 3600343 TBseq_1.0_2096_LEFT 2 + CCGACGGCCTACATGATGG

NC_000962.3 3602385 3602405 TBseq_1.0_2096_RIGHT 2 - CGGGATCGTCACTCTATCGC

NC_000962.3 3601908 3601930 TBseq_1.0_2097_LEFT 1 + GCATCTTTGCCCTTAGGACACG

NC_000962.3 3603928 3603947 TBseq_1.0_2097_RIGHT 1 - CGGTGTGTTGGACGGTCAG

NC_000962.3 3603665 3603684 TBseq_1.0_2098_LEFT 2 + CGGTGTTGCGGTTTGTTCC

NC_000962.3 3605708 3605729 TBseq_1.0_2098_RIGHT 2 - CATGCGGCTAGTCAAGTCTGG

NC_000962.3 3605232 3605250 TBseq_1.0_2099_LEFT 1 + AAGCAGTTCGCCGACCTG

NC_000962.3 3607196 3607216 TBseq_1.0_2099_RIGHT 1 - GAAGGCCATGTTCGGGATCT

NC_000962.3 3606419 3606439 TBseq_1.0_2100_LEFT 2 + TGAGAATTCACGCAACCGGG

NC_000962.3 3608398 3608415 TBseq_1.0_2100_RIGHT 2 - TCTCGCCTGGTACGCCA

NC_000962.3 3608009 3608030 TBseq_1.0_2101_LEFT 1 + GGTTTGATGAACAGGGTCGCG

NC_000962.3 3610000 3610021 TBseq_1.0_2101_RIGHT 1 - TCTGTTGATCACCAATGCCCC

NC_000962.3 3609363 3609383 TBseq_1.0_2102_LEFT 2 + GGTTGTACCAGGACCGATCG

NC_000962.3 3611330 3611350 TBseq_1.0_2102_RIGHT 2 - CTGGAGAGTGGCAGACGAGT

NC_000962.3 3610556 3610577 TBseq_1.0_2103_LEFT 1 + TTGGCCGGAATTCGTTACCAA

NC_000962.3 3612607 3612626 TBseq_1.0_2103_RIGHT 1 - CCTACTTGCCGCTGTTCGC

NC_000962.3 3612027 3612047 TBseq_1.0_2104_LEFT 2 + CGCCATGACGAACACATACG

NC_000962.3 3613985 3614005 TBseq_1.0_2104_RIGHT 2 - ACGGTGTTGACGTCGACTTT

NC_000962.3 3613741 3613761 TBseq_1.0_2105_LEFT 1 + GAGTAGGTCGCGCTCTTCGA

NC_000962.3 3615723 3615744 TBseq_1.0_2105_RIGHT 1 - GTTGGTAAAGGTGCTGGCAAC

NC_000962.3 3615269 3615289 TBseq_1.0_2106_LEFT 2 + CGGGCGTACGTTTTCCAAGA

NC_000962.3 3617206 3617225 TBseq_1.0_2106_RIGHT 2 - GCTGCGATACTCGTGCCTG

NC_000962.3 3616701 3616725 TBseq_1.0_2107_LEFT 1 + TGGGTAAGGCCATAGCAGTAAAAA

NC_000962.3 3618778 3618799 TBseq_1.0_2107_RIGHT 1 - GGGAGTCGCGCTTCTATTTGT

NC_000962.3 3618598 3618620 TBseq_1.0_2108_LEFT 2 + CGTATTTGAGGACGTTCTTGCG

NC_000962.3 3620547 3620572 TBseq_1.0_2108_RIGHT 2 - TCGCCTTATCTAAAGACTCCTACAC

NC_000962.3 3620354 3620377 TBseq_1.0_2109_LEFT 1 + GAGGGTTTCTGGGTTTTTCTGGT

NC_000962.3 3622313 3622335 TBseq_1.0_2109_RIGHT 1 - CTGCGTTATCTGGAAAGCGTGA

NC_000962.3 3621187 3621208 TBseq_1.0_2110_LEFT 2 + CATTACGGCCCTTGAACACGA

NC_000962.3 3623152 3623172 TBseq_1.0_2110_RIGHT 2 - TATTGCCCGGATGAGTGCTG

NC_000962.3 3622897 3622918 TBseq_1.0_2111_LEFT 1 + CCAATCCGAACCGTGACTTCC

NC_000962.3 3624950 3624971 TBseq_1.0_2111_RIGHT 1 - CGATGCCACCGGAATACAAGG

NC_000962.3 3624812 3624831 TBseq_1.0_2112_LEFT 2 + CGTTCGACGGTTCCGATGG

NC_000962.3 3626725 3626745 TBseq_1.0_2112_RIGHT 2 - GGGCCAAGGTCGAAAAGGAT

NC_000962.3 3626541 3626559 TBseq_1.0_2113_LEFT 1 + ACTGAGGCCCCGTGTCAT

NC_000962.3 3628573 3628592 TBseq_1.0_2113_RIGHT 1 - GAAGGACCACGCGATCCTG

NC_000962.3 3628349 3628368 TBseq_1.0_2114_LEFT 2 + ATCTGGGCGATCGTCTGGT

NC_000962.3 3630351 3630371 TBseq_1.0_2114_RIGHT 2 - CTACTGTCGCGCCTGTGAAG

NC_000962.3 3630085 3630107 TBseq_1.0_2115_LEFT 1 + AACGCTTCGTAAAAGTTGCCGA

NC_000962.3 3632077 3632098 TBseq_1.0_2115_RIGHT 1 - GAACTAGCTTCGCCGCGTATT

NC_000962.3 3631757 3631778 TBseq_1.0_2116_LEFT 2 + GTCAAGAAGCGGCAACAAGAC

NC_000962.3 3633735 3633755 TBseq_1.0_2116_RIGHT 2 - CTCGAAAACCGTCACCGTGG

NC_000962.3 3633465 3633485 TBseq_1.0_2117_LEFT 1 + ACCGAGACGCCGAAAACAAC

NC_000962.3 3635454 3635474 TBseq_1.0_2117_RIGHT 1 - CCTATGTGCGTGGTTTCGGT

NC_000962.3 3635179 3635202 TBseq_1.0_2118_LEFT 2 + CCCTCGGTGCATAACAAGATCTG

NC_000962.3 3637172 3637191 TBseq_1.0_2118_RIGHT 2 - ACGAGGGCGAACTGGACTT

NC_000962.3 3636094 3636114 TBseq_1.0_2119_LEFT 1 + AGCAACGAGGTTTGGCCATG

NC_000962.3 3638111 3638131 TBseq_1.0_2119_RIGHT 1 - GGTCCTTGGGGTGATCGACT

NC_000962.3 3637760 3637783 TBseq_1.0_2120_LEFT 2 + CCTTGATATAGGAGTGCCCAACC

NC_000962.3 3639747 3639769 TBseq_1.0_2120_RIGHT 2 - GTGCAGCAATTCACCAAGTTCC

NC_000962.3 3638635 3638654 TBseq_1.0_2121_LEFT 1 + ATCTCTTCGCCGACCAGCC

NC_000962.3 3640530 3640551 TBseq_1.0_2121_RIGHT 1 - GACCTTCACATCGCAGGACAG

NC_000962.3 3640364 3640391 TBseq_1.0_2122_LEFT 2 + CGAAATGACACTGATGTGATTAGACAC

NC_000962.3 3642303 3642321 TBseq_1.0_2122_RIGHT 2 - CGGACCTGCGCAACAGTT

NC_000962.3 3642073 3642094 TBseq_1.0_2123_LEFT 1 + CGACCCATACGGCAATGAGTT

NC_000962.3 3644036 3644058 TBseq_1.0_2123_RIGHT 1 - CATAGTGGCGGACTGTTTGAGT

NC_000962.3 3643757 3643777 TBseq_1.0_2124_LEFT 2 + GCAGCTACGCGAATTCCTGC

NC_000962.3 3645722 3645744 TBseq_1.0_2124_RIGHT 2 - GATCGAATACGTGACCGAGGAG

NC_000962.3 3645635 3645656 TBseq_1.0_2125_LEFT 1 + GAGCACATCGCCGTTAAACAC

NC_000962.3 3647615 3647634 TBseq_1.0_2125_RIGHT 1 - TCAATTGCGCCGCCTACAC

NC_000962.3 3647020 3647038 TBseq_1.0_2126_LEFT 2 + CGACAGCGCGGAATAGCT

NC_000962.3 3649040 3649060 TBseq_1.0_2126_RIGHT 2 - GGTGGTAAACCCCTTGGAGC

NC_000962.3 3649022 3649043 TBseq_1.0_2127_LEFT 1 + CGGTGTCAAAGGTGTTGAGCT

NC_000962.3 3651022 3651039 TBseq_1.0_2127_RIGHT 1 - CGCGCAGGATGAGGCTT

NC_000962.3 3650214 3650236 TBseq_1.0_2128_LEFT 2 + CGGAAGAAAGGTACAGGCAATG

NC_000962.3 3652278 3652297 TBseq_1.0_2128_RIGHT 2 - TTGTCCTCCGGCATGACCT

NC_000962.3 3651526 3651546 TBseq_1.0_2129_LEFT 1 + CTTCGTTCCCACCTCGTTCA

NC_000962.3 3653546 3653567 TBseq_1.0_2129_RIGHT 1 - CTTGATGACTTCAGCCCCCAG

NC_000962.3 3652948 3652968 TBseq_1.0_2130_LEFT 2 + GATGCCGATCACCGAGGTCT

NC_000962.3 3654954 3654975 TBseq_1.0_2130_RIGHT 2 - GGCGATCGTCATCAGACACAG

NC_000962.3 3654107 3654127 TBseq_1.0_2131_LEFT 1 + CATCTCAATCGGGCCGCTAG

NC_000962.3 3656063 3656084 TBseq_1.0_2131_RIGHT 1 - TCGATGTAGTCTGCGTTCAGG

NC_000962.3 3655880 3655900 TBseq_1.0_2132_LEFT 2 + GGTCGTGGCGATCGTATTCC

NC_000962.3 3657903 3657923 TBseq_1.0_2132_RIGHT 2 - ATTCCTCCGGTTTCAACGCC

NC_000962.3 3656812 3656835 TBseq_1.0_2133_LEFT 1 + AGGTCATCGGCATATTCTTCGAC

NC_000962.3 3658839 3658858 TBseq_1.0_2133_RIGHT 1 - TTCACCTCTACGGCAAGGC

NC_000962.3 3658482 3658501 TBseq_1.0_2134_LEFT 2 + GAACATCGCCTCAGGGGTG

NC_000962.3 3660442 3660462 TBseq_1.0_2134_RIGHT 2 - CGCCATTTGCAGCAAGTTGC

NC_000962.3 3660121 3660144 TBseq_1.0_2135_LEFT 1 + GCACCACATTCATCATCGACACA

NC_000962.3 3662191 3662212 TBseq_1.0_2135_RIGHT 1 - CTTTTTCGACGGCATCCTCAC

NC_000962.3 3660775 3660795 TBseq_1.0_2136_LEFT 2 + ACGTCCCGGTGCGAAAAATC

NC_000962.3 3662683 3662704 TBseq_1.0_2136_RIGHT 2 - ACATCTGGCTGGTCTGATCGA

NC_000962.3 3662395 3662413 TBseq_1.0_2137_LEFT 1 + TCTTCAGCCAGGACGCCA

NC_000962.3 3664407 3664425 TBseq_1.0_2137_RIGHT 1 - TTGCGTTCCGGTCAGCGT

NC_000962.3 3663908 3663926 TBseq_1.0_2138_LEFT 2 + AGACGAACAATCCGGCCC

NC_000962.3 3665846 3665867 TBseq_1.0_2138_RIGHT 2 - TCGGATACCACCTCTGCTAGC

NC_000962.3 3665439 3665457 TBseq_1.0_2139_LEFT 1 + TCAGCCGCTGATCGACGT

NC_000962.3 3667420 3667439 TBseq_1.0_2139_RIGHT 1 - GGGCGCCGGTAGGAAGTTA

NC_000962.3 3667205 3667226 TBseq_1.0_2140_LEFT 2 + GTCGAATACCTGGTCGGTCAG

NC_000962.3 3669237 3669256 TBseq_1.0_2140_RIGHT 2 - CTGGATTTCGACGCCGTGG

NC_000962.3 3669032 3669051 TBseq_1.0_2141_LEFT 1 + AAGGTCTGGACGTCGTCGG

NC_000962.3 3671075 3671095 TBseq_1.0_2141_RIGHT 1 - TGTTTTGTGGCCGAACCCAT

NC_000962.3 3670831 3670851 TBseq_1.0_2142_LEFT 2 + GAACACATTGTCGGCGACCT

NC_000962.3 3672787 3672806 TBseq_1.0_2142_RIGHT 2 - GCGGGGTCAAAGAGTTGCC

NC_000962.3 3672925 3672946 TBseq_1.0_2143_LEFT 1 + CCTGGTACGACGAGTTGTCCA

NC_000962.3 3674905 3674924 TBseq_1.0_2143_RIGHT 1 - CCCGATGTTGACGTTGGCG

NC_000962.3 3674680 3674699 TBseq_1.0_2144_LEFT 2 + CTCACCGGGCGCCTACTAT

NC_000962.3 3676730 3676750 TBseq_1.0_2144_RIGHT 2 - GACAAAAACCGCCTACCGCT

NC_000962.3 3675849 3675869 TBseq_1.0_2145_LEFT 1 + TCGACGTTCCTAGCTTTCGC

NC_000962.3 3677843 3677862 TBseq_1.0_2145_RIGHT 1 - GCCACCACCGCTTTGAGTT

NC_000962.3 3678461 3678480 TBseq_1.0_2146_LEFT 2 + GTGACGTGATCTCGCTGGG

NC_000962.3 3680462 3680483 TBseq_1.0_2146_RIGHT 2 - CGGCCCAAATCAATTCCCACA

NC_000962.3 3679526 3679543 TBseq_1.0_2147_LEFT 1 + TAGCCCGTGACGCCGAA

NC_000962.3 3681467 3681487 TBseq_1.0_2147_RIGHT 1 - TCGGATGAACAGGTGCTTGC

NC_000962.3 3680922 3680941 TBseq_1.0_2148_LEFT 2 + CGAACAGCCGGACTACCAC

NC_000962.3 3682915 3682934 TBseq_1.0_2148_RIGHT 2 - TCGGTAGCGACGTTCCAGT

NC_000962.3 3682220 3682241 TBseq_1.0_2149_LEFT 1 + GGTATGTCCGCCATTGTCGAT

NC_000962.3 3684284 3684304 TBseq_1.0_2149_RIGHT 1 - ATCGAACAATACGGCGGCAT

NC_000962.3 3683997 3684017 TBseq_1.0_2150_LEFT 2 + GCACGAAACTGGTTCCGTCC

NC_000962.3 3685972 3685992 TBseq_1.0_2150_RIGHT 2 - CCGGCCGTGACACACATATC

NC_000962.3 3685530 3685549 TBseq_1.0_2151_LEFT 1 + TTGGAGAGCTAGCCAACGC

NC_000962.3 3687558 3687578 TBseq_1.0_2151_RIGHT 1 - CATGCGGACGGTCTATCACC

NC_000962.3 3687051 3687069 TBseq_1.0_2152_LEFT 2 + CGTGCCGCCATTCTCGAT

NC_000962.3 3689062 3689082 TBseq_1.0_2152_RIGHT 2 - TTGTTCCCGCTGACCAAACG

NC_000962.3 3688678 3688700 TBseq_1.0_2153_LEFT 1 + AACGCTTGAATCTCATCGGTCC

NC_000962.3 3690693 3690714 TBseq_1.0_2153_RIGHT 1 - CTTCGACGACGCCAAGATCTC

NC_000962.3 3690211 3690231 TBseq_1.0_2154_LEFT 2 + TTTTGAACAGCCGGACTCCC

NC_000962.3 3692286 3692306 TBseq_1.0_2154_RIGHT 2 - GGCTTGAAGTCGGCAAGGTC

NC_000962.3 3691952 3691972 TBseq_1.0_2155_LEFT 1 + CCAACGGTAATAGCAACGCC

NC_000962.3 3693843 3693860 TBseq_1.0_2155_RIGHT 1 - AAGGCTCAGGCGCTGGT

NC_000962.3 3692713 3692732 TBseq_1.0_2156_LEFT 2 + CCCAACTCCGGATAGCGGT

NC_000962.3 3694727 3694750 TBseq_1.0_2156_RIGHT 2 - CCAGATTTGTCACCAGGGATACG

NC_000962.3 3695260 3695280 TBseq_1.0_2157_LEFT 1 + AGATCACGGCGTCACACAAC

NC_000962.3 3697235 3697255 TBseq_1.0_2157_RIGHT 1 - CCAAGGCACGGTATACCCTG

NC_000962.3 3696866 3696885 TBseq_1.0_2158_LEFT 2 + AACACAGGAACCAGCAGGG

NC_000962.3 3698932 3698952 TBseq_1.0_2158_RIGHT 2 - CATTGCGGCCCAGGAAGATC

NC_000962.3 3698357 3698377 TBseq_1.0_2159_LEFT 1 + CTCAATCCGGCCGAAGACAA

NC_000962.3 3700340 3700361 TBseq_1.0_2159_RIGHT 1 - CTCTGATCGGCATACCATCGC

NC_000962.3 3699756 3699778 TBseq_1.0_2160_LEFT 2 + CCAACAACGTAGAAAACAGCCC

NC_000962.3 3701695 3701715 TBseq_1.0_2160_RIGHT 2 - CAATGTCCCGGGAGATCGCA

NC_000962.3 3701160 3701181 TBseq_1.0_2161_LEFT 1 + ACGGAATGAACGCGGATTTCA

NC_000962.3 3703198 3703217 TBseq_1.0_2161_RIGHT 1 - TGGACAAGCATTCCACCGG

NC_000962.3 3702934 3702952 TBseq_1.0_2162_LEFT 2 + ACGGTGCCGGTAATGTCG

NC_000962.3 3704835 3704858 TBseq_1.0_2162_RIGHT 2 - GGGTCGAATGTCACTATCACGTA

NC_000962.3 3703697 3703719 TBseq_1.0_2163_LEFT 1 + GCACAAAGTCAAGCCATACGAG

NC_000962.3 3705665 3705684 TBseq_1.0_2163_RIGHT 1 - GGAACACGATGCCGATGCC

NC_000962.3 3705483 3705503 TBseq_1.0_2164_LEFT 2 + TTTACGCGCTGGATTTGGCT

NC_000962.3 3707474 3707495 TBseq_1.0_2164_RIGHT 2 - AGGATTCGGTGCAGTTGAACG

NC_000962.3 3706442 3706460 TBseq_1.0_2165_LEFT 1 + ATCCACGCGCTCAAGGAG

NC_000962.3 3708496 3708515 TBseq_1.0_2165_RIGHT 1 - CACGAGGGGCAGGGTAACT

NC_000962.3 3707697 3707720 TBseq_1.0_2166_LEFT 2 + ACTGATCGAAGGTGACAAATCGG

NC_000962.3 3709706 3709731 TBseq_1.0_2166_RIGHT 2 - CCGAGGATGGATGTTGAATGATTAC

NC_000962.3 3709526 3709546 TBseq_1.0_2167_LEFT 1 + GCTGGCGCTATGGATTCGTT

NC_000962.3 3711512 3711534 TBseq_1.0_2167_RIGHT 1 - GGTTTGATCAGCTCGGTCTTGT

NC_000962.3 3711233 3711253 TBseq_1.0_2168_LEFT 2 + CCTTTGTCACCGACGCCTAC

NC_000962.3 3713239 3713257 TBseq_1.0_2168_RIGHT 2 - ATGCGGGTCTGCGGTCAT

NC_000962.3 3711749 3711771 TBseq_1.0_2169_LEFT 1 + TGGTTGTTGTTGGAACCGATGC

NC_000962.3 3713651 3713671 TBseq_1.0_2169_RIGHT 1 - TGGTCCGGTTCATACTCGGG

NC_000962.3 3715682 3715705 TBseq_1.0_2170_LEFT 2 + CCGGACGCAAATATCTTCATCTG

NC_000962.3 3717620 3717639 TBseq_1.0_2170_RIGHT 2 - GCTGATACGCGGTCACCAA

NC_000962.3 3717621 3717640 TBseq_1.0_2171_LEFT 1 + TGGTGACCGCGTATCAGCT

NC_000962.3 3719522 3719542 TBseq_1.0_2171_RIGHT 1 - CACCGTCCGGAAGAGCTGAT

NC_000962.3 3719162 3719182 TBseq_1.0_2172_LEFT 2 + CGGATGCGACCTACACACAG

NC_000962.3 3721176 3721193 TBseq_1.0_2172_RIGHT 2 - AGTGCGGGCGATCGTGA

NC_000962.3 3720626 3720646 TBseq_1.0_2173_LEFT 1 + GACGGCGGGGATTTCCTTTT

NC_000962.3 3722613 3722635 TBseq_1.0_2173_RIGHT 1 - GGGTTCCTTCTATAACGGGGGT

NC_000962.3 3721952 3721971 TBseq_1.0_2174_LEFT 2 + CAGCCGGCCAATCATCCAG

NC_000962.3 3723968 3723989 TBseq_1.0_2174_RIGHT 2 - AACGAGTTGCCGACGATATGG

NC_000962.3 3723755 3723774 TBseq_1.0_2175_LEFT 1 + GAGAGCCGGTCCTACTGCT

NC_000962.3 3725753 3725776 TBseq_1.0_2175_RIGHT 1 - TGTCATCTGGAAGCTCATCAAGG

NC_000962.3 3725558 3725583 TBseq_1.0_2176_LEFT 2 + GCCACATCTTCTTCAGGTTGAATTC

NC_000962.3 3727490 3727511 TBseq_1.0_2176_RIGHT 2 - GGTGGGTACATCGGAGATCGT

NC_000962.3 3727091 3727111 TBseq_1.0_2177_LEFT 1 + CGACGTGCTTTCGGTCAACT

NC_000962.3 3729130 3729148 TBseq_1.0_2177_RIGHT 1 - ATGGCGCTGCACCGTAGT

NC_000962.3 3753420 3753438 TBseq_1.0_2178_LEFT 2 + AGTGTCTGGGCCCGTGAA

NC_000962.3 3755490 3755510 TBseq_1.0_2178_RIGHT 2 - ATCATGGTGCGCTGGAACAA

NC_000962.3 3767440 3767462 TBseq_1.0_2179_LEFT 1 + ACGTAAGGTTGGGCAAAGTTCC

NC_000962.3 3769414 3769433 TBseq_1.0_2179_RIGHT 1 - ACCGAGGGGCAGTACATCG

NC_000962.3 3768972 3768991 TBseq_1.0_2180_LEFT 2 + GCGAAGAAACGTCTGGCGA

NC_000962.3 3770975 3770994 TBseq_1.0_2180_RIGHT 2 - CGAGTGCCCAGCCTTATCC

NC_000962.3 3770654 3770673 TBseq_1.0_2181_LEFT 1 + CCAGTCTGGCATGCGTGTC

NC_000962.3 3772553 3772576 TBseq_1.0_2181_RIGHT 1 - CACAATGGGTTGCACTTCTTCAC

NC_000962.3 3772041 3772061 TBseq_1.0_2182_LEFT 2 + CATCACAGTCGACGAGGCAC

NC_000962.3 3773945 3773963 TBseq_1.0_2182_RIGHT 2 - ACGGTGGCGATGGACTTC

NC_000962.3 3773286 3773303 TBseq_1.0_2183_LEFT 1 + AGCACGGCGAGCGTGAA

NC_000962.3 3775353 3775376 TBseq_1.0_2183_RIGHT 1 - GGGAGGATAGGTTTCAACAGGAG

NC_000962.3 3774859 3774879 TBseq_1.0_2184_LEFT 2 + CAAGCGGGCTTTCATTGCTG

NC_000962.3 3776868 3776888 TBseq_1.0_2184_RIGHT 2 - GGCAGATGACGATGCAGGAG

NC_000962.3 3780438 3780458 TBseq_1.0_2185_LEFT 1 + ATTCGTCGTAGGGAATGCCG

NC_000962.3 3782354 3782373 TBseq_1.0_2185_RIGHT 1 - TGGTTCAAGCTGCACCACC

NC_000962.3 3781105 3781126 TBseq_1.0_2186_LEFT 2 + GTGGTTCTACTTCGACGGGAC

NC_000962.3 3783026 3783044 TBseq_1.0_2186_RIGHT 2 - ACAAAGACGACTGCGCGG

NC_000962.3 3783029 3783048 TBseq_1.0_2187_LEFT 1 + CGCAGTCGTCTTTGTCCCA

NC_000962.3 3784963 3784982 TBseq_1.0_2187_RIGHT 1 - CGGATCGCGGGACTTGAGA

NC_000962.3 3785715 3785737 TBseq_1.0_2188_LEFT 2 + GTACCCTGGAGAAAACCGATGG

NC_000962.3 3787689 3787709 TBseq_1.0_2188_RIGHT 2 - GCGTCTCGAGTTCTCTGGCA

NC_000962.3 3787217 3787237 TBseq_1.0_2189_LEFT 1 + GGGACAAGGGGAAAACGCTG

NC_000962.3 3789184 3789204 TBseq_1.0_2189_RIGHT 1 - GTCGGAAAGCGAATCGACCC

NC_000962.3 3788966 3788986 TBseq_1.0_2190_LEFT 2 + CATACGACGCGACGGTTGTC

NC_000962.3 3790989 3791008 TBseq_1.0_2190_RIGHT 2 - CCTTGTCGGCGCAGATCAG

NC_000962.3 3790672 3790690 TBseq_1.0_2191_LEFT 1 + ACATCGCCGACTGGGTTG

NC_000962.3 3792658 3792681 TBseq_1.0_2191_RIGHT 1 - ATCGAAGGTTATTACGGCGAGTA

NC_000962.3 3791688 3791707 TBseq_1.0_2192_LEFT 2 + GAGATGTACGCCAAGGCCC

NC_000962.3 3793682 3793701 TBseq_1.0_2192_RIGHT 2 - GCATACACCACCACCGCAT

NC_000962.3 3793489 3793508 TBseq_1.0_2193_LEFT 1 + CGGCAATCGCATCTTCGAC

NC_000962.3 3795454 3795473 TBseq_1.0_2193_RIGHT 1 - AGCAAGCCATCTGGACCCG

NC_000962.3 3795259 3795281 TBseq_1.0_2194_LEFT 2 + GGTTTGATCAGCTCGGTCTTGT

NC_000962.3 3797280 3797299 TBseq_1.0_2194_RIGHT 2 - GAAGGCCCGGTGTATGTGC

NC_000962.3 3796791 3796812 TBseq_1.0_2195_LEFT 1 + AGCAGATGTCTTCAGAGGGGG

NC_000962.3 3798775 3798797 TBseq_1.0_2195_RIGHT 1 - GCAAGATACTCCGAGAAAGGCC

NC_000962.3 3798482 3798504 TBseq_1.0_2196_LEFT 2 + CTCCCATTCTTAACCTCACCGG

NC_000962.3 3800451 3800470 TBseq_1.0_2196_RIGHT 2 - CCAGCAGCGCCTCATTGAG

NC_000962.3 3804889 3804909 TBseq_1.0_2197_LEFT 1 + AGGCCTGCACAGTTCTAGCC

NC_000962.3 3806905 3806926 TBseq_1.0_2197_RIGHT 1 - TGTTCACCAGGTAGTCCACGT

NC_000962.3 3805858 3805875 TBseq_1.0_2198_LEFT 2 + ACTGTGCGGCGGTACAC

NC_000962.3 3807874 3807894 TBseq_1.0_2198_RIGHT 2 - CTGCCCATGTCGTTCACCTT

NC_000962.3 3807397 3807416 TBseq_1.0_2199_LEFT 1 + GCCGAATTCTGCACCAGCT

NC_000962.3 3809430 3809451 TBseq_1.0_2199_RIGHT 1 - CTACGAATGACTTGCGCCGAA

NC_000962.3 3809122 3809143 TBseq_1.0_2200_LEFT 2 + GCTGCGGTTCTACTTCGAGTC

NC_000962.3 3811106 3811126 TBseq_1.0_2200_RIGHT 2 - CGGGTCTGCGGCTATGAGAT

NC_000962.3 3811980 3812001 TBseq_1.0_2201_LEFT 1 + CCCGTGAATTTCATCTGTGCG

NC_000962.3 3813956 3813977 TBseq_1.0_2201_RIGHT 1 - TCGAGAAGCACGGGTTTTCTC

NC_000962.3 3813730 3813748 TBseq_1.0_2202_LEFT 2 + ACCGTATTCGCGAGTGCC

NC_000962.3 3815793 3815813 TBseq_1.0_2202_RIGHT 2 - CGTCTCGGTTGAACTGGTGC

NC_000962.3 3815631 3815652 TBseq_1.0_2203_LEFT 1 + CGTGTGGTGAATCGCAATCCA

NC_000962.3 3817563 3817582 TBseq_1.0_2203_RIGHT 1 - CCCAGGCCATACACCGTCT

NC_000962.3 3817115 3817135 TBseq_1.0_2204_LEFT 2 + GCGGACGACCACATAACTCG

NC_000962.3 3819018 3819041 TBseq_1.0_2204_RIGHT 2 - ACAGGTGAAATAACCCGAAACGC

NC_000962.3 3817790 3817807 TBseq_1.0_2205_LEFT 1 + GAGCACATCGCCGGCAA

NC_000962.3 3819840 3819859 TBseq_1.0_2205_RIGHT 1 - CGTGAACGCGTGACTCTGC

NC_000962.3 3819653 3819670 TBseq_1.0_2206_LEFT 2 + CCGACGCCGCCAACATT

NC_000962.3 3821621 3821642 TBseq_1.0_2206_RIGHT 2 - CCTCATCTGCACGTTGCTACC

NC_000962.3 3821303 3821323 TBseq_1.0_2207_LEFT 1 + GAGCACAATCGGTAGCTCCC

NC_000962.3 3823371 3823391 TBseq_1.0_2207_RIGHT 1 - GTGACCTGGCCGAAATCGTC

NC_000962.3 3822973 3822994 TBseq_1.0_2208_LEFT 2 + TTGCAGATCATGTAGGGCAGC

NC_000962.3 3824927 3824946 TBseq_1.0_2208_RIGHT 2 - GATATCCCGTGGGCCAGCT

NC_000962.3 3824622 3824639 TBseq_1.0_2209_LEFT 1 + AGCCCCCAACACCAGCT

NC_000962.3 3826673 3826693 TBseq_1.0_2209_RIGHT 1 - ACTCTCATCGACTCGACCCG

NC_000962.3 3825802 3825820 TBseq_1.0_2210_LEFT 2 + CGTCACGACCAAACCGCT

NC_000962.3 3827773 3827793 TBseq_1.0_2210_RIGHT 2 - TCAATCCGGACCTGGACCTG

NC_000962.3 3826784 3826806 TBseq_1.0_2211_LEFT 1 + GGTCTAGCTGAAACGATCGGAC

NC_000962.3 3828766 3828786 TBseq_1.0_2211_RIGHT 1 - CTGACGCCACCTGAGAGACT

NC_000962.3 3828029 3828048 TBseq_1.0_2212_LEFT 2 + AACCCCTTCACCGTGGTCA

NC_000962.3 3829928 3829949 TBseq_1.0_2212_RIGHT 2 - CCAACTACTACGCGCGCTAAC

NC_000962.3 3829858 3829878 TBseq_1.0_2213_LEFT 1 + CGCTGAGTTCATAGGTGCGG

NC_000962.3 3831917 3831937 TBseq_1.0_2213_RIGHT 1 - CACTCGTCGCAGCACACTAC

NC_000962.3 3831677 3831702 TBseq_1.0_2214_LEFT 2 + GAACTCAATTTCTGGCGTTATCACC

NC_000962.3 3833757 3833775 TBseq_1.0_2214_RIGHT 2 - ACAGCTGCGCGAACTCAC

NC_000962.3 3833365 3833386 TBseq_1.0_2215_LEFT 1 + CCGTACAGAAACGCCAGGAAT

NC_000962.3 3835419 3835437 TBseq_1.0_2215_RIGHT 1 - TGGTGACTTGGCCGCTGA

NC_000962.3 3834919 3834940 TBseq_1.0_2216_LEFT 2 + CCAACGCAGACATCTGGAACT

NC_000962.3 3836874 3836895 TBseq_1.0_2216_RIGHT 2 - CCTGATGAGCAAGCTGATCGA

NC_000962.3 3836741 3836760 TBseq_1.0_2217_LEFT 1 + GTTGGTAACCGTGGGTCCG

NC_000962.3 3838643 3838662 TBseq_1.0_2217_RIGHT 1 - GCTTGCGCCGGCGATATTA

NC_000962.3 3838271 3838291 TBseq_1.0_2218_LEFT 2 + CCCCCAAGCAGCCGAATATG

NC_000962.3 3840319 3840339 TBseq_1.0_2218_RIGHT 2 - ACGAGGCGATTTTGTTCGGG

NC_000962.3 3839706 3839725 TBseq_1.0_2219_LEFT 1 + GACCAAGTCGCGATCCTGG

NC_000962.3 3841724 3841743 TBseq_1.0_2219_RIGHT 1 - CGACCGCCAAAGCCCATAC

NC_000962.3 3841095 3841113 TBseq_1.0_2220_LEFT 2 + CCGGTGCGGTAATGCCAT

NC_000962.3 3843013 3843036 TBseq_1.0_2220_RIGHT 2 - TCTGCTTGTTCTTTGCTCCTTTC

NC_000962.3 3842133 3842153 TBseq_1.0_2221_LEFT 1 + ACAGCACAACCTTGCACTGA

NC_000962.3 3844138 3844157 TBseq_1.0_2221_RIGHT 1 - AAGCCGCTCGTGCTCATTC

NC_000962.3 3843852 3843871 TBseq_1.0_2222_LEFT 2 + TGGTAGGTCTTAGCGGCCC

NC_000962.3 3845807 3845828 TBseq_1.0_2222_RIGHT 2 - GTAGTGAGGCGCAGATCGATT

NC_000962.3 3845217 3845237 TBseq_1.0_2223_LEFT 1 + GCCAACTTTGAGGTGCGTGT

NC_000962.3 3847140 3847163 TBseq_1.0_2223_RIGHT 1 - TGTTTGTTCTTTGCTCCTTTCGG

NC_000962.3 3846584 3846604 TBseq_1.0_2224_LEFT 2 + ACTATCCGCCCTAGACTGCC

NC_000962.3 3848576 3848594 TBseq_1.0_2224_RIGHT 2 - ACGTACTGGCGGTGCTGA

NC_000962.3 3848356 3848377 TBseq_1.0_2225_LEFT 1 + CGCAGCTTGGTGATATCCCAC

NC_000962.3 3850402 3850421 TBseq_1.0_2225_RIGHT 1 - ACAAGGTCAAGCCGGGTGG

NC_000962.3 3849984 3850001 TBseq_1.0_2226_LEFT 2 + ATATCGCCGGCCGTGGT

NC_000962.3 3852013 3852033 TBseq_1.0_2226_RIGHT 2 - CGGTCTATCTCAATCCGGCC

NC_000962.3 3851788 3851807 TBseq_1.0_2227_LEFT 1 + GGACTACAGGGCAGCCAGA

NC_000962.3 3853861 3853880 TBseq_1.0_2227_RIGHT 1 - GTGTGGCACTTCGTCAGCA

NC_000962.3 3853660 3853680 TBseq_1.0_2228_LEFT 2 + ACAGCAGCAGGTACGGTTCC

NC_000962.3 3855628 3855650 TBseq_1.0_2228_RIGHT 2 - AAAACGTTTCTGGCGCAGATTG

NC_000962.3 3855026 3855046 TBseq_1.0_2229_LEFT 1 + GACGGACTTGGCCAGATTCC

NC_000962.3 3857091 3857109 TBseq_1.0_2229_RIGHT 1 - ACTTGTACGCCGCTTCGC

NC_000962.3 3856981 3857003 TBseq_1.0_2230_LEFT 2 + ACACTTGTCCAACCCATATGCC

NC_000962.3 3858978 3858998 TBseq_1.0_2230_RIGHT 2 - CGCACTTCGAGATTCCGGAC

NC_000962.3 3858819 3858836 TBseq_1.0_2231_LEFT 1 + ACCTGGCGCCGATCCAA

NC_000962.3 3860736 3860755 TBseq_1.0_2231_RIGHT 1 - ATCAACGCCGAACCCAACG

NC_000962.3 3860540 3860560 TBseq_1.0_2232_LEFT 2 + TCACCGGCCTCTTTCATTGC

NC_000962.3 3862529 3862548 TBseq_1.0_2232_RIGHT 2 - TTGACCTGCGTGGATGCTC

NC_000962.3 3862157 3862179 TBseq_1.0_2233_LEFT 1 + GAGTGCCGGTAAACCATCTTGT

NC_000962.3 3864064 3864085 TBseq_1.0_2233_RIGHT 1 - CCAGGTAGGCAGTGTCATTGC

NC_000962.3 3863746 3863766 TBseq_1.0_2234_LEFT 2 + GGTCTTTGCAGGGTTGTCGC

NC_000962.3 3865741 3865759 TBseq_1.0_2234_RIGHT 2 - TGCTCGCCGAACTGGAGT

NC_000962.3 3865450 3865471 TBseq_1.0_2235_LEFT 1 + CCAGCCGTAACTCGATTCGAC

NC_000962.3 3867364 3867383 TBseq_1.0_2235_RIGHT 1 - GCAACGGTATTGGCCCACG

NC_000962.3 3866669 3866691 TBseq_1.0_2236_LEFT 2 + CTGATATGCCGTGACACTGACC

NC_000962.3 3868587 3868609 TBseq_1.0_2236_RIGHT 2 - GGACTTATGCAAGACCAGGACG

NC_000962.3 3868017 3868036 TBseq_1.0_2237_LEFT 1 + ACCACCAACGAGACCAGCA

NC_000962.3 3870082 3870101 TBseq_1.0_2237_RIGHT 1 - ACATAGTCACCGCCGGCAA

NC_000962.3 3868854 3868874 TBseq_1.0_2238_LEFT 2 + GGTGATTGCATGCCGGACAT

NC_000962.3 3870798 3870815 TBseq_1.0_2238_RIGHT 2 - ATCACCTGCCGTGCGGT

NC_000962.3 3870657 3870674 TBseq_1.0_2239_LEFT 1 + ACCTCGCTCAGCCCGTT

NC_000962.3 3872568 3872589 TBseq_1.0_2239_RIGHT 1 - GAGCAAATGTCGGTAGCTGTG

NC_000962.3 3872092 3872110 TBseq_1.0_2240_LEFT 2 + TGCGGCGCACCTGGAATA

NC_000962.3 3874140 3874161 TBseq_1.0_2240_RIGHT 2 - CTTAGCCGGAATCTATGGGGC

NC_000962.3 3873404 3873427 TBseq_1.0_2241_LEFT 1 + ACGCTTTGTCAGTAAGCCCATAA

NC_000962.3 3875376 3875397 TBseq_1.0_2241_RIGHT 1 - CTGGCTCAAGACCACCAGGAT

NC_000962.3 3874697 3874718 TBseq_1.0_2242_LEFT 2 + CGGTACAACATCGACCTGGAT

NC_000962.3 3876662 3876680 TBseq_1.0_2242_RIGHT 2 - ATGCCTTGCCGAACGCTT

NC_000962.3 3876320 3876338 TBseq_1.0_2243_LEFT 1 + AGTCCAGCCGCTGCAGAT

NC_000962.3 3878294 3878313 TBseq_1.0_2243_RIGHT 1 - GGTGCCCGGGGTCAAAGAA

NC_000962.3 3877919 3877939 TBseq_1.0_2244_LEFT 2 + CACGTCCAGGATCAGCTTGT

NC_000962.3 3879894 3879913 TBseq_1.0_2244_RIGHT 2 - TGATCCACCTGCGCGACTA

NC_000962.3 3879227 3879246 TBseq_1.0_2245_LEFT 1 + GTGATTTGCGGGTGACGGG

NC_000962.3 3881201 3881220 TBseq_1.0_2245_RIGHT 1 - GCATGCCCGACTCCGATAC

NC_000962.3 3880997 3881016 TBseq_1.0_2246_LEFT 2 + CAGAAATCGAGGCGCTGGG

NC_000962.3 3882945 3882965 TBseq_1.0_2246_RIGHT 2 - GTCCAAACTGTGACCTGCGA

NC_000962.3 3881499 3881521 TBseq_1.0_2247_LEFT 1 + CCAGGCGCTCGATATGTACTTC

NC_000962.3 3883415 3883434 TBseq_1.0_2247_RIGHT 1 - CGCGCATCTCCCCAATGAA

NC_000962.3 3883043 3883064 TBseq_1.0_2248_LEFT 2 + GCCAAGTATGTGACCTGCGTT

NC_000962.3 3885091 3885111 TBseq_1.0_2248_RIGHT 2 - GGTTCGACCTAAACGCGGTC

NC_000962.3 3886093 3886114 TBseq_1.0_2249_LEFT 1 + CACTCGATCGATGTCTTGCGG

NC_000962.3 3888004 3888025 TBseq_1.0_2249_RIGHT 1 - GGCATATTTGGTGACGGCAGG

NC_000962.3 3887604 3887626 TBseq_1.0_2250_LEFT 2 + ACGTCCAAGAAATGCAGGTACC

NC_000962.3 3889653 3889672 TBseq_1.0_2250_RIGHT 2 - AGGATCTTGTAGCCGGCGG

NC_000962.3 3889388 3889411 TBseq_1.0_2251_LEFT 1 + GAGATACTCAGGATCCAGGCACT

NC_000962.3 3891436 3891455 TBseq_1.0_2251_RIGHT 1 - GGTCATCAGCCGTTCGACG

NC_000962.3 3891277 3891297 TBseq_1.0_2252_LEFT 2 + CTACGACCACATCAACCGGG

NC_000962.3 3893213 3893233 TBseq_1.0_2252_RIGHT 2 - AGTATGTGACGCTGGTGGGT

NC_000962.3 3892501 3892523 TBseq_1.0_2253_LEFT 1 + CCTGATCGCGTTCCTTAAGAGC

NC_000962.3 3894396 3894416 TBseq_1.0_2253_RIGHT 1 - CCTTCCGCTCGAATACCGTC

NC_000962.3 3894069 3894090 TBseq_1.0_2254_LEFT 2 + GGGCTAAATGAGGAGGAGCAC

NC_000962.3 3896117 3896136 TBseq_1.0_2254_RIGHT 2 - GGTGTTTTCTTGTCCCGCG

NC_000962.3 3895662 3895684 TBseq_1.0_2255_LEFT 1 + CTGGCAATCTGGTCTAAGGGAC

NC_000962.3 3897636 3897656 TBseq_1.0_2255_RIGHT 1 - TCGTGGCGAGATCGAATAGC

NC_000962.3 3897311 3897329 TBseq_1.0_2256_LEFT 2 + GTGTGCTAGTGCGGACGA

NC_000962.3 3899354 3899373 TBseq_1.0_2256_RIGHT 2 - ATCCTGGCGACGTTGGGTA

NC_000962.3 3898656 3898676 TBseq_1.0_2257_LEFT 1 + CTCCGGCAGGCTCCTTTTTG

NC_000962.3 3900570 3900595 TBseq_1.0_2257_RIGHT 1 - ACGACTTAAAGACTGGATGGAGAAG

NC_000962.3 3900051 3900072 TBseq_1.0_2258_LEFT 2 + TAGGTCCAGGGCGTGACTATG

NC_000962.3 3901995 3902015 TBseq_1.0_2258_RIGHT 2 - GGACCAGACATCGTCGACCC

NC_000962.3 3901808 3901825 TBseq_1.0_2259_LEFT 1 + TGAGTCGCTTCGGCAGC

NC_000962.3 3903741 3903761 TBseq_1.0_2259_RIGHT 1 - GTAGACGGCATGGTTCAGGC

NC_000962.3 3903281 3903304 TBseq_1.0_2260_LEFT 2 + GGCAACAACATGAACATCTTGCT

NC_000962.3 3905241 3905260 TBseq_1.0_2260_RIGHT 2 - GACATCACCGACGAGGAGC

NC_000962.3 3904854 3904873 TBseq_1.0_2261_LEFT 1 + TGACGGGCACAACCAGATC

NC_000962.3 3906916 3906935 TBseq_1.0_2261_RIGHT 1 - GTCATGTGCGGGCCGAATT

NC_000962.3 3906205 3906222 TBseq_1.0_2262_LEFT 2 + TTGCGGCGCGAGGTGAA

NC_000962.3 3908267 3908287 TBseq_1.0_2262_RIGHT 2 - CGAAGGTCTCCGAATCGCAA

NC_000962.3 3907565 3907589 TBseq_1.0_2263_LEFT 1 + GGAGTTTGCTGTACGTCTATTGGT

NC_000962.3 3909579 3909601 TBseq_1.0_2263_RIGHT 1 - CTTCCTCTATCCCGTCCTTGAC

NC_000962.3 3909008 3909026 TBseq_1.0_2264_LEFT 2 + ACGAGTTGGCGCCTTTCC

NC_000962.3 3911012 3911033 TBseq_1.0_2264_RIGHT 2 - CCCTATCGTCTTCGGGTCATC

NC_000962.3 3910458 3910481 TBseq_1.0_2265_LEFT 1 + GTGCTATTAAATCGGCGTGATGT

NC_000962.3 3912402 3912422 TBseq_1.0_2265_RIGHT 1 - CAAGATCGACCTGCACGACC

NC_000962.3 3912179 3912201 TBseq_1.0_2266_LEFT 2 + TTGGTTCCAACTGGCACATAGC

NC_000962.3 3914116 3914135 TBseq_1.0_2266_RIGHT 2 - TCGAGAATCACCGAGGCCA

NC_000962.3 3914027 3914047 TBseq_1.0_2267_LEFT 1 + GATCTCTTCCAACGCACCGA

NC_000962.3 3916022 3916044 TBseq_1.0_2267_RIGHT 1 - GGACCCGGATTCAATGTCAACG

NC_000962.3 3915614 3915634 TBseq_1.0_2268_LEFT 2 + GGCACCTTGACGTCCTTGGA

NC_000962.3 3917521 3917542 TBseq_1.0_2268_RIGHT 2 - CTGAAAGGCTTTGACGCTGAC

NC_000962.3 3917215 3917237 TBseq_1.0_2269_LEFT 1 + CCGCAACAGTTCCGTAAGATCC

NC_000962.3 3919285 3919305 TBseq_1.0_2269_RIGHT 1 - CCTCGCTGATCGTGGTTGTT

NC_000962.3 3918971 3918993 TBseq_1.0_2270_LEFT 2 + CGCTGTAGCTGATATCCGTGAC

NC_000962.3 3921026 3921048 TBseq_1.0_2270_RIGHT 2 - CGAATCACCACGCCAACATAGG

NC_000962.3 3920316 3920338 TBseq_1.0_2271_LEFT 1 + GTCAGACCGTTGATAAACGCAC

NC_000962.3 3922283 3922302 TBseq_1.0_2271_RIGHT 1 - TGGAACTAGCAGCGACCGG

NC_000962.3 3921707 3921727 TBseq_1.0_2272_LEFT 2 + GATGCCGGCATTGTTCACCA

NC_000962.3 3923613 3923633 TBseq_1.0_2272_RIGHT 2 - ATGATGTCGCGCTGGACTTC

NC_000962.3 3923362 3923383 TBseq_1.0_2273_LEFT 1 + TCCTCAAGCTGATCAACTGGG

NC_000962.3 3925381 3925399 TBseq_1.0_2273_RIGHT 1 - GCTGCACTTCACCGCCTT

NC_000962.3 3936884 3936904 TBseq_1.0_2274_LEFT 2 + CAAGCGATGGCACCACTACA

NC_000962.3 3938898 3938917 TBseq_1.0_2274_RIGHT 2 - TGGTGCCTTGGCTATGGGG

NC_000962.3 3937165 3937187 TBseq_1.0_2275_LEFT 1 + TGGCTCCACAATCCTGAAATCG

NC_000962.3 3939207 3939226 TBseq_1.0_2275_RIGHT 1 - CGCAACGGTTCCTACACGG

NC_000962.3 3951856 3951876 TBseq_1.0_2276_LEFT 2 + TCTCCTGTCGCAAAGTCGGT

NC_000962.3 3953857 3953876 TBseq_1.0_2276_RIGHT 2 - ACATCGGCCAGCTTGAGGT

NC_000962.3 3953433 3953453 TBseq_1.0_2277_LEFT 1 + ATCGAACCATTCCTCGGCAG

NC_000962.3 3955501 3955520 TBseq_1.0_2277_RIGHT 1 - TGACTGAAGCTCCGGACGT

NC_000962.3 3955342 3955362 TBseq_1.0_2278_LEFT 2 + GAGAACAGCTCGGGTTGACG

NC_000962.3 3957350 3957370 TBseq_1.0_2278_RIGHT 2 - TCGTGGAGGCAGGGATGAAG

NC_000962.3 3956272 3956292 TBseq_1.0_2279_LEFT 1 + CGAAGCCATCAGCCCAATCT

NC_000962.3 3958260 3958283 TBseq_1.0_2279_RIGHT 1 - CGTCGGAAACCAAATGCAAAAAC

NC_000962.3 3957521 3957539 TBseq_1.0_2280_LEFT 2 + TGGGGCCCACGTTAAGCA

NC_000962.3 3959560 3959580 TBseq_1.0_2280_RIGHT 2 - CGACATACTTGGTCTGCCCG

NC_000962.3 3958606 3958626 TBseq_1.0_2281_LEFT 1 + GGTTCGTCGGATTACCTGGC

NC_000962.3 3960675 3960693 TBseq_1.0_2281_RIGHT 1 - GTTTCTCGCAGCCGACCA

NC_000962.3 3960122 3960146 TBseq_1.0_2282_LEFT 2 + GATCCAATACGTTTCGATGAGACG

NC_000962.3 3962193 3962211 TBseq_1.0_2282_RIGHT 2 - GTTCAATGCCGTGCTGCG

NC_000962.3 3961882 3961902 TBseq_1.0_2283_LEFT 1 + CCGTTCCTTCAATCGGTCCC

NC_000962.3 3963857 3963877 TBseq_1.0_2283_RIGHT 1 - CCCCAGCCTCTTTACGGTCA

NC_000962.3 3963648 3963667 TBseq_1.0_2284_LEFT 2 + CCGGTCGATGCTACTGCTG

NC_000962.3 3965598 3965619 TBseq_1.0_2284_RIGHT 2 - CATCGATAGCGCTGACATCGT

NC_000962.3 3965315 3965334 TBseq_1.0_2285_LEFT 1 + GGGACCACCTTGATTGCCA

NC_000962.3 3967301 3967319 TBseq_1.0_2285_RIGHT 1 - CATTCGCTGGCCACGGAA

NC_000962.3 3967098 3967119 TBseq_1.0_2286_LEFT 2 + TCGCTGGCCAGGAAAAGAATC

NC_000962.3 3969048 3969072 TBseq_1.0_2286_RIGHT 2 - AGATCAATAATGGGTAGCTCGTGC

NC_000962.3 3968730 3968750 TBseq_1.0_2287_LEFT 1 + GGTTGTCCAGGCCCATCTTC

NC_000962.3 3970631 3970650 TBseq_1.0_2287_RIGHT 1 - CGGTTGCGCTACTACTGCA

NC_000962.3 3970669 3970691 TBseq_1.0_2288_LEFT 2 + CCGGTTATGGCATTCGATACCG

NC_000962.3 3972711 3972733 TBseq_1.0_2288_RIGHT 2 - GGGTCTACTCCAGCTTCCTCAA

NC_000962.3 3972577 3972597 TBseq_1.0_2289_LEFT 1 + CCAGCTCGCGTTTGATTTCC

NC_000962.3 3974515 3974535 TBseq_1.0_2289_RIGHT 1 - TTTGGTCCGGTTGTCGTTCG

NC_000962.3 3973402 3973426 TBseq_1.0_2290_LEFT 2 + TCGAGAAGCCATAGTTGAACGATG

NC_000962.3 3975338 3975361 TBseq_1.0_2290_RIGHT 2 - ACGTTATAGAATTGCTCGGGTCG

NC_000962.3 3975203 3975220 TBseq_1.0_2291_LEFT 1 + ATGGGCGGCGGTCAGTT

NC_000962.3 3977101 3977121 TBseq_1.0_2291_RIGHT 1 - CCAAGAGAATTCGACGGGCG

NC_000962.3 3975824 3975843 TBseq_1.0_2292_LEFT 2 + GCGTATGGCAAGGTTCCGC

NC_000962.3 3977710 3977729 TBseq_1.0_2292_RIGHT 2 - ATCGCCTTGCAGGTCATCC

NC_000962.3 3977823 3977841 TBseq_1.0_2293_LEFT 1 + AACGTGTGGAAGGACGGC

NC_000962.3 3979781 3979801 TBseq_1.0_2293_RIGHT 1 - CAGACCGCAGTCCTCTACGA

NC_000962.3 3980246 3980267 TBseq_1.0_2294_LEFT 2 + GAAAACGAATTGTCCACGCCG

NC_000962.3 3982288 3982307 TBseq_1.0_2294_RIGHT 2 - CAAGCGGGCTCTTGGTGAG

NC_000962.3 3982005 3982024 TBseq_1.0_2295_LEFT 1 + CAGACGCGGCGATCATCTC

NC_000962.3 3983919 3983937 TBseq_1.0_2295_RIGHT 1 - CTGGCAACGCTGGGTCTT

NC_000962.3 3983778 3983798 TBseq_1.0_2296_LEFT 2 + CACAAAGCTAGTGGCAGGCC

NC_000962.3 3985727 3985746 TBseq_1.0_2296_RIGHT 2 - CGAACTGGGTCACGCAACC

NC_000962.3 3985429 3985451 TBseq_1.0_2297_LEFT 1 + GGTGATTCCACGACACTAGCTG

NC_000962.3 3987382 3987402 TBseq_1.0_2297_RIGHT 1 - GTTCCGGTCTATGGGGCCTA

NC_000962.3 3987035 3987053 TBseq_1.0_2298_LEFT 2 + TACTTCCTGCGCGACGGT

NC_000962.3 3989082 3989101 TBseq_1.0_2298_RIGHT 2 - ACAGTGACTCTCGCCGAGG

NC_000962.3 3988408 3988427 TBseq_1.0_2299_LEFT 1 + ATCGGACGCCAAAAACGCT

NC_000962.3 3990393 3990414 TBseq_1.0_2299_RIGHT 1 - GTCACCGAGATTCAAGTGGGC

NC_000962.3 3990092 3990112 TBseq_1.0_2300_LEFT 2 + CAAGCGGGTCTACTACGGGT

NC_000962.3 3992079 3992096 TBseq_1.0_2300_RIGHT 2 - ACCGACGGCAACAGCAG

NC_000962.3 3991497 3991516 TBseq_1.0_2301_LEFT 1 + TGCGGGACAGGGAGATACG

NC_000962.3 3993511 3993530 TBseq_1.0_2301_RIGHT 1 - GCGCAGGTACCGGTAGAAC

NC_000962.3 3992920 3992938 TBseq_1.0_2302_LEFT 2 + CGCGGTCACGTTCGTGAT

NC_000962.3 3994905 3994927 TBseq_1.0_2302_RIGHT 2 - CGATCATCAGGGTCACCAGTGA

NC_000962.3 3994756 3994775 TBseq_1.0_2303_LEFT 1 + CCCATACTTGCGCCGACTG

NC_000962.3 3996661 3996680 TBseq_1.0_2303_RIGHT 1 - GATTTCCTTTGGCGCGCAG

NC_000962.3 3995979 3995998 TBseq_1.0_2304_LEFT 2 + CCGGGTCGGCCTTGATTTC

NC_000962.3 3997875 3997896 TBseq_1.0_2304_RIGHT 2 - CGGAAGAATATGCGCAATCCG

NC_000962.3 4000518 4000540 TBseq_1.0_2305_LEFT 1 + GGCGCGGGTGAATAGATATAGC

NC_000962.3 4002560 4002581 TBseq_1.0_2305_RIGHT 1 - GGTCAGCACGATGTCGATGTC

NC_000962.3 4001196 4001216 TBseq_1.0_2306_LEFT 2 + GGGCTCCGAATATCCCTGAC

NC_000962.3 4003270 4003292 TBseq_1.0_2306_RIGHT 2 - CCAATAACGGTGTTGCGCAAAG

NC_000962.3 4003211 4003230 TBseq_1.0_2307_LEFT 1 + GAGTTCCTCGCCGCCAATG

NC_000962.3 4005135 4005154 TBseq_1.0_2307_RIGHT 1 - CTCGCAGGTGAACCCGATG

NC_000962.3 4004740 4004757 TBseq_1.0_2308_LEFT 2 + ACGCCGGGTGATTGCCA

NC_000962.3 4006784 4006807 TBseq_1.0_2308_RIGHT 2 - TCATCACTGATCAACCGGACATC

NC_000962.3 4005904 4005923 TBseq_1.0_2309_LEFT 1 + TCCAGGCCCTCAAGCATCG

NC_000962.3 4007933 4007952 TBseq_1.0_2309_RIGHT 1 - GGGTTATGTGCTGGCCGAA

NC_000962.3 4007307 4007326 TBseq_1.0_2310_LEFT 2 + CAAGCGGCGACATCGAAGA

NC_000962.3 4009241 4009258 TBseq_1.0_2310_RIGHT 2 - ACGTTCCGCTCCGTGCT

NC_000962.3 4009038 4009060 TBseq_1.0_2311_LEFT 1 + CGTCTTTCTGCTTTTCGGTCAG

NC_000962.3 4010980 4011002 TBseq_1.0_2311_RIGHT 1 - CTGAAACTGCACTACCACGAGG

NC_000962.3 4010200 4010223 TBseq_1.0_2312_LEFT 2 + TGCCACCTCCCAGAAATTCAATC

NC_000962.3 4012257 4012281 TBseq_1.0_2312_RIGHT 2 - GGCAAAAGTAAGTGACGTCCATTC

NC_000962.3 4012199 4012219 TBseq_1.0_2313_LEFT 1 + CGAATCTCCGGCAGCAGATT

NC_000962.3 4014216 4014236 TBseq_1.0_2313_RIGHT 1 - GCTGGCCGAATACCTGATGG

NC_000962.3 4012792 4012811 TBseq_1.0_2314_LEFT 2 + TCGACGCCGATTTCCTCCT

NC_000962.3 4014867 4014886 TBseq_1.0_2314_RIGHT 2 - ACGCCGGAACAGATCGAAC

NC_000962.3 4014915 4014933 TBseq_1.0_2315_LEFT 1 + ACCAGCCGATCACCAGGT

NC_000962.3 4016877 4016901 TBseq_1.0_2315_RIGHT 1 - ACATACTGTCGATGAGCTTTTCGA

NC_000962.3 4016299 4016323 TBseq_1.0_2316_LEFT 2 + TCAAGTCGAAGGCAATAACTCCAG

NC_000962.3 4018264 4018283 TBseq_1.0_2316_RIGHT 2 - ACGGGGAGCTCAAGAACGA

NC_000962.3 4017339 4017358 TBseq_1.0_2317_LEFT 1 + TGTCCGCTGTGCACATCAG

NC_000962.3 4019341 4019361 TBseq_1.0_2317_RIGHT 1 - AGCCATCAGTCATCAGGGCC

NC_000962.3 4019228 4019249 TBseq_1.0_2318_LEFT 2 + CCCAATATCGCGCGGTATTCC

NC_000962.3 4021125 4021145 TBseq_1.0_2318_RIGHT 2 - GGCACTAACACCAGAGTCGC

NC_000962.3 4021366 4021386 TBseq_1.0_2319_LEFT 1 + AGGTTCTGGGGATCTAGCCG

NC_000962.3 4023283 4023302 TBseq_1.0_2319_RIGHT 1 - CATCAGGGCGAAGGTGTGG

NC_000962.3 4022328 4022346 TBseq_1.0_2320_LEFT 2 + ACGCCTGCGCCTTTCTTG

NC_000962.3 4024265 4024285 TBseq_1.0_2320_RIGHT 2 - GTGCTGGCTGGTAGGGTTGT

NC_000962.3 4024039 4024058 TBseq_1.0_2321_LEFT 1 + ATCACCTGCACCACAGCGT

NC_000962.3 4026047 4026066 TBseq_1.0_2321_RIGHT 1 - CAGATCCACCGCTTTGCCT

NC_000962.3 4025660 4025679 TBseq_1.0_2322_LEFT 2 + AGTGCATCGCCGAATCTGG

NC_000962.3 4027613 4027633 TBseq_1.0_2322_RIGHT 2 - CCTACCTCGCCGATCATGAC

NC_000962.3 4027865 4027884 TBseq_1.0_2323_LEFT 1 + CCACTAGGGCGCGGTACAT

NC_000962.3 4029843 4029862 TBseq_1.0_2323_RIGHT 1 - CCGATAACAGGCCACCGTT

NC_000962.3 4029094 4029116 TBseq_1.0_2324_LEFT 2 + TGTACCACGAGATTGTAGGTGC

NC_000962.3 4031067 4031086 TBseq_1.0_2324_RIGHT 2 - CAACTCCATCAGCGCGACC

NC_000962.3 4033565 4033584 TBseq_1.0_2325_LEFT 1 + TGCGTCCAGGAAGTACCGG

NC_000962.3 4035533 4035550 TBseq_1.0_2325_RIGHT 1 - AGGCCACCTGCTTTCGC

NC_000962.3 4036424 4036442 TBseq_1.0_2326_LEFT 2 + AGTTCGACCACCCCCTCA

NC_000962.3 4038491 4038511 TBseq_1.0_2326_RIGHT 2 - GACCTGATGATCAGCCGGGT

NC_000962.3 4039204 4039223 TBseq_1.0_2327_LEFT 1 + GGTGAGCTTGAACACCGGG

NC_000962.3 4041200 4041227 TBseq_1.0_2327_RIGHT 1 - CTATGAGATCGACCTTTCCACTAAGAA

NC_000962.3 4040854 4040879 TBseq_1.0_2328_LEFT 2 + CCGCATAGGAGATAAACGAGAAAAC

NC_000962.3 4042921 4042940 TBseq_1.0_2328_RIGHT 2 - GTGAGTGCCGCTGACACAG

NC_000962.3 4042660 4042681 TBseq_1.0_2329_LEFT 1 + TCATCACTTGCAGCTGGGTAC

NC_000962.3 4044738 4044757 TBseq_1.0_2329_RIGHT 1 - CCGGATCGGGTGTTCTTCG

NC_000962.3 4044330 4044347 TBseq_1.0_2330_LEFT 2 + CGGTGCCGGCGAAAGTT

NC_000962.3 4046398 4046415 TBseq_1.0_2330_RIGHT 2 - GTGGTTTGCATGCGCGG

NC_000962.3 4044775 4044795 TBseq_1.0_2331_LEFT 1 + CTTTAGCACGACCGTCAGCA

NC_000962.3 4046768 4046788 TBseq_1.0_2331_RIGHT 1 - CAACAGGAGCGACCAGTCTG

NC_000962.3 4046895 4046915 TBseq_1.0_2332_LEFT 2 + CCTCCGGCACATCGATGATC

NC_000962.3 4048852 4048871 TBseq_1.0_2332_RIGHT 2 - CCGTTGAGGTGGCGGTACA

NC_000962.3 4048102 4048122 TBseq_1.0_2333_LEFT 1 + GCACCGCCACCAACAGATAT

NC_000962.3 4050018 4050040 TBseq_1.0_2333_RIGHT 1 - CGGACTGTTCAAAACCAATGCC

NC_000962.3 4052661 4052683 TBseq_1.0_2334_LEFT 2 + GTCGGTCTCGTTGTTACCCTTC

NC_000962.3 4054641 4054660 TBseq_1.0_2334_RIGHT 2 - TTCGACGCCGTGGATCTCT

NC_000962.3 4054053 4054074 TBseq_1.0_2335_LEFT 1 + GCTCTATTAATGCTGAGCCGC

NC_000962.3 4056126 4056145 TBseq_1.0_2335_RIGHT 1 - GGCAGACCTCGATCGTCAG

NC_000962.3 4055115 4055136 TBseq_1.0_2336_LEFT 2 + TCCGTCATTGATCGTGTCCCT

NC_000962.3 4057053 4057076 TBseq_1.0_2336_RIGHT 2 - GATTCAGGCTTTTCTTCGCAAGG

NC_000962.3 4056770 4056789 TBseq_1.0_2337_LEFT 1 + GGCTGTGCTAACCCACCAT

NC_000962.3 4058761 4058780 TBseq_1.0_2337_RIGHT 1 - TCGTATTGCAACGCCACGG

NC_000962.3 4058216 4058235 TBseq_1.0_2338_LEFT 2 + CCGACGCCGAACTCAATGG

NC_000962.3 4060255 4060278 TBseq_1.0_2338_RIGHT 2 - AGGAGGACACATGACCATCAACT

NC_000962.3 4060269 4060288 TBseq_1.0_2339_LEFT 1 + TGTCCTCCTGAGTCGTCGG

NC_000962.3 4062252 4062272 TBseq_1.0_2339_RIGHT 1 - AGCCTGTCGTGTGGTCTTCA

NC_000962.3 4060405 4060428 TBseq_1.0_2340_LEFT 2 + CTGATTCATCTGGGTCATGGTGT

NC_000962.3 4062430 4062449 TBseq_1.0_2340_RIGHT 2 - TTGTAGGGCCCGTCGATGC

NC_000962.3 4062986 4063006 TBseq_1.0_2341_LEFT 1 + CGGTCAGCTACTCGATTGGC

NC_000962.3 4064965 4064984 TBseq_1.0_2341_RIGHT 1 - GCAAGGCATTCGTCGACCA

NC_000962.3 4064482 4064503 TBseq_1.0_2342_LEFT 2 + CAACACTGTCTCGGCCTGATC

NC_000962.3 4066400 4066422 TBseq_1.0_2342_RIGHT 2 - GTCTCAGATGATGAACGCCTCC

NC_000962.3 4065978 4065999 TBseq_1.0_2343_LEFT 1 + GAGATGAACGCGAAGGTGAGC

NC_000962.3 4067973 4067993 TBseq_1.0_2343_RIGHT 1 - CCTAACAGTCAGGTCCCCCA

NC_000962.3 4067794 4067816 TBseq_1.0_2344_LEFT 2 + CACTACAAGGACCTGGAACCAG

NC_000962.3 4069796 4069816 TBseq_1.0_2344_RIGHT 2 - CATGATCAGCCAGGCAACCG

NC_000962.3 4068517 4068538 TBseq_1.0_2345_LEFT 1 + AACACTGGCCACTATCACCGG

NC_000962.3 4070493 4070517 TBseq_1.0_2345_RIGHT 1 - CCATTTAAGCCCACAAGTTACACG

NC_000962.3 4070095 4070114 TBseq_1.0_2346_LEFT 2 + CGGATTCGGGCGCTAATCG

NC_000962.3 4072114 4072136 TBseq_1.0_2346_RIGHT 2 - TCGTCATACACCACGACAACAG

NC_000962.3 4071663 4071688 TBseq_1.0_2347_LEFT 1 + GCCTACAGTTCTTAGAGTTACTGCA

NC_000962.3 4073718 4073741 TBseq_1.0_2347_RIGHT 1 - GTCCCAATAGAAATAGCCCCCAA

NC_000962.3 4073684 4073702 TBseq_1.0_2348_LEFT 2 + ACGGTGCAGCTGGTGGTT

NC_000962.3 4075750 4075771 TBseq_1.0_2348_RIGHT 2 - CAATCCTCCACGCTCAACACT

NC_000962.3 4075496 4075518 TBseq_1.0_2349_LEFT 1 + ACGCATACAACAATCAATCGGC

NC_000962.3 4077495 4077517 TBseq_1.0_2349_RIGHT 1 - CAATTGGAAGAACAGGTTGGCG

NC_000962.3 4076865 4076883 TBseq_1.0_2350_LEFT 2 + CCAAACTGCTGCGACGCA

NC_000962.3 4078811 4078828 TBseq_1.0_2350_RIGHT 2 - ATCGGCCCGCTGATGGA

NC_000962.3 4078027 4078047 TBseq_1.0_2351_LEFT 1 + GAGAATCGGCCGGAATCTCG

NC_000962.3 4079947 4079967 TBseq_1.0_2351_RIGHT 1 - GTTCAAGGGGATCCTCGACG

NC_000962.3 4079318 4079337 TBseq_1.0_2352_LEFT 2 + TCGAGTCCGGCACAGATCC

NC_000962.3 4081344 4081366 TBseq_1.0_2352_RIGHT 2 - CTCCATCTTTGCAGGTCAGGTC

NC_000962.3 4080790 4080810 TBseq_1.0_2353_LEFT 1 + AGGATAGTCGACAGCGCTGA

NC_000962.3 4082772 4082794 TBseq_1.0_2353_RIGHT 1 - GAAAGATCACGGAATTGTCGCG

NC_000962.3 4082010 4082027 TBseq_1.0_2354_LEFT 2 + ACGTGCCAGCCCCAAAG

NC_000962.3 4083950 4083969 TBseq_1.0_2354_RIGHT 2 - TTGAGCAGCTGGACCACCT

NC_000962.3 4083375 4083395 TBseq_1.0_2355_LEFT 1 + GTCTGAACGGGTGTTACGGC

NC_000962.3 4085301 4085322 TBseq_1.0_2355_RIGHT 1 - CTTTTTGACGACACCCACGGA

NC_000962.3 4084844 4084863 TBseq_1.0_2356_LEFT 2 + CGTTCTGCGCGGTGATCTC

NC_000962.3 4086781 4086800 TBseq_1.0_2356_RIGHT 2 - CCCGCGACCTAGACATCGA

NC_000962.3 4086247 4086266 TBseq_1.0_2357_LEFT 1 + TTTTCGTACAGCCGCTGGG

NC_000962.3 4088289 4088309 TBseq_1.0_2357_RIGHT 1 - CGCAAAAAGCCCCCAAAACG

NC_000962.3 4087328 4087349 TBseq_1.0_2358_LEFT 2 + TGCCGAATCCCAGGTGAATTC

NC_000962.3 4089327 4089349 TBseq_1.0_2358_RIGHT 2 - ACGATCACAAAGCGGAGATTCC

NC_000962.3 4088516 4088537 TBseq_1.0_2359_LEFT 1 + AGTTCCCTGTGGCATTTCTCG

NC_000962.3 4090560 4090579 TBseq_1.0_2359_RIGHT 1 - ACAGTCACCGGCACCAAAC

NC_000962.3 4090418 4090439 TBseq_1.0_2360_LEFT 2 + GTCGTTGACTCGCTGGATTTC

NC_000962.3 4092354 4092375 TBseq_1.0_2360_RIGHT 2 - TGGGTGTTCCTTGCCAATCAG

NC_000962.3 4090986 4091005 TBseq_1.0_2361_LEFT 1 + CGTGTGTGCAATCCCCCAA

NC_000962.3 4093021 4093043 TBseq_1.0_2361_RIGHT 1 - CCAGTTCAGTTCAGAGCGCATA

NC_000962.3 4092667 4092687 TBseq_1.0_2362_LEFT 2 + CGACGCCATGACAAGAGACC

NC_000962.3 4094581 4094602 TBseq_1.0_2362_RIGHT 2 - TAGCGGGGAGAACCAACTACG

NC_000962.3 4093428 4093447 TBseq_1.0_2363_LEFT 1 + CGCCATGGTGTTTCGGACA

NC_000962.3 4095486 4095505 TBseq_1.0_2363_RIGHT 1 - TACTCGTGGCGCGGATGAC

NC_000962.3 4095054 4095076 TBseq_1.0_2364_LEFT 2 + CAGGTTTGAGTGTGCTGTGACA

NC_000962.3 4097125 4097143 TBseq_1.0_2364_RIGHT 2 - TGCTCTGCACAGCCAGCT

NC_000962.3 4097278 4097297 TBseq_1.0_2365_LEFT 1 + CCCCTAACCTCACCGACCA

NC_000962.3 4099262 4099282 TBseq_1.0_2365_RIGHT 1 - TGCACGTCTGTCCCCAATTG

NC_000962.3 4098608 4098628 TBseq_1.0_2366_LEFT 2 + ATTCAGCCGTCCACCCTGTA

NC_000962.3 4100661 4100686 TBseq_1.0_2366_RIGHT 2 - CGTGGTTCCTTTGTACTACTAGACC

NC_000962.3 4100134 4100154 TBseq_1.0_2367_LEFT 1 + CGAGGACGGCAAGTACACAG

NC_000962.3 4102201 4102227 TBseq_1.0_2367_RIGHT 1 - GTATTTGTTCATCAGCCATGATCTGG

NC_000962.3 4102517 4102538 TBseq_1.0_2368_LEFT 2 + CGAGTACATGGGATCCAGGCT

NC_000962.3 4104433 4104453 TBseq_1.0_2368_RIGHT 2 - TGCTGATTCTTGTCGTGGCG

NC_000962.3 4103719 4103738 TBseq_1.0_2369_LEFT 1 + AGCGTCGCCCATCATCATG

NC_000962.3 4105663 4105685 TBseq_1.0_2369_RIGHT 1 - GCTACATCAACCCGGAATTCGA

NC_000962.3 4105441 4105463 TBseq_1.0_2370_LEFT 2 + CAACATACCAGCCCATGTCAGG

NC_000962.3 4107425 4107445 TBseq_1.0_2370_RIGHT 2 - GTGATCTACGCTCACACCCG

NC_000962.3 4106864 4106886 TBseq_1.0_2371_LEFT 1 + CAACGGCGTCATAGGACATCAG

NC_000962.3 4108825 4108849 TBseq_1.0_2371_RIGHT 1 - ATTGTCACGCCGTATTTTTCGATG

NC_000962.3 4108650 4108671 TBseq_1.0_2372_LEFT 2 + CGATGCGCACCATTTTCGATG

NC_000962.3 4110614 4110633 TBseq_1.0_2372_RIGHT 2 - CATACGAGGCCAGCCAACA

NC_000962.3 4110500 4110524 TBseq_1.0_2373_LEFT 1 + CGTCAGACACTTGAGAACAGATCC

NC_000962.3 4112565 4112585 TBseq_1.0_2373_RIGHT 1 - GAGGCCATCAGACTCAGTGG

NC_000962.3 4111272 4111293 TBseq_1.0_2374_LEFT 2 + TGACAAAACCCCTGTGACACC

NC_000962.3 4113195 4113214 TBseq_1.0_2374_RIGHT 2 - CGGTCATTGGGGTAGGGGT

NC_000962.3 4113190 4113209 TBseq_1.0_2375_LEFT 1 + GCTGCACCCCTACCCCAAT

NC_000962.3 4115100 4115123 TBseq_1.0_2375_RIGHT 1 - CAATGACGACGTTAACCGGAAAG

NC_000962.3 4115106 4115126 TBseq_1.0_2376_LEFT 2 + GGTTAACGTCGTCATTGCGG

NC_000962.3 4117040 4117060 TBseq_1.0_2376_RIGHT 2 - GTGCCTTGTTCACCGTCTCG

NC_000962.3 4116631 4116651 TBseq_1.0_2377_LEFT 1 + CGGGGAAGGTCAAGATCGGT

NC_000962.3 4118611 4118630 TBseq_1.0_2377_RIGHT 1 - AGATCCTCGACCAGTGGGG

NC_000962.3 4118085 4118103 TBseq_1.0_2378_LEFT 2 + TTCCACCGGCGCATCCAA

NC_000962.3 4120105 4120125 TBseq_1.0_2378_RIGHT 2 - CCCGTTCGGGTCCAGAGTAT

NC_000962.3 4119954 4119973 TBseq_1.0_2379_LEFT 1 + CGATTGACCCAGCCAAGCG

NC_000962.3 4121880 4121899 TBseq_1.0_2379_RIGHT 1 - ACCTCCTGTCACTCCACCG

NC_000962.3 4121671 4121690 TBseq_1.0_2380_LEFT 2 + AGTCAGGTGGTCGTGTGCC

NC_000962.3 4123577 4123596 TBseq_1.0_2380_RIGHT 2 - CGTTGCGGTCGATGAGCTG

NC_000962.3 4123308 4123325 TBseq_1.0_2381_LEFT 1 + CAAACACCGCGTTCGCC

NC_000962.3 4125256 4125275 TBseq_1.0_2381_RIGHT 1 - GGCAGCAGAATCTCACCGG

NC_000962.3 4125503 4125521 TBseq_1.0_2382_LEFT 2 + CTGGTGCACGGATGTCGA

NC_000962.3 4127451 4127470 TBseq_1.0_2382_RIGHT 2 - TGAAACGCTGTCTGGGTGC

NC_000962.3 4126516 4126535 TBseq_1.0_2383_LEFT 1 + GCGATAGGCTGTCGTGGCT

NC_000962.3 4128564 4128581 TBseq_1.0_2383_RIGHT 1 - GGATTTCGCGCGGCCTT

NC_000962.3 4128045 4128064 TBseq_1.0_2384_LEFT 2 + TAGCGCACGGTCAACTGCT

NC_000962.3 4129982 4130002 TBseq_1.0_2384_RIGHT 2 - ATCGGCCCAGCATGAAACAG

NC_000962.3 4129488 4129508 TBseq_1.0_2385_LEFT 1 + GCCAAAATCTTCAGCCCCAC

NC_000962.3 4131412 4131430 TBseq_1.0_2385_RIGHT 1 - AGCCGCGATCGCATTACC

NC_000962.3 4130382 4130402 TBseq_1.0_2386_LEFT 2 + CCCGAAACCCGACACTGAGA

NC_000962.3 4132287 4132308 TBseq_1.0_2386_RIGHT 2 - ATCGAGGTCGGCGATCATTTG

NC_000962.3 4132854 4132873 TBseq_1.0_2387_LEFT 1 + GGCCCAGTGACACCTACCA

NC_000962.3 4134862 4134881 TBseq_1.0_2387_RIGHT 1 - CAACTTACGGGTGCTGGCC

NC_000962.3 4134551 4134571 TBseq_1.0_2388_LEFT 2 + TTCTCGACGGAATTCTGGCC

NC_000962.3 4136550 4136570 TBseq_1.0_2388_RIGHT 2 - CGATACCGCTGGTGCTCTTC

NC_000962.3 4136752 4136772 TBseq_1.0_2389_LEFT 1 + ACCCAGAACCCGATCAGCAC

NC_000962.3 4138671 4138691 TBseq_1.0_2389_RIGHT 1 - CAACGGCGGCATGTATTTCG

NC_000962.3 4138434 4138454 TBseq_1.0_2390_LEFT 2 + CGGCCTGGATCTGCATACAC

NC_000962.3 4140494 4140515 TBseq_1.0_2390_RIGHT 2 - AAGAAGGGTGAGATCGTTCGC

NC_000962.3 4138883 4138905 TBseq_1.0_2391_LEFT 1 + GTGTTCAGCAGCAGAAAATTGC

NC_000962.3 4140839 4140860 TBseq_1.0_2391_RIGHT 1 - TCCTCCAGCTCTAGGCAAGTG

NC_000962.3 4140678 4140698 TBseq_1.0_2392_LEFT 2 + CTCACCGGCATGGAGATCGA

NC_000962.3 4142661 4142681 TBseq_1.0_2392_RIGHT 2 - TCGTATGGCGGGAATTCGAC

NC_000962.3 4141073 4141092 TBseq_1.0_2393_LEFT 1 + CTGCCATGCCGGATCCATC

NC_000962.3 4143059 4143078 TBseq_1.0_2393_RIGHT 1 - GGCTATTCGGGCACGCTTG

NC_000962.3 4143507 4143527 TBseq_1.0_2394_LEFT 2 + AGAGTTGGTAACGGACGCAC

NC_000962.3 4145481 4145500 TBseq_1.0_2394_RIGHT 2 - CTGATGAACGCCGACGGTT

NC_000962.3 4144835 4144856 TBseq_1.0_2395_LEFT 1 + CTGCAGGCCAGAGTACACATC

NC_000962.3 4146800 4146819 TBseq_1.0_2395_RIGHT 1 - GACGATGCCGAGCTTTGCT

NC_000962.3 4146788 4146809 TBseq_1.0_2396_LEFT 2 + GGTCATACTGGCAGCAAAGCT

NC_000962.3 4148822 4148840 TBseq_1.0_2396_RIGHT 2 - GTCGGTCGGCAACATCGT

NC_000962.3 4148414 4148435 TBseq_1.0_2397_LEFT 1 + CGCAGCCTGGTTTGAACAAAG

NC_000962.3 4150406 4150423 TBseq_1.0_2397_RIGHT 1 - CGACGGCGGTTGGAACA

NC_000962.3 4149756 4149778 TBseq_1.0_2398_LEFT 2 + GCATGCTTGCCGAGGATATAGC

NC_000962.3 4151795 4151813 TBseq_1.0_2398_RIGHT 2 - GATGCCGGTGCTCAAGGT

NC_000962.3 4151116 4151136 TBseq_1.0_2399_LEFT 1 + TGCGGGACAAGGAAAGACAC

NC_000962.3 4153136 4153157 TBseq_1.0_2399_RIGHT 1 - CAACGCCAAGATCATCGACGT

NC_000962.3 4152460 4152482 TBseq_1.0_2400_LEFT 2 + GTCGTACAGCAGCTGTGAGAAG

NC_000962.3 4154351 4154370 TBseq_1.0_2400_RIGHT 2 - ACTGCGTGCCCGGGTATTT

NC_000962.3 4154098 4154119 TBseq_1.0_2401_LEFT 1 + CGACTTCGACTTCGTCAGAGA

NC_000962.3 4156124 4156141 TBseq_1.0_2401_RIGHT 1 - AACGGCCGGGTGACTCA

NC_000962.3 4155987 4156007 TBseq_1.0_2402_LEFT 2 + CGTGGGTCCGTTTTACCTCG

NC_000962.3 4157933 4157952 TBseq_1.0_2402_RIGHT 2 - CACCCCGTCTGCATGCTTG

NC_000962.3 4156908 4156925 TBseq_1.0_2403_LEFT 1 + TGAATCCAACGCGGGCG

NC_000962.3 4158915 4158934 TBseq_1.0_2403_RIGHT 1 - CTAACGCCCCACCTAACGC

NC_000962.3 4158429 4158449 TBseq_1.0_2404_LEFT 2 + GCACCTACGTCGATATCCGG

NC_000962.3 4160466 4160486 TBseq_1.0_2404_RIGHT 2 - CGTCCAGGACCTGATTGACG

NC_000962.3 4160237 4160258 TBseq_1.0_2405_LEFT 1 + TTGGGTTCCTCGACAATGCAC

NC_000962.3 4162174 4162193 TBseq_1.0_2405_RIGHT 1 - GCTGGCCGACTATGAGACC

NC_000962.3 4161858 4161878 TBseq_1.0_2406_LEFT 2 + CGGCCTGGATCTTCTTCAGC

NC_000962.3 4163860 4163880 TBseq_1.0_2406_RIGHT 2 - CGGTAGCTAAGTAGGTGGCG

NC_000962.3 4163536 4163558 TBseq_1.0_2407_LEFT 1 + GCAAGATCGAGAACAAGGTGAG

NC_000962.3 4165510 4165527 TBseq_1.0_2407_RIGHT 1 - AGCCGAAACACCAGCCC

NC_000962.3 4165340 4165359 TBseq_1.0_2408_LEFT 2 + AGCGTGTTGTCCTCTAGCG

NC_000962.3 4167253 4167270 TBseq_1.0_2408_RIGHT 2 - ATCGGCCCGGACAAGGT

NC_000962.3 4166718 4166738 TBseq_1.0_2409_LEFT 1 + GGTAGAGAGCCACGTCAGCA

NC_000962.3 4168785 4168805 TBseq_1.0_2409_RIGHT 1 - CAGCACGTGAGCCATGATCG

NC_000962.3 4168514 4168534 TBseq_1.0_2410_LEFT 2 + TTCACCAGCCTAAGGAGGGG

NC_000962.3 4170580 4170598 TBseq_1.0_2410_RIGHT 2 - GACGAAACAGCGCCGTGA

NC_000962.3 4170019 4170038 TBseq_1.0_2411_LEFT 1 + ATCGGTCCCCTGTACCAGC

NC_000962.3 4172005 4172024 TBseq_1.0_2411_RIGHT 1 - GACGGCCGGGTGAAAAGTC

NC_000962.3 4171513 4171532 TBseq_1.0_2412_LEFT 2 + CGGTGCGGTATCTGCGGAT

NC_000962.3 4173540 4173560 TBseq_1.0_2412_RIGHT 2 - AAAGAATTCCTGCGCCTGGG

NC_000962.3 4172882 4172900 TBseq_1.0_2413_LEFT 1 + TTTCTCCCGTGGACCGCA

NC_000962.3 4174831 4174856 TBseq_1.0_2413_RIGHT 1 - ACGTTCTTGAAGGATCAGGGATATG

NC_000962.3 4174090 4174112 TBseq_1.0_2414_LEFT 2 + GCAGGGAGAAGGTTTCTGGAAA

NC_000962.3 4175990 4176013 TBseq_1.0_2414_RIGHT 2 - CAACCTTGAGATACCACAGCAGG

NC_000962.3 4175482 4175501 TBseq_1.0_2415_LEFT 1 + TGGGGGTATTCGCTGTGCT

NC_000962.3 4177438 4177459 TBseq_1.0_2415_RIGHT 1 - CGTAGATTTGCGCATCCACCC

NC_000962.3 4176549 4176570 TBseq_1.0_2416_LEFT 2 + GATGTGTCCGATGCGCTCTAC

NC_000962.3 4178598 4178619 TBseq_1.0_2416_RIGHT 2 - CCAGAGTCGGCGAAACAGATC

NC_000962.3 4178235 4178255 TBseq_1.0_2417_LEFT 1 + GTGGACTAGCCCAATGACGC

NC_000962.3 4180245 4180264 TBseq_1.0_2417_RIGHT 1 - GCCACCAGGACGCAGAATC

NC_000962.3 4179271 4179288 TBseq_1.0_2418_LEFT 2 + ACCGACGGGGAGCATGT

NC_000962.3 4181174 4181195 TBseq_1.0_2418_RIGHT 2 - GGGGGTAGAGGGATTCACGTA

NC_000962.3 4180903 4180923 TBseq_1.0_2419_LEFT 1 + TGGTGTAGTCGTCGGGATCC

NC_000962.3 4182910 4182929 TBseq_1.0_2419_RIGHT 1 - CAGATGTTACGCGGCCGAA

NC_000962.3 4182609 4182628 TBseq_1.0_2420_LEFT 2 + AACGAGTTCTCCCGCTGGA

NC_000962.3 4184622 4184642 TBseq_1.0_2420_RIGHT 2 - GCTGGGAATCTCGACTTCGG

NC_000962.3 4184144 4184164 TBseq_1.0_2421_LEFT 1 + TCGACCTCGGGGAACTTACG

NC_000962.3 4186046 4186066 TBseq_1.0_2421_RIGHT 1 - CACCAATACCTCTCAGCCGG

NC_000962.3 4185135 4185155 TBseq_1.0_2422_LEFT 2 + TGCTTGACGCTCTTGATCCG

NC_000962.3 4187121 4187140 TBseq_1.0_2422_RIGHT 2 - TGGGTCGTGGGGTAAGTGC

NC_000962.3 4186788 4186806 TBseq_1.0_2423_LEFT 1 + AATCGAATCGGCCGCAGC

NC_000962.3 4188686 4188705 TBseq_1.0_2423_RIGHT 1 - GCATAGCTCGCGATCGTCA

NC_000962.3 4190257 4190277 TBseq_1.0_2424_LEFT 2 + GCACTGTCGGCACTGGATTT

NC_000962.3 4192176 4192196 TBseq_1.0_2424_RIGHT 2 - AGCACGCCATGGAACTGATG

NC_000962.3 4190717 4190739 TBseq_1.0_2425_LEFT 1 + TCATTTCCACGAGCTACGATGC

NC_000962.3 4192663 4192683 TBseq_1.0_2425_RIGHT 1 - GGTCTACAACCCATGGGACC

NC_000962.3 4193034 4193051 TBseq_1.0_2426_LEFT 2 + TCGCCTTCTCGTGCTGC

NC_000962.3 4194967 4194985 TBseq_1.0_2426_RIGHT 2 - ACTCGGTGCGTGGGCTAA

NC_000962.3 4194991 4195008 TBseq_1.0_2427_LEFT 1 + TGCGGGCCGTTACCAGT

NC_000962.3 4196998 4197015 TBseq_1.0_2427_RIGHT 1 - TAACCGGCAGGCGCAGT

NC_000962.3 4196688 4196706 TBseq_1.0_2428_LEFT 2 + ATGTTGTCCACCTGCGGC

NC_000962.3 4198612 4198633 TBseq_1.0_2428_RIGHT 2 - AACGGACAGTACTTCCACCCC

NC_000962.3 4197164 4197183 TBseq_1.0_2429_LEFT 1 + GCAGCATCCCTACAACGCA

NC_000962.3 4199105 4199129 TBseq_1.0_2429_RIGHT 1 - CAAATCCCTTTGACTGTTAGTGCA

NC_000962.3 4198710 4198729 TBseq_1.0_2430_LEFT 2 + AAGCTACTGCGCCGCTAAC

NC_000962.3 4200767 4200787 TBseq_1.0_2430_RIGHT 2 - GGGCTCTGTTGAACAAGCCG

NC_000962.3 4200567 4200584 TBseq_1.0_2431_LEFT 1 + GTGCTGGCCGTTCCGAT

NC_000962.3 4202563 4202584 TBseq_1.0_2431_RIGHT 1 - CCTGCTAACCGCCAGTAACTG

NC_000962.3 4201855 4201874 TBseq_1.0_2432_LEFT 2 + GCGCGGAGTCAGATCAGAG

NC_000962.3 4203823 4203842 TBseq_1.0_2432_RIGHT 2 - GACGCACGACATCGACGAG

NC_000962.3 4203317 4203340 TBseq_1.0_2433_LEFT 1 + GCCTTCTTGCCTTTTTGGAACTC

NC_000962.3 4205338 4205356 TBseq_1.0_2433_RIGHT 1 - CCTACGTCGCGCAACTGT

NC_000962.3 4204606 4204626 TBseq_1.0_2434_LEFT 2 + ACAAGCGGCACAATGTTTCC

NC_000962.3 4206592 4206612 TBseq_1.0_2434_RIGHT 2 - GCCGTTCCAGGTTGTCGAAT

NC_000962.3 4206139 4206158 TBseq_1.0_2435_LEFT 1 + TTAAGTCCAGCGCGGGGTC

NC_000962.3 4208026 4208045 TBseq_1.0_2435_RIGHT 1 - GCCGAAAACGCCACGCATA

NC_000962.3 4207666 4207686 TBseq_1.0_2436_LEFT 2 + CAAACCAGCCCATGTAACGC

NC_000962.3 4209688 4209707 TBseq_1.0_2436_RIGHT 2 - ATCGTCTCCGCTGTGGTCA

NC_000962.3 4208862 4208884 TBseq_1.0_2437_LEFT 1 + TGTTCCATCGGCACTACATTGC

NC_000962.3 4210905 4210926 TBseq_1.0_2437_RIGHT 1 - CCGCTCGGTGTTGATGTATCC

NC_000962.3 4210389 4210408 TBseq_1.0_2438_LEFT 2 + CGGACCGGTAGCTTGACCT

NC_000962.3 4212345 4212362 TBseq_1.0_2438_RIGHT 2 - ACCAATTCGGACGGGCG

NC_000962.3 4211812 4211833 TBseq_1.0_2439_LEFT 1 + TGCGTCACAGCGTCAAGTATC

NC_000962.3 4213803 4213822 TBseq_1.0_2439_RIGHT 1 - CGTTCGCGCGGATCTTTGT

NC_000962.3 4212914 4212934 TBseq_1.0_2440_LEFT 2 + AGGACCTCACTCCGGTTGAC

NC_000962.3 4214870 4214889 TBseq_1.0_2440_RIGHT 2 - AATCACTTGTCGAGGGCGC

NC_000962.3 4214045 4214066 TBseq_1.0_2441_LEFT 1 + GTCTACAGGTTGCAGAGGAGC

NC_000962.3 4216098 4216118 TBseq_1.0_2441_RIGHT 1 - GGATCAGCCGGCATACCATC

NC_000962.3 4216463 4216480 TBseq_1.0_2442_LEFT 2 + CGTGGACAGCGCGAACT

NC_000962.3 4218452 4218473 TBseq_1.0_2442_RIGHT 2 - TCTCGGTCTTCTCGGTCGAAT

NC_000962.3 4218193 4218213 TBseq_1.0_2443_LEFT 1 + GAGCGTGGCCCGTAAGAAAA

NC_000962.3 4220172 4220192 TBseq_1.0_2443_RIGHT 1 - CCACCGCGTATTCATGGTCG

NC_000962.3 4219277 4219294 TBseq_1.0_2444_LEFT 2 + TCGGTGCGCGAGGTCAA

NC_000962.3 4221349 4221369 TBseq_1.0_2444_RIGHT 2 - AGTCGGTTTAGGGTGAGGCT

NC_000962.3 4220823 4220843 TBseq_1.0_2445_LEFT 1 + GGGCGGTTTGATCAGTAGCG

NC_000962.3 4222855 4222876 TBseq_1.0_2445_RIGHT 1 - GATGATGTCGCTTACTCCCGG

NC_000962.3 4222374 4222398 TBseq_1.0_2446_LEFT 2 + CGACAGCCGAATCTTCTTACCTAG

NC_000962.3 4224348 4224367 TBseq_1.0_2446_RIGHT 2 - CGGGCGATGACCAAACTGG

NC_000962.3 4224203 4224223 TBseq_1.0_2447_LEFT 1 + CGGAAAACCATCGCCCCAAT

NC_000962.3 4226193 4226217 TBseq_1.0_2447_RIGHT 1 - GATCTTCTTGACCAGCAGAATGAG

NC_000962.3 4225003 4225027 TBseq_1.0_2448_LEFT 2 + CGCTAATCGCTTTGATTTTGCTGA

NC_000962.3 4226966 4226987 TBseq_1.0_2448_RIGHT 2 - TTCTAGGAGTGTGTTGCTGCG

NC_000962.3 4226802 4226823 TBseq_1.0_2449_LEFT 1 + CTCGAGGCCATCCAAGTCCTC

NC_000962.3 4228707 4228729 TBseq_1.0_2449_RIGHT 1 - TCGTCTATGTTGCACACCATCG

NC_000962.3 4228341 4228364 TBseq_1.0_2450_LEFT 2 + CAGGGATGACTGAATCGGTCTTC

NC_000962.3 4230295 4230315 TBseq_1.0_2450_RIGHT 2 - CAGTGACTCGGTCAGATGGC

NC_000962.3 4228783 4228803 TBseq_1.0_2451_LEFT 1 + AAGTGAATTGCGCACCGAGG

NC_000962.3 4230766 4230786 TBseq_1.0_2451_RIGHT 1 - GATGTTGAACGGACGGACGA

NC_000962.3 4230988 4231008 TBseq_1.0_2452_LEFT 2 + GGAAAGATACCCGGGTGAGG

NC_000962.3 4232985 4233007 TBseq_1.0_2452_RIGHT 2 - CTTGAGGGTGGAGCACTATCTG

NC_000962.3 4232215 4232236 TBseq_1.0_2453_LEFT 1 + CGGGATACAGGTGGAGACAGT

NC_000962.3 4234296 4234315 TBseq_1.0_2453_RIGHT 1 - GGGCTTGCAGCGAATGACG

NC_000962.3 4233095 4233112 TBseq_1.0_2454_LEFT 2 + GCCAATGCGGGTTGTGC

NC_000962.3 4235126 4235146 TBseq_1.0_2454_RIGHT 2 - TCAGGGTTTCGGCTTCTCGG

NC_000962.3 4234793 4234812 TBseq_1.0_2455_LEFT 1 + CGAGTCAAAGGGGCTAGCG

NC_000962.3 4236846 4236868 TBseq_1.0_2455_RIGHT 1 - TGAGAAACGAGTAGTGACCCGA

NC_000962.3 4236274 4236294 TBseq_1.0_2456_LEFT 2 + AGGACGCCGAACTGTTCTGG

NC_000962.3 4238285 4238308 TBseq_1.0_2456_RIGHT 2 - GAGATGCCGAACAGATACAGCTT

NC_000962.3 4237834 4237855 TBseq_1.0_2457_LEFT 1 + GTAAGGAATTGGTTTGGGCGC

NC_000962.3 4239898 4239918 TBseq_1.0_2457_RIGHT 1 - ACGGAGGTAGATGGTAGCCG

NC_000962.3 4239231 4239249 TBseq_1.0_2458_LEFT 2 + AAGCGGTCCAAGCCCTTG

NC_000962.3 4241259 4241278 TBseq_1.0_2458_RIGHT 2 - CAGTGGTAGCACGCCGAAC

NC_000962.3 4243016 4243036 TBseq_1.0_2459_LEFT 1 + ACTGGTTTAGGGACTGGGGC

NC_000962.3 4245079 4245097 TBseq_1.0_2459_RIGHT 1 - TTGATGCCCGAGGTTGCC

NC_000962.3 4244792 4244812 TBseq_1.0_2460_LEFT 2 + GTGCTGGTGTTGCTGTTCTG

NC_000962.3 4246838 4246859 TBseq_1.0_2460_RIGHT 2 - CGAACAACGCCTGCAAATTGG

NC_000962.3 4246535 4246555 TBseq_1.0_2461_LEFT 1 + GCAAAAGCACCCCAAATCGG

NC_000962.3 4248445 4248465 TBseq_1.0_2461_RIGHT 1 - CTGGAGACATACCACCAGCC

NC_000962.3 4247672 4247690 TBseq_1.0_2462_LEFT 2 + CGGCCATGGTCTTGCTGA

NC_000962.3 4249651 4249671 TBseq_1.0_2462_RIGHT 2 - AGGTAGGTGGCCATGACGTG

NC_000962.3 4249095 4249116 TBseq_1.0_2463_LEFT 1 + GTGGTATCTCCTGCCTAAGCC

NC_000962.3 4251033 4251054 TBseq_1.0_2463_RIGHT 1 - TGTCTAGCTCTAGGCAGCGAT

NC_000962.3 4250516 4250535 TBseq_1.0_2464_LEFT 2 + ACGGAGATCGGGCTACCAC

NC_000962.3 4252549 4252566 TBseq_1.0_2464_RIGHT 2 - AATGCGGCGCGAGACCA

NC_000962.3 4252352 4252372 TBseq_1.0_2465_LEFT 1 + CGGCAATGGCTACATCGAGA

NC_000962.3 4254346 4254366 TBseq_1.0_2465_RIGHT 1 - GCAGACGTAAAAGCCCCCAA

NC_000962.3 4253690 4253710 TBseq_1.0_2466_LEFT 2 + CTGCCCGGATGCGATTCAAT

NC_000962.3 4255651 4255671 TBseq_1.0_2466_RIGHT 2 - TGTTCTCGCACGACCAAACC

NC_000962.3 4255195 4255216 TBseq_1.0_2467_LEFT 1 + GGACGGCAGAAACGACAGAAA

NC_000962.3 4257167 4257186 TBseq_1.0_2467_RIGHT 1 - ACCCAGACCGCTGAGGAGA

NC_000962.3 4257316 4257335 TBseq_1.0_2468_LEFT 2 + TCGACGCGGTTTTTGATGC

NC_000962.3 4259284 4259303 TBseq_1.0_2468_RIGHT 2 - TTGCGGCCCAAGAGGTTAC

NC_000962.3 4258569 4258592 TBseq_1.0_2469_LEFT 1 + GAACACTTCTCTGATTTCGTCGG

NC_000962.3 4260589 4260610 TBseq_1.0_2469_RIGHT 1 - CCTGAAGGACATCAAGGGCTT

NC_000962.3 4259756 4259775 TBseq_1.0_2470_LEFT 2 + GGTGCCCGACGTTGGTTTT

NC_000962.3 4261795 4261814 TBseq_1.0_2470_RIGHT 2 - AACTGCCGGACGGACAGAT

NC_000962.3 4261539 4261560 TBseq_1.0_2471_LEFT 1 + GCACTCGAGATCCTGTGGGTA

NC_000962.3 4263458 4263479 TBseq_1.0_2471_RIGHT 1 - AGCCGGTCCATGCTATGTATG

NC_000962.3 4263158 4263178 TBseq_1.0_2472_LEFT 2 + CAGTTTGCAGCCAGCTTTGA

NC_000962.3 4265224 4265245 TBseq_1.0_2472_RIGHT 2 - ATGTCAGTAACTGGGTCACCG

NC_000962.3 4265569 4265593 TBseq_1.0_2473_LEFT 1 + ACGGGATTTAGGAAACATCTAGCG

NC_000962.3 4267623 4267642 TBseq_1.0_2473_RIGHT 1 - GCCACCCGGGTCACCTATT

NC_000962.3 4267357 4267380 TBseq_1.0_2474_LEFT 2 + CATGCCCATGTTGGTGAAAAACA

NC_000962.3 4269310 4269331 TBseq_1.0_2474_RIGHT 2 - CCAAAATCCCGCTGTCCAAGT

NC_000962.3 4269173 4269193 TBseq_1.0_2475_LEFT 1 + GAACCGCAGATAGGTGCTGG

NC_000962.3 4271066 4271087 TBseq_1.0_2475_RIGHT 1 - CCTGGAGCGATAAGGATGACG

NC_000962.3 4270675 4270694 TBseq_1.0_2476_LEFT 2 + GCGGTCAGGTTGTGCATGA

NC_000962.3 4272712 4272731 TBseq_1.0_2476_RIGHT 2 - CGCTACTTCGACTACGCCG

NC_000962.3 4271607 4271627 TBseq_1.0_2477_LEFT 1 + GATGGAGAGCCGACTACCCA

NC_000962.3 4273619 4273642 TBseq_1.0_2477_RIGHT 1 - TGTGGTTGATGTGACTAGAGTGG

NC_000962.3 4273500 4273519 TBseq_1.0_2478_LEFT 2 + CCCGATTTGGCAGCTCGTG

NC_000962.3 4275488 4275508 TBseq_1.0_2478_RIGHT 2 - CGGAGAGTAGTCGTTGCTCC

NC_000962.3 4274623 4274642 TBseq_1.0_2479_LEFT 1 + CCAAGGCGTCGGTCATTGC

NC_000962.3 4276516 4276535 TBseq_1.0_2479_RIGHT 1 - ATCAGCCCGGCAAATGGAG

NC_000962.3 4278424 4278442 TBseq_1.0_2480_LEFT 2 + ACGCCGTCCTCGCTATTG

NC_000962.3 4280349 4280368 TBseq_1.0_2480_RIGHT 2 - GATGTCCCGATTGTGCCCG

NC_000962.3 4279795 4279816 TBseq_1.0_2481_LEFT 1 + CTTGCATCTCGGCCTTGATCA

NC_000962.3 4281776 4281796 TBseq_1.0_2481_RIGHT 1 - CTTGATGAACTCGCAGCCGG

NC_000962.3 4281063 4281082 TBseq_1.0_2482_LEFT 2 + CCTTCGGGCGAAACAGCTT

NC_000962.3 4283032 4283049 TBseq_1.0_2482_RIGHT 2 - GCTTTTGGGCGCCGAAC

NC_000962.3 4282735 4282759 TBseq_1.0_2483_LEFT 1 + AGGATTTCATCGGTTCTTCGAAAC

NC_000962.3 4284777 4284796 TBseq_1.0_2483_RIGHT 1 - GTCGCGCAGATCTCCTTCG

NC_000962.3 4284555 4284578 TBseq_1.0_2484_LEFT 2 + GCTGATACCGATTGACCCACATC

NC_000962.3 4286535 4286554 TBseq_1.0_2484_RIGHT 2 - TGCCAGGTAGCTGACGAGG

NC_000962.3 4286480 4286499 TBseq_1.0_2485_LEFT 1 + AGGCACTGGCTGTCGTTAC

NC_000962.3 4288435 4288453 TBseq_1.0_2485_RIGHT 1 - TGTTGGTTGGGCAGGCGA

NC_000962.3 4288112 4288131 TBseq_1.0_2486_LEFT 2 + GTCGAGCGTGAACTGAGGG

NC_000962.3 4290102 4290121 TBseq_1.0_2486_RIGHT 2 - CTCTCGCCGACCTAGAGCA

NC_000962.3 4289639 4289657 TBseq_1.0_2487_LEFT 1 + CGGATCGGCAGTGCACAT

NC_000962.3 4291690 4291710 TBseq_1.0_2487_RIGHT 1 - TGTCGCGCGGGTGATTTTAT

NC_000962.3 4291453 4291474 TBseq_1.0_2488_LEFT 2 + TTGGCCTAAGCGGTTTCGATC

NC_000962.3 4293379 4293403 TBseq_1.0_2488_RIGHT 2 - TACCCGAGTATGGACTTGATTCGT

NC_000962.3 4293136 4293159 TBseq_1.0_2489_LEFT 1 + TCTTTGACCGCTGACAACTCTAC

NC_000962.3 4295131 4295150 TBseq_1.0_2489_RIGHT 1 - CAGCTGGGCACGGATTTCG

NC_000962.3 4294785 4294805 TBseq_1.0_2490_LEFT 2 + CGCAATAGCTCTCGACGCTG

NC_000962.3 4296753 4296773 TBseq_1.0_2490_RIGHT 2 - GGCGATCACCGGATACACAA

NC_000962.3 4296494 4296513 TBseq_1.0_2491_LEFT 1 + CTCGCATTCGCCGGATACC

NC_000962.3 4298472 4298491 TBseq_1.0_2491_RIGHT 1 - CCGGGGTGCTAGGGCTAAT

NC_000962.3 4297410 4297429 TBseq_1.0_2492_LEFT 2 + TCTCGCCGCTCCCATATGT

NC_000962.3 4299391 4299412 TBseq_1.0_2492_RIGHT 2 - GGGATTGCGACGAGTTCTACG

NC_000962.3 4298572 4298595 TBseq_1.0_2493_LEFT 1 + TTGCATATTCAATGGGGTCACCG

NC_000962.3 4300540 4300557 TBseq_1.0_2493_RIGHT 1 - TGCAGAAACGCCACCGG

NC_000962.3 4300248 4300271 TBseq_1.0_2494_LEFT 2 + ACTAGATCTGGACTCGCCGATAC

NC_000962.3 4302215 4302234 TBseq_1.0_2494_RIGHT 2 - GTGCTGGCAATCTCGGTGC

NC_000962.3 4301837 4301859 TBseq_1.0_2495_LEFT 1 + CAGCGTCCAAACCTTCAACAAC

NC_000962.3 4303908 4303927 TBseq_1.0_2495_RIGHT 1 - AGGTTCAGCAGCAGTGGGA

NC_000962.3 4303693 4303714 TBseq_1.0_2496_LEFT 2 + TCGGTGCCAACCTAGTGATCT

NC_000962.3 4305733 4305750 TBseq_1.0_2496_RIGHT 2 - AGTCTCGCCGTGGCCAA

NC_000962.3 4304709 4304730 TBseq_1.0_2497_LEFT 1 + GTGAATTGGACCACCGGATCG

NC_000962.3 4306767 4306786 TBseq_1.0_2497_RIGHT 1 - GTCATTGCAGCTCGGCAGG

NC_000962.3 4305915 4305940 TBseq_1.0_2498_LEFT 2 + TGGTACTTCAACATTCGTTTTGTGC

NC_000962.3 4307916 4307934 TBseq_1.0_2498_RIGHT 2 - CCGCAAGTTCGACTGCGA

NC_000962.3 4307215 4307234 TBseq_1.0_2499_LEFT 1 + ACGAGCACCACCGGATGTT

NC_000962.3 4309208 4309225 TBseq_1.0_2499_RIGHT 1 - GTGACCAGCCCGGCAAT

NC_000962.3 4309028 4309049 TBseq_1.0_2500_LEFT 2 + CCTGTCAGAATGGAGCGGATG

NC_000962.3 4310935 4310959 TBseq_1.0_2500_RIGHT 2 - GCATTGCAGTCTAGGACGAAGATA

NC_000962.3 4310685 4310705 TBseq_1.0_2501_LEFT 1 + ATCCATGAGGTCGCCCATCA

NC_000962.3 4312717 4312741 TBseq_1.0_2501_RIGHT 1 - AGCCTAAGTTAACTAATGCACGGT

NC_000962.3 4312488 4312506 TBseq_1.0_2502_LEFT 2 + GCACGCACGCATAATCCG

NC_000962.3 4314466 4314486 TBseq_1.0_2502_RIGHT 2 - GGTGACTGTGCGTTCCTGAT

NC_000962.3 4313764 4313785 TBseq_1.0_2503_LEFT 1 + CTGCACCTGGATCACTATGCC

NC_000962.3 4315812 4315832 TBseq_1.0_2503_RIGHT 1 - CGTCAGCAACGCGATATCGA

NC_000962.3 4315549 4315570 TBseq_1.0_2504_LEFT 2 + CGGCCCATGTCATGTCCACTA

NC_000962.3 4317590 4317611 TBseq_1.0_2504_RIGHT 2 - GACGGGAGGTTATTCGAGTGC

NC_000962.3 4317039 4317059 TBseq_1.0_2505_LEFT 1 + CCCCACCATTCACCAGGAGT

NC_000962.3 4319007 4319026 TBseq_1.0_2505_RIGHT 1 - GTCACCGGCATTCTCGACA

NC_000962.3 4318293 4318314 TBseq_1.0_2506_LEFT 2 + GGACCGGTTGATCTTCTAGGC

NC_000962.3 4320258 4320278 TBseq_1.0_2506_RIGHT 2 - AGTGGATCGACGCCCAAAAC

NC_000962.3 4319887 4319906 TBseq_1.0_2507_LEFT 1 + GTCGCGCAACACAATCCGA
[truncated: 8,574 more chars]
